# Supplementary material for: Genetic diversity and relationship of Bugesera and Rwamagana indigenous chicken populations with SASSO chickens using DArTseq SNPs
Source: PLoS One. 2025 Sep 12;20(9):e0331316. doi: 10.1371/journal.pone.0331316 (PMC12431124; doi:10.1371/journal.pone.0331316)
Supplement: S1 Checklist — (PDF) [file pone.0331316.s001.pdf]

Inclusivity in global research

PLOS' policy on inclusivity in global research aims to improve transparency in the reporting of research performed outside of researchers' own country or community and ensures that PLOS publications reporting global research adhere to high standards for research ethics and authorship. Authors of relevant research articles may be asked to complete the questionnaire below, which outlines ethical, cultural, and scientific considerations specific to inclusivity in global research. This questionnaire may be requested when researchers have travelled to a different country to conduct research, if research uses samples collected in another country, research with Indigenous populations or their lands, or if research is on cultural artefacts. Researchers travelling to another country solely to use laboratory equipment will not normally be required to complete the questionnaire. However, the questionnaire can be requested at the journal's discretion for any submission – if you have been requested to complete this questionnaire by the PLOS journal you submitted to, please do so.

Please complete the questionnaire below and include this as a Supporting Information file with your manuscript. Note that if your paper is accepted for publication, this checklist will be published with your article in the supporting information files. Please ensure that you reference the checklist in the main body of your manuscript. We suggest adding a subsection 'Inclusivity in global research' to your Methods section and adding the following sentence: "Additional information regarding the ethical, cultural, and scientific considerations specific to inclusivity in global research is included in the Supporting Information (SX Checklist)"

The questions have been designed to be applicable to a wide range of study types, and there are subsections for both human subjects research and non-human subjects research. If any of the questions are not relevant to your research please mark them as "N/A" as appropriate.

Ethical considerations, permits and authorship

This section is applicable to all research types.

Provide details as to who granted permissions and/or consent for the study to take place in the Methods section of your manuscript. This should include the names of **all** ethics boards, governmental organizations, community leaders or other bodies that provided approval for the study. If individuals provided approval refer to these people by their role or title but do not list their name(s).

Reported on page number: Page 7: L183 – L187

If there were any deviations from the study protocol after approval was obtained please provide details of these changes in the Methods section of your manuscript.

N/A

Did this study involve local collaborators that are residents of the country where the research was conducted or members of the community studied? If you do not have any authors from said communities, please provide an explanation for this below.

Yes

Everyone listed as an author should meet PLOS' criteria for authorship and all individuals who meet these criteria should be included in the author byline, rather than the acknowledgements. For further information please see the journal's Authorship Policy.

Human subjects research (e.g. health research, medical research, cross-cultural psychology)

Did you obtain written informed consent from a representative of the local community or region before the research took place? How did you establish who speaks for the community? Details of written informed consent obtained from study participants should be reported separately in the Methods section of your manuscript.

Yes, the rwiten informed consent from participants was obtained. Every participant spoke to researcher her or himself. The statement of written informed consent was provided on page 7: L192 – L196.

How did members of the local community provide input on the aims of the research investigation, its methodology, and its anticipated outcome(s)?

They provided the research materials (blood samples from their indigenous chickens) used for genotyping.

When engaging with the local community, how did you ensure that the informed consent documents and other materials could be understood by local stakeholders?

The documents used were translated and written in local language, and the researcher himself interacted with the participants engaged in the study and tried to answer to all quastions raised.

Will the findings of the research be made available in an understandable format to stakeholders in the community where the study was conducted (e.g. via a presentation, summary report, copies of publications, etc.)? Please provide details of how this will be achieved.

Findings will reach the local community through community outreach activities (trainings and workshops) focusing on the genetic improvement of indigenous chickens, and the reports and publications will be available in public libraries and accessible to all who will be interested in the study. All materials will be in languages used in the country so that every one can have access to information

Non-human subjects research using specimens/ animals collected as part of the study, or those housed in archival collections. Examples include archaeology, paleontology, botany and zoology.

Did the permission you obtained from a local authority to perform the study include an agreement on access to outputs and benefit sharing? This may include procedures to enable fair distribution of the benefits and resources arising from the research performed. Please include any details of Prior Informed Consent and Benefit Sharing Agreements obtained. These may be required by field-specific regulations, for example the Convention on Biological Diversity (CBD) and the associated Nagoya Protocol.

Yes, the permission included agreement on access to outputs and benefit sharing. This permission followed the Nagoya Protocol regarding the Convention on Biological Diversity. Information included in Prior Informed Consent were: the title, parties involved in the study, research description, the study area, resources to have access to, study procedures and study period, the assurance of non-commercial use, and confidentiality. In addition to title and parties involved, the scope of study, types of benefits to be shared, benefit delivery, evaluation and monitoring, dispute resolution were included in the documents of Benefit Sharing Agreement.

If the material used in your study was imported, please A) provide the year it was imported and B) indicate whether permits were obtained to import/export the materials used, C) provide details of any permits obtained. If this information is not available, please indicate this.

The material used (blood sample) for geneotyping was exported outside the country, from Rwanda to Kenya (ILRI). The import permits from the Republich of Kenya were obtained on August 23rd, 2019 before sample shipping . Two types of import permit were required: Import permit for veterinary drugs vaccines accessories biological materials and Samples for Clinical Trials Import Permit. The first reflected the application approval, applicant details (ILRI), consignee details (ILRI), importer details (ILRI), exporter details (Valentin MUJYAMBERE, consignor details (Valantin MUJYAMBERE), meetting regulatory conditions, required documents for approval, purpose of import/export, terms and conditions,description of the material, transport details, and the name and signature of organ in charge in Kenya (DIRECTORATE OF VETERINARY SERVICES). The information in the second permit included application approval, applicant details (ILRI), consignee details (ILRI), importer details (ILRI), exporte details (Valentin MUJYAMBERE), consignor details (Valentin MUJYAMBERE), conditions of approval, purpose of import/export (research), terms and conditions (ethical committee approval letter), description of the material (blood sample), transport details, the name of organization in charge in Kenya (PHARMACY AND POISONS BOARD).

If you used archival specimens, please state how the material used in your study was acquired by the institute it is held in and provide details of any permits obtained for the original excavations/ sample collection. If this information is not available, please indicate this.

N/A

How was the potential cultural significance of the materials collected in your study to local communities considered in your research design? Were Indigenous peoples and/or local researchers and institutions involved with archaeological excavations / collection of specimens? If so, please provide a description of their involvement.

The potential cultural significance of blood samples collected from indigenous chickens was related to social and gender roles. The head of household, especially a man (or a woman in women-le households) is the main decision maker on the use of livestock and products. However, the consent from him or her is required. Yes, indigounous people and local researchers/technicians were involved in specimen collection. Indigenous people were birds' owners while researcher/technicians were responsible to collect samples.

If your manuscript includes photographs of human remains please indicate whether authors obtained permission from descendants or affiliated cultural communities to do so.

N/A
