# Supplementary material for: Genetic diversity and relationship of Bugesera and Rwamagana indigenous chicken populations with SASSO chickens using DArTseq SNPs
Source: PLoS One. 2025 Sep 12;20(9):e0331316. doi: 10.1371/journal.pone.0331316 (PMC12431124; doi:10.1371/journal.pone.0331316)
Supplement: S1 File — (PDF) [file pone.0331316.s005.pdf]

Kingdom of the Netherlands

TRAIDE

Poultry Sector Analysis Rwanda

based on the Poultry Learning Event 2019

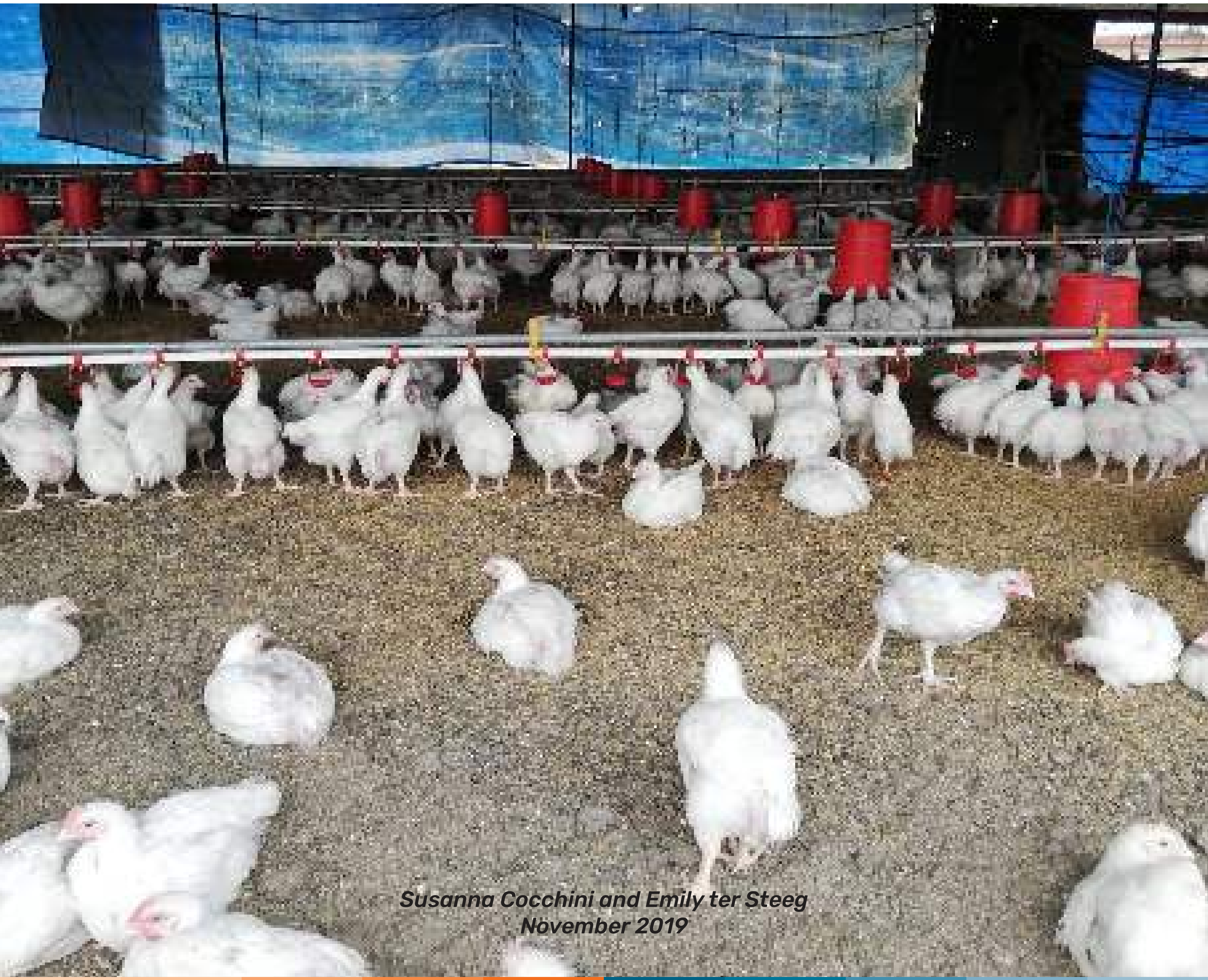

Susanna Cocchini and Emily ter Steeg

November 2019

TRAIDE

Contents

**FACT SHEET POULTRY SECTOR IN RWANDA**	**3**
**INTRODUCTION**	**4**
Rwandan context	5
Development of the poultry sector in Rwanda	5
**THE POULTRY INDUSTRY IN RWANDA**	**6**
Broiler poultry in Rwanda	7
Layer poultry in Rwanda	8
**INPUTS AND SERVICES**	**9**
Day old chicks	10
Chicken breeds	10
Animal feeds	10
Feeding equipment	11
Biosecurity, animal health & veterinary services	12
**Access to fnance**	**12**
**MARKETS AND TRADE**	**13**
**Domestc market**	**14**
Import and export	15
**OVERVIEW OF ACTORS OF THE POULTRY INDUSTRY**	**16**
Private sector	17
Public sector	17
**CHALLENGES AND OPPORTUNITIES**	**18**
Challenges	19
**Opportunites**	**20**
Conclusion	21
**REFERENCES**	**22**
**ANNEX 1**	**23**

RESILIENCIA RWANDA

TRAIDE

Fact sheet Poultry sector in Rwanda

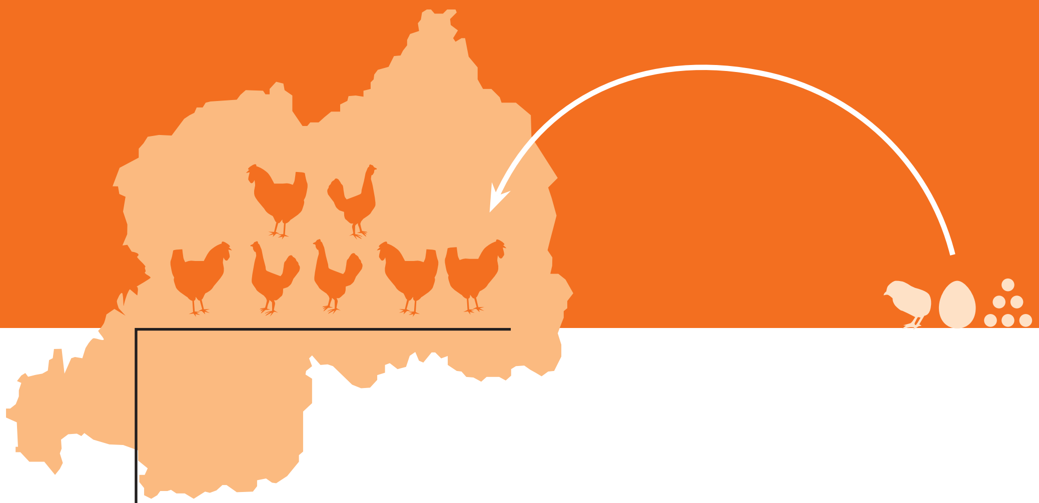

POULTRY SECTOR IMPORT:

- day-old chicks
- fertilized eggs
- ingredients for feeds

currently
5–7 million chickens
are reared in Rwanda[6]

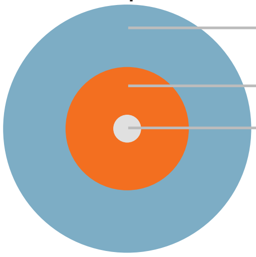

2021/22 objective
10,920,000 chickens

2017
5,426,000 chickens

1990s
1,226,000 chickens

ANNUAL EGG CONSUMPTION PER CAPITA IN RWANDA[6]

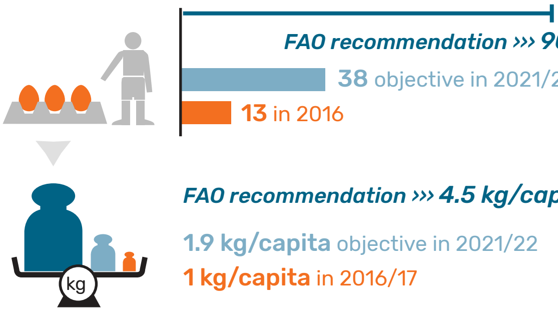

FAO recommendation ››› 90

38 objective in 2021/22

13 in 2016

FAO recommendation ››› 4.5 kg/capita

1.9 kg/capita objective in 2021/22

1 kg/capita in 2016/17

CHICKEN MEAT AND EGG PRODUCTION[6]

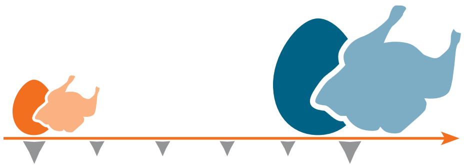

2017

243.7 million eggs produced

15,715 tons of chicken meat produced

2022

513.1 million eggs objective

35,170 tons of chicken meat objective

strong poultry sector

IDENTIFIED OPPORTUNITIES IN THE POULTRY SECTOR:

TRAINING

In Rwanda, technical and/or specialized knowledge on poultry is often missing. Moreover, poultry farmers often lack management skills needed to successfully run their **businesses. Considering the fnancial position of farmers,** this is an opportunity for development cooperation rather than businesses.

FEED

Poultry feed is scarce, expensive and often of low quality. Increased regional imports of raw materials (maize), increased local production of raw materials – or new **production facilities – would undoubtedly beneft the** sector. There would be strong market demand for innovative substitutes for maize.

BREEDING AND GENETICS

The demand for day old chicks is high and is not met by local hatcheries. Day old chicks are mostly imported **from Uganda and Europe. New hatcheries could ofer an** alternative to imports.

FINANCE

Improved access to and afordability of fnance would highly beneft poultry farmers, allowing them to invest in their businesses and obtain a higher and better-quality production.

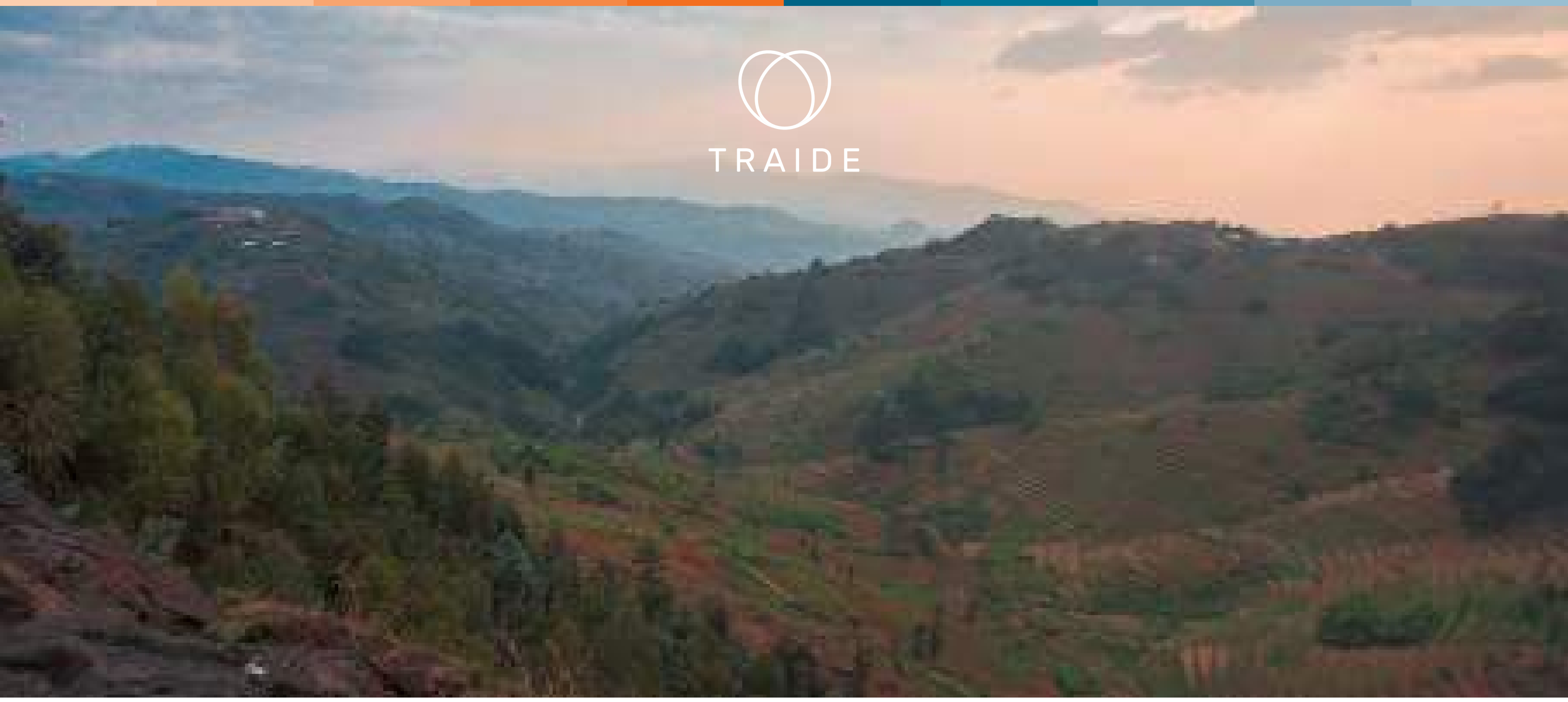

01.

Introduction

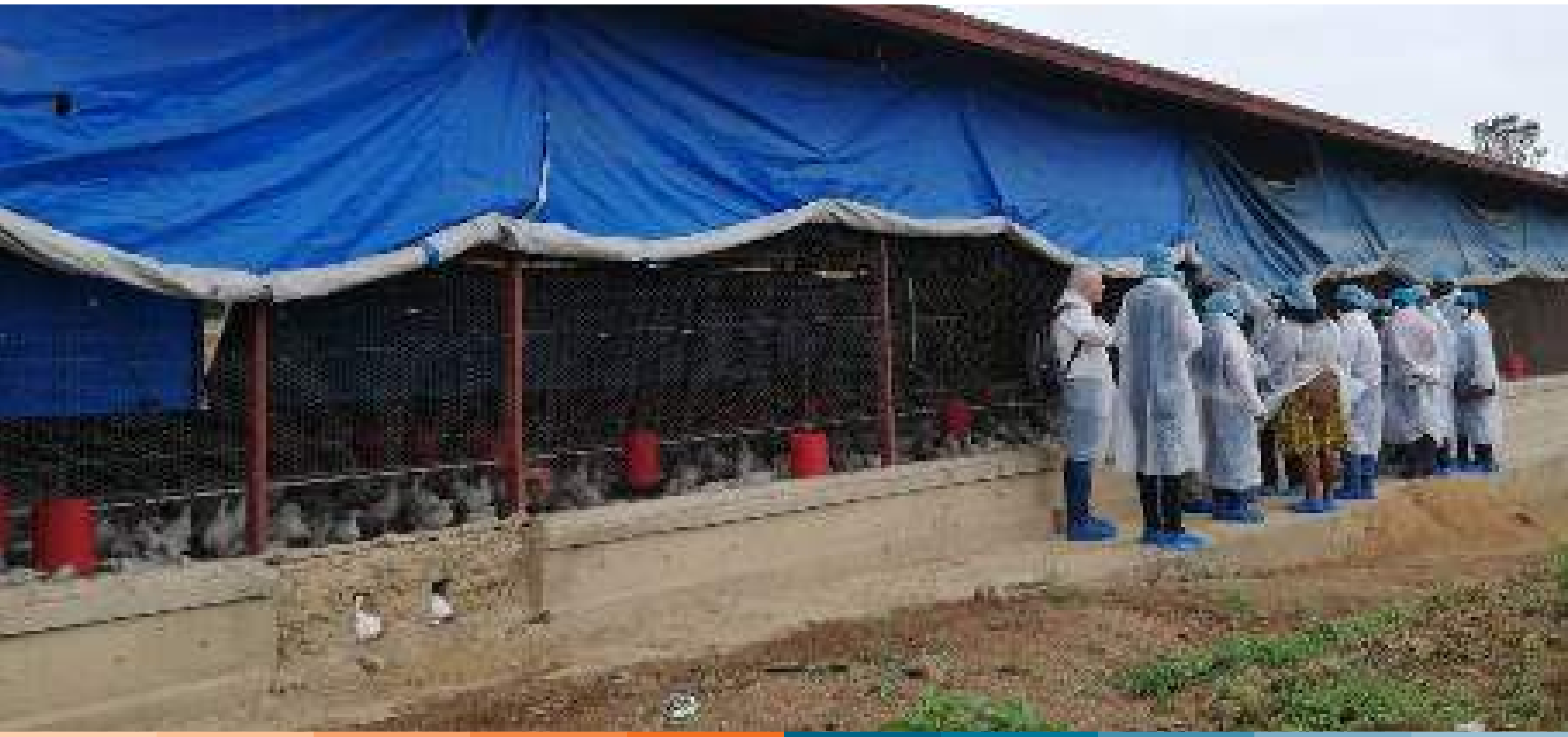

This document aims at providing insights in the current functoning of the poultry sector in Rwanda. Moreover, it ident fes opportunites for foreign and local investors, focusing in partcular on Dutch businesses operatng in the sector. The report was writen following the second editon of the Poultry Africa 2019 expo in Kigali, Rwanda.* Data was collected on the businesses of emerging farmers and a literature review was conducted. The results from data collecton and literature review efforts have been compiled in this report.

RWANDAN CONTEXT

Rwanda has an agrarian economy; which consttutes a third of the country's GDP, accounts for almost half of the exports and employs two **thirds of the populaton**[1]. The agricultural sector accounted for about **31% of the Rwandan Gross Domestc Product (GDP) in 2017**[2], which was 9,509 billion RWF in 2018[3] **(equal to 9.26 billion Euro). The Government** of Rwanda is planning on moving from low to middle income country and to be recognized as a knowledge-based economy. This structural trans-**formaton of the economy frstly requires increased productvity in the** agricultural sector, followed by increased employment in other sectors.

The Natonal Agricultural Policy published in 2018 outlines the development objectves of the Rwandan government. It aims at transforming the agricultural sector from a subsistence sector to a knowledge-based value creatng sector. Modernizaton of the agricultural sector will be realized via the implementaton of policies promotng technological upgrading, crop intensifcaton, high-value commodites, land registraton, organisaton in farmer cooperatves and decentralizaton. Private sector-led development and foreign investments are also major components of the government strategy.

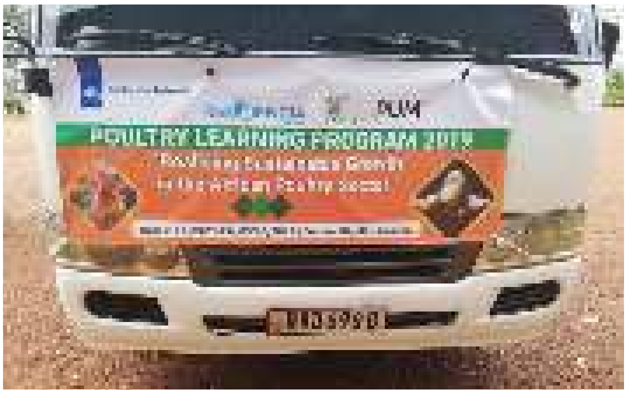

In October 2019, AgriProFocus Rwanda, Resiliencia Rwanda (in the context of the TRAIDE Program) and PUM organized a poultry learning event that was atended by 47 farmers from seven diferent African countries: Burundi, Cameroon, Ethiopia, Rwanda, Tanzania, Uganda and Zambia. Field visits were organized to four diferent farms (broiler and layer) and workshops on biosecurity, feeding tech-niques and economic management were organised. The program **was funded by the Netherlands Embassy in Kigali and RVO.**

More info on the TRAIDE Program can be found at:
htps://resiliencebv.com/projects/traide-rwanda/
More info on the Poultry Learning event can be found at:
htps://agriprofocus.com/poultry-masterclass-2019

DEVELOPMENT OF THE POULTRY SECTOR IN RWANDA

The poultry sector in Eastern Africa has been substantally growing in the 5–7 years, driven by rapid urbanizaton, growing middle class in the region and growing demand for animal protein[4]. Similarly, the Rwandan poultry **sector is growing rapidly along with the populaton and demand for animal** protein. In Rwanda, poultry farming is prevalently rural and family-based, **with 69% of all poultry farmers rearing one to two chickens**[5]**. Overtme, the sector has started to atract entrepreneurs that set up medium to large-scale farms, initatng the development of the commercial poultry system in Rwanda.**

The Government of Rwanda recognizes the important and strategic role the poultry sector can play. Its vision for the sector is described in the Livestock Master Plan, published in 2017 and covers market prospects for the next 5 years. **The objectve is to transform the country's poultry industry from dominantly** subsistence-based to knowledge-intensive and market-oriented. The aim is **to raise the number of hens from 5.2 million in 2016 to 7.1 million in 2022**[6]. **The government wants to enhance productvity in three coexistng sub-systems: Improved Traditonal Family Chicken (ITFC), Crossbreed Family Chicken (CFC) and Specialized Commercial Chicken (SP) producton. This transformaton would result in a more advanced poultry sector, beter income for chicken growers and improved food and nutriton security for the Rwandan people.**

Table 1. AIMS FOR THE POULTRY SECTOR, ADAPTED FROM LIVESTOCK MASTER PLAN (SOURCE: ILRI *et al.*, 2017: 76)

System(s)	Item (unit)	2016/17 (baseline)	2021/22 (target)	Change (%)
Family poultry (ITFC & CFC)	**Hens (million)**	5.2	7.07	**+35 %**
	**Eggs (thousands)**	83,370	**190,754**	**+129 %**
	**Chicken meat (MT)**	5,081	9,018	**+77 %**
Commercial poultry (SP)	**Layers (million)**	0.77	1.55	**+101 %**
	**Broilers (million)**	0.97	**2.42**	**+149 %**
	Total chicken **(million)**	**1.74**	3.97	**+128 %**

* See box above.

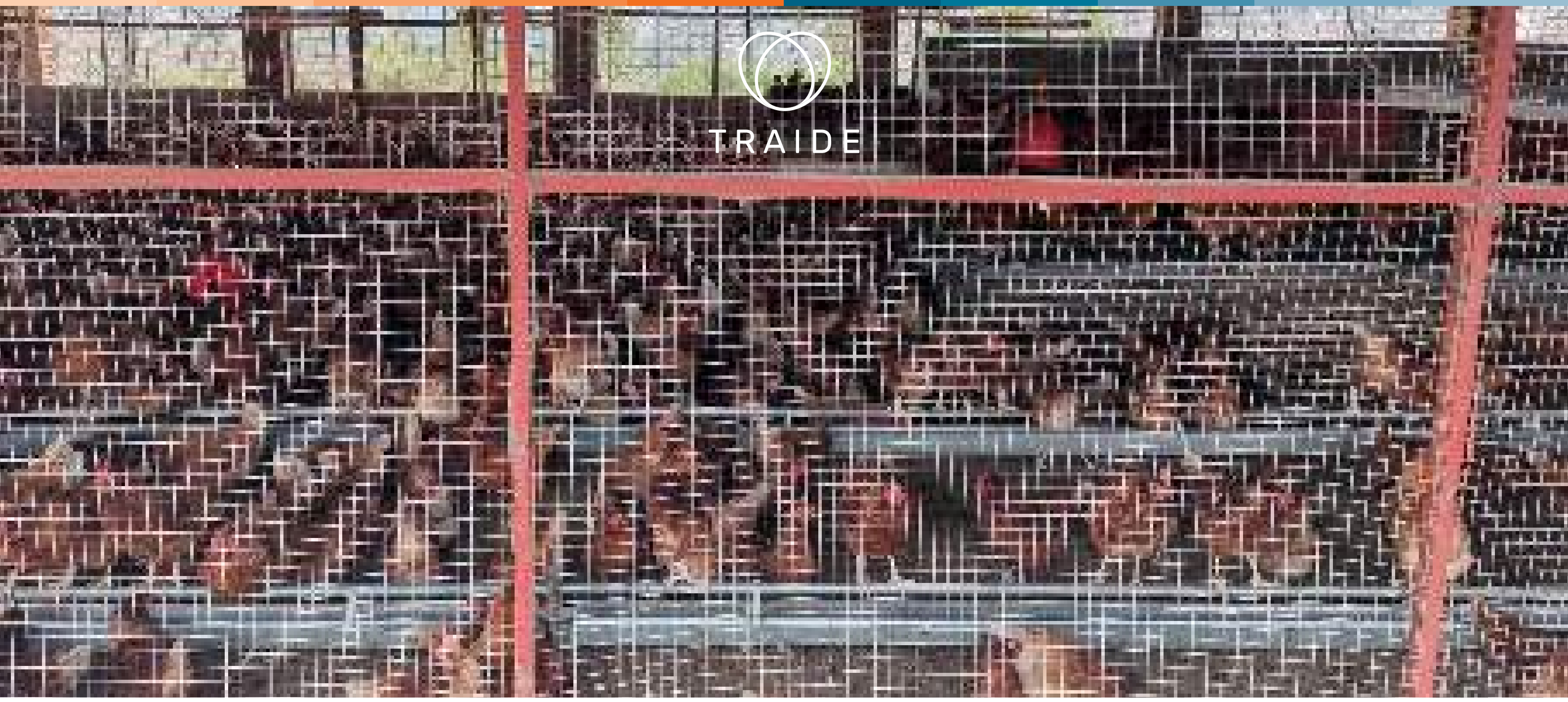

02.

The poultry industry in Rwanda

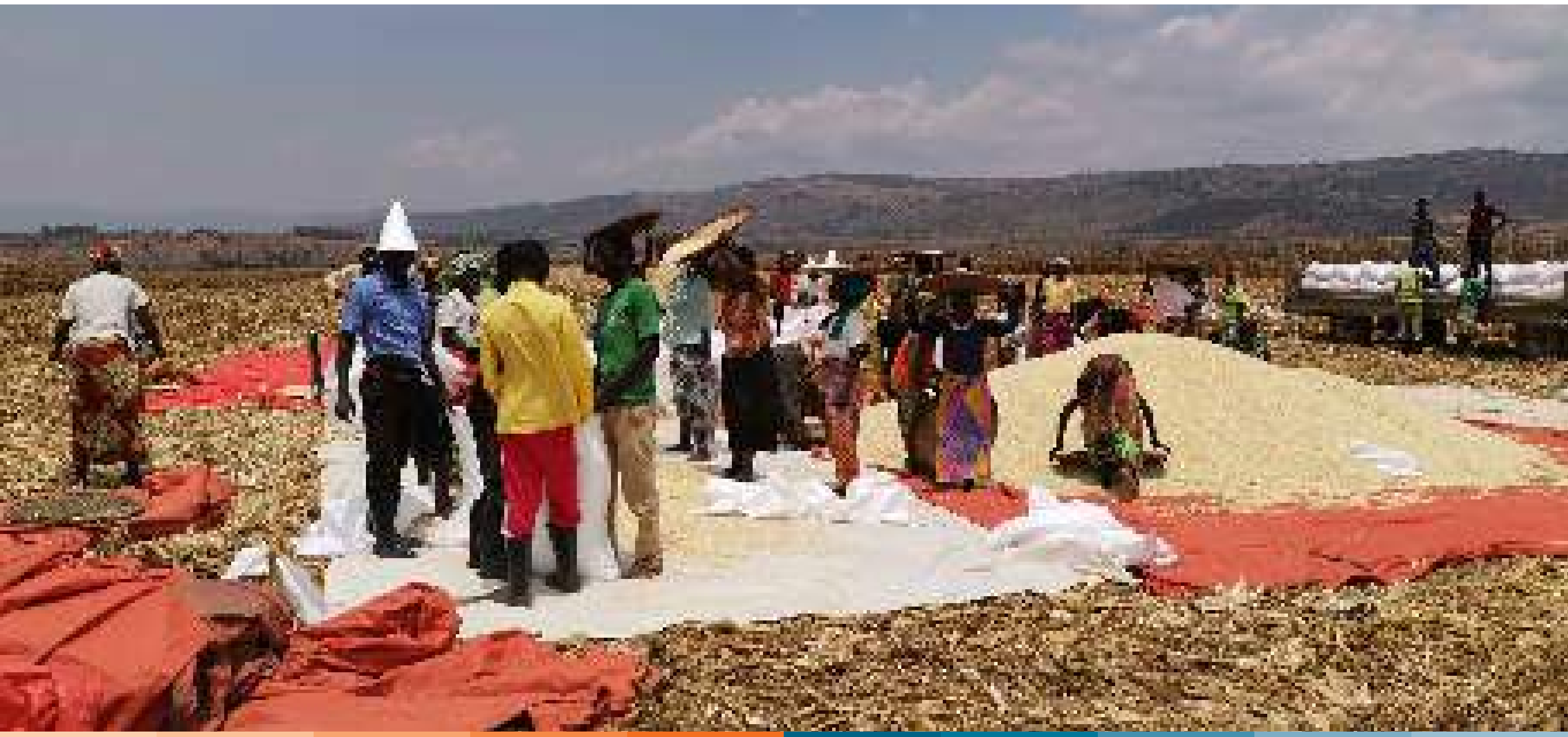

In Rwanda, there are two coexistng poultry producton systems: traditonal/rudimentary village poultry (which roughly corresponds to the aforementoned ITFC and CFC) versus commercial poultry. Traditonal village poultry is the dominant system. The number of chickens reared in Rwanda varies between 5 and 7 million, of which only one million is part of the commercial producton system[6]. In 2017, the total chicken meat producton in the country counted for 15,715 tons, while total egg producton counted for 243.7 million eggs[6] (ca 8,160 tons).

Traditional system

The traditonal system is relatvely well-distributed throughout rural, urban and peri-urban areas. It is characterized by minimal biosecurity measures **and by a direct connecton between producers and consumers. In the** village poultry system, farmers usually consume eggs and chicken meat **themselves, or sell/give it to family and neighbours, ofen in exchange** for other agricultural products they lack. Rudimentary village poultry is **largely practced by Rwandan families living in rural areas. In village poultry farming, fock size is usually between 2 and 20 birds per household, with** the majority being below 10 chickens per household[7,8]. **Traditonal poultry farmers initally buy the birds from neighbours or village markets. Afer that, replacement of fock almost exclusively happens with their own birds**[8]. **Cross breeding of local chicken with high performing improved/pure breed is very scarcely applied in traditonal poultry, despite evident positve efects in terms of both meat and egg producton**[8]. Scavenging and free-**range are by far the most difused chicken farming systems amongst low cost poultry producton. However, these traditonal systems result in low producton rates and high risks (that, if not properly addressed, can also impact commercial farms) linked to diseases, worms, pests and predators.**

Commercial system

The commercial poultry sector in Rwanda stll remains in the inital phase of development. It is characterized by larger fock sizes than village poultry and sales of chicken meat and eggs produced in the farm. Moreover, commercial **farm implement (basic) biosecurity measures and make use of (semi)automatc feeding systems. In commercial poultry producton, a distncton must be made between broiler poultry, aimed at chicken meat producton, and layer poultry, aimed at egg producton. In additon to selling meat and eggs, commercial** poultry farms generate some income from selling chicken manure to farmers **and the remains of birds processing (heads, innards and feet) to pig growers.**

BROILER POULTRY IN RWANDA

In Rwanda, the majority of poultry farms are small (meaning their capacity is less than 1000 chickens) and medium, with a capacity of 1000–5000 chickens. There are approximately twenty-six small farms, **eleven medium-size farms and fve large-scale broiler poultry farms*.**

In commercial broiler poultry, chickens are reared in open sided chicken **houses, with fve to ten chickens per square meters. Afer the frst two weeks, chickens are usually moved to a diferent chicken house. When birds are forty-two/forty-three days old, they are slaughtered and the meat is processed, packaged and stored untl sales. Some large-scale poultry farms are equipped with more facilites: hatcheries, feed mills, slaughterhouses and cold storages. It is not unusual for broiler farms with processing facilites** to buy live chickens from smaller farmers, process them and sell the meat. **Small-scale farmers ofen do not have the required fnancial resources or access to fnance to invest in slaughtering facilites. Moreover, they have limited market connectons. Therefore, they beneft from selling live chickens** to farms that can process them and easily place the meat on the market.

During the Poultry Learning Event two broiler farms were visited: PEAL, one of the most advanced farms in Rwanda, and Bishenyi Chicken Farm, a **smaller and more traditonal farm. PEAL has a capacity of 50,000 chickens with all necessary facilites in-house: hatchery, feed mill and slaughtering and storage plants. They either import chicks or fertlized eggs (Cobb breed) that they hatch for twenty-one days and then move to chicken houses. Afer two weeks, chickens are transferred to a diferent house to be slaughtered when they reach forty-fve days of age and a weight of 2.4/2.5 kg. The slaughtering plant can process up to four hundred birds per hour. Subsequently, the meat is stored in a 12T cold storage (also called a 'chiller') from zero to fve degrees Celsius and moved to a 16T cold container (freezer). In additon to chicken rearing, PEAL has also set up an outgrowing scheme. They provide small farmers with inputs (chicks, feed and medicaton) and buy the fully-grown chickens, process them and sell the meat.**

The second farm visit was to Bishenyi Chicken Farm, with a total capacity **of 24,000 chickens. At the moment of the visit, the farm only hosted 6,000 chicks following the producton cycle. Bishenyi imports chickens** every two weeks, mostly from the Netherlands. They manually feed chicks non-stop for two weeks. Then, they proceed by feeding twice a **day in the morning and afernoon. The farm owner also has a butchery and gives the farm manager indicatons of the required number and** weight of chickens in order to plan the slaughtering process accordingly.

*** Name, locaton and capacity of large-** scale farms are reported below in the document.

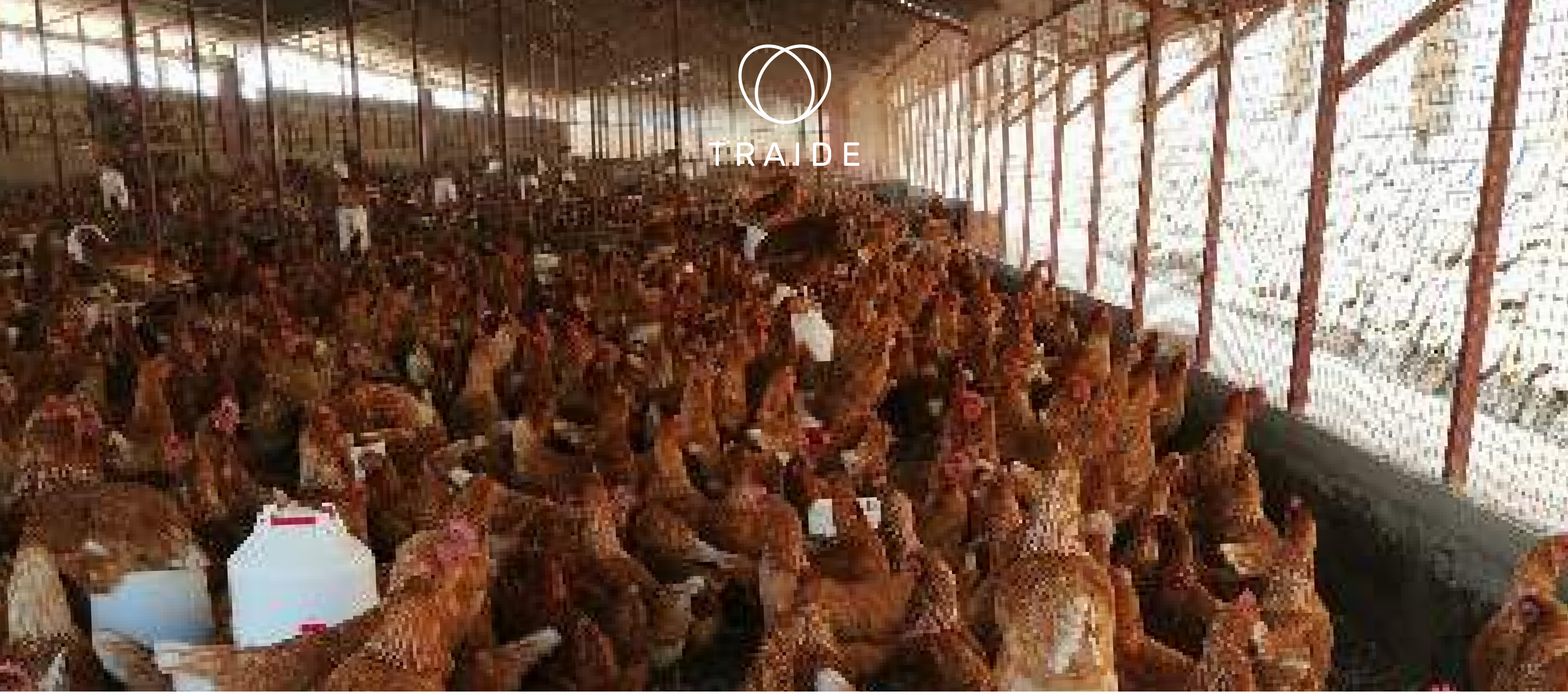

LAYER POULTRY IN RWANDA

Similar to broiler farms, small and medium producers count for the vast majority of layer poultry farms in Rwanda. Approximately 210 small farms, 108 medium-size farms and 27 large-scale farms are actve in the country*.

In commercial layer poultry, chicks are frst reared in a rearing house for twelve to fourteen weeks and then transferred to a layer house untl they reach around ninety weeks of age. It is common practce that, once their laying capacity is over, chickens are sold alive on local markets, to hotels and restaurants or to chicken processors.

During the Poultry Learning Event, partcipants visited two layers farms: Abusol and Isimbi. Abusol is the biggest and most advanced layer farm in Rwanda. They import day-old chicks (Isa Brown) from the Netherlands (ISA Export BV, under Hendrix Genetcs) and rear them in houses with a capacity of 8,000 chickens. At Abusol, a deep liter system is in place, meaning chicken dropping and bedding materials compost inside chicken houses. The manure is immediately removed from the property and sold to neighbouring farmers. Abusol has its own feed mill which they use to mix raw materials with nutrient formulas/supplements they buy. The raw materials used in the mix depends on availability and price. The peak producton is roughly 80% and reaches peaks of 95% at seventeen weeks. Most eggs are exported and sold to the Democratc Republic of Congo (DRC) at 75 RWF each (0,08 Euro).

Isimbi has a capacity of 15,000 chickens and rears Isa Brown breed chicks they buy from Agrotech. Half of the fock at Isimbi is reared in deep liter houses, while the other half is kept in three ter batery cage systems. The later system allows for a cleaner and easier collecton of eggs. Feed is bought from Rwandan producers, and is a mix of premix, maize, soya, sunfower cake, and calcium. On average, producton reaches 16,000 eggs per day. Eggs are then sold to local businesses: supermarkets, small shops and restaurants.

* Name, locaton and capacity of the fve main large-scale farms are reported below in the document.

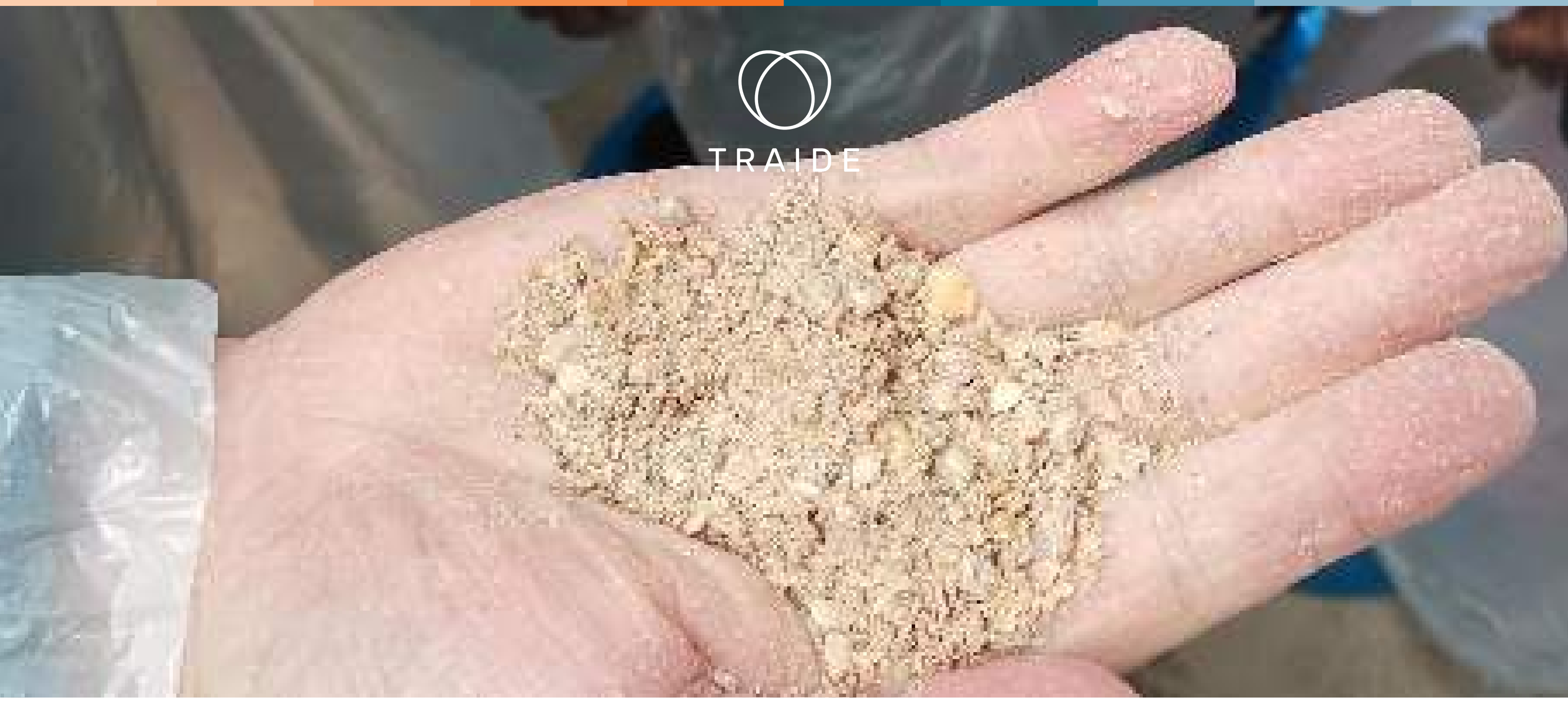

03.

Inputs and services

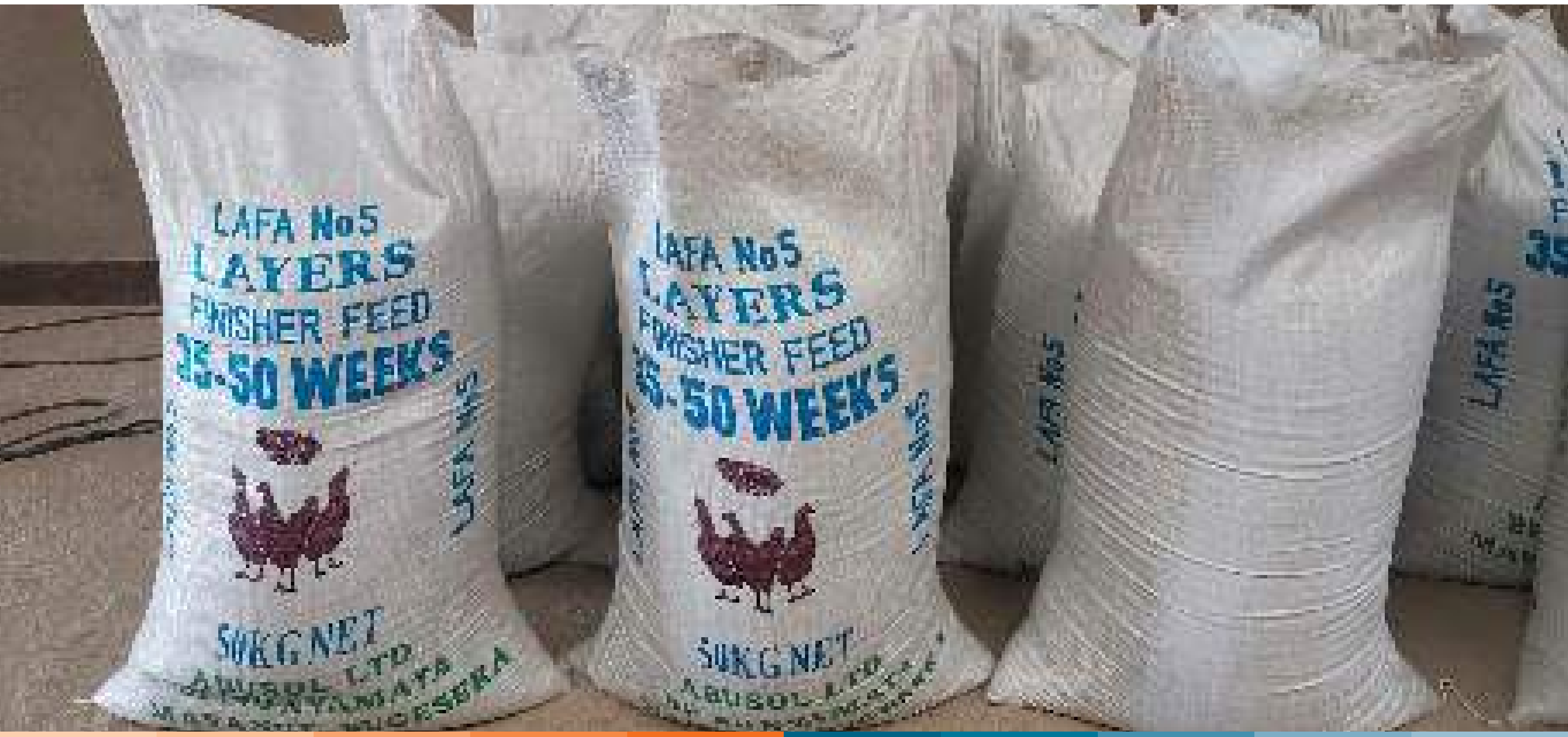

DAY OLD CHICKS

Three large hatcheries are operatonal in Rwanda: Easyhatch Ltd, Rwandachick Ltd and Uzima Chicken. The later was previously known as the Rubilizi Natonal Hatchery, but it was privatzed in 2017 following a three million USD investment from AgDevCo. This investment will enable Uzima Chicken to reach an annual producton capacity of eight to ten million day-old chicks. Uzima Chicken is the only Rwandan producer of Sasso breed dual purpose chicks, and also ofers Isa Brown breed. The parent lines of both breeds are imported from the Netherlands (Hendrix Genetcs). Easyhatch ofers chicks for both layers (Lohmann Brown) and broilers (Ross 308). The total producton of day-old chicks in Rwanda reached 413,760 units in 2015[9].

Despite the presence of local hatcheries, the demand of day-old chicks in Rwanda contnues to exceed supply. Commercial farms ofen import day-old chicks or fertlized eggs, if they have hatching facilites. Most Rwandan commercial poultry farmers buy day old chicks from Belgium, the Netherlands, or Uganda[10,9]. Uganda is a good source of inputs in terms of day-old chicks and chicken feed thanks to its scale of producton. However, the Government of Rwanda seeks to prevent overdependency on imports. This objectve can also explain the privatzaton of the natonal hatchery. In village poultry producton, farmers mainly get their chickens from local markets or neighbours.

Currently, the main importers of day-old chicks are Agrotech Ltd and Biyinzika that, in 2015, imported approximately 276,000 and 420,000 day-old chicks[9]. The two companies import chicks and resell them to Rwandan farmers. Agrotech exclusively imports from Europe, mostly from Belgium, where they buy 20,000 layers and 3,000 broilers chicks per month[9]. Biyinzika buys 19,000 layers and 16,000 broilers every month from Uganda[9]. Combined, these two companies count for around 80% of all the imported day-old chicks in the country. For broiler poultry, an imported day-old chick costs between 540 and 900 RWF (0.52 and 0.87 Euro), while imported layer day-old chicks are sold at 790–1025 RWF[9] (0.76 and 0.99 Euro).

CHICKEN BREEDS

In Rwanda, the most diffused breeds are the following:

- Cobb 500 and Ross 308
  Both breeds are suitable for broiler and are usually imported from Uganda. Sometimes they're imported from Europe.

- Kuroiler
  Hybrid dual purpose breed. It can produce up to 150 eggs per year and yield 2.5/3.5 kg of meat, outperforming indigenous breeds.

- Isa Brown and Lohmann Brown
  Both breeds come from Belgium and are suitable for layer poultry. Chicken of these breeds start laying eggs after 6 months and live around 1.5 years. In ideal conditions, brown breeds chickens lay more than 400 eggs during their lifetime. In Rwanda Isa Brown yields about 360 eggs at 90 weeks of age. For both breeds, the price of a day-old chick is around 1200 RWF (1.16 Euro).

- Sasso breed (France)
  The Sasso breed is suitable for both layer and broiler poultry. During their lifetime, they lay 200–300 eggs. The price of a Sasso day-old chick is 600 RWF (0.58 Euro). Farmers often start their business with Sasso breed chickens, as they are cheap and easy to manage.

- Inyarwanda (Rwanda)
  Local breed suitable for layer poultry; chickens start laying eggs between 8 and 11 months.

In rudimentary village chicken farming, indigenous breeds are dominant. Local breeds have lower productvity but higher resistance to local diseases and lower quality feeds in comparison to European breeds. Hence, they are more suitable for the rudimentary systems. Dwarf breeds are widely difused. These chickens are smaller and ofer poor producton and growth parameters. Nonetheless, dwarf breeds are characterized by high prolifcacy, adaptability to harsh environmental and poor feeding conditons, and resistance to diseases. Improved exotc chickens produce more eggs and meat than indigenous chicken breeds, but they are not always compatble with tropical climate, e.g. because of high temperature, diseases and shortage of feed.

ANIMAL FEEDS

In Rwanda, farmers have two optons to procure feed: they can either buy it or produce it. The required components for chicken feed are carbohydrates (e.g. maize, rice and wheat bran), protein (e.g. soy, coton seed cake and fsh meal) plus vitamins and minerals (concentrates). Farmers can either buy complete feed or a pre-mix, a concentrated formula that covers for the vitamins and minerals components. In the frst case, the feed is already mixed and homogenous. In the second case, the farmer needs to properly mix the concentrate and raw materials (sources of carbohydrates and protein); otherwise there is a risk of obtaining non-homogeneous feed. This will result in non-homogeneous chicken growth because chickens will neglect eatng the smaller parts with the micronutrients. This risk is also higher in case of (small) farmers who use shovels to mix the ingredients.

During diferent stages of life, chickens need diferent nutrients and thus need to be fed accordingly. In broiler poultry, three feeding phases can be identfed:

1. Starter feed, from 0 to 10 days of age. In this frst phase, it is crucial to have high quality tasty feed, that will establish a good appetite and maximize early growth. During the first 10 days, 22% of the intake should be crude protein.
2. Grower feed, from 11 to 25 days of age. Grower feed has less protein than starter feed and is more energy dense. The major sources of energy in poultry feeds are carbohydrates (such as corn and wheat) and fats (oils). The transiton from starter to grower feed involves a change of texture from crumbs/mini-pellets to pellets, which should be as uniform as possible.
3. Finisher feed, from 26 days of age untl slaughtering (if chickens are slaughtered later than the usual 42/43 days of age, a second fnisher formula may be needed). Finisher feed usually accounts for the major volume and cost of feeding a broiler chicken.

In layer poultry, four different feeding stages are identified:

1. Starter: for the frst weeks afer hatching; it is formulated to support organs' development and is dispensed in crumbles;
2. Grower: from the 5th untl the 10th week; supports the development of the birds' skeleton and is dispensed in mash or crumbles;
3. Developer: from the 11th untl the 16th week; it is dispensed in mash or bigger crumbles;
4. Pre-lay: afer the 16th week, dispensed in mash.

At the moment, farmers report feed prices in Rwanda ranging from 375 and 410 RWF/kg (0.36 and 0.40 Euro). The price of animal feed in Rwanda is very high and is subject to major fuctuatons. It was reported by Rwandan farmers that prices doubled in the course of one year. There are two main reasons why this is the case:

- Scarcity of raw materials and dependency on imports. The main reason for high and fuctuant prices of chicken feed in Rwanda is linked to the natonal (and regional) scarcity of raw materials for animal feed producton. Such scarcity led to increased prices for both maize and soy. The situaton is even worsened by export and import bans that countries in the region are implementing (e.g. Uganda, Tanzania and Zambia). Rwandans are therefore forced to import grains from abroad, sometmes from as far as Malawi, for very high prices.
- Lack of economies of scale. A multtude of feed producers operates in Rwanda, each at (relatvely) small scales. In 2015, the total amount of feed produced in the country was 12,800 MT[9]. In the same year, Tanzania produced 58,400 MT of poultry feed, Uganda produced 284,520 MT and Kenya produced 654,260 MT[4].
- Limited access to finance also subjects farmers to fluctuations in feed prices. Farmers reported they would like to invest in greater amounts of feed whenever prices drop. However, they do not have the financial resources to develop storage facilities or to purchase great amounts of feed at once.

In Rwanda, the main feed producers are PAFI, Gorilla Feed, PEAL and Zamura Feeds, that together account for sixty percent of the total producton sold in the country[10,9]. The remaining forty percent is covered by ten other smaller non-specialized units[9]. Feed producers do not always follow the recommended formulas[9], hence reducing the quality of the product. This may be due to the unavailability of certain components or to the lack of specifc knowledge on poultry of the animal feed producer. The combinaton of high and fuctuant prices and low quality incentvizes farmers to prepare the feed themselves, with an average cost of 290 RWF/kg[9] (0.28 Euro). Despite the aforementoned issues, the import of ready mixed poultry feed is low, while it is more common to import raw ingredients and mix them according to the demand[9].

FEEDING EQUIPMENT

In order to feed and water their chickens, advanced commercial farms in Rwanda usually install feeders to be manually reflled and 'line' drinkers, as shown in the picture below.

Smaller poultry farms utlize non-fxed feeders that employees place on the ground in the chicken houses during the appropriate feeding tme. This feeder positoning triggers serious health risks, as it implies high chances of faecal contaminaton in feed and consequent spread of diseases. According to the age (and size) of chickens, feeders must hover above the ground in order to prevent chickens from climbing on top. In village poultry farming, chickens mostly practce scavenging and are ofen fed on open feeder and drinkers, again resultng in serious risks of disease.

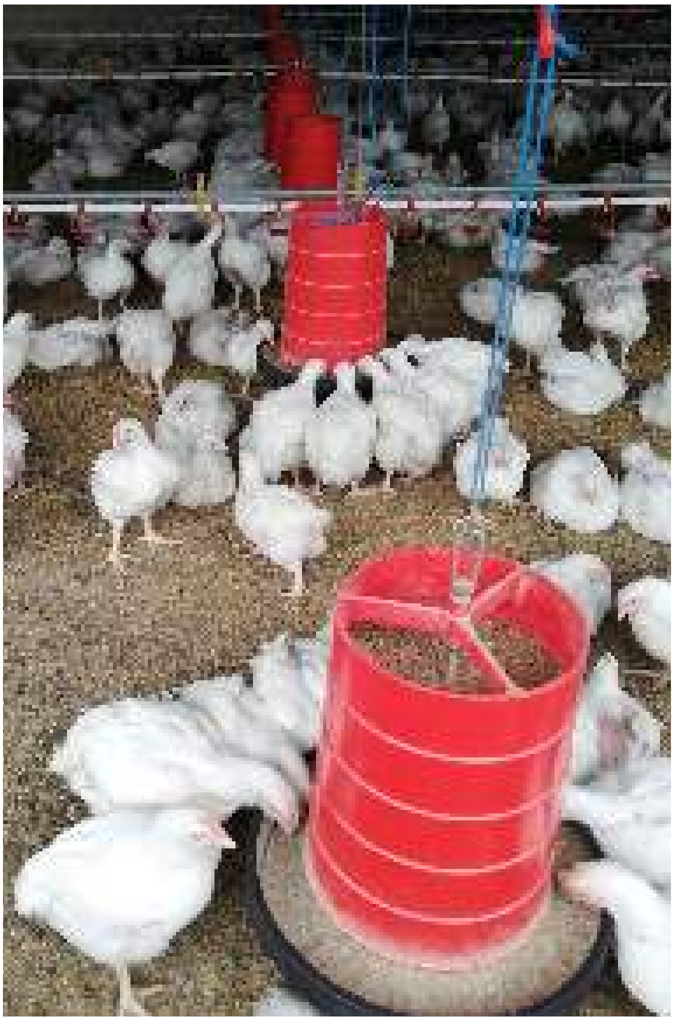

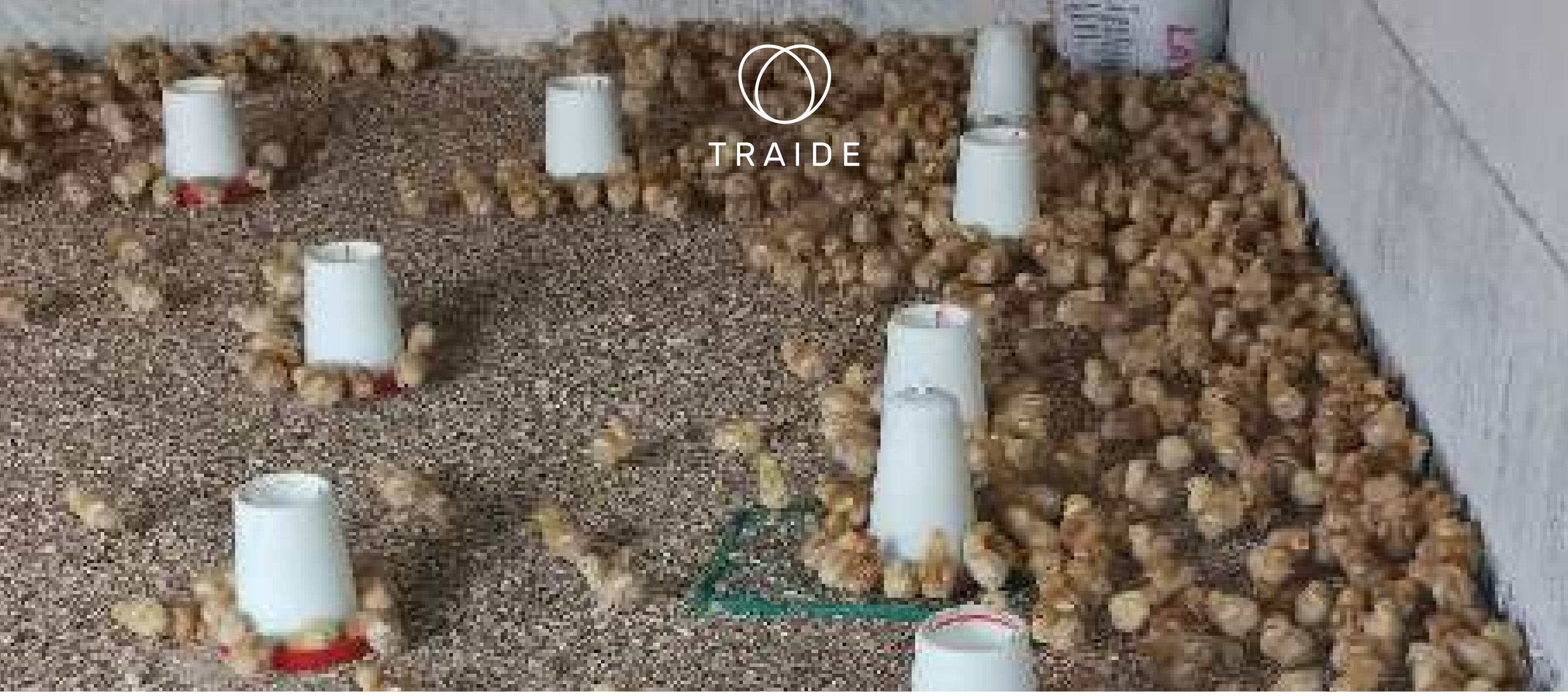

BIOSECURITY, ANIMAL HEALTH & VETERINARY SERVICES

The implementaton of biosecurity measures contnues to be a challenge for many Rwandan poultry farmers. Efectve implementaton is crucial for the preventon of diseases. Large-scale commercial farms usually have beter biosecurity protocols in comparison to small poultry farms. In traditonal village poultry producton, biosecurity measures are almost non-existent, with, for instance, records of chickens living in family houses[11]. These free-ranging village chickens pose a risk to poultry businesses nearby as they are contnuously exposed and at risk of diseases, which they can spread.

In commercial poultry in Rwanda, farmers are generally aware regarding biosecurity measures and health risks. For instance, the large majority (80%) of the partcipants to the Poultry Learning Event declared they regularly use antbiotcs and/or medicines in their farm. A larger percentage (83%) has equipped the farm with a hand washing staton with soap and trained (77%) personnel and occasional workers to wash their hands frequently. However, such measures are not sufcient. When entering the diferent areas of a poultry farm, it is necessary that both workers and visitors follow precise measures, amongst which changing clothes (or wearing single use security gear), stepping in a disinfectant footbath before entering chicken houses and washing cars' wheels before allowing them in the premises. Moreover, chicken houses must be cleaned and disinfected regularly, especially (but not only) when a new batch of day-old chicks is brought in.

Chickens are subject to several diseases of diferent nature, both infectous and parasitc. The main diseases are Newcastle, Gumboro (infectous bursal disease) and Coccidiosis (parasite). Other diseases commonly afectng chickens are Marek, Salmonellosis (bacteria), infectous bronchits, fowl pox, epidemic tremor and avian infuenza. In this last case, the Government is forced to shut down and isolate the farm. Every farm, independently of its size, should follow a strict vaccinaton plan, for instance the one suggested by RAB, in order to prevent their chickens to get sick on the most difused diseases.

The access of poultry farmers to medicine and health services is stll limited. Antbiotcs and medicines are usually bought from local agro-dealers, which are usually part of the Agrotech network. Agrotech is also the main supplier of vaccines. However, shops tend to run out of stock and vaccines are mostly sold in Kigali[9]. Depending on the diferent vaccinatons, they can be dispensed through drinking water, aerosol, eye drop, injecton, wing web or oral. With regards to veterinary services, diferences arise between commercial and village poultry farmers. The majority of the former consult veterinarians regularly or in case of a disease outbreak. By contrast, village poultry farmers ofen leave their sick birds for self-cure or use traditonal indigenous treatments[7,11].

ACCESS TO FINANCE

Poultry farmers interviewed during the Poultry Learning Event reported that one of their main difcultes is to access fnance to improve their businesses. Loans are not easily obtained by small farmers. When this happens, farmers face very high interest rates that can even reach 25%. Limited availability and accessibility to fnance hampers farmers from investng in more advanced and/or larger scale equipment. This is ofen because of lack of collaterals and guarantees small-scale farmers can ofer. Farmers are trying to team up to invest in machinery together like feed mills. Hence, it keeps them from growing and improving their businesses contributng to the development of the poultry sector.

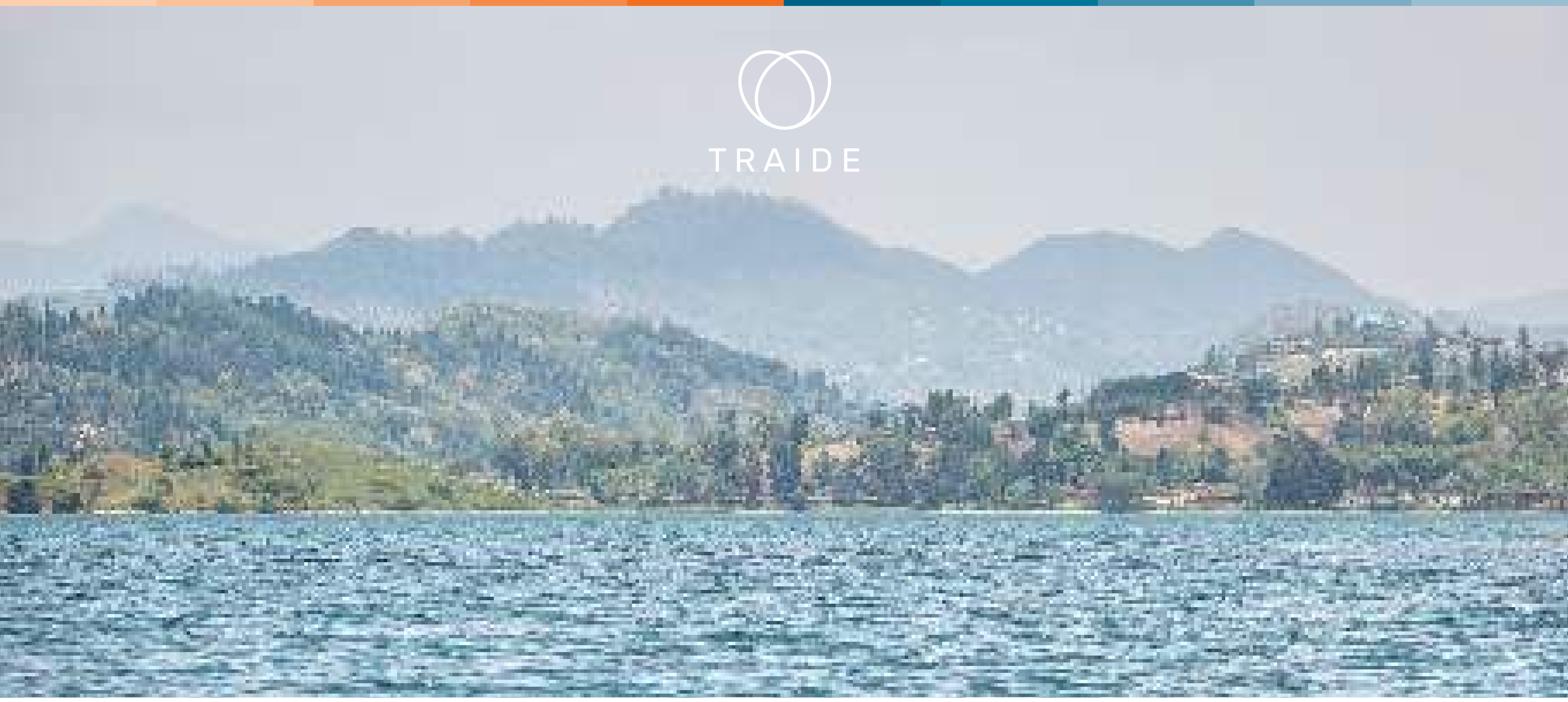

04.

Markets and trade

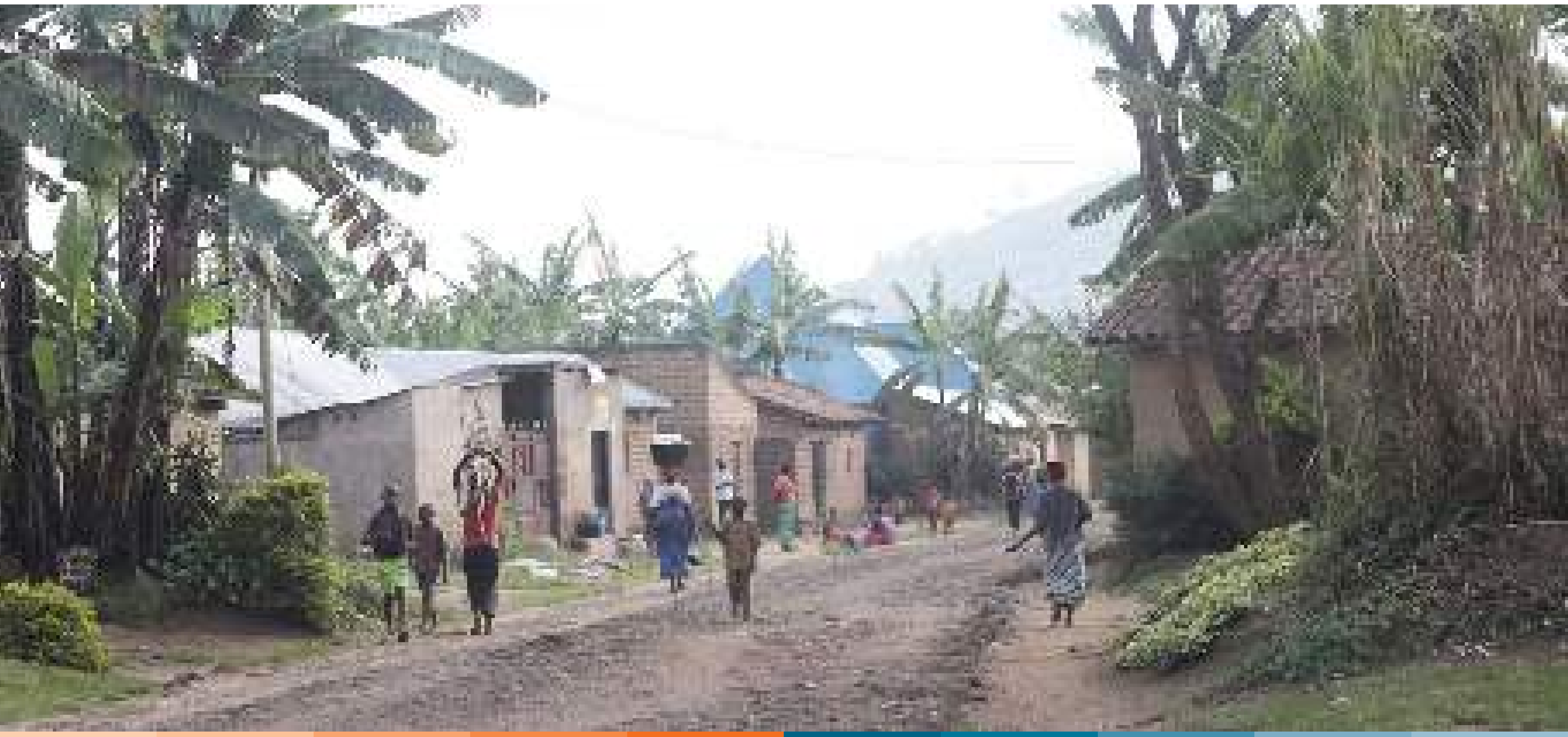

DOMESTIC MARKET

Africa has the lowest meat consumpton worldwide[12], that ofen implies a protein shortage in one's diet. The average annual poultry meat **consumpton per capita in Africa is 6.73 kg, followed by Asia (9.79 kg), Europe (23.35 kg), Americas (39.13 kg) and Oceania (42.67 kg). The world's average is 14.99 kg per person per year[12].** Furthermore, Rwanda is one of the African countries with the lowest consumption of poultry meat per capita, with only 0.2 kg of chicken meat consumed per capita per year in 2011[4] **(no more recent data is available).**

Alongside meat and plant-based protein, eggs are a great source of **nutrients. During the Poultry Africa Expo, the Dutch Embassy organized a promotion event for the egg together with VNU, Abusol, GAIN and the** Rwandan Government. The average Dutch person eats 281 eggs a year. In **Rwanda, egg consumption counted for 0.63 kg/person/year in 2014[1]**, against **an African average of 2.5 kg/person/year[4].** These numbers illustrate a huge difference in the development of the poultry sector. Protein deficiency is therefore not only a problem in Rwanda but for many African countries.

OneEgg Rwanda provides kids with one egg a day at their preschool activites. In 2018, they reached 4,400 kids across 36 preschools[16]. In the same year, the World Food Programme handed over ownership and manage**ment of their School Feeding Programme to GoR (which however did not** include the distribution of eggs[17]**). Moreover, a National Early Childhood** Development Programme is in place with the aim, amongst others, of improving nutrition in children[18]. The further growth and extension of such programmes can play a crucial role in fostering demand for eggs.

The growth of the local poultry sector ofers a relatvely sustainable soluton to the difused protein defciency in Rwanda. It is necessary to look at feed conversion rates: a low conversion rate means greater e f ciency. The producton of one kilo of chicken meat or eggs requires around 1.2–2 kilos of chicken feed[13,14]**. The production of a kilo of beef requires 6–10 kilos** of animal feed[13,15]. In terms of nutrition, poultry is a great solution, as it is affordable, it is a source of proteins, contains many essential vitamins and minerals and has less saturated fats than other types of meat. Therefore, the poultry sector has great growth potential in Rwanda, further enhanced by the low investment required and the short life cycle of broiler poultry. This indeed allows farmers to have a stable and recurrent source of income every few weeks. Finally, chicken meat is acceptable by all sorts of consumers, irrespective for instance of religious constraints.

Commercial poultry farmers mostly sell to local markets (or on the Kigali market), hotels and restaurants. Nowadays, chicken meat is sold at around 1300 RWF/kg (1.3 euro/kg), while eggs are sold at 70–75 RWF (0.07–0.08 Euro) each.

Figure 1. AVERAGE ANNUAL POULTRY MEAT CONSUMPTION PER CAPITA. (SOURCE: FAOSTAT**, 2019)**

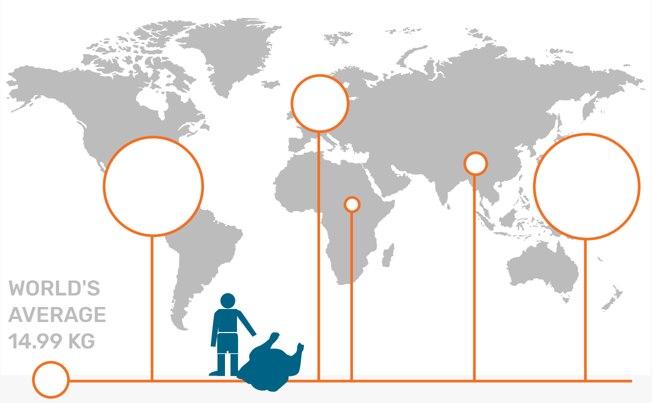

Oceania 42.67 kg/person/year
Americas 39.13 kg/person/year
Europe 23.35 kg/person/year
Asia 9.79 kg/person/year
Africa 6.73 kg/person/year
Rwanda 0.2 kg/person/year

IMPORT AND EXPORT

Meat and egg consumption in Rwanda are on the rise. National production volumes do not always meet the demand. In 2015, Rwanda imported **2,960 tonnes of chicken meat, 16% of the country's demand**[9]. In the same year, **exports reached 1,480 tonnes, counting for the 8% of the national demand**[9]. At this point, there are no formal regular export flows of poultry products from Rwanda to its neighbouring countries. Rwanda does not have the **production capacity to compete with its neighbouring countries like Uganda, Tanzania and Kenya, where the poultry industry is more advanced. Informally, poultry farms in the Western province of the country do often sell meat and** eggs across the border with DRC. In DRC, prices can be much higher with, **for instance, a tray of 30 eggs that is sold for 2.1 USD (1.9 Euro) in Rwanda, being sold at 4–5 USD (3.62–4.53 Euro). The same dynamics apply to the sale** of live chickens. Some Rwandan farmers also sell their products in Burundi.

The following tables illustrate the position of Rwanda in the region, in terms of live chickens and egg production.

Figure 2. DATA 1990–2017 LIVE ANIMALS: CHICKEN (SOURCE: FAOSTAT, 2017)

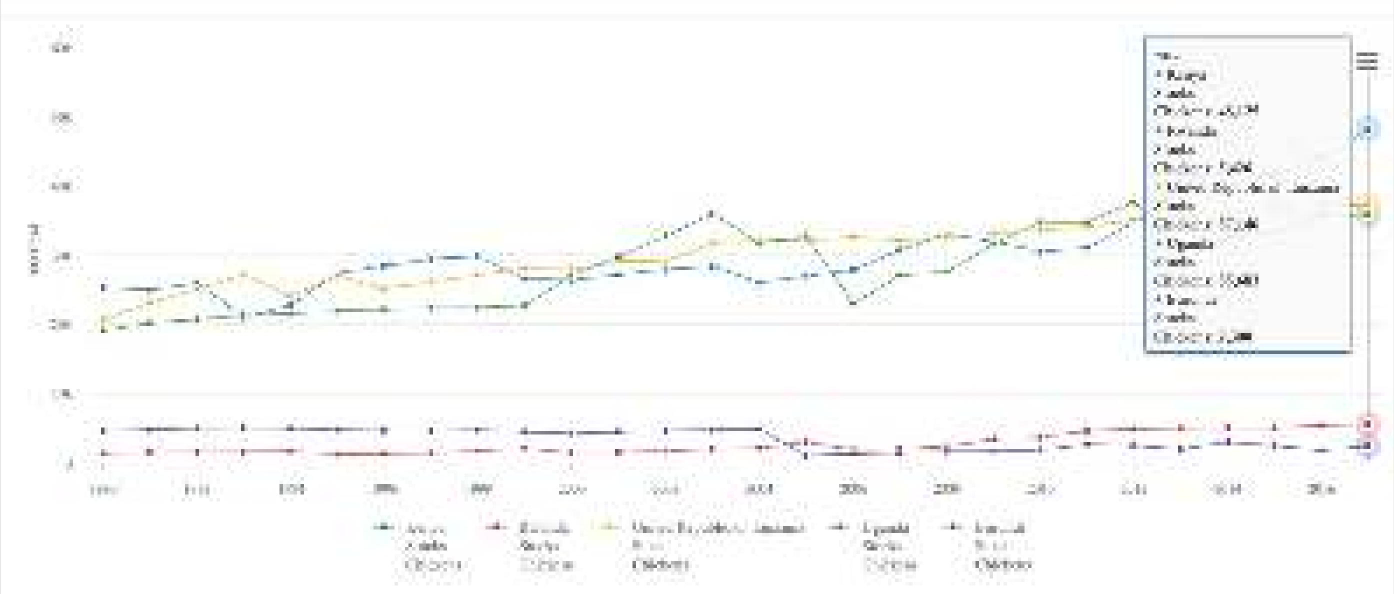

Figure 3. DATA 1990–2017 PRODUCTION QUANTITY: EGGS (SOURCE: FAOSTAT, 2017)

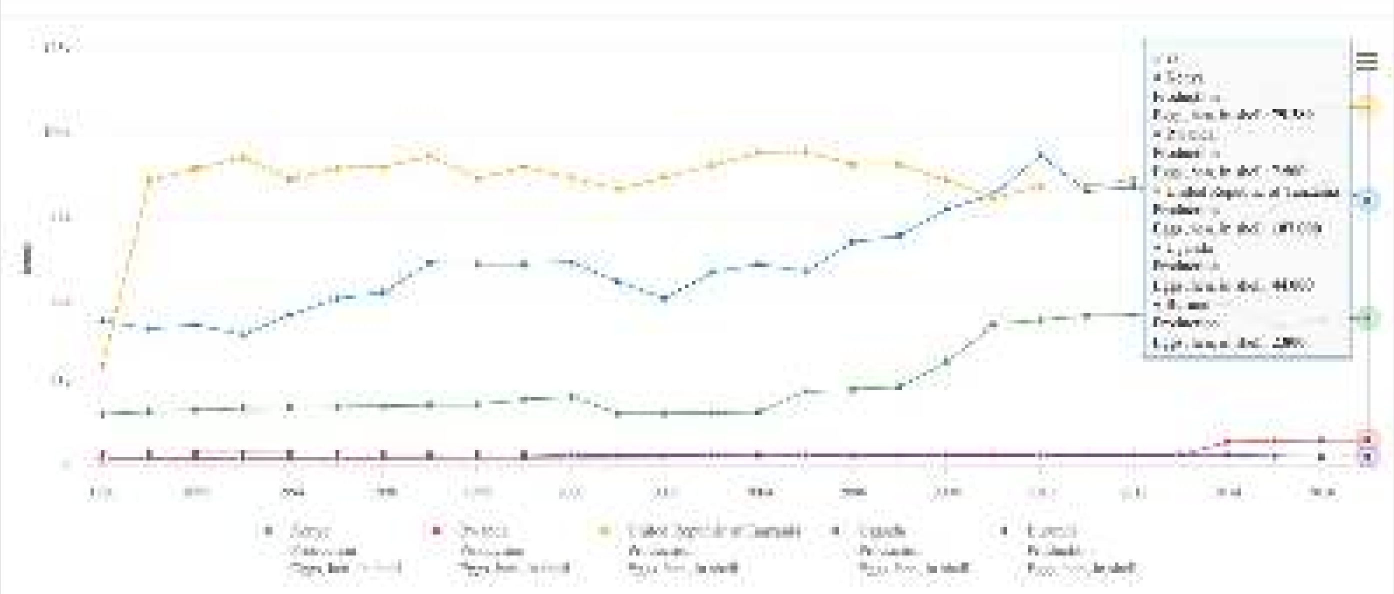

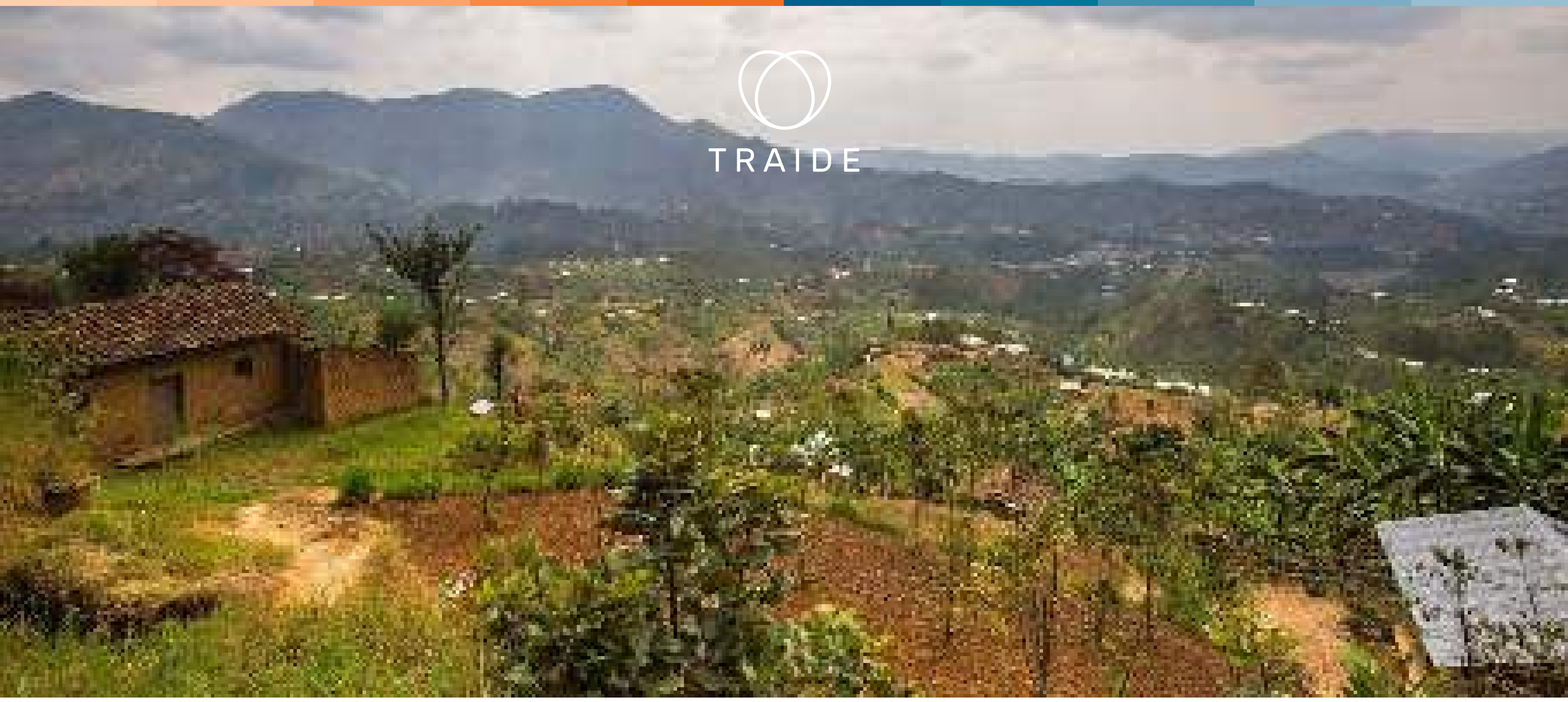

05.

Overview of actors of the poultry industry

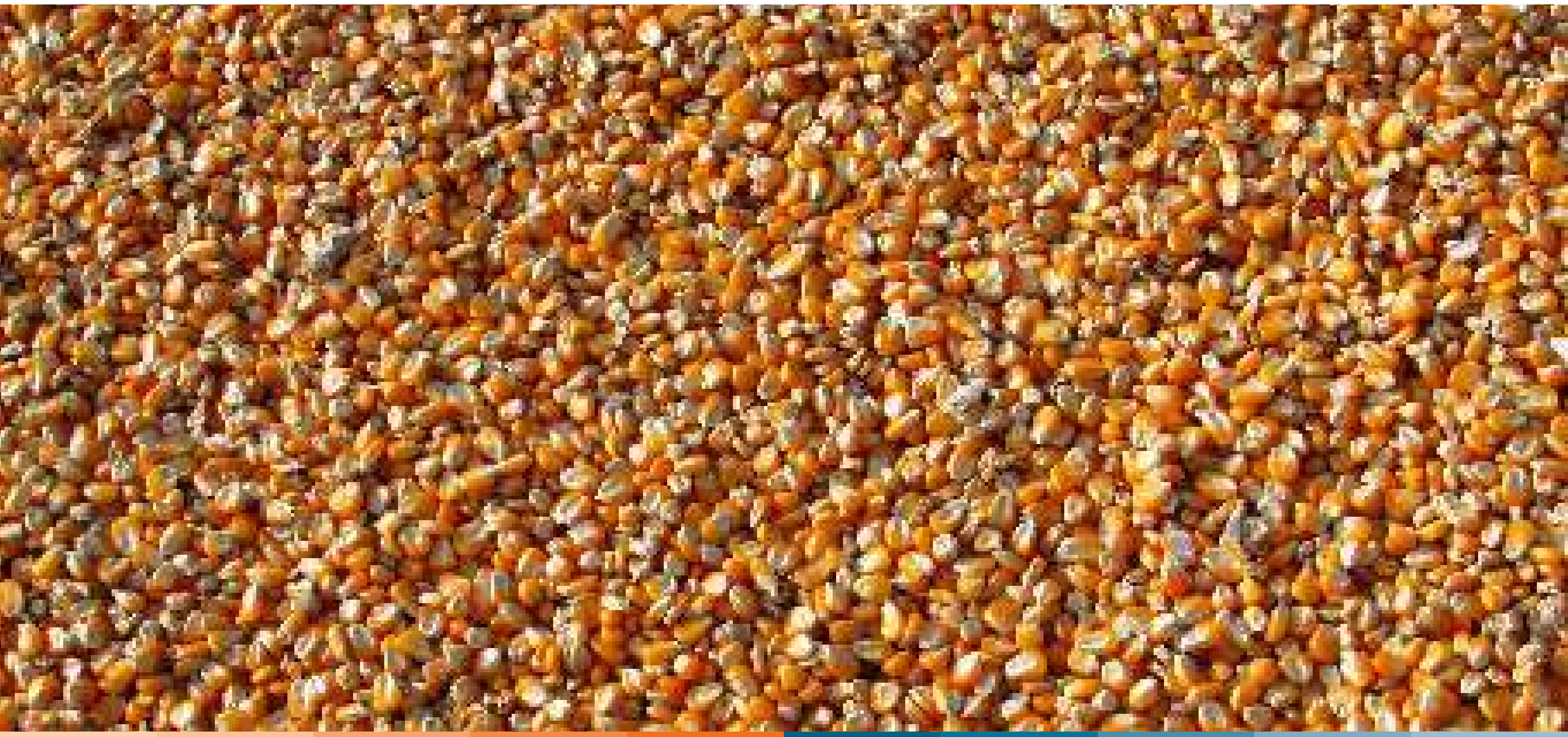

PRIVATE SECTOR

Table 2. provides a clear overview of the main private actors involved in different activities of the poultry value chain in Rwanda[9].

PUBLIC SECTOR

- **Minister of Agriculture: as explained in the Livestock Master Plan of** 2017, Rwanda aims at transforming the traditional chicken farming from subsistence to knowledge-based, with consequent increase in households' income and food and nutrition security.
- **Rwanda Agriculture Board (RAB): provides extension services.**
- **Rwanda Poultry Industry Association (RPIA): the association was born** to bring together all the stakeholders in the poultry industry, with the objectives of defending professional interests of the members, promoting members' activities and serving as a permanent forum for dialogue between the stakeholders and the public and private sector.

Table 2.

Category	Actor/Organizaton	Locaton	Capacity
Day-old chick producers	**Easyhatch Ltd**	Musanze	**20,000 chicks/week**
	Rwandachick Ltd	**Kigali**	
	**Uzima Chicken**	**Kigali**	**8 mil chick/year (planned capacity, not yet reached)**
Day-old chick importers	Agrotech Ltd	**Imports from Europe,** mostly Belgium	**Import 2015:**<br>**240,000 layers**<br>**36,000 broilers**
	Biyinzika	**Imports from Uganda**	**Import 2015:**<br>228,000 layers<br>192,000 broilers
Feed producers	**Zamura Feeds**	Musanze	**Producton 2015:**<br>300 MT layers feed<br>100 MT broilers feed
	PAFI	Rwamagana	**Producton 2015:**<br>95 MT layers feed<br>38 MT broilers feed
	Gorilla Feed	**Kigali**	**Producton 2015:**<br>53 MT layers feed<br>**41 MT broilers feed**
	**PEAL**	Bugesera	**Producton 2015:**<br>75 MT broiler feeds
Broiler chicken farmers and processors	**PEAL**	Bugesera	50,000
	Mugisha Farm	Bugesera	**24,000**
	Indian Farm	**Kabuye/ Nyarugenge**	20,000
	**dr. Abel Ukundimana**	**Kicukiro**	**16,000**
	**Kime Ltd**	**Kamonyi**	5,000
Layer chicken farmers	Abusol Ltd	Bugesera	51,000
	**Kigali Golden Farm**	**Kicukiro**	27,500
	dr. Gakuba	Gakenke	20,000
	Butera Antoine	**Kicukiro**	19,500
	Isimbi Farm	Rwamagana	15,000
Veterinary products suppliers	**Agrotech is by far the biggest provider of veterinary products. Agrotech set up a multtude of local branches that cover all the provinces of Rwanda, allowing farmers to avoid long trips to bigger ctes in order to get their supplies. However, vaccines are reported to be only available in Kigali (FAO, 2016).**		

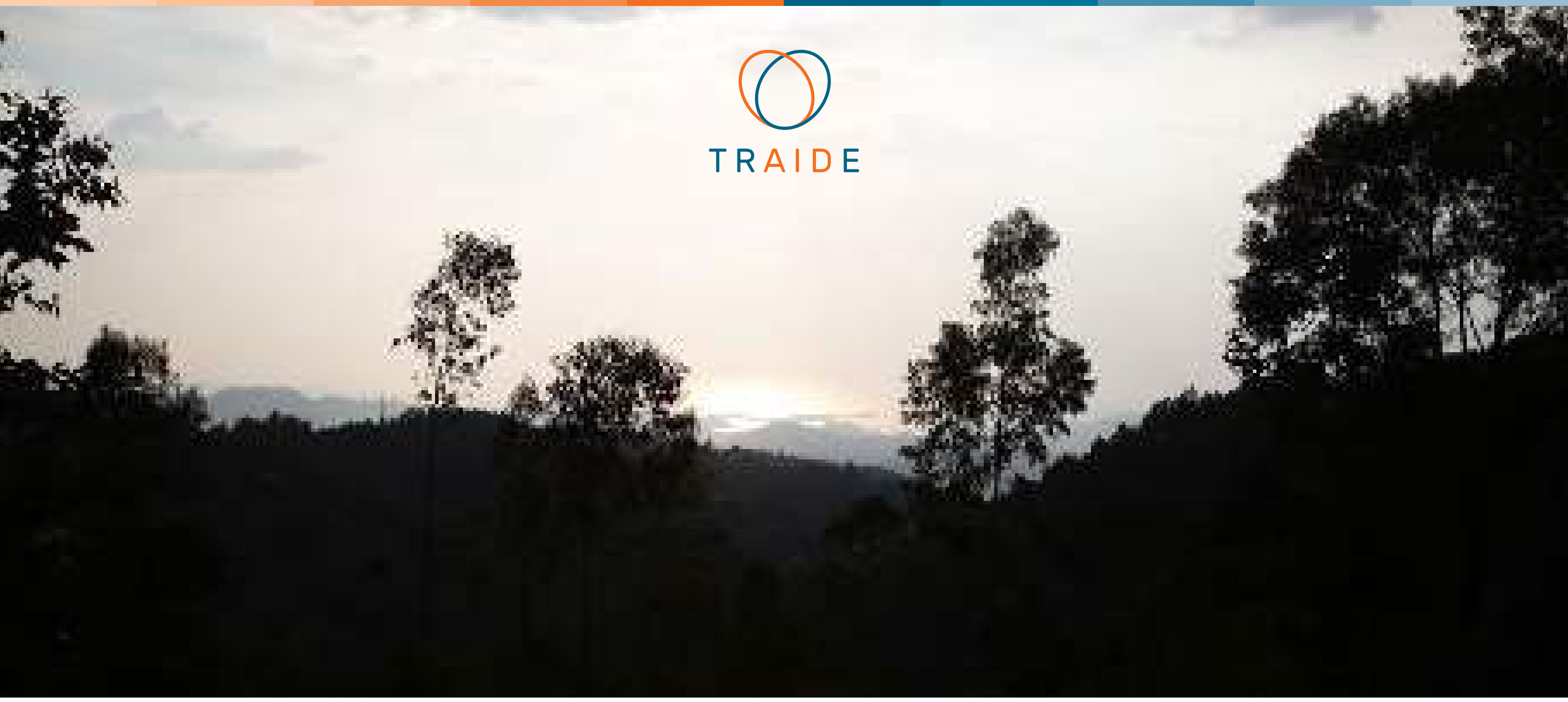

06.

Challenges and opportunities

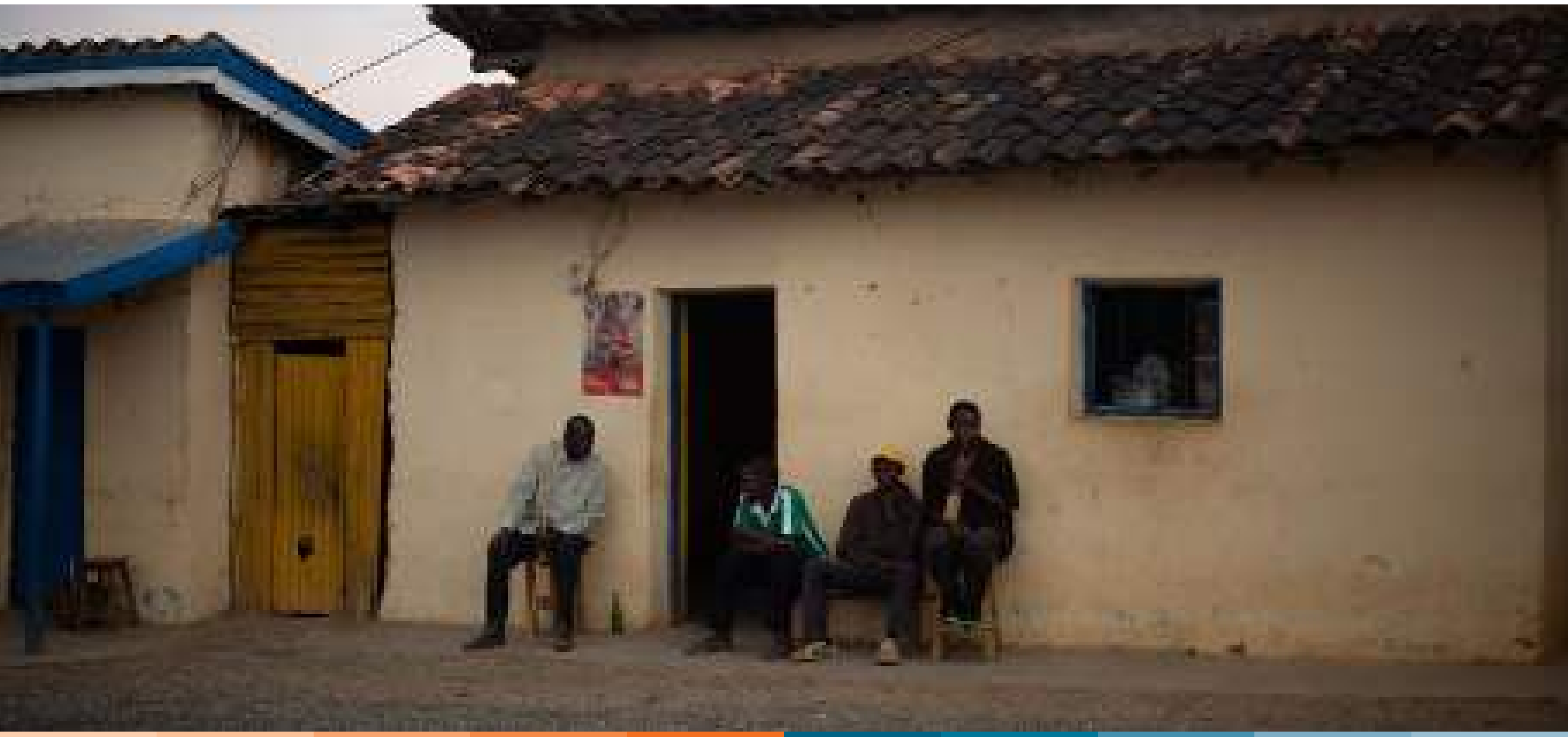

CHALLENGES

The poultry sector in Rwanda is still developing and obstacles hampering growth remain. Poultry farmers face several challenges, the most common are reported in the following table, together with potential solutions. The second column intends to indicate the prevalence of the issue according to the responses of farmers to the questionnaire dispensed **during the Poultry Learning Event. The question stated:** *'What are the main challenges (maximum 3 answers) you experience in poultry farming?'*.

When it comes to village poultry, more challenges need to be added, namely: lack of training on modern poultry producton practces and lack of atenton from agricultural policy makers, researchers, development and extension workers.

Challenge	Recogniton of issue* in PLE questonnaire	Interventon needed
**1. FEED**		
Low availability of maize and soy, due to lower regional **yields (most likely climate change-related) and competton with human consumpton (mostly concerning maize)**	Low	• **Make land available for additonal producton of cereals needed in** poultry feed<br>• **Introduce innovatve substtutes for maize as raw material for animal** feed like rice and cassava
High and variable price	**Very high**	• Resolving the issue of low availability would in turn reduce the price **(and its variability) of chicken feed**
Low quality	Low	• **Improve policy, regulatons and controls on the mater**<br>• Raise awareness on the importance of high-quality feed for healthy and **productve chickens**
**2. DAY-OLD CHICKS AND GENETICS**		
Dependency on DOC imports	Low	• Support the establishment of new hatcheries in Rwanda that, at high **producton volumes, can compete with import from Uganda or Europe**
**Low yielding potental of local breeds****	Not applicable	• **Shif towards high-yielding dual-purpose breeds (e.g. Sasso breed)**
**3. ANIMAL HEALTH AND BIOSECURITY**		
Diseases incidence	High	• **Publicize RAB vaccinaton program**<br>• Make vaccines more easily accessible in every province
**Low biosecurity measures implementaton**	Low	• **Raise awareness and ofer support for implementaton through extension** services
**4. LACK OF KNOWLEDGE/TRAINING AND SKILLS ON POULTRY FARMING**		
Lack of technical training	Medium	• Improve extension services<br>• Collaborate with young graduates from Colleges of Agriculture and **Veterinary Medicine and of Business and Economics**
Lack of management training	Low	
**5. POOR ACCESS TO CREDIT**		
	Medium	• **Guarantee easier access through (micro)fnance**
**6. MARKET: FREQUENT FLUCTUATIONS IN CHICKEN MEAT AND EGGS PRICE**		
	Medium	• Promote the value of chicken meat and eggs

* **Very high: more than 30% respondents mentoned the issue**
High: between 25 and 30% of the respondents mentoned the issue
Medium: between 10 and 25% of the respondents mentoned the issue
Low: less than 10%/ of the respondents mentoned the issue

** This issue mostly applies to village poultry, as commercial chicken farmers largely **rear exotc breeds.**

OPPORTUNITIES

The challenges listed below can be considered opportunities for investors and traders. The list shows the gaps, which can be filled making use of new technologies, knowledge or perhaps foreign investments.

Training

Poultry farmers in Rwanda are often unaware of certain requirements for rearing healthy and productive chickens and hence, training can have an enormous impact on the sector. Farmers firstly need to gain knowledge on poultry farming and subsequently, they can make the right investments. Businesses should take into account this need for education and proper aftercare when entering this emerging market. Moreover, farmers reported to be lacking management skills that would help them improve and maximize their investments and revenues.

These knowledge gaps highlight an opportunity for development programs **to provide training to Rwandan poultry farmers. Knowledge institutions or development actors (like PUM Senior Experts) with extensive experience** and know-how can collaborate with government or donor programs. These training sessions would have the immediate benefit of spreading knowledge amongst farmers. Moreover, they would raise awareness amongst farmers **regarding possible investments to upgrade their farm productivity. Hence, it would make the Rwandan poultry sector more appealing to potential investors.**

An example of an ongoing program funded by the Netherlands Ministry of **Foreign Affairs is SEAD: Strengthening Education for Agricultural Development. SEAD strengthens linkages between education and the labor market working with education institutions, extension service providers and private sector actors. In the poultry sector, SEAD seeks to tailor education and** training of extension services providers as well as farmers to better fit market demand. The program aims to improve education and training; research and community services in Agricultural production; value chain management and land and water management.

Feed

The decisive factors for future growth of the Rwandan poultry sector are the quantity and quality of poultry feed available. Nowadays, commercial farms often are unable to make use of their full production capacity because **they cannot afford or procure enough raw materials and/or concentrated** premix. This situation could however be improved with increased imports **of premix (and/or raw materials, but this is hardly a problem that can be solved from outside the region). Foreign companies could team up with** local importers to sell their concentrated premix to Rwandan farmers.

A second solution is for a foreign company to initiate a new feed producing plant in the country, benefitting from their experience in **the business (e.g. connection with suppliers of raw materials) and** knowledge. This would allow to offer a high-quality product. If new producers enter the market and have large enough volumes, it could result in economies of scale that would allow them to sell feed at affordable prices. This however still depends on the availability and price of raw materials. High production scale and lower prices would be appealing not only to commercial but also to village poultry farmers, allowing them to obtain higher yields and better nutrition for their households.

In such case, a broader system could be put in place, where the same actor produces – internally or via an outgrowing scheme – the raw materials necessary to the production of poultry feed. However, **this option would require further studies, as available (flat) land is** scarce in Rwanda and could hamper the creation of such a system.

It must be noted that the shortage of poultry feed, but also animal feed in general in Rwanda, cannot be solved by sporadic investments. It requires a **structural targeted intervention. The East-African region is struggling with** underproduction of maize and soybean and consequent rising prices. Alter**native feed components (like cassava or rice) are available and cheaper but** these have limitations; they can only substitute for a certain percentage of maize when fed to layers and broilers. Annex 1 shows possible alternatives to maize and soybean, their characteristics and their limitations.

Breeding and genetics

There is high demand for day-old chicks, which ensures a market for both layer and broiler farms. New hatcheries in Rwanda could offer an alternative **to imports from Europe. Parent lines would still be imported from Europe.** With proper conditions, a large-scale production resulting in economies of **scale, these hatcheries could compete with imported chicks from Uganda.**

Newcomers could compete on the sale of Sasso breed, currently only produced **by Uzima chicken. As seen above, this is a dual-purpose breed, optimal food** security and easy to grow. Moreover, Sasso chickens are reported to be very similar in taste to village chickens, often preferred by locals. Alternatively, **attention can be given to hybrid breed like Cobb 500 (currently imported from Uganda) and Ross 308, that offer good yielding values in the region.**

Access to finance

The provision of loans at affordable interest rates would be highly beneficial for those farmers that practice village poultry farming and sell their products on local markets. Improved access to finance would benefit the poultry sector providing commercial poultry farmers the possibility to expand their businesses and expand larger production scale. This would also benefit **consumers, giving them access to a larger offer at (possibly) lower prices.**

Business development

In Rwanda, eggs are sold in supermarkets, kiosks, and markets; while chicken meat is mostly sold in butcheries and supermarkets. During the past decades, awareness about the important role of eggs in a balanced diet has grown. Purchasing eggs is not as expensive as it used to be, which lead to higher consumption overtime. Differently, poultry meat is still often considered as a food for wealthy people, especially in rural areas.

In terms of business development, opportunites (mainly for development projects) lie in raising awareness about the importance of eggs and poultry meat in one's diet. Moreover, organizations can partner **up with existing projects, such as OneEgg Rwanda or school feeding** programmes. Such initiatives have a strong potential of raising demand for poultry products. Finally, Democratic Republic of Congo and Burundi can be considered as stable pools of demand for chicken meat and egg. Rwandan producers can easily place their products across the borders, often for higher prices than in the domestic market.

CONCLUSION

The poultry sector in Rwanda would benefit from the entrance on the market of actors with availability of capital and technical knowledge. Nonetheless, according to the Dutch exhibitors present at the expo in **Kigali, the Rwandan market is not yet ready for advanced technologies** and hence, for the products they are selling. Production volumes are **still (far) too low for slaughtering plants that have processing capacity** of 500 to 15,000 birds per hour or for automated feeders. Moreover, there is no quality-based market with higher prices for larger eggs and hence, there is no need for egg grading machines. Nonetheless, there is a general agreement that the poultry sector in Rwanda has potential. **The stakeholders present at the Poultry Learning Event and Poultry** Africa 2019 agree that the poultry **market in Rwanda (and in East Africa)** is slowly but steadily growing. Moreover, Rwanda acknowledges and recognizes the potential of poultry as a way to reduce malnutrition and protein deficits. The efforts and commitment of the Rwandan Government to improve the sector are demonstrated by – amongst other things – the publication of the first Livestock Master Plan in 2017, that lays the **foundaton for a further strengthening of the poultry industry in Rwanda.**

REFERENCES

1 Ministry of Agriculture and Animal Resources (MinAgri), 2015. Annual report 2014/2015.

2 Central Intelligence Agency (CIA), 2017. Rwanda: The World Factbook. htps://www.cia.gov/library/publicatons/the-world-factbook/geos/rw.html

3 World Bank, 2019. htps://data.worldbank.org/country/rwanda Accessed in November 2019.

4 Vernooij, A., Masaki, M. N., Meijer-Willems, D. (2018). Regionalisaton in poultry development in Eastern Africa. RAPPORT 1121, Wageningen University and Research.

5 The New Times, June 2016 htps://www.newtmes.co.rw/secton/read/200515; October 2017 htps://www.newtmes.co.rw/secton/read/221541/; December 2017 htps://www.newtmes.co.rw/secton/read/226435

6 Internatonal Livestock Research Insttute (ILRI), Ministry of Agriculture (MinAgri) and Rwandan Agricultural Board (RAB), 2017. Rwanda Livestock Master Plan. htp://extwprlegs1.fao.org/docs/pdf/rwa172923.pdf

7 Mazimpaka, E., Tukei, M., Shyaka, A. and Gatari, E. (2017). Poultry producton and constraints in Eastern Province of Rwanda: case study of Rukomo sector, Nyagatare district. Tropical Animal Health and Producton, 50(4), pp.753–759.

8 Mbuza, F., Denis, M., Janvier, M. and Xavier, R. (2016). Characterizaton of low cost village Poultry producton in Rwanda. Internatonal Journal of Livestock Producton, 7(9), pp.76–82.

9 Food and Agriculture Organizaton (FAO), 2016. Draf report on study on the Situaton of Poultry Sub-sector in Rwanda country.

10 Embassy of the Kingdom of the Netherlands (EKN) (2016). Factsheet Poultry in Rwanda.

11 Mbuza, F., Manishimwe, R., Mahoro, J., Simbankabo, T. and Nishimwe, K. (2016). Characterizaton of broiler poultry producton system in Rwanda. Tropical Animal Health and Producton, 49(1), pp.71–77.

12 FAOstat, 2019. http://www.fao.org/faostat/en/#data. Accessed in November 2019.

13 Natonal Geographic, 2019. htps://www.natonalgeographic.com/foodfeatures/aquaculture/

14 Watagnet, November 2011. htps://web.archive.org/web/20160616092918/htp://www.watagnet.com/artcles/10427-poultry-performance-improves-over-past-decades

15 Shike, Dan W., 2013. Drifless Region Beef Conference 2013, University of Illinois at Urbana-Champaign. htps://lib.dr.iastate.edu/cgi/viewcontent.cgi?artcle=1027&context=driflessconference

16 OneEgg Rwanda, 2020. htps://www.oneegg.org/rwanda/

17 World Food Programme, 2010. School Feeding in 2018. htps://docs.wfp.org/api/documents/WFP-0000110344/download/

18 Natonal Early Childhood Development Programme, 2020. htp://ecd.gov.rw/index.php?id=27

19 Food and Agriculture Organizaton, 2013. Poultry development review.

TRAIDE

ANNEX 1

ALTERNATIVE ENERGY SOURCES		
**Feedstuff**	**Characteristics**	**Maximum use**
Wheat	Can be used when cost competitive. Limitation: high non-starch polysaccharide contents result in intestinal digesta viscosity problems.	Can be used without restriction when exogenous carbohydrates are added
Sorghum	Limitation: tannins lower protein and energy digestibility	Low-tannin sorghum can completely replace maize
Millet	Limitations: high fibre contents, presence of tannins	Can replace 50–65% of maize, depending on millet type
Rice bran/polishing	Limitations: high fibre, phytic acid, rancidity	Good-quality material can be used at levels of 5–10% in broiler diets and up to 40% in layer diets
Wheat bran/pollard	Limitation: high fibre	5% in broiler diets and 15% in layer diets
Cassava root meal	High in starch, excellent energy source. Limitations: low protein, powdery texture, needs detoxification to remove the cyano-genic glucosides	Can be used at levels of 30–40% in nutritionally balanced, pelleted diets
Cassava peel meal	Limitations: high fibre, very high levels of cyanogenic glucosides, needs processing (drying)	Carefully prepared meal may be used at 5% level
Sweet potato tuber meal	High in starch, good energy source. Limitation: powdery texture	Can be used at levels up to 50% in nutritionally balanced, pelleted diets
Banana and plantain meal	Limitation: low palatability due to tannins in the peel; removal of peels improves nutritive value	10–20%
Mango seed kernel meal	Limitation: high levels of tannins	Processed meal can be used at levels of 5–10%
Distillers dried grains with solubles (DDGS)	High fat content (10%), good energy source	25%
**ALTERNATIVE PROTEIN SOURCES**		
**Feedstuff**	**Characteristics**	**Maximum use**
Cottonseed meal	Limitations: high fibre, presence of gossypol. Limit use in layer diets because of effects on internal quality of eggs	Low-gossypol meal can be used at levels of 10–15% in broiler diets
Canola meal	Limitation: glucosinolate	Low-glucosinolate meals can be used at up to 30%
Sunflower meal	Rich in methionine. Limitation: high fibre	15%
Grain legumes (e.g. lupins, field peas, chickpeas, cowpeas, pigeon peas, faba beans)	Limitations: presence of anti-nutrients, low in methionine; current cultivars contain low levels of anti-nutrients	20–30% when processed and supplemented with methionine
Leaf meals, aquatic plant meals	Rich in minerals, moderate levels of protein. Limitations: high fibre, high moisture content and requires drying	Most green meals can be used at levels less than 5%
Distillers dried grains with solubles (DDGS)	Good source of protein, amino acids and available energy. Limitation: variable amino acid availability	Good-quality meals can be used at up to 25%

Adapted from: FAO, 2013[19]

TRAIDE
