# Supplementary material for: Genetic diversity and relationship of Bugesera and Rwamagana indigenous chicken populations with SASSO chickens using DArTseq SNPs
Source: PLoS One. 2025 Sep 12;20(9):e0331316. doi: 10.1371/journal.pone.0331316 (PMC12431124; doi:10.1371/journal.pone.0331316)
Supplement: S2 File — (PDF) [file pone.0331316.s006.pdf]

# Rwanda Livestock Master Plan

## Developed by the International Livestock Research Institute (ILRI)

## Livestock Master Plan (LMP) team


**Dr. Barry I Shapiro, Team Leader and Dr. Getachew Gebru,**

**Dr. Solomon Desta and Mr. Kidus Nigussie**



August 20, 2017

The Rwanda Livestock Master Plan (R-LMP) was prepared by the International Livestock Research Institute (ILRI), with input from the Ministry of Agriculture and Animal Resources (MINAGRI) and Rwanda Agriculture Board (RAB) of The Republic of Rwanda, as well as other Rwandan livestock experts. The study was funded by the United Nations Food and Agriculture Organization (FAO).

## Acknowledgments

Many Individuals, institutions and agencies in Rwanda contributed to the realization of this plan. It was developed under the auspices of H.E. Dr. Geraldine Mukeshimana, Minister of Agriculture, MINAGRI, Government of Rwanda, with oversight provided by Dr. Theogen Rutagwenda, Director General, Animal Resources, MINAGRI. The work of the ILRI LMP team was assisted the Rwandan livestock experts who participated in the expert consultation meetings, provided data and technical advice and validated the results of the livestock sector analysis (LSA). Most particularly the support and guidance provided by Dr. Theogen Rutagwenda and Mr. Otto Muhinda, Assistant Country Representative, FAO-Rwanda helped to bring the LMP to fruition.

The ILRI LMP team would like to acknowledge the African Partnership for Livestock Development (Alive) group, the African Union inter-African Bureau for Animal Resources (AU-IBAR), and the specialists from the Centre de cooperation international en recherché agronomique pour le development (CIRAD), the World Bank, FAO, and ILRI who have developed the set of analytical tools employed in this report: the Livestock Sector Investment and Policy Toolkit (LSIPT) and the Livestock Master Plan tools. The ILRI LMP team used these tools to produce the Rwanda Livestock Sector Analysis and Livestock Master Plan.

## Table of Contents


**Acknowledgments** ........ III

**Rwanda LMP Executive Summary** ........ 1

**Rwanda LMP Introduction and Context** ........ 8

**Rwanda LSA Executive Summary** ........ 16

**Dairy value chain development roadmap (2017/18 – 2021/22)** ........ 24
*Vision* ........ 24
*Overall Targets under the recommended level of investment scenario (RLI)* ........ 24
*Improved Family Dairy (IFD) under RLI* ........ 29
*Commercial Specialized Dairy (CSD) Production under RLI* ........ 43
*Conclusions* ........ 48

**Red meat VC development roadmap (2017/18 – 2021/22)** ........ 51
*Vision* ........ 51
*Overall targets under the recommended level of investment scenario (RLI)* ........ 52
*Improved Traditional Cattle, Sheep and Goat* ........ 55
*Specialized cattle feedlots and culled dairy cattle* ........ 65
*Conclusions* ........ 72

**Chicken value chain development roadmap (2017/18-2021/22)** ........ 75
*Vision* ........ 75
*Overall Targets under the Recommended Level of Investment Scenario (RLI)* ........ 76
*The improved Traditional Family Chicken (ITFC) and Crossbreed Family Chicken (CFC) sub-systems (20167/17-2021/22)* ........ 76
*Specialized commercial chicken production (2016/17-2021/22)* ........ 81
*Conclusions* ........ 100

**Pork value chain development roadmap (2017/18-2021/22)** ........ 103
*Vision* ........ 103
*Overall Target under the recommended level of investment (RLI) scenario* ........ 103
*Modernizing the semi-intensive mixed family pig production system (IFP)* ........ 103
*Expanding Commercial Specialized Piglet fattening systems (CSP) RLI scenario* ........ 106
*Conclusions* ........ 116

## List of tables and figures

Table 1: Percent (%) change in overall national production and contribution to GDP between BAU and the RLI and MLI scenarios in 2021/22 .......... 4
Table 2: Total investment cost required to carry out the recommended Rwanda LMP roadmap 5
Table 3: Species based traditional livestock production systems, herd/flock and specialized .... 12
**Table 4: Percent (%) change in production and GDP between BAU and the "with case", 2031/32** .......... 20
*Table 5* Current and projected number of crossbreed cattle at national and by production system/zone under RLI .......... 25
*Table 6.* Current and projected milk production in Rwanda at national level and by production zone/system .......... 26
Table 7 Annualized milk productivity of cows in Meat-Milk, IFD and CSD systems (liters per year) .......... 27
Table 8 Current and projected GDP contribution of milk in Rwanda at national level and by production zone/system .......... 27
Table 9: Key challenges and strategies related to IFD: .......... 29
*Table 10.* Five year dairy improvement investment capital costs (2017/18 – 2021/22) .......... 35
Table 11. Total investment and share of dairy improvement investment costs from public and private sources .......... 40
Table 12: Activities time line and sequencing: Gantt chart under RLI .......... 41
Table 13: Key challenges and strategies related to CSD under RLI .......... 44
Table 14. Milk production and GDP contribution at three investment level scenarios by 2021/22 .......... 47
Table 15. Five year dairy improvement investment capital costs under the MLI scenario (half the investment of the RLI scenario) .......... 48
Table 16: Annual increase in number of cattle RLI .......... 52
Table 17: Annual increase in number of sheep and goats in the traditional system under RLI.. 53
Table 18: Contribution of cattle to national red meat production (in tonnes) under RLI .......... 53
Table 19: Contribution of cattle Feedlot and dairy sub sector to national red meat production (in tonnes) .......... 53
Table 20: Contribution of Sheep and Goat meat to national red meat production (in tonnes) under RLI .......... 54
Table 21: Key challenges and interventions related to improving red meat VC under the RLI scenario .......... 55
Table 22: Five-year Red Meat production improvement investment costs for RLI, the recommended investment scenario (2017/18 – 2021/22) .......... 59
Table 23:Total investment and recurrent costs for red meat VC development under RLI .......... 61
Table 24: The percent contribution of public and private investments by investment areas for improving the red meat VC under RLI .......... 61
Table 25: Change in red meat production from cattle, sheep and goats under RLI (in tonnes) .. 62
Table 26: Red meat production from local and crossbred cattle by system and typology zone for the baseline year (2016/17) and 2021/22 with the RLI intervention (in tonnes) .......... 62
Table 27: GDP contribution from red meat improvement under RLI (in RWF millions) .......... 63

Table 28: Cattle GDP for baseline year (2016/17) and 2021/22 with red meat intervention in all zones and systems (in RWF 000,000) under RLI ........ 63
Table 29: Activity timeline and sequencing: Gantt chart RLI ........ 64
Table 30: Contribution of the cattle feedlot system to national meat production (in tonnes) under RLI ........ 65
Table 31: Contribution of the dairy production system to national meat production (in tonnes) ........ 65
Table 32: Challenges and interventions in the specialized systems under RLI ........ 66
Table 33: Projected number of cattle fattened under RLI ........ 67
Table 34: Estimated amount of additional concentrate feed needed for additional cattle going to feedlot by the year 2021/22 under RLI ........ 67
Table 35: Investment in slaughter house establishment under RLI ........ 68
Table 36: Change in meat production from cattle feedlots (2016/17 to 2021/22) with feedlot intervention under RLI ........ 68
Table 37: Change in Livestock GDP due to interventions on cattle feedlots under RLI ........ 68
Table 38: Activity timeline and sequencing: Gantt chart RLI ........ 69
Table 39: Rwanda red meat production under BAU and MLI (2021/22) ........ 70
Table 40: Rwanda red meat value added or GDP impacts under BAU and MLI (2021/22) ........ 70
Table 41: Activity timeline and sequencing: Gantt chart MLI ........ 70
Table 42: Total investment and recurrent costs for red meat production under MLI ........ 71
Table 43: Number of hens and chicken eggs and meat production in ITFC and CFC 2016/17-2021/22 (RLI) ........ 76
Table 44: Key challenges and interventions related to improving the family systems (ITFC and CFC) under the RLI ........ 77
Table 45: Changes in GDP contribution of improved family chicken (RLI) ........ 80
Table 46: Number of chicken and chicken meat production in SP chicken subsystems (RLI) ........ 81
Table 47: Egg production from specialized layers (RLI) ........ 81
Table 48: Commercial layer and broiler projected population at national level (RLI) ........ 81
Table 49: GDP contribution from commercial specialized chicken system 2016/17 and 2021/22 (RLI) ........ 82
Table 50: Total chicken meat and eggs production with additional investment RLI ........ 83
Table 51: Total chicken meat and eggs production GDP contribution with additional investment RLI ........ 83
Table 52: Per capita availability of eggs and chicken meat RLI ........ 84
Table 53: Rwanda chicken meat production under BAU and RLI scenario (2021/22) ........ 84
Table 54: Intervention activity timeline and sequencing: Gantt chart RLI ........ 84
Table 55: Five-year Chicken meat and egg production improvement investment costs for RLI, the recommended investment scenario (2017/18 – 2021/22) ........ 87
Table 56:Total investment and recurrent costs for chicken meat and egg production VC development under RLI ........ 91
Table 57: The percent contribution of public and private investments by investment areas for improving the chicken meat and egg production VC under RLI ........ 91
Table 58: Rwanda chicken meat production under BAU and MLI (2021/22) ........ 93
Table 59: Rwanda chicken meat value added or GDP impacts under BAU and MLI (2021/22) ........ 93

Table 60: Intervention activity timeline and sequencing: Gantt chart for MLI scenario ............ 94
Table 61: Five-year Chicken meat and egg production improvement investment costs under medium investment scenario (2017/18 – 2021/22)................................................................. 97
Table 62: MLI and RLI production and GDP comparison ......................................................... 101
Table 63: Number of sows and tonnes of pork in improved family mixed (IFP) pig subsystems (RLI scenario).................................................................................................................. 103
Table 64: Key Challenges and Interventions related to the recommended level of investment (RLI) to improve family pig system (IFP) ................................................................................. 104
Table 65: Pig meat production and GDP contribution family system (RLI) ............................... 106
Table 66: Population growth of piglets fattening and meat production RLI ............................ 106
Table 67: Total pig meat production under RLI ....................................................................... 107
Table 68: Total GDP contribution Family (IFP) and Commercial/Specialized Pig (CSP) under RLI ........................................................................................................................................ 107
Table 69: Activity Sequencing (Gantt chart) under RLI............................................................ 108
Table 70: Five year pig/pork production improvement recommended level of investment costs scenario (2017/18 – 2021/22) RLI.......................................................................................... 109
Table 71:Total investment and recurrent costs for pig production VC development under RLI 111
Table 72: The percent contribution of public and private investments by investment areas for improving the pig production VC under RLI ............................................................................ 111
Table 73: Rwanda total pig meat production and GDP contribution under BAU and MLI scenario (2021/22) ................................................................................................................................ 113
Table 74: Activity sequencing Gantt chart under MLI ............................................................. 113
Table 75: Five year pig/pork production improvement investment costs under medium investment scenario (2017/18 – 2021/22) MLI ......................................................................... 115
Table 76: MLI and RLI production and GDP comparison 2021/22 ............................................ 116

Figure 1: Diagram showing the different modules and sub-modules of LSIPT .......................... 14

# Rwanda LMP Executive Summary

The LMP sets out the investment interventions—better genetics, feed and health services, which, together with complementary policy support—which could help meet the national development plan targets of Rwanda by improving productivity and total production in the key livestock value chains for cow dairy, red meat-milk, poultry, and pork. If the proposed investments—of about USD 287 million over the 5-year LMP period—47% from the public sector and 53% from private sector investors—were successfully implemented, the resulting further modernization of the sector has the potential to have a substantial positively impact on livestock keepers by increasing their incomes and the food and nutritional security of their households. The success of the LMP is critical to the achievement of food security at the sectorial and national levels.

Beyond these positive impacts on rural people, the anticipated transformation of the livestock sector has the potential to impact positively on urban consumers through more and better quality animal source foods and lower animal product prices. The success of the LMP is critical to the achievement of food and nutrition security at household, sectorial and national levels.

**Development of the LMP**

The LSA and LMP interventions, based on investment scenarios related to productivity enhancing technologies and improved policies developed or identified by livestock experts of MINAGRI, RAB, and other research institutes and universities in Rwanda were tested in accordance with the Rwandan national development objectives:

- Reduce poverty
- Achieve food and nutritional security
- Contribute to economic growth
- Contribute to exports and foreign exchange earnings
- Contribute to industrialization and employment
- Mitigate **livestock's** contribution to GHG emissions

Using measurable economic or environmental indicators for the above objectives, four key livestock value chains—live animals and red meat and milk (from indigenous cattle, sheep, and goats), dairy with crossbred cows, and chicken and pigs—were identified in the LSA as producing the greatest productivity increases contributing to national economic development objectives and the long-run development of the sector. The LMP, moreover, comprises two sub-value chains: smallholder family and commercial specialized production systems. These sub-value chains are found in one or more of the four major production typology zones of Tanzania: central; coastal and lake; highlands; and commercial specialized dairy. The rigorous ex-ante technical and financial analysis conducted of alternative intervention options (investment scenarios) carried out in the LMP is thus a guide to the choice and prioritization of public and private investments with the highest payoffs for livestock sector transformation.

**LMP Commodity Value Chains: Rwanda**
Based on results of the LSA analysis, to reach the objectives and goals of the GoR, the key VCs targeted in the LMP roadmaps are:

1. Cow dairy
   Improved family dairy
   Commercial specialized dairy

2. Red meat (and milk) from cattle, sheep, and goats
   Improved traditional red meat-milk
   Ranches
   Specialised feedlots

3. Chicken meat and eggs
   Improved family chicken
   commercial specialized chicken (layers and broilers)

4. Pork
   Improved traditional system (semi-scavenging or stall fed)
   Commercial specialized piglet fattening

**Key results**

**Cow dairy VC development**
The projected increase in national cow milk production as a result of the proposed interventions—due to an increase in crossbred and pure exotic cows, including through artificial insemination and synchronization, combined with improved feed and health interventions, value addition and complementary policy changes—during the LMP period (2016/17–2021/22) is 2.2 billion liters of milk, an 18.2% increase in milk production over the BAU investment level given the RLI scenario. The crossbred dairy cow herd increases by 46%, milk production by 65%, and contribution to GDP by 53%, while improving the processing and marketing of dairy products. This additional milk production could then be substituted for imported milk products and used domestically for new or additional industrial uses (e.g. in the baking industry), or exported as milk powder UHT or casein milk protein to raise foreign exchange earnings.

**Red meat VC development**
Under the RLI, the increases in red meat production from cattle, sheep and goats from 2016/17 (the baseline year) to 2021/22 is 32%, 33%, and 50 %, respectively. Cattle (72%) and goats (23%) are the primary sources of red meat in 2021/22. Total red meat grows from 58,579 tonnes in 2016/17 to 79,586 tonnes in 2021/22, an increase by 36%. Under RLI the GDP contribution from red meat coming from cattle, sheep and goats from all production zones shows an overall increase by 26% compared to the base year with the 2021/22 projection. The

GDP contribution of red meat coming from cattle from all production zones and systems shows an overall increase by 19% comparing the base year with the 2021/22 projection.

The negative change in production of meat from local cattle breeds is negative 19% and big positive production change in crossbreds in all the production zones is 47%, with similar increases and decreased in meat production from crosses and locals, respectively. This negative change in meat production from local breeds is expected due to the population decreases of local cattle breeds as they are replaced by the crosses or pure exotic breeds. The *Girinka* program or **"One Cow per Family Program"** of MINAGRI is transforming dairying and meat production in Rwanda, through the provision of pregnant crossbred cows to smallholders. As well, the number of cattle going through the feedlot fattening system for meat production is **expected to increase rapidly, partly as a result of the government's Gako program which is** seeking to improve the meat breeds in Rwanda, especially with Ankole cattle.

Due to extremely limited access to land for grazing and feed production, and limited ability to enhance the genetic potential of local ruminant breeds in the medium-term, it is unlikely the red meat production gap can be closed in the next five years.

**Chicken VC development**

Successful poultry interventions would allow the poultry sub-sector to move to improved family poultry with semi-scavenging crossbreds and for substantial increases in the scale of specialized layer and broiler operations. Such a transformation—depending on successful interventions in the areas of breed selection, health services, particularly in treating Newcastle disease, feed, extension, private investment and trade policies—would contribute considerably to improving food and nutrition security and household, as well as increasing the contribution of poultry to GDP.

The chicken meat and egg production under the RLI scenario show substantial increments, 124% and 110% respectively compared to the base year. The RLI scenario would contribute significantly to fulfilling the national all meat requirement and would increase per capita egg consumption for Rwanda to help meet one of the Rwandan national development objectives of improving food and nutrition security. Under the RLI scenario, although the per capita egg consumption increases, a significant shortage continues to exist to meeting the FAO recommended requirement, which is 4.5 kg per person.

Taking advantage of the benefits of the potential poultry revolution would require substantial investments in promotional activities to change tastes and preferences from beef, as well as from local to exotic chicken meat and eggs. The substitution of the surplus chicken meat for domestic red meat consumption would also put downward pressure on domestic meat prices and enable an increase in the export of live animals (of cattle, sheep, and goat), potentially raising foreign exchange earnings.

**Pork VC development**
The pig meat production and GDP impacts under the RLI scenario show substantial increments, 239% and 214% respectively, compared to the base year. The RLI would contribute significantly to fulfilling the national all meat requirement and would increase per capita pig meat consumption for Rwanda enough that it would help to meet the Rwandan national development objective of improving food and nutrition security.

It should be noted that improving pork meat requires a focus on upon controlling African swine fever pigs, to increase pig productivity and meat production to help close the projected all-meat consumption gap projected in 15 years. Industrializing pork production (in large commercial scale operations and processing for product transformation will lower domestic meat prices, while enabling an increase in exports and foreign exchange earnings.

**Comparison of RLI an MLI**
The LSA investment interventions under RLI result in production of 663 thousand MT of meat, 2.2 billion liters of milk, and 1.9 billion eggs which would substantially increase availability of ASFs in Rwanda to help meet the nutritional requirements of the rapidly growing human population, expected to reach 16.3 million people in 2031/32.

Compared to the BAU, the MLI impact on meat production and GDP contribution show significant declines, indicating the MLI would not be warranted before conducting a thorough financial feasibility analysis, to calculate the benefit-cost ratio (BCR) and net present value (NPV). It is strongly recommended to do a financial feasibility analysis of the MLI scenario before committing the investment resources, no matter how great the budget constraint may be to committing to the RLI.

Table 1: Percent (%) change in overall national production and contribution to GDP between BAU and the RLI and MLI scenarios in 2021/22

| | **BAU** | | **MLI/BAU** | | **RLI/BAU** | |
|---|---|---|---|---|---|---|
| **Livestock products** | Production in tonnes (MT) | GDP in RWF (10^6) | Production (% change) | GDP (% change) | Production (% change) | GDP (% change) |
| Beef | 53,729 | 82,410 | 3% | -4% | 7% | -5% |
| Mutton | 3,171 | 5,538 | 6% | 5% | 16% | 12% |
| Goat meat | 16,050 | 31,615 | 5% | 4% | 15% | 11% |
| Pork | 26,665 | 28,184 | 70% | 76% | 154% | 135% |
| Chicken meat | 20,589 | 27,278 | 35% | 15% | 40% | 71% |
| Total Meat | 120,204 | 175,025 | 24% | 14% | 62% | 28% |
| Milk | 1,041 | 158 | 4% | 0% | 18.2% | 9.5% |
| Eggs in million | 369.7 | 16,328 | 17% | 17% | 53% | 39% |
| Hides & skins | 1,322 | 624 | - | - | 15% | 15% |
| Organic matter | 8,251,268 | 9,178 | - | - | 7% | 57% |

**Priority investment and complementary policy interventions**

The huge deficit in projected demand (without additional investments at the RLI level) for red meat is being driven by high human population and urbanization growth, as well as income growth. Limited access to land for improving feed production, including on grazing lands, and the low genetic potential of local breeds of cattle and small ruminants are the main constraints to increased red meat production. That said red meat from small ruminants, especially sheep will be of little help in closing the meat gap due to their low numbers, limited feed resources and low genetic potential of indigenous breeds, nor will pork help much unless outbreaks of African swine fever are controlled effectively.

**Total investment in the Rwanda LMP**

The total investment costs required to carry out the LMP roadmap are RWF 237,969.7 million. The proportion of investment from the public and private sectors is 47% (RWF 112,580.85 million) and 53% (RWF 125,388.75 million) respectively.

Table 2: Total investment cost required to carry out the recommended Rwanda LMP roadmap

| Investment interventions | Total investment cost in million RWF | | | Total Cost in million USD |
|---|---|---|---|---|
| | Public | Private | Total | |
| Cow dairy | 21,943.1 | 28,524.5 | 50,467.7 | 60.8 |
| Red meat/milk and feedlot | 73063 | 57464 | 130,527 | 157.3 |
| Poultry | 16,320 | 34,680 | 51,000 | 61.4 |
| Pork | 1254.75 | 4720.25 | 5,975 | 7.2 |
| Total | 96,277.17 | 90,743.43 | 187,020.7 | 286.7 |

*US$ 1 = RWF 830

**Key take-away messages**

Investment in poultry has the most potential to close the projected meat consumption gap and could enable export of ruminant animals and red meat.

However, domestic consumer preferences for white meat and particularly chicken meat would need significant investment and effort in promotion to change consumer preferences for red meat, especially beef and goat meat

The projected gap in milk demanded could be closed and a surplus produced through AI and synchronization for breed improvement, combined with feed and health interventions addressing young and adult stock mortality (YASM)

Feed is the biggest constraint to animal productivity improvement. Access to land appropriate for grazing, and land for feed production, needs to be addressed to overcome the very serious existing feed deficit

Red meat production cannot be expected to increase much over time and help significantly to close the projected all-meat production-consumption gap due to the present limited access to land for feed production and grazing, the need to expand animal health services, and the low genetic potential of local cattle breeds and small ruminants

Small ruminants are also not expected to be help much in closing the meat gap due to their low numbers, in addition to limited feed resources and low genetic potential of indigenous breeds

Pork has potential to help close the projected all-meat production-consumption gap but it is prone to African Swine Fever requiring improved prevention and control, and its demand is limited; hence it cannot be a priority solution for closing the meat gap

**Priority investment interventions**

Various combinations of the three standard types of livestock technology interventions are needed to generate higher incomes and animal productivity, and to lead to the achievement of national development objectives: improved genetics, health and feed. The appropriate combinations, depending upon the biophysical, agro-ecological and market conditions facing livestock in the four production zones in Tanzania, include:

Ensuring artificial insemination and synchronization, feed and health interventions addressing young and adult stock mortality (YASM) to help facilitate a surplus in milk demanded;

Targeting animal health interventions for young and adult stock mortality (vaccinations, parasite control) ensuring improved productivity, thereby increasing animal and product off take of meat and dairy;

Prioritizing beef production from on-farm fattening and commercial feedlots as a way of reducing the red meat deficit;

Feed is the biggest constraint to animal productivity improvement. Access to land appropriate for grazing, and land for feed production, needs to be addressed to overcome the very serious existing feed deficit

Improving the quality and quantity of livestock feed resources through introducing improved forage crops and improved animal feed management practices, feed production on irrigated land, as well as increased access to existing lands appropriate for grazing;

**Complementary policy interventions**

Animal health services need to expand dramatically, especially in remote areas where pastoralists predominate, and PPPs should be tried where private investments are too risky and the returns are too low;

Undertaking investments in promotional activities to change tastes and preferences from beef to white meat, especially chicken;

Prioritizing investments in genetic improvement by focusing on crossbreds and exotic chicken pure breeds for both family and commercial enterprises;

Prioritizing policies creating a conducive environment for investment in commercial meat and milk production and processing;

Promoting land allocation and ownership policies which facilitate the investments required to increase feed for meat, egg and milk production;

Promoting land leasing, including land under irrigation, for animal and feed production and providing of tax incentives and subsidized leasing rates to private entrepreneurs;

Promoting exports to more remunerative markets in the region through the introduction of a practical and affordable system of animal ID and traceability, as well as food safety and animal health programs through the monitoring of abattoirs and disease surveillance;
Promoting substantial private investment in livestock product transformation through high value-added processing; and
Improving the enabling environment for agribusiness investment through the streamlining of regulations and procedures in order to attract and maintain substantial levels of private investment.

# Rwanda LMP Introduction and Context

In recent years, the government of Rwanda has prioritized the transformation of the agricultural sector. Due to agro-ecological conditions conducive to intensified livestock production, and despite very limited land, the livestock sector of Rwanda provides major opportunities to increase further its contribution to economic growth (Rwanda having already achieved above 6% annual GDP growth for the last 10 years) while improving incomes to reduce poverty and improve the nutritional security of many rural people. This potential has **already been proven by the "One cow per family program" that has transformed dairying in** Rwanda, and proven to be the key to sustainably improving vegetable and legume production on once infertile hillsides by providing ample manure. Recent growth in livestock production largely reflects significant productivity gains. As well, with rapidly growing population, increasing urbanization and rising incomes, the demand for meat, milk and eggs is expected to increase significantly for the foreseeable future.

Still, clear investment roadmaps to further develop the livestock sector could bring even greater impacts from future sector investment plans. The sector is still constrained by too many local animals with low reproductive rates, and high morbidity and mortality rates. Detailed inter-disciplinary livestock sector analysis (the LSA) revealed the potential benefits of a comprehensive livestock master plan (LMP) for Tanzania.

The Rwanda LMP shared here sets out the investment interventions—better genetics, feed and health services, which, together with complementary policy support—which could help meet **Rwanda's national development targets by further improving productivity and total production** in the key livestock value chains for cow dairy, red meat (and milk) from cattle, goats and sheep, poultry (chicken meat and eggs), and pork. If the proposed investments—of USD 287 million—47% from the public sector and 53% from private sector investors—were successfully implemented, the anticipated transformation of the sector has the potential to impact positively on rural livestock keepers by increasing their incomes and food and nutritional security, and on urban consumers through lower animal product prices. The success of the LMP investment roadmaps is critical to also making further progress in food security at the sectorial and national levels.

### Development of the LMP

The Rwanda Livestock Master Plan (LMP) was undertaken by the International Livestock Research Institute (ILRI) with input from the Ministry of Agriculture and Animal Resources (MINAGRI), the Rwanda Agriculture Board (RAB), and other research institutes and universities of The Republic of Rwanda. The study was funded by the Food and Agriculture Organization (FAO) to develop an evidence based LMP built on quantitative analysis of the sector.

Using available data from secondary sources and national livestock experts, the ILRI LMP team, with input from Rwanda livestock experts (including from MINAGRI, RAB, other research institutes and universities, private sector and NGO actors, etc.) developed a national herd and economic sector model (HESM) and then carried out an assessment of the current state of the sector to establish a data baseline for 2016/17. Then, with the HESM, the team developed long-term targets and then did long-term foresight analysis to identify the most promising investment interventions and policies to arrive at a long-term strategy for livestock development in Rwanda over 15-years (a Livestock Sector Analysis (LSA)). The LSA also presents the current status and future potential for poverty reduction and economic growth through the further modernization of the livestock sector.

The results of the LSA was used, in turn, to guide the preparation of the Rwanda Livestock Master Plan (LMP) which is a series of five-year investment implementation plans or **'roadmaps', to help prepare and implement the next 5**-year national development program to further modernize the Rwandan livestock sector. The LMP will also help realize the development vision of Rwanda, namely the: Rwanda Development Vision 2025 to 2050 (Ministry of Finance and Economic planning 2016).

This LMP seeks to inform Government of Rwanda (GoR) policymakers, private investors, and development partners (DPs or donors) involved in livestock development on the priority livestock commodity value chains and investment interventions (combining technologies and policies) which could transform the sector through further investment.

The Livestock Master Plan (LMP) is a series of 5-year investment implementation plans or roadmaps for the key livestock commodity value chains (VC), and production systems within **each value chain, chosen based on the GoR's priority development objectives. They each** include specific VC visions and targets, challenges and strategies, and the priority combined technology and policy investment interventions, with expected outputs, outcomes and impacts. The roadmaps are also fully budgeted, and include timed and sequenced activity plans (Gantt charts).

To identify the priority combined technology and policy investment interventions for each LMP roadmap, the impacts of alternative technology and policy investments needed to be assessed against the agreed upon national development objectives of Rwanda. Indicators for the objectives were also needed. The following national objectives and indicators were identified and agreed upon with key Rwandan livestock sector stakeholders. The indicators for these objectives then became the decision criteria to compare the alternative available investment interventions (combined technology and policy) and choose the priority investment interventions in both the LSA and LMP. The objectives and indicators were:

Reducing poverty (by increasing income per animal and in livestock keeping households)

Achieving food and nutritional security (by increasing household herd and national production levels and by also increasing availability of ASFs to meet FAO individual consumption targets)

Increasing economic growth (by increasing livestock contributions to GDP (or national income))
Increasing exports (by increasing production beyond domestic consumption requirements which improves the potential for export of live animals and livestock products, and realization of foreign currency)
Contributing to industrialization and employment (by increasing investment in processing and other value addition)
Mitigating climate change (by focusing more on introducing low emitting livestock species for meat (chicken and pork), and dairy intensification through introduction of higher productivity crossbreds) to reduce greenhouse gas (GHG) emissions)

Using the measurable economic or environmental indicators for the above objectives, four key livestock value chains were identified in the LSA—red meat (and milk) (from indigenous and crossbred cattle, sheep, and goats), dairy from crossbred cows, chicken and pork—as producing the greatest potential productivity and production increases in the long run, as well as potential contributions to national economic development objectives under the recommended levels of investment, and thus the potential to contribute to the long-run development of the sector. The LMP, moreover, comprises two types and/or scales of operations for each of these value chains: smallholder family and commercial specialized production systems. These systems are complementary in the sense that they support the development of the other so both types are included in the LMP.

**Rwanda LMP priority commodity value chains**

Based on results of the LSA analysis, to reach the objectives and goals of the GoR, the key VCs targeted in the LMP roadmaps are the following, along with the main investment interventions to improve them and increase their development impact:

**1. Cow dairy**

**Improved family dairy (IFD):** smallholder family dairying practiced in all zones, usually with 1-2 milking cows and improved with crossbreds or exotics, along with improved feed and health services. IFD is practiced by farmers in mixed crop-livestock production systems, uses small levels of inputs and results in a moderate level of milk production per cow.
**Commercial specialized dairy (CSD):** both the grazing based (i.e., Gishwati) and non-grazing based or stall fed (all zones) commercial scale specialized dairy production systems with high level of inputs and high milk productivity or yields. The main LMP intervention is increasing the number of crossbred and/or exotic cows.

**2. Red meat (and milk) from cattle, sheep, and goats**

**Improved family red meat-milk (IFRM):** these family systems are practiced in all production zones and the main LMP improvements are in feeding and animal health (vaccinations and dipping or spraying for parasites), but improvement of local cattle and goats and crossbreeding is also analyzed.

**Ranches:** meat production on big commercial farms or ranches practiced in areas designated for improved ranching systems in the low rainfall production areas (e.g., Gako); the improvements entailing better feeding, animal health and genetics and better linking to marketing and slaughter houses

**Cattle specialized fattening or feedlots (CSF) –**- using both indigenous breeds and crossbred males, with the main interventions being to increase the number of animals and production units, and better linking to marketing and slaughter houses

**3. Chicken meat and eggs**

**Improved traditional family chicken (IFC):** IFC can be practiced all over the country and in all livestock production zones (low, medium and high rainfall zones), using local breeds with improved productivity through selection, along with health, feed and management interventions, especially improved brooding, and scavenging or semi-scavenging feeding; or distribution of higher yielding tropical pure and crossbred chicken to producers.

**Commercial specialized chicken (CSC):** scaling up of the commercial chicken broiler and layer systems and better integration of production units with the upper end of the value chain.

**4. Pork**

**Improved traditional family pig system (IFP), both stall fed and semi-scavenging systems:** improvement of the mixed semi scavenging can be practiced in all the livestock production zones (low, medium and high rainfall and altitude zones); the proposed interventions involve improving the semi-intensive family mixed production system.

**Commercial specialized piglet fattening system (CSP):** the specialized semi-intensive piglet fattening system can also be practiced in all the livestock production zones (low, medium and high rainfall and altitude zones); the improvement is intensification and expansion of the piglet fattening system.

These priority value chains are found in one or more of three major production typology zones of Rwanda (classified thus to facilitate the sector modelling and model analysis):

In defining the three major production typology zones, rainfall or moisture and altitude are closely linked with the quantity and type (quality) of forage production and extent of use of crop residues and other feed supplementation which would impact on the performance of livestock and delimit the kind of production practices. Hence, the classification or the livestock production systems into zones helps to capture variability in the productivity performance of the different livestock species and systems, as well as identify opportunities and constraints which exist within and across the production zones.

The three livestock production zones used in the Rwanda LSA and LMP are then:

Low rainfall low altitude livestock production zone (LRLA): 800-1000 mm and 1450 to 1500 meters asl

Medium rainfall medium altitude livestock production zone (MRMA): 1000 – 1400 mm and 1500-1800 meter asl
High rainfall high altitude livestock production zone (HRHA): 1400 – 1800 mm and >1800 meter asl

The LRLA zone predominantly covers the eastern savanna and the eastern plateau of Rwanda and the MRMA zone is found predominantly in the central plateau and the HRHA covers the western highland, Lake Kivu and South West regions.

The livestock production practices whose production methods are modern, and mainly depend on the procurement of feed from outside the farm are to a large extent independent of the zonation and are classified as a fourth livestock systems category. These are defined as specialized commercial production (SP) systems and are analyzed independently due to their unique modern production characteristics and commercial orientation. They can be found throughout most of the country and are thus often independent of agroecology (rainfall and altitude).

The specialized commercial livestock production (SP) systems are classified as follows:
**Dairy Systems (CSD) –** specialized grazing and non-grazing based commercial systems, both using crossbreds and exotic breeds.
**Cattle Fattening Operations (CFS) –**- using both indigenous breeds and crossbred males.
**Commercial specialized chicken (CSC) –** differentiated into commercial broiler and layer operations using exotic breeds
**Commercial specialized piglet fattening (CSP) –** stall feeding and using crossbreds and exotic breeds, as well as professional health management

The following priority livestock systems were then classified for the purpose of assessing and recommending potential system specific technology and policy interventions to modernize and transform the sector. The lower level systems classifications are species/breed based (cattle, sheep, goats, chicken and pigs) and associated with herding practices.

Table 3: Species based traditional livestock production systems, herd/flock and specialized

| **Livestock systems by species** | **LRLA** | **MRMA** | **HRHA** | **Specialized** |
|---|---|---|---|---|
| Cattle system | Local breed | Local breed | Local breed | Non-grazing dairy |
| | Family dairy (Cross breed) | Family dairy (Cross breed) | Family dairy (Cross breed) | Grazing dairy |
| | Family dairy (Cross breed) | | | Fattening |
| Sheep system | Local Breed | Local Breed | Local Breed | |
| | Cross Breed | Cross Breed | Cross Breed | |

| Livestock systems by species | LRLA | MRMA | HRHA | Specialized |
|---|---|---|---|---|
| Goats systems | Local Breed | Local Breed | Local Breed | |
| | Cross Breed | Cross Breed | Cross Breed | |
| Chicken systems | Family | Family | Family | Layers |
| | | | | Broilers |
| Pigs systems | Family | Family | Family | Pig fattening |

**Analysis of Investment scenarios**

The rigorous ex-ante technical and financial analysis conducted on alternative investment intervention options (investment scenarios) carried out in the LMP is meant to be a guide to the choice and prioritization of public and private investments with the highest payoffs for livestock sector transformation by system and production zone.

In the investment analysis done to create the LMP roadmaps, two investment scenarios are usually analyzed, BAU (Business As Usual) and RLI (Recommended Level of Investment). Here, three levels of investment and their implications for commodity value chain contributions to production and GDP are examined to provide three investment choices for the decision makers in Rwanda; the government, private investors and development partners (donors). These scenarios are referred to here as BAU (Business As Usual or the base case), MLI (Medium Level of Investment) and RLI (Recommended Level of Investment). BAU presents the scenario analysis of the base case to understand the implications of the current level of investment and spending profile on chicken meat and eggs production and GDP contributions throughout the LMP period of 5 years. The second, RLI, is the recommended level of investment which could achieve the agreed upon targets for the national development objectives.

The additional scenario or MLI option analyzes the implications and impacts on production and GDP of a third option created for investment decision makers who face a budget constraint. It represents a medium level of investment between the BAU and the RLI option which could achieve the 3$^{rd}$ year impact of the targets for production and contribution to GDP under the RLI. For this 3-option analysis, the MLI level of investment was obtained by assuming the RLI option would only be followed through the 3$^{rd}$ year (see table 1 above) of the 5-year LMP period.

**Methodology**

**LSIPT - Livestock Sector Investment and Policy Toolkit**

The ILRI team used LSIPT, the Livestock Sector Investment and Policy Toolkit, to build the herd and sector model (HESM) to then carry out the analysis to produce the LSA and LMP.

Spearheaded by AU-IBAR, LSIPT was developed by livestock experts at CIRAD, FAO and the World Bank. It had been tested in Mali and Zambia, and is now operational and has been applied in Ethiopia and Tanzania by the ILRI LMP team and national collaborators from the ministries of agriculture and/or livestock.

LSIPT integrates micro, meso, and macro analysis for quantitative and qualitative assessment of household vulnerability, the role of livestock in strategy for poverty reduction, and the contribution of livestock to the overall economy. It accounts for the multiple functions of livestock, including: those of cultural importance; the contribution to food security and nutrition; and the supplier of draught power and manure for soil fertility.

LSIPT provides a systematic framework for organizing accessible quantitative data (mostly from secondary sources), and includes tools to carry out analyses which help to understand the production potential of the sector, and its contribution to agricultural and overall economic growth (GDP), as well as reduction in rural poverty and food insecurity. Furthermore, LSIPT enables the running of alternative technology and policy scenarios to gauge the supply response of potential government investments in research and extension (such as technologies that impact on feed availability, animal health, etc.), as well as private sector investments over 5 to 15 year projection periods. The scenario analysis is transparent and readily understandable, and thus useful to policy makers and development investors. Moreover, analysis of potential impacts from changes in key aspects of policy, such as the enactment of food quality standards and regulations required to compete in formal markets (including export markets) can also be evaluated with the ALive toolkit. Further description of the LSIPT methodology is given in the diagram (below).

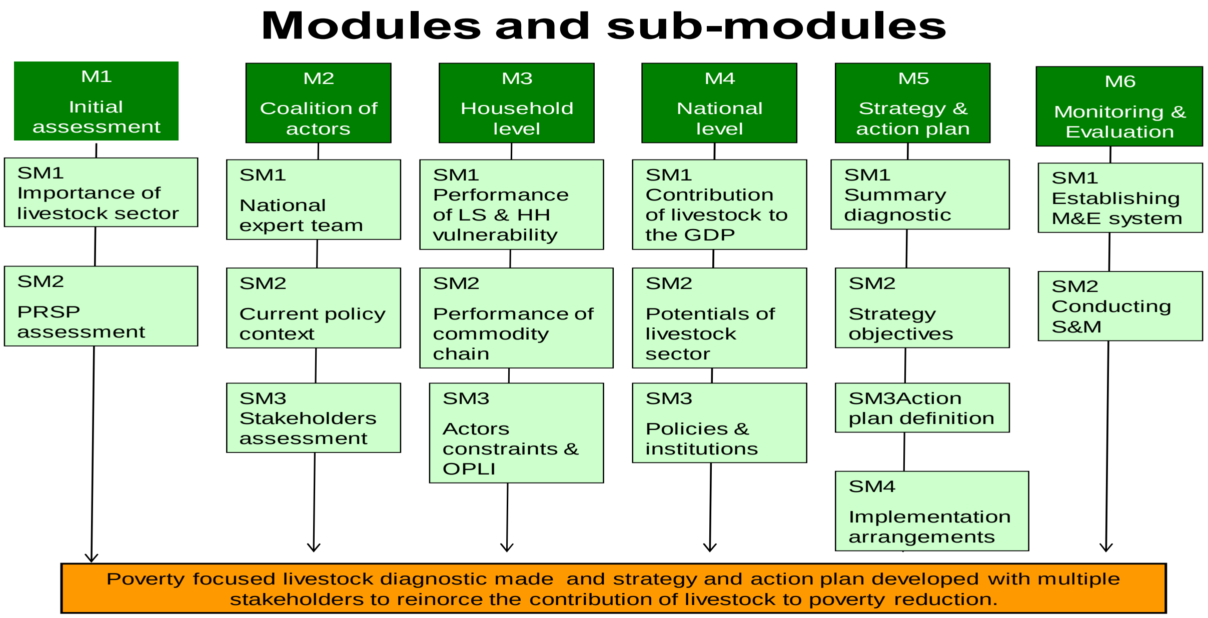


Figure 1: Diagram showing the different modules and sub-modules of LSIPT

The core elements of LSIPT are the modules 3, 4 and 5. Module 3 assesses the productivity at household, value chain, and production systems level. These micro and mesa data figures are then consolidated and extrapolated to the national level in module 4. Once this data base is established, the participatory tools of module 5 can be used to identify, with all stakeholders, the priority sectors, target groups, and the most effective technology and policy interventions, to ensure optimal use scarce human and financial resources. Once the interventions are identified, financial, economic, social and environmental impacts can be rapidly assessed using the database established in modules 3 and 4. Finally using the tools, the implementation arrangements can be established on a mutually agreed upon basis.

Key information and data needed for the quantitative analysis carried out in Module 3 and 4 include:

a. A typology of the main different production systems prevailing in the country, with their respective number of livestock keeping households and livestock numbers
b. Main livestock performance data for each species of livestock in each production system in each typology zone, with a breakdown by age group and sex, reproductive performance, mortality by age group and sex, average production, live-weight, off-take rate, carcass weight, etc.
c. Feed requirements by species, age and sex of animals, as well as feed availability, as related to land and water availability for forage production, and crop residues and industrial byproducts
d. Dry matter yields of grass and crop lands (for crop-residues estimates for animal feed) in each of the main agro-ecological zones

Available data and parameters required for the herd models were collected from published **papers and consultancy reports, as well as other "grey" literature. Gaps were filled through** consultations with national experts. For the household survey data where required, available survey data collected by the Central Statistics Bureaus and research organizations was used, and this data was assessed to determine how representative the surveys are, and gaps filled in as needed.

# Rwanda LSA Executive Summary

This Rwanda Livestock Sector Analysis (R-LSA) was undertaken by the International Livestock Research Institute (ILRI) with input from the Ministry of Agriculture and Animal Resources (MINAGRI) and Rwanda Agriculture Board (RAB) of The Republic of Rwanda. The study was funded by the Food and Agriculture Organization (FAO) to develop a fact based LSA and Livestock Master Plan for Rwanda (LMP).

Using available data from secondary sources and national livestock experts, the International Livestock Research Institute (ILRI), developed a herd and livestock sector model and then carried out an assessment of the current state of the sector (for 2016/17) to inform the long-term potential for livestock development in Rwanda over 15-years (a Livestock Sector Analysis (LSA)). The results of this LSA will guide in turn the preparation of the Rwanda Livestock Master Plan (LMP) which is a series of five-**year investment implementation plans or 'roadmaps', to be** used to direct the implementation of the national program to further modernize and transform the Rwandan livestock sector. It is also meant to help realize the development vision of Rwanda, namely the: Rwanda Development Vision 2025 to 2050 (Ministry of Finance and Economic planning 2016).

This report seeks to inform Government of Rwanda policymakers, private investors, and development partners (DPs or donors) involved in livestock development on the current status and future potential for poverty reduction and economic growth of the livestock sector.

**Development of the LSA**

The LSA interventions, based on investment scenarios related to productivity enhancing technologies and improved policies developed by MINAGRI, RAB and other Rwandan research institutes and universities, were tested in accordance with agreed upon national development objectives. The criteria used to assess the investment interventions were to:

- Reduce poverty
- Achieve food security
- Contribute to economic growth
- Contribute to exports
- Contribute to industrialization and employment
- Mitigate contribution to GHG emissions

Using measurable economic indicators for the above objectives, four key livestock value chains: dairy with crossbred cows; red meat and milk (from cattle, sheep, and goats); chicken for meat and eggs and pork (both white meat) **–** were identified in the LSA as having the most potential

for productivity increase through new investments to achieve these national economic development objectives and contribute most to the long-run development of the sector.

A summary of the ***findings*** of the report shows:

The national herd, consisting at present (in 2016/17) in about 1.39 million cattle, 700 thousand sheep, 2.94 million goats, 1.8 million pigs and about 7 million layers, broilers and indigenous chicken, produces currently about 94.2 thousand Metric Tons (MT) of meat, 747 thousand MT of milk, and 243 million eggs per year. In addition the herd provides about 6.8 million MT of organic fertilizer.

About half (46%) of the dressed meat in Rwanda comes from cattle, followed by swine (21%). Chicken and goats make up 17% and 13% of dressed meat respectively, and sheep only 3%.

Sixty five percent (65%) of domestically consumed beef comes from crossbred cattle. Meanwhile, for mutton and goat meat only 26 and 8% come from crossbreds, respectively.

The contribution of the local cattle breeds to total milk production is only 9%, though they represent 43% of the national cattle herd.

Although the local, indigenous chicken now constitute 75% of the chicken population, they produce only 32% of the chicken meat and 34% of the eggs in the village chicken systems. Sixty eight percent (68%) of the chicken meat comes now from specialized broilers and layers and 66% of the eggs from specialized layers.

To facilitate the use of an analytical model created for this LSA study, a typology of the different livestock systems was developed, consisting at the first level in the (a) Low Rainfall Low Altitude (LRLA) zone which predominantly covers the eastern savanna and the eastern plateau of Rwanda with a significant feature of farmland based grazing; (b) the medium rainfall and medium altitude (MRMA) zone which is predominantly found in the central plateau of Rwanda; and (c) the high rainfall high altitude (HRHA) which covers the western highland, Lake Kivu and South West regions. Livestock production practices which are modern, and which are to a certain extent independent of location and the broader zonation are classified as specialized production (SP) systems and analyzed independently due to their unique modern production characteristics and commercial orientation.

The geographic distribution of ruminant livestock (cattle, sheep and goats) show that they are not equally found across the three production zones. The LRLA and MRMA production zones support 74% of the cattle population and the HRHA 25%. The specialized cattle system is at its initial development stage. Most of the sheep are found in the HRHA (48%) and MRMA (43%) production zones. Sheep production in the LRLA zone (9%) is minimal at

present. On the other hand the largest proportion of the goats (40%) are found in the LRLA zone, followed by the MRMA (31%) and the HRHA (29%) production zones.

Fifty one percent (51%) of the national cattle herd are crosses, 6% pure and 43% locals/indigenous. This denotes that a significant achievement of dairy sector modernization has already accomplished by Rwanda.

Based on the sector and herd model projection, the direct and indirect contribution of livestock to GDP in 2016/17 is estimated at RWF 268 billion ($321 million)[1], which amounts to 4.6 percent of national GDP and 14% of agricultural GDP in 2015. This contribution at the production stage does not include value added by the livestock value chains.

The largest livestock value added now is generated by dairy cow milk production which accounts for about 42%, followed by beef which accounts for 25%. Overall, including organic matter and hides, cattle accounts for about 71% of the livestock value added showing the continuing importance of the family cattle production system in Rwanda. Value added from goat meat amounts to about 10%, mutton 2% and the remaining 16% come from pork (8.4%) and chicken (8). The contribution from the specialized cattle, chicken and pig systems is less than 5%. Family cattle production for meat and milk cannot be ignored in any livestock sector development plan.

In the case of meat, if the proposed LSA investment interventions are successfully implemented, the results project production of 663 thousand metric tons of meat in 2031/32 which is 3.5 times the production under the business as usual (BAU) scenario over the same period. This increase in production would increase the per capita availability of meat from domestic production to 41 kg per capita, despite the fact that the human population in Rwanda is projected to increase, by38% to 16.3 million in 2031/32 from 11.8 million in 2016/17 (NISR 2014).

To increase the future per capita meat availability, it will be essential that both semi intensive family pig and specialized piglet fattening systems and the number of specialized **broiler units be vastly increased. According to the LSA results the "with intervention"** scenario, shows that 95% of the additional meat in 2031/32 would come from chicken (37%) and pigs (58%).

Overall, in 2031 with the LSA intervention, 80% of the total meat comes from pigs (48%) and chicken (32%). Cattle would contribute only 14%, goats 5% and sheep less than 1%.

Successful investment in broiler and pig improvement could result in an overall increase of 600% in all meat production and 410% in per capita meat availability.

[1] 1 US $ is equivalent to about 830 Rwanda Francs

In the case of cow milk, with the proposed investment interventions the LSA production projection for 2031/32 is 2.2 billion liters of milk which is 1.4 times the BAU production level for the same period. This would increase the per capita availability of milk in Rwanda in 2031/32 to 136 liters per capita or an increase of 115% from 2016/17 and 36% over the BAU situation in 2031/32.

The LSA results show that the future projected increase in milk production could be realized through focusing investment in better genetics, feed and health services to improve family crossbred based dairy farms, as well as commercial-scale specialized dairy production and processing units.

In the case of eggs, with the proposed LSA investment interventions, the projected production would be 1.9 billion eggs in 2031/32 or 2.14 times compared to the BAU case of 869 million. This could lead to per capita production of eggs in 2031/32 reaching to 114 eggs or about 5.7 kg per capita or an increase of 570% from 2016/17 and 211% from the 2031/32 BAU.

In the case of livestock GDP, if the proposed LSA investment interventions are implemented, the results project a livestock GDP of RWF 1.04 trillion ($1.24 billion) in 2031/32 which is an increase of 286% from the baseline year of RWF 268 billion ($321 million) and an increase of 81% from the 2031/32 BAU case of RWF 570 billion.

According to the LSA results, with the investment interventions total meat would contribute 60% of the livestock GDP in which case pork contributes 25%, chicken meat 17%, beef 11%, goats 6.4% and mutton 1%. For meat GDP, pork and chicken meat would become more important than beef. Cow milk would contribute 29% and eggs 7%, and organic matter 4%. This contribution to GDP at the production stage does not include value added from livestock activities across the value chains.

With improvements in animal health services and animal genetics, combined with improved feeding and better management practices, livestock performance improves substantially. In order to increase the production of meat in particular, a broader effort will be required to improve the chicken and pig sector.

Feed supply, in particular forage and fodder for ruminant livestock and grains and oil seeds for chicken and pigs, is foreseen to be the main physical constraint to expanding the livestock production. The huge increase in pig and chicken production would necessitate an allocation of adequate land for the production of maize and soy beans and importation of additional feed ingredients.

In 2014/15 the per capita meat consumption for the people of Rwanda was only 7.9 kg/year for meat, 59 liters/year for milk and 0.63 kg per year for eggs (MINAGRI/RAB 2015). In the LSA (2016/17) baseline the per capita projection for meat was 8 kg, 63 liters of milk and 21

eggs or about 1 kg. These values are far below the FAO nutritionally recommended level of consumption of animal source foods (FAO 1999 in MINAGRI 2004).

Table 4 below shows the incremental production and GDP benefits that could be obtained for each livestock commodity as a result of the LSA additional investment interventions compared to the BAU interventions. With the additional investment recommended in the LSA, pork and chicken meat show a spectacular change in both production and contribution to GDP, followed by eggs. The change in GDP contributions for beef is small and this is due to the higher intermediate costs incurred per unit of change in live weight of crossbreed cattle and the shrinking number of the local cattle breeds.

Table 4**: Percent (%) change in production and GDP between BAU and the "with case", 2031/32**

| | BAU | | "With Case" | |
|---|---|---|---|---|
| **Livestock products** | Production in MT | GDP in RWF | Production (% change) | GDP (% change) |
| Beef | 75,173 | 11,4387 | 24% | 0.3% |
| Goats | 27,699 | 54,639 | 25% | 21% |
| Mutton | 4,161 | 7,263 | 29% | 24% |
| Pork | 47,692 | 50,415 | 568% | 405% |
| Chicken | 35644 | 43,466 | 493% | 311% |
| Total Meat | 190,369 | 270,170 | 248% | 131% |
| Milk | 1,628,347 | 247,580 | 37% | 22% |
| Eggs in number | 869,193,349 | 38,713 | 113% | 77% |
| Hides & skins | 2,259 | 1,078 | 45% | 47% |
| Organic matter | 12,009,806 | 13,359 | 30% | 214% |

The LSA investment interventions result in production of 663 thousand MT of meat, 2.2 billion liters of milk, and 1.9 billion eggs which would substantially increase availability of ASFs in Rwanda to help meet the nutritional requirements of the rapidly growing human population, expected to reach 16.3 million people in 2031/32. The per capita meat production/availability in Rwanda reaches to 41 kg which is eighty-two percent (82%) of the FAO per capita consumption requirement for a healthy life - a very significant improvement! The per capita milk production/availability reaches to 136 liters which exceeds the global average by 51% and that of the average for developing countries by 200%. This means milk and dairy products could be exported from Rwanda. In the case of eggs, the per capita production of eggs reaches to 114 eggs or about 5.7 kg. This exceeds the FAO recommended value and could supply the domestic bakery industry and other industries, or it could be exported after meeting the domestic consumption requirements.

This leads to the following ***strategic policy recommendations***:

Priority given to an increase in productivity or production per animal, by addressing the feed deficit, animal health and genetics. Key policy and investment actions to support

increasing productivity would be: the enhancement of veterinary coverage through private vets and private-public partnerships to reduce mortality and morbidity, the promotion of fodder, and maize and soya bean production through allocation of lands; and the accelerated introduction of improved genetics once feed production and health services are in place.

Through a focus on: increasing production and consumption of low GHG-emitting chickens and pig, dramatically regulating the higher GHG emitters, especially cattle as well as rapid reduction of local breeds of all species, where feasible except goats,  through a higher offtake rate than currently, as well as additional investments to help achieve increased productivity for all livestock species identified as priorities in the LSA, the climate resilience of the sector (Vision 2050) could be improved substantially and the other national development objectives of the nation could still be met.

For the proposed LSA investment interventions to help improve food security for animal source foods (ASFs), an increase in the numbers of livestock will have to occur, but at a lower growth rate. The environmental consequences will need to be closely monitored. More productive crossbreds would also replace the less productive indigenous breeds. For example, with the LSA intervention the proportion of the indigenous cattle in the national cattle herd would drop down to 24% in 2031/32.

Indigenous cattle breeds do not seem to have a future for milk production in a land scare in country like Rwanda, as their yield per cow is too low crossbreeding should continue to be the focus of dairy development.

The key to realizing the great potential of the Rwanda smallholder dairy sub-sector is more investment in milk processing to ensure stable market prices.

Investment in sheep production improvement was not found to have much potential for helping to meet the national development objectives and is not recommended to be a high priority investment option.

Success in modernizing the chicken and pig sub-sector would also require complementary policy intervention: that sufficient land be allocated and put into chicken and pig  feed production (especially maize and soybean), and the private sector be encouraged to invest in chicken and pig agribusinesses **–** especially day old chick production and meat and egg processing and pig meat processing.

If chicken and pork substitute for red meat consumption, then red meat could be exported to meet the GoR export goals to earn more foreign exchange.  However, tastes and preferences for local chicken would have to be changed through promotion of exotic chicken meat and changes in cuisine.

The need for balanced policies to encourage investment in animal production and meat processing to meet domestic per capita meat consumption would also help with export promotion.

Special incentives (to improve the business climate, lower taxes, and increased training) could promote more value addition through processing and product transformation, combined with a clearer role of the public and private sector.

This, in turn leads to the following scenarios that have been tested for their economic feasibility. The agent of change will be a technology intervention; however, they have to be supported by policy adjustments to be fully effective:

Dairy breeding improvement interventions, combining artificial insemination (AI) using higher quality exotic semen with estrus synchronization in all production zones.

In particular for poverty reduction an improvement in the local chickens through selection and reduction of reproductive wastage through provision of brooding facilities and artificial incubators.

Transformation of traditional backyard family chicken (now 75% of the total chicken population) relying on mass introduction of dual purpose crossbred chickens, which have far higher genetic potential for both eggs and meat, when combined with supplemental feeding and adequate health services.

The number and size of specialized commercial scale broiler and layer units also need to be substantially increased. A massive importation and dissemination of exotic chicken breeds by the private sector and through PPPs where the private sector is reluctant to enter on its own.

The number and size of family mixed semi intensive pig system and piglet fattening units also need to be substantially increased. The biosafety (HASP) measure at the production and processing units should be strengthened and killer pig diseases such as ASF should be controlled.

Rapid increase in private and PPP animal health services to provide critical vaccines, and GoR extension services to promote improved feeding and exotic chicken meat and eggs consumption;

Promotion of exports to more remunerative markets through the introduction of a practical and affordable system of animal ID and traceability.

Strengthening animal health infrastructure, quarantine facilities and services

# Dairy value chain Development Roadmap

**(2017/18– 2021/22)**

# Dairy value chain development roadmap (2017/18 – 2021/22)

## *Vision*

Increase milk production enough to meet domestic consumption requirements for dairy products and enable exports of the marketable surplus by increasing dairy cow productivity through improvement in dairy cow genetics, health and nutrition, and by increasing the crossbred dairy cow herd by 46%, milk production by 65%, and contribution to GDP by 53%, while improving the processing and marketing of dairy products.

**Investment scenarios analyzed**

The three dairy investment scenarios analyzed to here are referred to as BAU (Business As Usual), MLI (Medium Level of Investment) and RLI (Recommended Level of Investment). BAU represents the base case scenario with the analysis showing the impacts of continuing the current level of investment and recurrent spending on milk production and contribution to GDP throughout the LMP analysis period of 5 years. RLI is the recommended level of investment which could achieve the agreed upon national development objectives. MLI analyzes the impacts on milk production and GDP of a third option created for investment decision makers who face a budget constraint. It represents a medium level of investment between the BAU and the RLI option.

The RLI scenario is first analyzed and compared to the baseline year (2016/2017) production and contribution to GDP. Then the MLI option is compared to the baseline year (2016/2017) production and GDP contribution. RLI and MLI are then both compared to the BAU scenario impacts (production and GDP contribution) in the 5$^{th}$ year of the LMP period (2021/22) to gauge their differential impacts one to the other.

## *Overall Targets under the recommended level of investment scenario (RLI)*

Currently, there are about 799 thousand crossbreed dairy cattle in Rwanda and it is projected that the number will increase to 1.17 million by 2021/22. This will be a 46% increase (Table 5 below)

In Rwanda, there was about 747 million liters of milk produced in 2016/17 and it is targeted to grow to 1.2 billion liters by 2021/22, a 65% increase over 5 years (Table 6 below)

The current national annualized average milk production per cow is about 909 liters and the target for 2021/22 is to reach at 1,281 liters, 41% increase (Table 7).

Mainly due to the dairy, but also from the red meat improvement interventions, the GDP contribution of milk at nation level is expected to increase from 113 billion RWF in 2016/17 to 163 billion RWF in 2021/22, a 53% increase (Table, 8).

### Specific targets under RLI

The targets for dairy crossbred dairy cattle numbers, milk production, milk production per cow, and contribution to GDP are given for the specific dairy systems by production typology zones in tables 5-8 below.

To facilitate the use of the analytical model created for this LSA study, a typology of the different livestock systems was developed, consisting at the first level in the (a) Low Rainfall Low Altitude (LRLA) zone which predominantly covers the eastern savanna and the eastern plateau of Rwanda with a significant feature of farmland based grazing; (b) the medium rainfall and medium altitude (MRMA) zone which is predominantly found in the central plateau of Rwanda; and (c) the high rainfall high altitude (HRHA) which covers the western highlands, or the Lake Kivu and South West regions. Livestock production practices which are modern, and which are to a large extent independent of location and the production zonation are classified as specialized production (SP) systems and analyzed independently due to their unique modern production characteristics and commercial market orientation.

*Table 5* Current and projected number of crossbreed cattle at national and by production system/zone under RLI

| **Livestock production systems** | **Livestock production zone** | **National and production system crossbred number** | | | | | | **% Change** |
|---|---|---|---|---|---|---|---|---|
| | | **Base year (2016/17)** | **2017/18** | **2018/19** | **2019/20** | **2020/21** | **2021/22** | |
| **Improved Family Dairy (IFD)** | LRLA | 292,822 | 316,248 | 341,548 | 368,872 | 398,382 | 430,252 | 47% |
| | MRMA | 285,171 | 307,985 | 332,624 | 359,234 | 387,972 | 419,010 | 47% |
| | HRHA | 203,932 | 220,247 | 237,867 | 256,896 | 277,448 | 299,643 | 47% |
| | **Sub-total (IFD)** | **781,926** | **844,480** | **912,038** | **985,002** | **1,063,802** | **1,148,906** | **47%** |
| **Commercial Specialized Dairy (CSD)** | Grazing based (Gishwati) | 13,772 | 13,772 | 13,772 | 13,772 | 13,772 | 13,772 | 0% |
| | Non-grazing based (all zones) | 3,199 | 3,313 | 3,431 | 3,552 | 3,679 | 3,809 | 19% |
| | **Sub-total (CSD)** | **16,971** | **17,085** | **17,202** | **17,324** | **17,450** | **17,581** | **4%** |
| **National number of crossbred** | | **798,897** | **861,565** | **929,241** | **1,002,326** | **1,081,252** | **1,166,487** | **46%** |

*Table 6.* Current and projected milk production in Rwanda at national level and by production zone/system

| Livestock production zone | production system | National and production zone/system milk production (000' liters) | | | | | | % Change (five years) |
|---|---|---|---|---|---|---|---|---|
| | | **Base year (2016/17)** | **2017/18** | **2018/19** | **2019/20** | **2020/21** | **2021/22** | |
| | | **Meat-Milk system (Local breeds) and Improved Family Dairy (IFD) (crossbreds)** | | | | | | |
| LRLA | Meat-Milk (Local breeds) | 24,888 | 24,099 | 23,335 | 22,595 | 21,879 | 21,185 | -14.9% |
| | IFD (crossbred) | 227,488 | 253,663 | 282,850 | 315,396 | 351,686 | 392,152 | 72.4% |
| MRMA | Meat-Milk (Local breeds) | 28,183 | 27,263 | 26,373 | 25,512 | 24,680 | 23,874 | -15.3% |
| | IFD (crossbred) | 242,624 | 270,290 | 301,112 | 335,448 | 373,699 | 416,312 | 71.6% |
| HRHA | Meat-Milk (Local breed) | 17,643 | 17,064 | 16,504 | 15,962 | 15,437 | 14,930 | -15.4% |
| | IFD (crossbred) | 183,774 | 204,873 | 228,395 | 254,617 | 283,849 | 316,438 | 72.2% |
| **Sub-total (IFD)** | Meat-Milk (Local breed) | **70,715** | **68,426** | **66,212** | **64,069** | **61,995** | **59,989** | **-15.2%** |
| | IFD (crossbred) | **653,885** | **728,826** | **812,356** | **905,460** | **1,009,234** | **1,124,901** | **72.0%** |
| | | **Commercial Specialized Dairy (CSD)** | | | | | | |
| Gishwati | Grazing based (Gishwati) | 15,721 | 18,031 | 20,681 | 23,719 | 27,204 | 31,202 | 98% |
| All zones | Non-grazing based | 6,267 | 7,365 | 8,654 | 10,170 | 11,950 | 14,042 | 124% |
| **Sub-total (CSD)** | | **21,989** | **25,396** | **29,335** | **33,889** | **39,155** | **45,244** | **106%** |
| **Total milk production** | | **746,589** | **822,648** | **907,903** | **1,003,417** | **1,110,384** | **1,230,135** | **65%** |

Table 7 Annualized milk productivity of cows in Meat-Milk, IFD and CSD systems (liters per year)

| **Livestock production zones** | **Livestock production systems** | **Milk production per reproductive female per year (in liters)** | | | | | | **% Change** |
|---|---|---|---|---|---|---|---|---|
| | | **Base year (2016/17)** | **2017/18** | **2018/19** | **2019/20** | **2020/21** | **2021/22** | |
| | | **Meat-Milk system (Local breed) and Improved Family Dairy (IFD) (crossbred)** | | | | | | |
| LRLA | Meat-Milk (Local breed) | 216 | 218 | 221 | 223 | 226 | 228 | 6% |
| | IFD (crossbred) | 1,323 | 1,369 | 1,417 | 1,467 | 1,519 | 1,572 | 19% |
| MRMA | Meat-Milk (Local breed) | 216 | 218 | 221 | 223 | 226 | 228 | 6% |
| | IFD (crossbred) | 1,418 | 1,468 | 1,519 | 1,572 | 1,627 | 1,684 | 19% |
| HRHA | Meat-Milk (Local breed) | 216 | 218 | 221 | 223 | 226 | 228 | 6% |
| | IFD (crossbred) | 1,512 | 1,565 | 1,620 | 1,677 | 1,735 | 1,796 | 19% |
| **Average** | **Meat-Milk (Local breed)** | **216** | **218** | **221** | **223** | **226** | **228** | **6%** |
| | **IFD (crossbred)** | **1,407** | **1,456** | **1,507** | **1,560** | **1,615** | **1,671** | **19%** |
| | | **Commercial Specialized Dairy (CSD)** | | | | | | |
| Gishwati | Grazing based | 1,890 | 2,191 | 2,539 | 2,943 | 3,411 | 3,954 | 109% |
| All zones | Non-grazing based | 3,360 | 3,772 | 4,235 | 4,754 | 5,337 | 5,991 | 78% |
| **Average** | **CSD** | **2,167** | **2,500** | **2,884** | **3,326** | **3,837** | **4,426** | **104%** |
| **National average** | | **909** | **974** | **1,043** | **1,117** | **1,196** | **1,281** | **41%** |

Table 8 Current and projected GDP contribution of milk in Rwanda at national level and by production zone/system

| **Livestock production zone** | **production system** | **GDP contribution of milk by production zones and systems (10^6)** | | | | | | **% Change** |
|---|---|---|---|---|---|---|---|---|
| | | **Base year (2016/17)** | **2017/18** | **2018/19** | **2019/20** | **2020/21** | **2021/22** | |
| | | **Meat-Milk system (Local breed) and Improved Family Dairy (IFD) (crossbred)** | | | | | | |
| LRLA | Meat-Milk (Local breed) | 3,861 | 3,722 | 3,588 | 3,459 | 3,335 | 3,215 | -17% |
| | IFD (crossbred) | 33,712 | 36,991 | 40,587 | 44,534 | 48,865 | 53616 | 59% |
| MRMA | Meat-Milk (Local breeds) | 4,377 | 4,216 | 4,061 | 3,912 | 3,769 | 3,630 | -17% |
| | IFD | 36,346 | 39,880 | 43,757 | 48,011 | 52,679 | 57800 | 59% |

| Livestock production zone | production system | GDP contribution of milk by production zones and systems (10^6) | | | | | | % Change |
|---|---|---|---|---|---|---|---|---|
| | | Base year (2016/17) | 2017/18 | 2018/19 | 2019/20 | 2020/21 | 2021/22 | |
| | (crossbred) | | | | | | | |
| HRHA | Meat-Milk (Local breeds) | 2,906 | 2,798 | 2,695 | 2,595 | 2,499 | 2,407 | -17% |
| | IFD (crossbreds) | 29,288 | 32,197 | 35,394 | 38,909 | 42,772 | 47020 | 61% |
| **Sub-total (IFD)** | Meat-Milk (Local breeds) | **11,143** | **10,737** | **10,344** | **9,967** | **9,603** | **9,252** | -17% |
| | IFD (crossbreds) | **99,347** | **109,067** | **119,738** | **131,454** | **144,316** | **158,436** | 59% |
| | | **Commercial Specialized Dairy (CSD)** | | | | | | |
| Gishwati | Grazing based (Gishwati) | 2,343 | 2,343 | 2,343 | 2,343 | 2,343 | 4,086 | 74% |
| All zones | Non-grazing based | 576 | 576 | 576 | 576 | 576 | 1,592 | 176% |
| **Sub-total (CSD)** | | **2,919** | **2,919** | **2,919** | **2,919** | **2,919** | **5,678** | 95% |
| **Total GDP from milk** | | **113,409** | **122,723** | **133,002** | **144,340** | **156,838** | **173,367** | 53% |

Milk in Rwanda is produced from two species; cattle and goats. The milk coming from goats is so small it is not considered in this LMP report. This section analyzes the impacts of dairy improvement interventions on milk produced from cattle only. The milk is coming from cattle produced from two production systems; the Cow dairy system, which mainly uses crossbred cattle and the Meat-Milk System which is dominated by the local breeds. The Cow dairy production system can be further divided into two systems, Improved family dairy (IFD) and commercial specialized dairy (CSD). The IFD production system is practiced by farmers in mixed crop-livestock production systems, uses crossbred cattle with small level of inputs and results in a moderate level of milk production. The CSD system, on the other hand, is a commercial scale specialized dairy production system. It includes the specialized grazing based dairy production that is mainly practiced in the Gishwati Rangeland and non-grazing based dairy production that is practiced mainly around urban and per-urban areas.

The two Cow dairy systems (IFD and CSD) share a lot of common challenges, opportunities and improvement and investment options; however, to capture the distinct features of each system and make implementation of the plan easier, each system is discussed separately in terms of challenges, opportunities, improvement and investment options and impact of interventions.

## *Improved Family Dairy (IFD) under RLI*

### Targets

The number of crossbreed dairy cattle in the IFD system will increase from the current 781 thousand to 1.15 million in 2021/22. The growth rate in number of crossbreds will be the same between different production zones (Table 5).

The production of milk from the IFD system will increase from 653 million liters in the base year to 1.12 billion liters in 2021/22, a 72% increase from the base year (Table 6).

Annual milk production of a cow in the IFD system will increase from 1,407 lt/year in the base year to 1,671 lt/year in 2021/22, a 19% increase in productivity (Table 7).

Due to the dairy improvement interventions in the IFD system, the GDP contribution of milk from the system is expected to increase from 99 billion RWF in 2016/17 to 158 billion RWF in 2021/22, increase in 59% (Table 8).

Table 9: Key challenges and strategies related to IFD:

| | **Key challenges** | **Strategies** |
|---|---|---|
| **1.** | **Feed availability and quality** | |
| | Scarcity and low quality of feed stuff (forage, concentrate)<br>Limited supplementation<br>Inadequate use of available feed resources<br>Limited marketing and high cost of forage feed<br>Shortage of water | Strengthening the extension service and training on forage production, conservation and feeding<br>Improve the use of available feed resources (crop residues and all other resources)<br>Enforcing feed, forage and forage seed quality standards<br>Rainwater conservation |
| **2.** | **Low genetic potential of indigenous animals for milk production** | |
| | Inadequate and inefficient AI services.<br>Local breed low genetic potential for milk production<br>Exotic breeds have low adaptability to disease and harsh management conditions | Promoting, expanding, and strengthening privatization of AI, and AI with synchronization services;<br>Providing training, backup support and incentives to livestock farmers to work as AI technicians.<br>Establishing and strengthening dairy heifer multiplication farms through private, public and private-public joint ventures;<br>Encourage cross-breeding with improved dairy breeds<br>Improve extrinsic factors to enhance adaptability of exotic breeds<br>Put in place herd record scheme |

| | Key challenges | Strategies |
|---|---|---|
| | | |
| **3.** | **Animal health services** | |
| | High prevalence of TBD and other parasites (Tsetse Fly, helminthes), mastitis and brucellosis<br>Incidence of Transboundary diseases,<br>High calf mortality;<br>Inefficient animal health services;<br>Poor quality control of drugs and supplies;<br>Inadequate of professionalism in farming (hobby farming, low knowledge, low access to capital investment) | Reinforcement of diseases control interventions<br>Enhance disease surveillance (empower veterinary laboratories)<br>Enhance and encourage private animal health service delivery<br>Improving availability and quality control of vaccines and drugs<br>Harmonization of animal health management in the countries within the region |
| **4.** | **Marketing and processing** | |
| | Fluctuations in milk supply due to seasonality (dry and wet seasons)<br>Unreliable transport system<br>Insufficient number of milk processing plants<br>An absence of quality-based pricing incentives;<br>Poor milk quality control and enforcement mechanisms<br>Existing informal trade of raw milk which pose threat to zoonosis; and<br>Limited promotion of dairy-product consumption;<br>Lack of grading & pricing schemes of animal products<br>Informal milk cross border trade | Promote investment in milk processing<br>Put in place animal products grading and pricing policies<br>Introduction of quality-based standards and pricing to encourage quality milk supply<br>Strengthen enforcement of milk and milk products quality standards<br>Formalize milk trade by training and licensing of milk traders<br>Upscale the on-going school milk feeding programs to promote milk consumption<br>Create and promote awareness of milk consumption<br>Invest in Feeder roads<br>Put in place a framework for the enforcement of all existing regulations (eg; ministerial order on milk) |
| **5.** | **Policy** | |
| | Pricing policies causing disincentives to involve in milk processing industries | Put in place indicative prices for milk products<br>Reduce the bureaucracy and facilitate investment in the dairy industry |

## *Interventions to achieve targets*

**I. Intervention area/s (production zone/s):**

Dairy improvement intervention can be practiced in all over the country and all livestock production zones (low, medium and high rainfall zones)

**II. Priority interventions**

**Feed and feeding**

Promote improved grass and leguminous feed productions in all accessible areas including backyard, hedge and live fence. Feeds like Pennisetum, Brachiaria, Leucaena, Sesbana, Calliandra, Chloris gayana, Panicum Maximum, desmodium sp, etc.). Adoption rate and coverage of this technology expected to reach 50% and 100%, respectively, in the coming 5 years (González et.al, 2016[2]).

Improve production, marketing and quality control of forage and forage seeds through strengthening existing regulatory bodies

Improve quality control of processed feeds and industrial by-products used as feed in the dairy system

Increase the use of crop residues e.g.: maize drying on the field to be used and conserved for animal feed

Increasing access to water **–** prioritizing water shortage areas

Promote allocation of land for production of improved pasture/forage

Increase the efficient use of forages and crop residues (e.g. conservation)

Create an industry for producing feed additives (minerals, vitamins, amino acids)

Increase availability of cereals (e.g. importation)

Promote the use of concentrates/processed feeds from feed processing plants

Mobilize farmers associations to organize group purchase and distribution of processed feed

Research on:

Successful forages species according to zones

The real quantity of forages and post-harvest that Rwanda can produce in comparison to actual and projected demands

**Health**

Coverage of health service will reach 100%

Farmers that adopt the recommended rate of tick control treatments will reach 60% in the coming 5 years

All animals that need vaccination against endemic (ECF, BQ, brucellosis, Rabies), epidemic diseases (FMD, LSD, RVF) will receive vaccines.

Enforce FMD and CBPP free Rwanda in 5 years to improve the acceptance of products from importing countries

Rate (%) of animal/animal product movement permits issued will be 100%

---

[2] González C; Schiek B; Mwendia S; Prager SD. 2016. Improved forages and milk production in East Africa. A case study in the series: Economic foresight for understanding the role of investments in agriculture for the global food system. Cali, CO: Centro Internacional de Agricultura Tropical (CIAT). 48 p.

Strengthen capacity for disease surveillance, quarantines, and supervise mass vaccination programs

Strengthen veterinary diagnosis laboratories

Promote private vet service providers

Farmers that adopt mastitis control and management technologies will reach 60% in 5 years. Currently 60% of cattle have mastitis cases.

**Extension**

Intensive diary improvement trainings will reach 60% of dairy cattle owners in 5 years. Farmers will receive more intensive training in relation to dairy cattle management (feeding, breeding, deworming, tick control, hygiene and milk handling and transport)

Percent of MCCs and cooperatives providing dairy input supply, animal health, extension and financial services increases from about the current 38%3 to 80% in 5 years

Build the capacity of extension agents

Increase extension services delivery through farmer associations

**Genetics**

The population of local breed cattle will decrease while the number of crossbred and the total number of cattle increases. The growth rate of cattle number should slow down and eventually become zero percent depending on the available feed (the assumptions for future cattle population growth is indicated in Annex).

The number of local breed decrease by an annual growth rate of about -4%. The average growth rate of local breed cattle trend for the past 10 years was about -4% (RAB, report).

The number of crossbreed cattle increase by annual growth rate of up to 8% in the first five years (2016/17 – 2021/22). The average growth rate trend of crossbreed cattle for the last 10 years is about 16% (RAB, report).

The overall cattle population will be growing with only 2.7% the first five years and slows after that. The current cattle population growth rate is around 3% (RAB, report)

Improve the efficiency of AI service through promoting private AI practitioners and training of AI technicians

Extend the use of AI. Currently only up to 15% of cows can get the service4.

Continuous research on exotic breeds and their adaptability to the production systems

Expand performance and pedigree records

**Marketing and processing**[5]

The priority interventions related to marketing and processing of milk include:

[3] IFAD, 2016. Rwanda Dairy Development Project, detailed design report.

[4] Both insemination and semen production targets for 2016/17 shows that (RAB, 2016/17), only 15% of cows can be inseminated annually. There is a plan to inseminate 118,000 cows and produce 160,000 dose of semen by 2016/17. With 55% ratio for reproductive females (about 765,094 cows). The number of cows that would be inseminated with AI service would be 118,000/765,094 = 15%

[5] The December 2015 Ministerial Order (MO) regulating the collection, transportation and selling of milk, requires all milk sold in the country must first be collected at a place where its quality testing is possible. The law recognizes two types of milk collection points: simple sheltered milk collection sites and modern milk collection centers.

- Increase the functional capacity and utilization of existing MCCs6.
- About 150 additional MCCs are required at 90% utilization of capacity with milk collection capacity of 5,000 lts/day to collect the total milk currently sold in the country.
    - o 150 MCCs and 200 milk collection sites will be established in the five years.
- Strengthen existing dairy cooperatives and establish new 150 cooperatives
- Enhance the capacity of existing MCCs to test antibiotic residues, mastitis and start to fully enforce existing milk quality standards at MCCs in the coming 5 years
- Establish functional linkage between private milk traders, MCCs, cooperatives and processing plants as observed in Gicimbu district, Kageyo dairy cooperative
- Improve grading and quality based pricing of milk and milk products
- Percent of milk collected in the formal market to increase from the current 10-15% to 80% in 5 years
- Attract local and international investors in milk cold chain and milk processing
- Improve feeder roads to and from MCCs/MCPs
- Encourage establishment of new milk processing plants (pasteurized milk, UHT and powdered milk) that can process at least a sum of 675 MT/day. Currently Rwanda has a capacity of processing 280 MT/day7. With the assumption that most of the milk collected by MCCs will go to processing plants, milk processing capacity of additional 675 MT/day is required in the coming 5 years.

## Assumptions and targets in the IFD system resulting from dairy VC improvement interventions under RLI

### I. Improved Family Dairy (IFD) production

Assumptions for the IFD system: the following assumptions hold for those who adopt the dairy improvement interventions:

- Parturition rate to increase by 10% (Argent, et.al. 20148)
- Mortality rate to decrease by 20% to 40%
- The growth rate for the number of crossbred cattle will be about positive 8% and local breeds negative 4% (due to the feed constraint, higher productivity and higher profitability of crossbred cows)

---

[6] Under current situation, about 30% of the milk is consumed at home while rest is sold and only 10-15% goes to the formal market. However, based on the new MO, up to 70% of the milk i.e. around 500,000 MT (709,399,650*70%) need to pass through milk collection sites/centers. In Rwanda, there are 100 MCCs (28 fully functional, 58 not fully functional and 14 not functional) (IFAD, 2016). Though more than 60% of MCCs work under capacity of the threshold of 2,000 lt/day, the total installed capacity of existing MCCs is around 200 - 300 MT/day (IFAD, 2016).

[7] The country has five main milk processing plants and about 25-30 small and medium scale processors of cheese and other dairy products (IFAD, 2016). In total, Rwanda is currently estimated to have about 280 MT/day milk processing capacity (around 84,000 MR/year), with utilization of around 35-40% of the total installed capacity. The five main processing plants include Inyange Industries, Crystal Industries (only 7 months old with a production capacity of 100 MT per day but currently operating at 10% capacity but rapidly expanding) Nyanza Dairy, Savanah Dairy, Haji Dairy and Blessed Dairies

[8] Argent J., Augsburg B., Raul I. 2014. Livestock asset transfers with and without training: Evidence from Rwanda. Journal of Economic Behavior & Organization 108. PP 19–39.

Lactation length to increase from 270 to 300 days
Daily milk production to increase by 30% (González et.al, 2016[9], Argent, et.al. 2014)
Expense for purchase of forage seeds and forage cuttings increases by 20%
Amount of purchased concentrate/processed feed to increase by at least 2-3 kg/day/lactating cow. At least 50% of crossbred cow owners in the country are assumed to feed 2-3 kg/day/cow by the 5th year (2021/22) of the LMP
Expenditure for veterinary services will double (increase 50%)

## IFD investments under RLI

The investment costs of dairy improvement are categorized into six major intervention areas – feed, genetics and breeding, health, extension, research and milk marketing and value addition. The investment cost for milk marketing and value addition takes the highest share of the total investment (about 40 billion RWF, 80%) and 71% will be funded by the private sector (Table 11). Genetics and breeding takes the second highest share of the total investment cost followed by research and feed improvement investment costs. The total dairy cattle improvement investment costs for the LMP 5-year period (2017/18 – 2021/22) add up to RWF 50.5 billion; out of which 58% is expected to come from the private sector, while the rest (42%) is expected to be **funded by the public sector (Gov't, NGOs, etc.**) (Table 11).

[9] González C; Schiek B; Mwendia S; Prager SD. 2016. Improved forages and milk production in East Africa. A case study in the series: Economic foresight for understanding the role of investments in agriculture for the global food system. Cali, CO: Centro Internacional de Agricultura Tropical (CIAT). 48 p.

*Table 10.* Five year dairy improvement investment capital costs (2017/18 **–** 2021/22)

| No | Investment Intervention | Unit | Quantity | Unite cost (10^6 RWF) | Investment cost (10^6 RWF) | | | | | | Budget source |
|---|---|---|---|---|---|---|---|---|---|---|---|
| | | | | | 2017/18 | 2018/19 | 2019/20 | 2020/21 | 2021/22 | Total | |
| **1** | **Animal Feeding** | | | | | | | | | | |
| | Support to forage seed multipliers (initial financial support and small equipment) | Ha | 0.7 | 0.02 | | 7.5 | | 7.5 | 3.7 | **18.7** | Public - 30%, private - 70% |
| | Support small scale feed processing units (initial investment) | Processing units | 25 | 2.49 | 24.9 | | 24.9 | | 12.5 | **62.3** | Public - 30%, private - 70% |
| | Construction of 3 Commercial animal feed plants | Feed plants | 3 | | | | | | | | This cost is covered in the chicken VC roadmap as 100% private |
| | Improve the capacity of existing feed quality control laboratories | laboratories | 1 | 664.00 | | 664.0 | | | | **664.0** | Public - 50%, private - 50% |
| | Strengthen the existing pasture/forage seed quality control laboratories | laboratories | 1 | 664.00 | 664.0 | | | | | **664.0** | Public - 50%, private - 50% |
| | Rangeland rehabilitation -- removing weeds, | ha | 1,000 | 0.04 | 13.2 | | 16.6 | | 11.6 | **41.6** | Public - 20%, private - |

| No | Investment Intervention | Unit | Quantity | Unite cost (10^6 RWF) | Investment cost (10^6 RWF) | | | | | | Budget source |
|---|---|---|---|---|---|---|---|---|---|---|---|
| | | | | | 2017/18 | 2018/19 | 2019/20 | 2020/21 | 2021/22 | Total | |
| | over-sowing, fertilizer application in Gishwati rangeland | | | | | | | | | | 80% |
| | **Sub-total** | | | | **695.5** | **678.1** | **33.2** | **15.8** | **22.0** | **1435.5** | |
| **2** | **Animal Health** | | | | | | | | | | |
| | Equipment/ kit for vet technicians | Vet technician | 175 | 1.00 | 41.5 | 41.5 | 49.8 | 41.5 | | **174.3** | Public – 100% |
| | Rehabilitation and equipping satellite laboratories | laboratory | 2 | 14.11 | 14.1 | 14.1 | | | | **28.2** | Public – 100% |
| | Equip the national veterinary laboratory | laboratory | 1 | 14.94 | 14.9 | | | | | **14.9** | Public – 100% |
| | Motorcycles for district DVMs and sector vets | Motorcycle | 300 | 1.25 | | | | | | **373.5** | Public – 100% |
| | **Revision of AHW's** training manual | Manual | 1 | 8.30 | 8.3 | | | | | **8.3** | Public – 100% |
| | Support private AHWs (kits including bicycle and cell phone) | AHWs | 70 | 1.00 | | | 33.2 | 19.9 | 16.6 | **69.7** | Public - 30%, private - 70% |
| | Support private veterinary doctors (kits) | Vet doctors | 9 | 1.16 | | 5.8 | 4.6 | | | **10.5** | Public - 30%, private - 70% |
| | **Sub-total** | | | | **153.6** | **144.4** | **87.6** | **194.2** | **99.6** | **679.4** | |
| **3** | **Animal Breeding and Genetics Investments** | | | | | | | | | | |
| | Purchase of cattle and accompanied | Heifers | 5,000 | 0.76 | 830.0 | 830.0 | 830.0 | 830.0 | 830.0 | **3818.0** | Public – 100% |

| No | Investment Intervention | Unit | Quantity | Unite cost (10^6 RWF) | Investment cost (10^6 RWF) | | | | | | Budget source |
|---|---|---|---|---|---|---|---|---|---|---|---|
| | | | | | 2017/18 | 2018/19 | 2019/20 | 2020/21 | 2021/22 | Total | |
| | packages in GRINKA program | | | | | | | | | | |
| | Rehabilitation of Masaka bull station and upgrade of lab equipment | Station | 1 | 182.60 | 91.3 | 91.3 | | | | **182.6** | Public **–** 100% |
| | Purchase of high genetic merit bulls | Bulls | 10 | 16.60 | 49.8 | 49.8 | | 33.2 | 33.2 | **166.0** | Public **–** 100% |
| | Production and diffusion of breeding plan | Document | 1 | 12.45 | 7.5 | 5.0 | | | | **12.5** | Public **–** 100% |
| | Nitrogen Plant Equipment installation | Nitro. plant | 1 | 415.00 | 415.0 | | | | | **415.0** | Public **–** 100% |
| | Support private AI service providers (kits including cell phone) | Technicians | 200 | 1.16 | | | 10.0 | 8.3 | 5.0 | **232.4** | Public - 30%, private - 70% |
| | Animal identification and performance recording (software, equipment and training) | Lump sum | 1 | 334.74 | 46.1 | 98.4 | 63.7 | 64.9 | 61.7 | **334.7** | Public **–** 100% |
| | **Sub-total** | | | | **1439.7** | **1074.4** | **903.6** | **936.4** | **929.8** | **5,161.2** | |
| **4** | **Extension** | | | | | | | | | | |
| | Prepare, publish and distribute dairy cattle management training manuals | Training manual | 1 | 37.35 | 20.8 | 16.6 | | | | **37.4** | Public **–** 100% |
| | Training of national dairy master trainers | Trainee | 10 | 24.90 | 249.0 | | | | | **249.0** | Public **–** 100% |

| No | Investment Intervention | Unit | Quantity | Unite cost (10^6 RWF) | Investment cost (10^6 RWF) | | | | | | Budget source |
|---|---|---|---|---|---|---|---|---|---|---|---|
| | | | | | 2017/18 | 2018/19 | 2019/20 | 2020/21 | 2021/22 | Total | |
| | (in country and study tour abroad) | | | | | | | | | | |
| | **Sub-total** | | | | **269.8** | **16.6** | **0.0** | **0.0** | **0.0** | **286.4** | |
| **5** | **Research** | | | | | | | | | | |
| | Infrastructure of livestock research centers and farm stations rehabilitated (infrastructure and lab equipment) | Research centers | 8 | 373.50 | 373.5 | 830.0 | | 954.5 | 830.0 | **2988.0** | Public **–** 100% |
| | **Sub-total** | | | | **373.5** | **830.0** | **0.0** | **954.5** | **830.0** | **2,988.0** | |
| **6** | **Marketing and Value Addition** | | | | | | | | | | |
| | Strengthen the capacity of milk quality and safety control laboratory | laboratories | 1 | 332.00 | 249.0 | 83.0 | | | | **332.0** | Public **–** 100% |
| | Strengthen existing dairy cooperatives | Cooperatives | 50 | 12.45 | 311.3 | 311.3 | | | | **622.5** | Public - 70%, private - 30% |
| | Establish new and re-establish non-functional dairy cooperatives (training, sensitization, equipping and facilities) | Cooperatives | 150 | 66.40 | | 2490.0 | 2490.0 | 2490.0 | 2490.0 | **9960.0** | Public - 70%, private - 30% |
| | Strengthen the capacity of | Cooperatives | 100 | 20.75 | | 518.8 | 518.8 | 518.8 | 518.8 | **2075.0** | Public - 70%, |

| No | Investment Intervention | Unit | Quantity | Unite cost (10^6 RWF) | Investment cost (10^6 RWF) | | | | | | Budget source |
|---|---|---|---|---|---|---|---|---|---|---|---|
| | | | | | 2017/18 | 2018/19 | 2019/20 | 2020/21 | 2021/22 | Total | |
| | cooperatives to enable them give services like milk collection, extension, health, AI, financial | | | | | | | | | | private - 30% |
| | Organize and capacity building of milk collectors (kits including bicycle) | Cooperatives | 1,000 | 1.25 | 249.0 | 249.0 | 249.0 | 249.0 | 249.0 | **1245.0** | Public - 30%, private - 70% |
| | Organize milk traders | Milk traders | 5 | 207.50 | 207.5 | 207.5 | 207.5 | 207.5 | 207.5 | **1037.5** | Private **–** 100% |
| | Strengthen existing MCCs | MCCs | 50 | 41.50 | 1660.0 | 415.0 | | | | **2075.0** | Public - 70%, private - 30% |
| | Establish new milk collection points | Milk collection points | 200 | 2.70 | | 134.9 | 134.9 | 134.9 | 134.9 | **539.5** | Public - 70%, private - 30% |
| | Establish new MCCs | MCCs | 150 | 2.49 | | 83.0 | 83.0 | 103.8 | 103.8 | **373.5** | Public - 70%, private - 30% |
| | Strengthen the capacity of National Dairy Farmers Federation of Rwanda (NDFFR) | Lump sum | 1 | 83.00 | | 41.5 | | 41.5 | | **83.0** | Public - 40%, private - 60% |
| | **Sub-total** | | | | **5,608.8** | **7,277.4** | **15,497.6** | **5,846.3** | **16,375.3** | **50,473.5** | |
| | **GRAND TOTAL INVESTMENT** | | | | **5608.8** | **7270.8** | **15497.6** | **5838.0** | **16375.3** | **50,467.7** | |

Table 11. Total investment and share of dairy improvement investment costs from public and private sources

| No | Investment Intervention | Investment cost (10^6 RWF) | | Investment cost share (%) | |
|---|---|---|---|---|---|
| | | Public | Private | Public | Private |
| 1 | Animal feeding | 696.62 | 753.98 | 48% | 52% |
| 2 | Animal health | 623.3 | 56.1 | 92% | 8% |
| 3 | Animal breeding and genetics | 4,998.5 | 162.7 | 97% | 3% |
| 4 | Extension | 286.4 | - | 100% | 0% |
| 5 | Research | 2,988.0 | - | 100% | 0% |
| 6 | Marketing and value addition | 11,690.6 | 28,232.5 | 29% | 71% |
| | GRAND TOTAL INVESTMENT | 21,279.26 | 29,188.64 | 42% | 58% |

## Impacts of dairy improvement interventions in the improved family dairy (IFD) system under RLI

### Milk production

Due to the dairy improvement interventions, the production of milk in the IFD is expected to increase from 653 million to 1.12 billion liters over five years (2016/17 - 2021/22), an increase in 72% (Table, 6).

### Change in milk productivity per cow

Annual milk production of a cow in the IFD system will increase from about 1,407 liters/year in the base year to 1,671 liters/year in 2021/22, a 19% increase in productivity (Table 7).

### GDP contribution

The GDP contribution of milk from the IFD system increases from 99 billion in the base year (2016/17) to 158 billion RWF in the 5$^{th}$ LMP year or 2021/22. The percent change in GDP contribution between 2016/17 and 2021/22 for IFD systems is more or less the same (59-61%) for all the three production zones for the dairy VC, as one would expect (Table 8).

Table 12: Activities time line and sequencing: Gantt chart under RLI

| No | Investment Intervention | Investment cost (10^6 RWF) | | | | |
|---|---|---|---|---|---|---|
| | | 2017/18 | 2018/19 | 2019/20 | 2020/21 | 2021/22 |
| **1** | **Animal Feeding** | | | | | |
| | Support to forage seed multipliers (initial financial support and small equipment) | | | | | |
| | Support small scale feed processing units (initial investment) | | | | | |
| | Construction of 3 Commercial animal feed plants | | | | | |
| | Improve the capacity of existing feed quality control laboratories | | | | | |
| | Strengthen the existing pasture/forage seed quality control laboratories | | | | | |
| | Range land rehabilitation and removing weeds, over-sowing, fertilizer application in Gishwati rangeland | | | | | |
| **2** | **Animal Health** | | | | | |
| | Equipment/ kit for vet technicians | | | | | |
| | Rehabilitation and equipping satellite laboratories | | | | | |
| | Equip the national veterinary laboratory | | | | | |
| | Motorcycles for district DVMs and sector vets | | | | | |
| | **Revision of AHW's training manual** | | | | | |
| | Support private AHWs (kits including bicycle and cell phone) | | | | | |
| | Support private veterinary doctors (kits) | | | | | |
| **3** | **Animal Breeding and Genetics Investments** | | | | | |
| | Purchase of cattle and accompanied packages in GRINKA program | | | | | |
| | Rehabilitation of Masaka bull station and upgrade of lab equipment | | | | | |
| | Purchase of high genetic merit bulls | | | | | |
| | Production and diffusion of breeding plan | | | | | |
| | Nitrogen Plant Equipment installation | | | | | |
| | Support private AI service providers (kits including cell phone) | | | | | |
| | Animal identification and performance recording (software, equipment and training) | | | | | |
| **4** | **Extension** | | | | | |
| | Prepare, publish and distribute dairy cattle management training manuals | | | | | |
| | Training of national dairy master trainers (in country and study tour abroad) | | | | | |
| **5** | **Research** | | | | | |
| | Infrastructure of livestock research centers and farm stations rehabilitated (infrastructure and lab equipment) | | | | | |
| **6** | **Marketing and Value Addition** | | | | | |

| No | Investment Intervention | Investment cost (10^6 RWF) | | | | |
|---|---|---|---|---|---|---|
| | | 2017/18 | 2018/19 | 2019/20 | 2020/21 | 2021/22 |
| | Strengthen the capacity of milk quality and safety control laboratory | | | | | |
| | Strengthen existing dairy cooperatives | | | | | |
| | Establish new and re-establish non-functional dairy cooperatives (training, sensitization, equipping and facilities) | | | | | |
| | Strengthen the capacity of cooperatives to enable them give services like milk collection, extension, health, AI, financial | | | | | |
| | Organize and capacity building of milk collectors (kits including bicycle) | | | | | |
| | Organize milk traders | | | | | |
| | Strengthen existing MCCs | | | | | |
| | Establish new milk collection points | | | | | |
| | Establish new MCCs | | | | | |
| | Strengthen the capacity of National Dairy Farmers Federation of Rwanda (NDFFR) | | | | | |
| | Construction of 2 milk processing plants | | | | | |
| | **Continue and expand "One cup of milk per child" program** | | | | | |

**Complementary intervention and success requirements**

The following are crucial aspects of the dairy improvement interventions and success requirements

- Improving the use of the capacity of the Milk Collecting Centers (MCCs) and dairy plants
- Infrastructure development (feeder roads, water, electricity, crushes, etc.)
- Establishment of community kraals (igik**umba cy'umudugudu), and the support and** follow up given to the GIRINKA program
- Strengthening animal feed factories and distribution selling points across the country
- Putting in place grading and pricing systems for animal products
- Increase access to finance/loans through rural banks and credit associations
- Making livestock insurance available
- Continued use of improved technologies
- Herd book development - introduction of the farm data recording policy
- Empowering and supporting the Rwanda Council of Veterinary Doctors (RCVD)
- Increasing the number of public and private extension workers
- Planning and carrying out an extensive crossbreeding/breeding scheme using AI, AI with synchronizing hormone and/or bull service with quality dairy bull breeds
- Improving the efficiency of AI, AI with synchronizing hormone and/or bull crossbreeding/breeding services
- Encouraging private health, extension and AI service providers
- Enforcing forage, concentrate feed and forage seed quality standards
- Creating a conducive environment for production and marketing of feeds and feed seeds
- Enforcing milk quality standards and supporting full capacity functioning of milk processing plants.

## *Commercial Specialized Dairy (CSD) Production under RLI*

### CSD Targets under RLI

- The number of crossbreed dairy cattle in the CSD will increase from the current number of about 17 thousand to about 18 thousand in 2021/22. The growth rate of crossbred cattle number in the grazing based (Gishwati) CSD will be zero as the grazing land already has reached the carrying capacity established by the GoR. In the case of non-grazing based CSD, the number of crossbred cattle is expected to grow by about 19% (Table 5).
- The production of milk from the CSD system will increase from 22 million liters in the base year (2016/17) to 45 million liters in 2021/22, a 106% increase from the base year (Table 6). More change in milk production is expected in the non-grazing based CSD (124%) than grazing based (Gishwati) CSD (98%).
- Annual average milk production of a cow in the CSD system will increase from around 2,200 lt/year in the base year to about 4,400 lt/year in 2021/22, a 104% increase in productivity (Table 7). Grazing based (Gishwati) CSD is expected to show more increase

(109%) than non-grazing based CSD (78%). This is due to the lower initial level of productivity of the grazing based (Gishwati) CSD dairy cows.

Due to the dairy improvement interventions in the CSD system, the GDP contribution of milk from the CSD is expected to increase from about 3 billion RWF in 2016/17 to 5.7 billion RWF in 2021/22, increase in 95% (Table 8). Similar to the milk production, this increase in GDP contribution of non-grazing based CSD comes from both increasing the cattle number and productivity increase; as opposed to the grazing based (Gishwati) CSD, in which case the increase in GDP contribution comes only from the change in productivity.

## Key challenges and strategies related to CSD under RLI

Many of the challenges and strategies listed in the IFD section are also pertinent for commercial specialized dairy (CSD). Thus, only specific challenges and strategies which are important to CSD are listed here.

Table 13: Key challenges and strategies related to CSD under RLI

| | **Key challenges** | **Strategies** |
|---|---|---|
| **1.** | **Feed availability and quality** | |
| | Lack of commercial scale forage production<br>Shortage of concentrate feed and roughage (both in quality and quantity)<br>Lack of effective feed quality control and standards enforcement mechanisms | Making land available for commercial forage production by investors;<br>Enforcing feed quality standards, quality monitoring and control;<br>Promoting the establishment of flour mills and oil processing plants which will make more concentrate feed ingredients available i.e. wheat bran, wheat short and seed cakes; |
| **2.** | **Marketing and processing** | |
| | Shortage of dairy technologists.<br>Inadequate number of MCCs and under performance of existing ones | Building the capacity of the dairy technology training institute (s).<br>Improving the efficiency of existing and establishing new MCCs and co-operatives |
| **3.** | **Policy and investment support** | |
| | Poor milk quality control and enforcement<br>Limited number of commercial specialized dairy farms and milk processing plants; | Enforcing milk-quality standards as well as grading and pricing policies;<br>Enforcing an effective land acquisition policy for dairy investments (preferential treatment for accessing land for specialized dairy production, milk processing and feed production);<br>Providing incentives for investors to establish specialized dairy farms and dairy processing plants: |

## Interventions to achieve CSD targets under RLI

The major interventions proposed to improve the CSD system are feed improvement (production, marketing and processing of more and better quality feed), increase in the number of crossbred dairy cattle and CSD farms, supporting private AI and health service providers to increase services to CSD farmers, and improved marketing of milk and milk products. Most of the interventions proposed in the IFD section will also work for the CSD system. The interventions listed below are assumed to be more related to the CSD system, though they also work for the IFD system.

- Improve the productivity of grazing land in Gishwati by over sowing with improved grass and leguminous forage seeds used and use of fertilizer where applicable
- Improve production, marketing and quality control of forage and forage seeds through strengthening existing regulatory bodies
- Improve quality control of processed feeds and industrial by-products used as feed in the dairy system
- Promote allocation of land for production of improved pasture/forage
- Create an industry for producing feed additives (minerals, vitamins, amino-acids)
- Increase availability of cereals (e.g. importation)
- Promote the use of concentrates/processed feeds from feed processing plants
- Promote the use of recommended rate of external and internal parasite treatments
- Ensure that all animals that need vaccination against endemic (ECF, BQ, brucellosis, Rabies,), epidemic diseases (FMD, LSD, RVF) receive vaccines
- Promote private vet service providers
- Farmers and dairy operators receive intensive dairy cattle management trainings (feeding, breeding, deworming, tick control, hygiene and milk handling and transport)
- Improve the efficiency of AI service through promoting private AI practitioners and training of AI technicians
- Expand performance and pedigree records

Interventions proposed to improve marketing and processing under the IFD section will also be equally important to the CSD system growth and development.

## Assumptions and Targets for the CSD systems under RLI

### I. Grazing based commercial dairy (Gishwati rangeland milk shed)

Assumptions[10] hold for those adopting the dairy improvement interventions in the grazing based commercial dairy (Gishwati rangeland milkshed):

- Parturition rate to increase to 85% (0.85)
- Mortality to decrease by 25% to 50%
- The number of cattle per herd (farm) assumed to remain the same (zero growth rate (0%))
- Lactation length to extend from 270 to 300 days
- Daily milk production increases from the current average 10 lt/day to 18 lt/day

[10] The assumptions below are mainly dependent on the ability of the milk shed to collect the milk produced in the Gishwati rangeland. Here, it is assumed that all the infrastructure (road, MCCs …) will be in place to increase marketing and collection of milk from the milk shade to 80% in five years from the current very low level.

Amount of purchased concentrate/processed feed to increase by at least 4 kg/day per lactating cow.
Expenditure on veterinary services and forage seed purchase will double

**II. Non-grazing based commercial dairy (Urban and per-urban CSD)**

The following assumptions hold for those who adopt the interventions in the Commercial non-grazing based dairy.

Parturition rate increases to 90%
Mortality decreases by 10% to 20%
The growth rate for the number of crossbred cattle will be about 4%
Lactation length to increase from 280 to 300 days
Daily milk production to increase from the current average 15 lt/day to 25 lt/day
Amount of purchased concentrate/processed feed to increases by at least 5 kg/day/lactating cow
Expenditure on veterinary services will increase by 50%

## Investments in CSD under RLI

The investments listed under the IFD, above, work equally for CSD and the investment will be shared among both IFD and CSD systems

## Impacts of dairy improvement intervention on CSD

**Milk production**

Due to the dairy improvement interventions in CSD, the production of milk in the system is expected to increase from about 22 million liters to 45 million liters over five years (2016-2020), an increase in 106% (Table, 6).

**Change in milk productivity per cow**

Annual milk production of a cow in the CSD system will increase from about 2,200 lt./year in the base year to about 4,400 lt./year in 2021/22, a 104% increase in productivity (Table 7).

**Contribution to GDP**

Dairy improvement intervention in the CSD increases the GDP contribution of milk from the system from the current 2.9 billion RWF to about 5.7 billion RWF, a 95% increase in five years (Table, 8)

## Activities time line and sequencing for CSD: Gantt chart

The Gantt chart presented in the IFD improvement section, above, works equally for both CSD and IFD systems

## Complementary intervention and success requirements for CSD

The following are crucial aspects of the dairy improvement interventions and success requirements for the CSD system:

- Provide incentives to investors and ease the bureaucracy to establish CSD farms.
- Make land accessible for forage production for the CSD farms and forage producers
- Enforce milk quality standards and payment schemes
- Support establishment/functioning of milk processing plants
- Enforce forages, concentrate feed and forage seed quality standards and ensure conducive environment for investment in production and marketing of feeds and feed seeds

## Medium Level of Investment Scenario (MLI)

The above proposed dairy interventions and the resulting improvements are the product of the RLI scenario applied in the livestock sector of Rwanda. What will be the impact of reducing this investment? Likely the impact will be reduced at least by half. An additional scenario analysis called Medium Level of Investment (MLI) Scenario is done to see the impact of reducing RLI investment. In the MLI scenario it is assumed that the RLI option would only be followed through the 3rd year of the 5-year LMP. Under the MLI scenario, the adoption rate of farmers and coverage of the dairy improvement technologies are assumed to be half the rates in the RLI scenario. Table 14 below summarizes the national milk production and GDP contribution under the MLI scenario and compares it with the other two investment level scenarios (BAU and RLI).

Table 14. Milk production and GDP contribution at three investment level scenarios by 2021/22

| **Livestock product** | **Baseline 2016/17** | **BAU Investment scenario (2021/22)** | **MLI scenario (2021/22)** | **RLI scenario (2021/22)** |
|---|---|---|---|---|
| National milk production (in million) | 747 | 1,041 | 1,082 | 1,230 |
| Milk GDP contribution (in billion RWF) | 113 | 158 | 158 | 173 |

As shown in Table 14 above, the medium investment scenario results in a slight increase in milk production and almost the same milk GDP contribution as the BAU investment scenario. The RLI scenario, on the other hand, shows a significant increase in both milk production and GDP contribution over the baseline, the BAU scenario, and the MLI scenario. The total cost of investment in the MLI scenario is about 25 billion RWF and is summarized in Table 15 below. This investment is half of the RLI, but brings little benefits over the BAU scenario.

Table 15. Five year dairy improvement investment capital costs under the MLI scenario (half the investment of the RLI scenario)

| No | Investment Intervention | Investment cost (10^6 RWF) | | | | | |
|---|---|---|---|---|---|---|---|
| | | 2017/18 | 2018/19 | 2019/20 | 2020/21 | 2021/22 | Total |
| 1 | Animal Feeding | 348 | 336 | 17 | 4 | 11 | 715 |
| 2 | Animal Health | 77 | 72 | 44 | 97 | 50 | 340 |
| 3 | Animal Breeding and Genetics Investments | 720 | 537 | 452 | 468 | 465 | 2,581 |
| 4 | Extension | 135 | 8 | - | - | - | 143 |
| 5 | Research | 187 | 415 | - | 477 | 415 | 1,494 |
| 6 | Marketing and Value Addition | 1,338 | 2,267 | 7,237 | 1,873 | 7,247 | 19,962 |
| | **GRAND TOTAL INVESTMENT** | **2,804** | **3,635** | **7,749** | **2,919** | **8,188** | **25,234** |

## *Conclusions*

The interventions proposed to improve cattle milk production and productivity in the RLI will transform traditional farms engaged in family dairy into more market-oriented improved family dairy systems (IFD) by:

Raising and realizing the genetic potential of local breeds for significantly higher milk production through crossbreeding with exotic dairy breeds using AI with or without synchronizing hormone, or use of bull services, combined with better feed and health services.

Milk production and productivity of the commercial specialized dairy system (CSD) will also increase significantly by:

Bringing more crossbred cattle into the commercial cattle dairy system; and

Increasing the availability of quality forage and concentrate feeds by improving forage and concentrate feed production and marketing.

Local cattle, or the vast majority of individual animals, also represent huge potential to bridge the national milk consumption and production gap. The intervention – mainly targeted at improving animal reproductive and weight gain performance – also increases animal productivity and total milk production significantly in all typology zones. These increases are achieved by:

The improvement of natural grazing (pasture and rangelands), coupled with health interventions to reduce mortality.

These combined interventions could result in:

A 65% increase in national cattle milk production over the period of 2016/17 – 2021/22.

The production of cow milk could increase from about 746 million liters by 2016/17 to 1.2 billion liters in 2021/22.

In addition to the above activities, the critical conditions which need emphasis for success of the plan are:

Encouraging the private sector to invest in feed production and CSD farms;

Ensuring availability of more and better feed, forage seed, forage production and marketing, and health services in all areas, whether breed improvement is implemented or not;
Ensuring more effective extension services to support production, processing and marketing of quality milk

It should also be noted that the following policy and institutional challenges need to be addressed to bring about the projected outputs:

Animal breeding strategy and regulation of animal breeding carried out
Livestock identification, traceability and disease surveillance reporting systems enforced
Enforcement of live animal and animal product traceability policy
Enforcement of milk products grading and incentive payments for milk quality
Enforcement and establishment of regulations for animal nutrition; standards on animal commercial feeds, crops residues, formulation of feeds, etc.
Standards on livestock infrastructure development enforced (slaughterhouses, laboratories, MCCs, milk processing factories, etc.)
Standards for vet and AI services and pharmaceuticals and supplies enforced
Strengthening the functional linkages and collaboration of government institutions from national to grassroots level, for enforcement of: all quality regulations and standards; livestock and animal products identification and traceability; and disease surveillance reporting systems

# Red meat value chain development roadmap

**(2017/18– 2021/22)**

# Red meat VC development roadmap (2017/18 – 2021/22)

## Introduction

Rwanda produced about 58,580 metric tons of red meat in 2016/2017, wherein 74 % is beef and the remaining 26% comes from sheep and goats. The red meat produced is predominately for domestic consumption, with little exports. Rather Rwanda imports a good deal of its meat and has a long way to go to meeting its domestic demand for red meat, and be able to export. There are technical, policy and institutional measures required to boost production and closing the domestic production-consumption gap to be able to pave the way for exporting.

## *Vision*

The projected production of red meat by 2021/22 is achieved and Rwanda will be able to meet the domestic demand for red meat arising from a rapidly growing population, increasing urbanization, and rising incomes; and Rwanda will be able to engage in the competitive international market so that live animal and meat exports will take place to generate foreign exchange earnings.

### Investment scenarios analyzed

To create the red meat roadmap, three levels of investment scenarios and their implications for total red meat production and contributions of the red meat systems to GDP are examined here to provide three investment choices for the decision makers in Rwanda; the government, private investors and development partners (donors). These scenarios are referred to here as BAU (Business As Usual), MLI (Medium Level of Investment) and RLI (Recommended Level of Investment). BAU presents the base case scenario analysis or the implications of the current level of investment and spending profile on red meat production and GDP contributions throughout the LMP analysis period of 5 years. RLI is the recommended level of investment which could achieve the agreed upon national development objectives. The MLI option analyzes the implications and impacts on red meat production and GDP of the third option created for decision makers who face a budget constraint. It represents a medium level of investment between the BAU and the RLI option. For this 3-option analysis, the MLI level of investment was obtained by assuming the RLI option would only be followed through the 3rd year (see tables 18, 19 and 20 below) of the 5-year LMP period. See the chicken road map for detail LMP three investment scenario analysis.

## *Overall targets under the recommended level of investment scenario (RLI)*

The overall target is to reach production of 79,586 tonnes of red meat by 2021/22, through improvement of feed from grazing land resources, animal health services, and animal genetics; and use of appropriate feeding technology. By 2021/22, a total of 64,400 heads of animals will pass through feedlot and non-traditional (culled dairy cattle) operations; and the contribution of local breeds to the overall red meat produced will be significantly reduced, leading the way for higher productivity improved beef breeds, young dairy calves and culled cows.

### Specific targets under RLI

The production targets are given for the specific red meat production systems by production typology zones in the tables below.

To facilitate the use of the analytical model created for this LSA study, a typology of the different livestock systems was developed, consisting at the first level in the (a) Low Rainfall Low Altitude (LRLA) zone which predominantly covers the eastern savanna and the eastern plateau of Rwanda with a significant feature of farmland based grazing; (b) the medium rainfall and medium altitude (MRMA) zone which is predominantly found in the central plateau of Rwanda; and (c) the high rainfall high altitude (HRHA) which covers the western highlands, or the Lake Kivu and South West regions. Livestock production practices which are modern, and which are to a large extent independent of location and the broader zonation are classified as specialized production (SP) systems and analyzed independently due to their unique modern production characteristics and commercial market orientation.

Table 16: Annual increase in number of cattle RLI

| | | **Base year 2016/17** | **2017/18** | **2018/19** | **2019/20** | **2020/21** | **2021/22** | **% Change** |
|---|---|---|---|---|---|---|---|---|
| LRLA | local | 209496 | 201,410 | 193,636 | 186,161 | 178,975 | 172,067 | -18% |
| | cross | 292822 | 316248 | 341548 | 368872 | 398382 | 430252 | 47% |
| MRMA | Local | 235092 | 225,172 | 215,669 | 206,568 | 197,851 | 189,502 | -19% |
| | Cross | 285171 | 307,985 | 332,624 | 359,234 | 387,972 | 419,010 | 47% |
| HRHA | local | 147176 | 140,936 | 134,960 | 129,238 | 123,758 | 118,511 | -19% |
| | cross | 203932 | 220,247 | 237,867 | 256,896 | 277,448 | 299,643 | 47% |
| Feedlot | local | 556 | 1,155 | 2,398 | 4,978 | 10,335 | 21,456 | 3,756% |
| Dairy with grazing | local | 13771 | 13,772 | 13,772 | 13,772 | 13,772 | 13,772 | 0% |
| Dairy with no grazing | cross | 3199 | 3,371 | 3,552 | 3,743 | 3,944 | 4,156 | 30% |
| Total | | 1,391,219 | 1,442,696 | 1,496,078 | 1,551,434 | 1,608,840 | 1,668,369 | 20% |

The total number of cattle shows an increase of 20% (1.4 to 1.7 million by 2021/22)
The total number of cattle in the Feedlot system[11] shows a growth of 3,756% (from 556 heads in 2016/17 to 21,456 in 2021/22)

[11] Per cycle, and three cycles per year

Table 17: Annual increase in number of sheep and goats in the traditional system under RLI

| Sheep | | Base year 2016/17 | **2017/18** | **2018/19** | **2019/20** | **2020/21** | **2021/22** | **change** |
|---|---|---|---|---|---|---|---|---|
| LRLA | local | 44,088 | 44,648 | 45,215 | 45,789 | 46,371 | 46,960 | 7% |
| | cross | 21,682 | 21,978 | 22,277 | 22,579 | 22,887 | 23,198 | 7% |
| MRMA | local | 234,896 | 240,910 | 247,077 | 253,402 | 259,889 | 266,542 | 13% |
| | cross | 73,721 | 75,572 | 77,469 | 79,413 | 81,406 | 83,450 | 13% |
| HRHA | local | 295,608 | 304,979 | 314,646 | 324,621 | 334,911 | 345,528 | 17% |
| | cross | 53,484 | 55,190 | 56,951 | 58,767 | 60,642 | 62,577 | 17% |
| Total | | 723,480 | 743,317 | 63,698 | 784,637 | 06,151 | 828,254 | 14% |
| Goat | | | | | | | | |
| LRLA | local | 1,126,400 | 1,188,690 | 1,254,424 | 1,323,794 | 1,397,000 | 1,474,254 | 31% |
| | cross | 32,351 | 35,055 | 37,986 | 41,162 | 44,603 | 48,332 | 49% |
| MRMA | local | 864,651 | 910,651 | 959,098 | 1,010,122 | 1,063,860 | 1,120,457 | 30% |
| | cross | 55,879 | 60,550 | 65,612 | 71,098 | 77,041 | 83,482 | 49% |
| HRHA | local | 794,068 | 832,421 | 872,627 | 914,775 | 958,959 | 1,005,276 | 27% |
| | cross | 67,643 | 73,298 | 79,425 | 86,065 | 93,260 | 101,057 | 49% |
| Total | | 2,940,991 | 3,100,984 | 3,269,681 | 3,447,554 | 3,635,105 | 3,832,858 | 30% |

The total sheep population in all production zones shows a 14% increase (reaching 828,000 by 2021/22)
The total number of goats in all production zones shows an increase of 30% by 2021/22 (from 2.9 million in 2016/17 to 3.8 million in 2021/22)

Table 18: Contribution of cattle to national red meat production (in tonnes) under RLI

| | | Base year 2016/17 | 2017/18 | 2018/19 | 2019/20 | 2020/21 | 2021/22 | change |
|---|---|---|---|---|---|---|---|---|
| LRLA | local | 5,124 | 4,940 | 4,762 | 4,591 | 4,425 | 4,266 | -17% |
| | cross | 10,384 | 11,251 | 12,190 | 13209 | 14,312 | 15,507 | 49% |
| MRMA | Local | 6,092 | 5,885 | 5,685 | 5,492 | 5,306 | 5,126 | -16% |
| | Cross | 10,050 | 10,902 | 11,826 | 12,827 | 13,914 | 15,093 | 50% |
| HRHA | local | **3,819** | 3,688 | 3,561 | 3,438 | 3,320 | 3,206 | -16% |
| | cross | 7,227 | 7,849 | 8,523 | 9,256 | 10,051 | 10,915 | 51% |
| | | 42,696 | 44,515 | 46,547 | 48,813 | 51,328 | 54,113 | 27% |

Table 19: Contribution of cattle Feedlot and dairy sub sector to national red meat production (in tonnes)

| Meat production | | Base year 2016/17 | **2017/18** | **2018/19** | **2019/20** | **2020/21** | **2021/22** | **change** |
|---|---|---|---|---|---|---|---|---|
| Fattening system | local | 64 | 134 | 278 | 576 | 1,196 | 2,484 | NA |
| Per-Urban Dairy | local | 622 | 629 | 637 | 645 | 653 | 661 | 6% |
| | cross | 170 | 180 | 191 | 202 | 214 | 227 | 34% |

The potential contribution of cattle to national red meat production grows from 43,553 (in 2016/17) to 57,500 tonnes by 2021/22 (32% increase).

The contribution of Feedlots to national red meat production grows from almost zero (in 2016/17) to 2,484 tonnes by 2021/22.
The contribution of the dairy subsector systems to national red meat production, through male calves and culled cows grows from 170 (in 2016/17) to 227 tonnes by 2021/22 (34% increase).

Table 20: Contribution of Sheep and Goat meat to national red meat production (in tonnes) under RLI

| Meat production | | 2016/17 | 2017/18 | 2018/19 | 2019/20 | 2020/21 | 2021/22 | change |
|---|---|---|---|---|---|---|---|---|
| **Sheep** | | | | | | | | |
| LRLA | local | 157 | 164 | 171 | 178 | 186 | 194 | 23% |
| | cross | 106 | 110 | 115 | 121 | 126 | 132 | 24% |
| MRMA | local | 837 | 884 | 934 | 986 | 1,041 | 1,099 | 31% |
| | cross | 354 | 374 | 395 | 418 | 441 | 466 | 32% |
| HRHA | local | 1054 | 1,119 | 1,189 | 1,263 | 1,342 | 1,425 | 35% |
| | cross | 263 | 280 | 298 | 316 | 337 | 358 | 36% |
| Total | | 2,771 | 2,932 | 3,102 | 3,282 | 3,473 | 3,674 | 33% |
| **Goats** | | | | | | | | |
| LRLA | local | 4523 | 4,920 | 5,352 | 5,823 | 6,334 | 6,890 | 52% |
| | cross | 216 | 242 | 270 | 302 | 338 | 377 | 74% |
| MRMA | local | 3507 | 3,767 | 4,047 | 4,347 | 4,670 | 5,017 | 43% |
| | cross | 372 | 416 | 466 | 522 | 585 | 656 | 76% |
| HRHA | local | 3187 | 3,444 | 3,721 | 4,020 | 4,344 | 4,694 | 47% |
| | cross | 450 | 504 | 565 | 632 | 708 | 794 | 76% |
| Total | | 12255 | 13,297 | 14,427 | 15,653 | 16,984 | 18,428 | 50% |

The amount of sheep meat produced grows from 2,700 tonnes (in 2016/17) to 3,674 tonnes by 2021/22 (33% increase).
The amount of goat meat produced grows from 12,225 tonnes (in 2016/17) to 18,428 tonnes by 2021/22 (50% increase).

## *Improved Traditional Cattle, Sheep and Goat*

Table 21: Key challenges and interventions related to improving red meat VC under the RLI scenario

| Challenges | Interventions to address challenges |
|---|---|
| **Feed** | |
| Lack of sufficient grazing areas to meet the feed needs of the animals<br>Poor quality grazing land resources<br>Inadequate knowledge on the use of crop residues and by-products<br>Limited availability of concentrates and feed supplements, when needed. | Sensitization on management of grazing lands<br>Rehabilitation of grazing land through over-sowing with improved grass and legumes;<br>Training and capacity building and skill development to increase the use and conservation of crop residues and by products.<br>Increase in the availability of cereals<br>Increased and better use of agro-industrial by-products from the processing of cereals/grains as concentrates for animal feeding.<br>Promotion of appropriate storage and marketing of concentrates and feed supplements<br>Training of farmers on forage seed multiplication, proper feeding, and availing improved plant materials |
| **Genetics** | |
| Low genetic improvement extension coverage<br>Poor farm genetics recording scheme and lack of harmonization<br>Lack of proven adapted exotic breeds for beef production<br>Sheep and goat parent stock lacking | The challenges of improving the genetic potential of local animals could be overcome through selection within the local breeds; establishing community based breeding programs, which include developing a farm recording scheme; as well as promoting an animal identification and traceability scheme.<br>Use of cross breeding programs to improve production and productivity of Cattle, sheep, and goats<br>Importation/acquisition of improved parent stocks to support breeders<br>Harmonizing existing livestock data |
| **Animal Health** | |
| Poor animal health extension advice;<br>Inefficient animal health services;<br>Inadequate supplies and qualities of vaccines and drugs; and<br>Poor quality control of drugs and supplies. | Strengthening animal health regulatory capacity under the coordination of the Livestock Sector Ministry<br>Promote private service providers<br>Enforce FMD and CBPP vaccinations<br>Increase efficiency in animal/animal products movement permits |
| **Marketing and processing** | |
| Poor market infrastructure;<br>Poor technical knowledge of VC actors, especially processing technicians;<br>Inadequate market information; and<br>Poor linkages between producers and processors and export abattoirs. | Strategic capacity building<br>Building infrastructure<br>Establishing slaughter houses and modern abattoirs |

## Key assumptions to achieve targets under RLI[12]

**Meat production from local cattle**

Intervention areas (production zones): Cattle meat improvement interventions can be practiced in all over the country and all the livestock production zones (low, medium and high rainfall zones).

**Priority interventions and assumptions**

**Feed and feeding**

- Establish backyard, hedge and live fence with improved grass and leguminous feed production at HH level. Feeds like Pennisetum, Brachiaria, Leucaena, Sesbana, **Calliandra, Chloris gayana, Panicum Maximum, desmodium sp, ...)**
- Improve production, marketing and quality control of forage and forage seeds through strengthening existing regulatory bodies
- Improve quality control of processed feeds and industrial by-products used as feed in the dairy system
- Increase the use of crop residues e.g.: maize drying on the field to be used and conserved for animal feed
- Over-sow grazing land with improved grass and leguminous forage seeds and use of fertilizer where applicable
- Allocate land for production of improved pasture/forage
- The timely harvesting of grass, and storage and conservation of hay.
- Invest in the production of forage seeds by GoR
- Increase the efficient use of forages and crop residues (e.g. conservation)
- Increase the available cereals (e.g. importation)
- Increase the use of concentrates from animal feed plants
- Mobilizing farmers associations to organize group purchase and distribution of processed feed
- Research on:
    - Successful forages species according to zones
    - The real quantity of forages and post-harvest that Rwanda can produce in comparison to actual and projected demands

**Health**

- Coverage of health service will reach 100%
- Privet vet service providers will be promoted.
- Farmers that adopt the recommended rate of tick control treatments and internal parasite control will reach 60% in the coming 5 years.
- All animals that need vaccination against endemic (ECF, BQ, brucellosis, Rabies,), epidemic diseases (FMD, LSD, RVF) will receive vaccines.
- Enforce FMD, CBPP free Rwanda to improve the acceptance of products from the country within 5 years.
- Rate (%) of animal/animal product movement permits issued will reach 100% in five years

[12] The detailed red meat interventions for all production are presented in the LSA report.

Strengthen veterinary diagnosis laboratories

**Extension**

Farmers will receive more intensive training in relation to local cattle management (feeding, breeding, deworming, tick control, hygiene and milk handling and transport)
Capacity of extension agents to be build
Increase extension services delivery through farmers associations

**Genetics**

The population of local breed cattle will decrease while the number of crossbred and the total number of cattle increases. The growth rate of cattle number should slow down and eventually become zero percent depending on the available feed.
The population of local breed decreases by an annual growth rate of about -4%.
The overall cattle population will be growing with only 2.7% the first five years and 1.63% and 0.5% the second and third five years.
Improve the efficiency of AI service through promoting privet AI practitioners and training of technicians
Continuous research on exotic meat breeds
Establish performance (and pedigree) records

**Marketing and processing**

Establish at least one export abattoirs[13]

**Meat from Sheep and Goats: Investment options**

**Intervention area/s (production zone/s):** Meat production from sheep can be practiced more in medium and high rainfall areas; and meat production form goats in all over the country and all the livestock production zones (low, medium and high rainfall zones).

I. **Priority interventions and assumptions**

**Feed and feeding**

Promote backyard, hedge and live fence with improved grass and leguminous feed production at HH level. Feeds like Pennisetum, Brachiaria, Leucaena, Sesbana, **Calliandra, Chloris gayana, Panicum Maximum, desmodium sp, ...)**
Improve quality control of processed feeds and industrial by-products used as feed in the dairy system
Increase the use of crop residues e.g: maize drying on the field to be used and conserved for animal feed

**In bigger farms:**

Over-sow grazing land with improved grass and leguminous forage seeds and use of fertilizer where applicable
Allocate land and produce pasture/fodder
Timely harvest grass, and conserve hay.

[13] The exact number of abattoirs to be determined after getting future production estimates

**Health**

Increase health coverage to reach 100%
Establish private vet service providers
Increase the number of farmers that adopt the recommended rate of tick control treatments and internal parasite control. Adoption to reach 60% in the coming 5 years.
Vaccination against endemic (PPR, BQ/Anthrax), epidemic diseases (FMD, RVF) will receive vaccines.
Improve the acceptance of products from Rwanda by creating an FMD, CBPP free Rwanda within 5 years.
Increase the Rate (%) of animal/animal product movement permits issued, to reach 100% in five years
Strengthen capacity for disease surveillance, quarantines, and supervise mass vaccination programs
Strengthen veterinary diagnosis laboratories

**Extension**

Carry out intensive training in relation to local and crossbreed goats and sheep management (feeding, breeding, deworming, tick control, hygiene and milk handling and transport)
Build the Capacity of extension agents
Increase extension services delivery through farmers associations

**Genetics**

Increase the population of crossbreed sheep (mostly Merino) in recommended areas, where owners practice good management
Establish record on the performance of crossbreed goats
Establish an on-farm herd/flock recording and selection scheme in the next 5 years to practice local breed selection and breed improvement
Improve the buck to ewe ratio at village level

## Investments under RLI

The time horizon for the project is 5 years.
Total investment budget is estimated at RWF 130,527 million, and 58% of the budget source is public. Private will provide 42% of the total budget respectively (Table 23) to be spent over the five year plan of the projects.

Table 22: Five-year Red Meat production improvement investment costs for RLI, the recommended investment scenario (2017/18 – 2021/22)

| Investment interventions | Investment cost (000,000 or million RWF) | | | | | Total Budget (million RWF) |
|---|---|---|---|---|---|---|
| | 2017/18 | 2018/19 | 2019/20 | 2020/21 | 2021/22 | |
| **Feeds interventions** | | | | | | |
| Supporting farmers on improving grazing land through over-sowing with improved grass and legumes | 5,500 | 2000 | 2000 | 1000 | 600 | 11,100 |
| Training and capacity building and skill development to increase the use and conservation of crop residues and by products. | 2000 | 1000 | 1000 | 1000 | - | 5,000 |
| Introducing technology on use of agro-industrial by-products from the processing of cereals/grains as concentrates for animal feeding | 1200 | 1000 | 1000 | 500 | 400 | 4100 |
| Establish appropriate storage and marketing of concentrates and feed supplements | 3500 | 2000 | 1800 | 1000 | - | 8300 |
| Training of farmers on forage seed multiplication, proper feeding, and availing improved plant materials | 575 | 350 | 275 | 245 | 245 | 1690 |
| **Total feed** | **12,775** | **6,350** | **6,075** | **3,745** | **1,245** | **30,190** |
| **Animal Health interventions** | | | | | | |
| Establish a national guideline for disease monitoring and controlling | 23 | - | - | - | - | 23 |
| Anti-parasite control and vaccination | 2000 | 3000 | 4000 | 4500 | 4500 | 18000 |
| Disease surveillance | 800 | 1000 | 700 | 700 | 700 | 3900 |
| Awareness creation among small producers on sanitation and housing | 700 | 500 | 500 | 500 | 450 | 2650 |
| **Total animal health** | 3523 | 4500 | 5200 | 5700 | 5650 | 24573 |
| **Genetics and breeding interventions** | | | | | | |
| Improve the genetic potentials of indigenous cattle-Ankole through selection | 600 | 500 | 450 | 450 | 450 | 2,000 |
| Importation of suitable breeds for feedlots | 16000 | 13500 | 7200 | 5700 | 5000 | 41,700 |

| Investment interventions | Investment cost (000,000 or million RWF) | | | | | Total Budget (million RWF) |
|---|---|---|---|---|---|---|
| | **2017/18** | **2018/19** | **2019/20** | **2020/21** | **2021/22** | |
| Establish an on-farm herd/flock recording and selection scheme in the next 5 years to practice local breed selection and breed improvement | 600 | 600 | 600 | 600 | 600 | 3,000 |
| **Total genetics** | **17200** | **14600** | **8250** | **6750** | **6050** | **46700** |
| **Marketing, extensions, and research interventions** | | | | | | |
| 2 Big slaughter houses | 1672 | 0 | 0 | 0 | 0 | 1672 |
| 1 Modern abboiter | 1200 | 0 | 0 | 0 | 0 | 1200 |
| Building the capacity of meat technology training staff | 2500 | 1000 | 240 | 200 | | 3940 |
| Developing programs to ensure food safety and animal health through monitoring of abattoirs | 1200 | | | | | 1200 |
| Establishing new feedlots infrastructure | 7000 | | 0 | 0 | 0 | 7000 |
| Establish quarantine facility | **4000** | | | | | 4000 |
| Extension services and research | **2000** | **1000** | **800** | **1200** | **5000** | 10000 |
| **Total marketing** | **19572** | **2000** | **1040** | **1400** | **5000** | **29012** |

Table 23:Total investment and recurrent costs for red meat VC development under RLI

| Investment category | Responsible actor (000 000 RWF) | | Total investment cost (million RWF) |
|---|---|---|---|
| | **Public** | **Private** | |
| Animal feeding | 21,890 | 8,300 | 30,190 |
| Animal health | 22,073 | 2,500 | 24,573 |
| Animal breeding and genetics | 5,000 | 41,700 | 46,700 |
| Research | 2,300 | 700 | 3,000 |
| Extension services | 3800 | 420 | 4,220 |
| Marketing and value addition | 18,000 | 3,844 | 21,844 |
| **Total investment** | 73,063 | 57,464 | 130,527 |

Table 23 below shows that 36% of the total red meat investment budget will go for animal breeding and genetics, and 73% this budget category will be sourced from the private sector (Table 23). The private sector investment amount for marketing and value addition is also quite small, especially compared to the public investment.

Table 24: The percent contribution of public and private investments by investment areas for improving the red meat VC under RLI

| Key investment area | Proportion (%) by responsible actor | | Proportions by key investment area |
|---|---|---|---|
| | **Public** | **Private** | |
| Animal feeding | 30% | 14% | 23% |
| Animal health | 30% | 4% | 19% |
| Animal breeding and genetics | 7% | 73% | 36% |
| Research | 3% | 1% | 2% |
| Extension services | 5% | 1% | 3% |
| Marketing and value addition | 25% | 7% | 17% |
| | 100% | 100% | 100% |

## Impacts

### Production impacts under RLI

Table 25 shows that under the RLI, the increases in red meat production from cattle, sheep and goats from 2016/17 (the baseline year) to 2021/22 is 32%, 33%, and 50 %, respectively. Cattle (72%) and goats (23%) are the primary sources of red meat in 2021/22. Total red meat grows from 58,579 tonnes in 2016/17 to 79,586 tonnes in 2021/22, an increase by 36%.

Table 25: Change in red meat production from cattle, sheep and goats under RLI (in tonnes)

| Species | 2016/17 (baseline) | 2017/18 | 2018/19 | 2019/20 | 2020/21 | 2021/22 | % Change in production |
|---|---|---|---|---|---|---|---|
| Cattle | 43554 | 46040 | 48667 | 51445 | 54380 | 57484 | 32% |
| Sheep | 2771 | 2932 | 3102 | 3282 | 3473 | 3674 | 33% |
| Goats | 12254 | 13296 | 14426 | 15653 | 16984 | 18428 | 50% |
| Total | 58579 | 62267 | 66196 | 70380 | 74837 | 79586 | 36% |

Table 26 shows negative changes in production of meat from local cattle breeds and big positive production changes over time from crossbreds in all the production zones. This negative change in meat production from local breeds is expected due to the population decreases of local cattle breeds as they are replaced by the crosses or pure exotic breeds. The *Girinka* program or **"One Cow per Family Program"** of MINAGRI is transforming dairying and meat production in Rwanda, through the provision of pregnant crossbred cows to smallholders. As well, the number of cattle going through the feedlot fattening system for meat production is expected to **increase rapidly, partly as a result of the government's Gako** program which is seeking to improve the meat breeds in Rwanda, especially with Ankole cattle.

Table 26: Red meat production from local and crossbred cattle by system and typology zone for the baseline year (2016/17) and 2021/22 with the RLI intervention (in tonnes)

| Production zone | Cattle | Total red meat in 2016/17 (tonnes) - baseline | Total red meat in 2021/22 (tonnes)- with red meat intervention | change in production due to red meat intervention |
|---|---|---|---|---|
| LRLA | local | 5124 | 4,266 | -17% |
| | cross | 10384 | 15507 | 49% |
| MRMA | local | 6092 | 5,126 | -16% |
| | cross | 10051 | 15,093 | 50% |
| HRHA | local | 3819 | 3,206 | -16% |
| | cross | 7228 | 10,915 | 51% |
| Fattening | local | 64 | 2,484 | 3756% |
| Dairy with grazing | local | 622 | 661 | 6% |
| Dairy with no grazing | cross | 170 | 227 | 34% |
| **Total** | | **43554** | **57,484** | **32%** |

**GDP impact**

Table 27 shows that under RLI the GDP contribution from red meat coming from cattle, sheep and goats from all production zones could show an overall increase by 26% comparing the base year with the 2021/22 projection. This amounts to RFW 95,145 in 2016/17, and RWF 120,161 in 2021/22.

Table 27: GDP contribution from red meat improvement under RLI (in RWF millions)

| Species | 2016/17 | 2017/18 | 2018/19 | 2019/20 | 2020/21 | 2021/22 | % Change in production |
|---|---|---|---|---|---|---|---|
| Cattle | 66,126 | 68,479 | 70,916 | 73,439 | 76,052 | 78,758 | 19% |
| Sheep | 4,846 | 5,091 | 5,349 | 5,619 | 5,904 | 6,202 | 28% |
| Goats | 24,173 | 26,060 | 28,094 | 30,288 | 32,652 | 35,201 | 46% |
| Total | 95,145 | 99,692 | 104,457 | 109,449 | 114,680 | 120,161 | 26% |

Table 28: Cattle GDP for baseline year (2016/17) and 2021/22 with red meat intervention in all zones and systems (in RWF 000,000) under RLI

| | | Total livestock GDP 2016/17 (in RWF 000,000) - baseline | Total livestock GDP 2021/22 (in RWF 000,000) - with red meat intervention | % change in national livestock GDP |
|---|---|---|---|---|
| LRLA | local | 7595 | 6,147 | -19% |
| | cross | 16,411 | 22021 | 34% |
| MRMA | Local | 8,576 | 7,015 | -18% |
| | Cross | 15,607 | 21,175 | 36% |
| HRHA | local | 5,384 | 4,397 | -18% |
| | cross | 11,269 | 15,493 | 37% |
| Fattening | local | 38 | 1,491 | 3,756% |
| Dairy with grazing | local | 1050 | 852 | -19% |
| Dairy with no grazing | cross | 195 | 169 | -13% |
| Total | | 66,126 | 78,758 | 19% |

The GDP contribution of red meat coming from cattle from all production zones and systems could show an overall increase by 19% comparing the base year with the 2021/22 projection. This amounts to RWF 66,126 in 2016/17, and 78,758 RWF in 2021/22.

Table 29: Activity timeline and sequencing: Gantt chart RLI

| Activities | 2016/17 | | 2017/18 | | 2018/19 | | 2019/20 | | 2020/2011 | |
|---|---|---|---|---|---|---|---|---|---|---|
| | Jul-Dec | Jan-June | Jul-Dec | Jan-June | Jul-Dec | Jan-June | Jul-Dec | Jan-June | Jul-Dec | Jan-June |
| Anti- parasitic control and treatment | | | | | | | | | | |
| Adult stock immunization | | | | | | | | | | |
| Young stock immunization | | | | | | | | | | |
| Animal disease surveillance | | | | | | | | | | |
| Strategic feed supplementation to the dams | | | | | | | | | | |
| Introduction of flock/herd recording scheme | | | | | | | | | | |
| Fodder production initiatives to get land | | | | | | | | | | |
| Grazing land rehabilitation | | | | | | | | | | |
| Extension work to support improved feeding of cattle, sheep, and goats | | | | | | | | | | |

## Complementary interventions and success requirements

Empowering and supporting RCVD and increasing the number of extension agents.

Infrastructure improvement (feeder road, water, electricity, crushes, etc.)

Reinforcement of feedlots and animal feed factories and distribution through the selling points across the country

Strengthening the legal framework in the livestock sector (reinforcing the existing framework, and reviewing, developing and enacting/enforcing the missing regulations)

Encouraging the private sector to invest in meat slaughter and processing plants.

Putting in place the grading and pricing of animal products

Increasing access to loans banks

Making livestock insurance available

Improving technologies through livestock for records keeping

Introduction through enacting a farm data records policy

Introducing a practical herd recording system

A special support to farmers associations such us R.P.F.A (Rwanda Pig Farmers Association) and R.P.I.A (Rwanda Poultry industry Association) as the main actors in meat value chain, for empowering and strengthening them.

Provide avenue to the farmers associations to contribute to the extension of livestock and an increase of meat production.

## *Specialized cattle feedlots and culled dairy cattle*

### Meat from improved dairy cattle

**I. Intervention area(s) (production zone(s)):**

Meat production from improved dairy can be practiced in all over the country and all the livestock production zones (low, medium and high rainfall zones)

**II. Priority interventions**

Promote the production of calf stall feeding and veal production system
The minimum number of calves per unit farm that can have sufficient economic of scale is about 20 calves/farm/cycle giving an average of about 3,000 USD/farm/year.

### *Meat from increase in the number of commercial big farms*

**I. Intervention area(s) (production zone(s)):**
Intervention area(s) (production zone(s)): Meat production from big commercial farms is being practiced in area designated for improved ranching system in the low rainfall production area.

***II.*** **Priority *interventions***
Farmers need to be recommended which specific breeds to rear in the farms
Increase the herd size of big private farms (in Gako) by range of 20-30% through purchase of additional heifers in the first three to four years; and maintaining constant herd size, once the carrying capacity is reached.
Promote and assist farm owners to practice more intensive production systems

### Targets

Table 30: Contribution of the cattle feedlot system to national meat production (in tonnes) under RLI

| Amount of meat (tonnes) | 2016/17 | 2017/18 | 2018/19 | 2019/20 | 2020/21 | 2021/22 | % change |
|---|---|---|---|---|---|---|---|
| Fattening | 64 | 134 | 278 | 576 | 1196 | 2484 | 3,756% |

Table 31: Contribution of the dairy production system to national meat production (in tonnes)

| Amount of meat (tonnes) | 2016/17 | 2017/18 | 2018/19 | 2019/20 | 2020/21 | 2021/22 | % change |
|---|---|---|---|---|---|---|---|
| Dairy with grazing | 622 | 629 | 637 | 645 | 653 | 661 | 6% |
| Dairy with no grazing | 170 | 180 | 191 | 202 | 214 | 227 | 34% |
| **Total** | **792** | **809** | **828** | **847** | **867** | **888** | **40%** |

Table 32: Challenges and interventions in the specialized systems under RLI

| Challenges | Interventions to address challenges |
|---|---|
| **Feed** | |
| Limited access to land for production of forage seed and forage;<br>Unable to meet the feed demand that is required at commercial feedlots<br>Poor access to quality concentrate feed; and inadequate concentrate available<br>Lack of effective feed quality control: standards and mechanisms of enforcement missing | Making land available to forage production investors;<br>Promoting production of forage for commercial feedlots;<br>Promoting the establishment of flour mills to make more concentrates available;<br>Strengthening the feed quality control authority to expand its operations<br>Promoting the establishment of agro-industries for increased availability of by-products that could be used as feed supplements. |
| **Animal Health** | |
| Poor animal health extension advice;<br>Inefficient animal health services;<br>Inadequate supplies of drugs;<br>Poor quality control of drugs and supplies;<br>Poor disease surveillance;<br>A lack of traceability and identification; and<br>Inadequate quality control in abattoirs. | Strengthening the animal health regulatory capacity under the coordination of the Livestock Sector Ministry is the main thrust. |
| **Marketing and processing** | |
| Absence of quality based pricing;<br>Lack of holding area and feedlot space;<br>Lack of knowledge and skill on meat-cutting and –grading; and<br>Poor links to export abattoirs. | Building the capacity of meat technology training staff at the TMB;<br>Increasing training of meat processing staff;<br>Promoting forward contracting of feedlots and abattoirs; and<br>Investing in export infrastructure for animal holding and quarantine, as well as programs to ensure food safety and animal health through disease surveillance, monitoring of abattoirs, animal identification and traceability, etc. |
| **Policy** | |

| Challenges | Interventions to address challenges |
|---|---|
| A lack of meat quality standards control and enforcement, grading, and pricing policies;<br>The policy on breeding not fully implemented ;<br>A need to strengthen feed production and land acquisition for feedlot investment;<br>Inadequate feed quality monitoring and controlling;<br>Need for further incentives to establish GAKO type feedlots (including land access in appropriate locations conducive to feed production, linkages with export market, and infrastructure – road access, power and water supply); | Development of appropriate standards for meat quality<br>Development guidelines on feed quality monitoring<br>Capacity building on quality control |

## Interventions to achieve targets under RLI

### Increasing the number of animals fattened

The specialized production feedlot system could be improved through better feed and health services, increasing the number of cattle feedlot units, and the number of cattle being fattened. Producers (fatteners) could be given training on cattle fattening procedures, including cattle selection and feeding; and on improving the efficiency of the beef value chain, targeting quality beef marketing.

Table 33: Projected number of cattle fattened under RLI

| | 2016/17 | 2021/22 | % change |
|---|---|---|---|
| Number of animals fattened/ unit/ year | 556 | 21,456 | 3,757 |
| Total cattle fattened[14] | 1,668 | 64,368 | |

### Increasing the availability of feed ingredients required by cattle feedlots

Table 34: Estimated amount of additional concentrate feed needed for additional cattle going to feedlot by the year 2021/22 under RLI

| | Number of animals | | Additional number of animals in 2021/22 relative to 2016 | Additional concentrate/year (thousand tonnes) |
|---|---|---|---|---|
| | 2016/17 | 2021/22 | | |
| | 234,333 | 1,795,974 | 1,561,641 | 15616 |

The additional concentrate needed per animal will be 0.01 tonnes per year.

[14] Number of cycles per year is 3

## Investments under RLI in cattle feedlot

Table 35: Investment in slaughter house establishment under RLI

| Type of processing | Cost per unit (RWF 000,000) | Number of new slaughter houses | Capacity | Investment cost/ plant (RWF 000,000) | Area/ population covered |
|---|---|---|---|---|---|
| Big slaughter houses (with rendering system) | 836 | 2 | 2,000 sheep and goats and 200 cattle per day | 1672 | Towns and cities with 200,000 population |
| Modern abattoir (with all required facilities) | 988 | 1 | 3,000 sheep and goats and 700 cattle per day | 1200 | Towns and cities with 200,000 population |

## Impacts RLI on cattle feedlot

### *Production*

Table 36: Change in meat production from cattle feedlots (2016/17 to 2021/22) with feedlot intervention under RLI

| Products | Total production 2016/17 baseline | Total production 2021/22 - with commercial feedlots intervention | % change in production |
|---|---|---|---|
| Beef in tonnes | | | |
| Cattle | 43,553 | 57,484 | 32% |

Total red meat production from cattle increases by 32% in 2021/22, from 43,553 tones in the base year, to 57,484 tonnes in 2021/22.

### *GDP from cattle feedlots under RLI*

Table 37: Change in Livestock GDP due to interventions on cattle feedlots under RLI

| Product (meat) | Total livestock GDP 2016/17 (RWF 000,000) - baseline | Total livestock GDP 2021/22 (RWF 000,000) - with specialized feedlots interventions | % change in national livestock GDP |
|---|---|---|---|
| Cattle | **66,126** | **78,758** | 19% |

Table 38: Activity timeline and sequencing: Gantt chart RLI

| **Activities** | **2016/17** | | **2017/18** | | **2018/19** | | **2019/20** | | **2020/2011** | |
|---|---|---|---|---|---|---|---|---|---|---|
| | Jul-Dec | Jan-Jun | Jul-Dec | Jan-Jun | Jul-Dec | Jan-Jun | Jul-Dec | Jan-June | Jul-Dec | Jan-June |
| Implementing the roadmap for the rationalization of PP veterinary services | | | | | | | | | | |
| Anti-parasitic treatment | | | | | | | | | | |
| Adult stock vaccinations | | | | | | | | | | |
| Disease surveillance | | | | | | | | | | |
| Establishing quarantine facilities | | | | | | | | | | |
| Establishing identification and traceability tools | | | | | | | | | | |
| Quality control in Abattoirs | | | | | | | | | | |
| Identifying potential locations for feedlot establishment | | | | | | | | | | |
| Creating new feedlots | | | | | | | | | | |
| Ensuring MoA support for establishment of feedlots in strategic locations | | | | | | | | | | |
| Establishing slaughter houses | | | | | | | | | | |

## Complementary interventions and success requirements RLI

Access enabled to sufficient production factors
Promoting the conducive policy and investment environment required to attract and facilitate private sector investment in feedlots and slaughter houses operations.

## Medium Investment level scenario (MLI)

The medium level investment scenario (MLI) assumes a budget constraint to investing fully (as in the recommended case -- RLI). The key assumption made here to simulate the MLI is that only half of the RLI is made available due to the budget constraint.

**Key challenges and interventions under MLI:** Key challenges and interventions to achieve the targets under the MLI remain the same as in the RLI for red meat production.

### Impacts under MLI scenario

Table 39: Rwanda red meat production under BAU and MLI (2021/22)

| Livestock product | 2016/17 (base year) | 2021/22 BAU | 2021/22 With MLI case | % difference MLI/base year | % difference MLI/BAU |
|---|---|---|---|---|---|
| Cattle | 43,554 | 53,728 | 55,552 | 28% | 3% |
| Sheep | 2,771 | 3,171 | 3,363 | 21% | 6% |
| Goats | 12,254 | 16,049 | 16,784 | 37% | 5% |
| Total | 58,579 | 72,948 | 75,699 | 29% | 4% |

Table 39 above shows that under the MLI scenario the impact on red meat production is moderately high as compared with the baseline situation. However, when compared with the BAU scenario the red meat production under the MLI option is higher by 4%.

Table 40: Rwanda red meat value added or GDP impacts under BAU and MLI (2021/22)

| Red meat GDP | 2016/17 (base year) | 2021/22 BAU | 2021/22 With MLI case | % difference MLI/base year | % difference MLI/BAU |
|---|---|---|---|---|---|
| Cattle | 66,126 | 82,410 | 79,444.80 | 20% | -4% |
| Sheep | 4,846 | 5,538 | 5,789.60 | 19% | 5% |
| Goats | 24,173 | 31,615 | 33,007.80 | 37% | 4% |
| Total | 95,145 | 119,563 | 118,242.20 | 24% | -1% |

Similarly, in Table 40 above, the value added from all red meat animals under the MLI scenario is higher than the baseline situation by 24% and lower than the BAU scenario by 1%.

Table 41: Activity timeline and sequencing: Gantt chart MLI

| Activities | 2016/17 | | 2017/18 | | 2018/19 | | 2019/20 | | 2020/2011 | |
|---|---|---|---|---|---|---|---|---|---|---|
| | Jul-Dec | Jan-Jun | Jul-Dec | Jan-Jun | Jul-Dec | Jan-Jun | Jul-Dec | Jan-June | Jul-Dec | Jan-June |
| Implementing the roadmap for the rationalization of PP veterinary services | | | | | | | | | | |
| Anti-parasitic treatment | | | | | | | | | | |
| Adult stock vaccinations | | | | | | | | | | |
| Disease surveillance | | | | | | | | | | |
| Establishing quarantine facilities | | | | | | | | | | |
| Establishing identification and traceability tools | | | | | | | | | | |
| Quality control in Abattoirs | | | | | | | | | | |
| Identifying potential | | | | | | | | | | |

| Activities | 2016/17 | | 2017/18 | | 2018/19 | | 2019/20 | | 2020/2011 | |
|---|---|---|---|---|---|---|---|---|---|---|
| | Jul-Dec | Jan-Jun | Jul-Dec | Jan-Jun | Jul-Dec | Jan-Jun | Jul-Dec | Jan-June | Jul-Dec | Jan-June |
| locations for feedlot establishment | | | | | | | | | | |
| Creating new feedlots | | | | | | | | | | |
| Ensuring MoA support for establishment of feedlots in strategic locations | | | | | | | | | | |
| Establishing slaughter houses | | | | | | | | | | |

Table 42: Total investment and recurrent costs for red meat production under MLI

| Investment category | Responsible actor (000 000 RWF) | | | Total investment cost (000 000 Tsh) |
|---|---|---|---|---|
| | Public | Private | PPP | |
| Animal feeding | 10,945 | 4000 | | 14,945 |
| Animal health | 11,037 | 1,250 | | 12,287 |
| Animal breeding and genetics | 2,500 | 20,850 | | 23,350 |
| Research | 1,200 | 350 | | 1,550 |
| Extension services | 2000 | 210 | | 2,210 |
| Marketing and value addition | 9,000 | 1,100 | **817** | 10,917 |
| **Total investment** | **36,682** | **27,760** | **817** | **65,259** |

As noted in table 42 above the total investment in the MLI is less than half of the RLI. This represents a seriously constrained investment option.

## *Conclusions*

The MLI impact on production and value added is substantial when compared with the baseline situation, but not encouraging at all when compared with the BAU situation. This result demonstrates that livestock, in particular cattle, which have a long production cycle require long-term and adequate investment commitments to realize their potential. Short term and limited investment means that the investment ceases before maturity, and thus ends up with limited production benefits, as depicted in the MLI situation here. Hence, the MLI is not a recommended option at least for red meat production improvement.

Given the potential production increases under the RLI initiative, there is a significant contribution made by the production systems i.e. the traditional cattle operations and Feedlots, towards improving food security, closing the red meat consumption and nutrition gap, and contributing towards economic growth. However, this can only be realized if:

- The RL investments are implemented by the GoR and private investors in a timely manner; and are adequately funded.
- Meeting the feed needs becomes a priority, and is followed by increasing pasture access (where possible) and fodder production, as well as increasing the availability of roughages, such as crop residues and agro-industrial by-products. The bulk of additional concentrate feed needed, particularly in Feedlots, is expected to come from investment by the private sector.
- The policy environment to attract and enable sustainable growth in feedlots is continued and the vision of GAKO is fully implemented as a public and private partnership.

Linkages are established for a viable stocker feeder program where the improved young male stock from the traditional sector are channelled to feedlot operations, including under GAKO, thus reducing the grazing pressure on the very limited grazing land in the traditional system.

It must also be noted that the technical interventions alone will not bring about the projected outcomes unless the existing policy and institutional challenges are addressed.

Needed policies and strategies **–** to be enacted and implemented

- On-farm recording scheme policy
- Livestock products pricing policy
- Animal breeding strategy
- Livestock Coding System Policy
- Livestock identification & reporting system policy
- Livestock animal and product traceability policy

Regulations and standards

- Regulations for animal breeding
- Regulations for animal nutrition; standards on animal commercial feeds, crops residues, formulation of the feeds, honey, etc.
- Regulations for livestock products pricing and standards on animal products and grading (milk, meat, honey, eggs, wool, hides and skins)

- Standards on livestock infrastructure development (slaughterhouses, laboratories, MCCs, milk processing factories, etc.)
- Standards on vet services and pharmaceuticals and supplies

Institutional framework

- Strengthening the functional linkages and collaboration of institutions from national level to grassroots

# Chicken value chain development roadmap

(2017/18-2021/22)

# Chicken value chain development roadmap (2017/18-2021/22)

## *Vision*

The chicken industry in Rwanda will transition from largely subsistence to a more knowledge intensive market oriented sub-sector adding growth and value to poultry products. Both the family and specialized commercial operations will be transformed by using improved and highly productive breeds to ensure household food and nutritional security and higher incomes and significantly contribute to achieving the all-meat consumption needs of the nation.

**Investment scenarios analyzed**
In the investment analysis done to create LMP roadmaps, three levels of investment scenarios and their implications for total chicken production and contributions of the chicken sub-sector to GDP are examined here to provide three investment choices for the decision makers in Rwanda; the government, private investors and development partners (donors). These scenarios are referred to here as BAU (Business As Usual), MLI (Medium Level of Investment) and RLI (Recommended Level of Investment). BAU presents the base case scenario analysis or the implications of the current level of investment and spending profile on chicken meat and eggs production and GDP contributions throughout the LMP analysis period of 5 years. RLI is the recommended level of investment which could achieve the agreed upon national development objectives. The additional scenario or MLI option analyzes the implications and impacts on chicken meat and eggs production and GDP of the third option created for decision makers who face a budget constraint. It represents a medium level of investment between the BAU and the RLI option which could not achieve the national development objectives. For this 3-option analysis, the MLI level of investment was obtained by assuming the RLI option would only be followed through the 3$^{rd}$ year of the 5-year LMP period.

**Transforming the traditional village chicken production system**

Transformation of the traditional village chicken production system involves improvement in the traditional chicken value chain (VC) through improved productivity and marketing and scaling up of the commercial chicken sub system (CFC) and integrating it with the upper end of the value chain.

**Transforming the Traditional Family Chicken (TFC) to Improved Family Chicken (IFC)**

The proposed transformation of the TFC will have two components. One is to improve the indegenous chicken productivity through improved breed selection, health, feed and mangement interventions and the second one is along with health, feed and mangmnet interventions to introduce and distribute higher yielding pure tropical breeds that do not reporoducce themselves and distribute them to producers.

Improvement of the traditional family chicken can be practiced all over the country and in all livestock production zones (LRLA, MRMA and HRHA zones).

## A. Scaling up the commercial specialized chicken production (SP layers and broilers)

The scaling up of the specialized chicken systems involve increasing the scale of operations or average number of commercial layers and broilers kept per farm, and increasing the number of specialized farms. Build capacity and put in place strict biosafety systems Integrate commercial production and marketing from downstream value chain to the upper end of the value chain including processing.

## *Overall Targets under the Recommended Level of Investment Scenario (RLI)*

The overall target for the Chicken development roadmap (2017/18-2021/22) is based on the RLI option which is to more than double the annual chicken meat production from the current 15.2 thousand to 35 thousand tonnes and the egg production from the current 244 thousand to 513 thousand eggs by 2021/22 through implementing the Improved Traditional Family Chicken (ITFC), Crossbreed Family Chicken (CFC) and expanded Specialized/Commercial Chicken (SP) systems – with Layers and Broilers. The recommended level of investment or RLI is needed to meet these targets.

## *The improved Traditional Family Chicken (ITFC) and Crossbreed Family Chicken (CFC) sub-systems (20167/17-2021/22)*

### Targets for ITFC and CFC under RLI

Table 43: Number of hens and chicken eggs and meat production in ITFC and CFC 2016/17-2021/22 (RLI)

| Chicken systems | Unit | 2016/17 (baseline) | 2017/18 | 2018/19 | 2019/20 | 2020/21 | 2021/22 | % change |
|---|---|---|---|---|---|---|---|---|
| ITFC hens | million | 5.2 | 5.5 | 5.9 | 6.2 | 6.6 | 7.0 | 34% |
| CFC hens | million | 0.001 | 0.002 | 0.004 | 0.011 | 0.027 | 0.068 | NA* |
| Total hens | million | 5.201 | 5.502 | 59.004 | 6.211 | 6.627 | 7.068 | 35% |
| ITFC eggs | Thousand | 83,310 | 97,712 | 114,605 | 134,418 | 157,657 | 184,913 | 122% |
| CFC eggs | Thousand | 60 | 150 | 374 | 935 | 2,337 | 5,841 | 9666% |
| Total egg | Thousand | 83,370 | 97,862 | 114,979 | 135,353 | 159,994 | 190,754 | 129% |
| ITFC meat | MT | 5,080 | 5,690 | 6,372 | 7,137 | 7,993 | 8,951 | 76% |
| CFC meat | MT | 1 | 2 | 4 | 11 | 27 | 67 | 9666% |
| Total meat | MT | 5,081 | 5,692 | 6,377 | 7,147 | 8,019 | 9,018 | 77% |

The number of hens in the ITFC grows from 5.2 million in the base year to 7 million in 2021/22, a 34% increase.
Number of hens in the CFC grows from almost nil to 68,000.
Chicken meat production from ITFC increases from 5.1 thousand tonnes in 2016/17 to 8.95 thousand tonnes in 2021/22, a 76% increase
Chicken meat production from the CFC increases from 1 ton in 2016/17 to 67 tonnes by 2022.

*CFC is a newly introduced chicken system that does not exist, or hardly exists, in the base year.

## Key assumptions for the ITFC and CFC systems under RLI

- The flock size in ITFC will increase from 1 to 5 improved hens
- Reproductive wastage due to time spent in brooding will be reduced by 60%.
- Eggs laid per hen/year will increase from 40 to 90 on average
- Average weight of sold adult chicken increases from 1.4 kg to 1.7 kg.
- Chicken mortality before marketing will drop down from 50% to 10%.
- The chicken will be supplemented with locally available feeds by up to 50% of their requirements
- With dual-purpose crossbred chickens the minimum flock size is 25 hens; with a production of 150 eggs/hen/year on average and weight at marketing will be 2.5 kg
- Adequate space for housing, better health services and feed supplementation is provided is delivered for dual purpose chicken

***The detailed and quantified interventions are given in the Livestock Sector Analysis (LSA) report in the Interventions Section.**

Table 44: Key challenges and interventions related to improving the family systems (ITFC and CFC) under the RLI

| Challenges | Interventions to address the challenges |
|---|---|
| **Feeds** | |
| Shortage of maize, soy beans and other raw materials to enable the existing chicken feed processing plants operate at their full capacity<br>Competition between human and mono-gastric animals for locally produced maize | Make more land available for production of additional cereals for production of processed feed (maize and soybean);<br>Facilitate import of chicken feed ingredients, maize and soybean. |
| Low nutritive value and low quality of commercial chicken feeds<br>Low institutional capacity to control quality of chicken feed produced and processed | Build capacity for Animal feed inspectors in various levels.<br>Strengthen feed quality testing labs to regulate feed quality (periodical processors compliance to set Standards)<br>Create awareness through sensitization on quality of chicken feeds<br>Build capacity for Animal feed processors Good processing practices<br>Regulating the exportation of oil crops and importation of cooking oils; and |
| Low incentive for the private sector to invest in feed processing plants | Tax holiday and other incentives to encourage the participation of private sector in the feed processing industries.<br>Provide incentives and suitable investment to promote agro-processing industries and feed processing factories<br>Facilitate establishment of additional large-scale feed processing plants to cater the |

| Challenges | Interventions to address the challenges |
|---|---|
| | growing chicken production |
| Low capacity of framers to formulate their own chicken feeds using local available materials | Extension and capacity building of farmers on technology and methods of formulating feed rations for chicken using locally available materials |
| **Genetics** | |
| Low genetic potential of the local chicken breeds | Improve the genetics composition of indigenous chicken breeds through selection within the local breeds for ITFC<br>Identify and distribute higher yielding dual purpose tropical adaptable chicken breeds to replace the less productive indigenous breeds in CFC<br>Reduce reproductive wastage of brooding hens using artificial incubation artificial brooding facilities in both |
| Lack of availability of commercial day old chicks: the national supply of day old chicks is very low. Almost all the chicks are imported, mostly from Uganda, Belgium and Netherlands. | Encourage private sector to invest in hatcheries and distribution of cross-bred DOCs and/or pullets to satisfy the increase in DOC demand<br>Through PPP upgrade and strengthen the existing government hatcheries |
| **Animal health** | |
| Prevalence of chicken diseases particularly ND, FP, IBD/Gumboro<br>Poor housing and sanitation | Formulate bio safety guidelines for disease control<br>Enforcing stricter disease controls on the importation of commercial replacement stock;<br>Introduce chicken health management system<br>Strengthen the capacity for disease surveillance, quarantines, and supervise mass chicken vaccination programs<br>Rationalize public and private veterinary services, with privatization where feasible<br>Awareness creation among small producers on sanitation and housing |
| **Marketing and processing** | |
| Weak chicken producers organizations<br>Lack of cutting and processing facilities to produce market suitable chicken meat products<br>Lack of slaughtering facilities for chicken<br>Lack of egg processing plants | Promote establishment of Rwanda chicken traders associations;<br>Facilitate construction of modern chicken cutting and processing plants<br>Facilitate construction of modern chicken slaughter houses<br>Facilitate construction of egg processing plants |

| Challenges | Interventions to address the challenges |
|---|---|
| | catering to domestic and export markets<br>Promote consumption of exotic chicken meat and egg in domestic households in Rwanda<br>Build capacity and put in place strict biosafety systems<br>Strengthen meat inspections (at abattoirs, standardized mechanized slaughters, and processing facilities |
| Weak Biosafety and HACCP facilities | Institutionalize mandatory biosafety and HACCP |
| Consumers poor preference for exotic chicken meat and eggs | Intensifying the promotion and extension work to change the attitudes of consumers towards consuming eggs and meat from hybrid and exotic breeds |
| **Extension** | |
| | Strengthen extension services to improve the management skills of households raising indigenous and dual purpose chicken<br>Build the capacity of extension agents<br>Promote private extension service providers<br>Increase the delivery of extension services and awareness through associations and co-operatives |
| **Policy** | |
| Policy related to importation of raw materials for processed feeds and regulatory enforcement of hatcheries and breeding farms. | Strengthen quality audit and compliance of the commercial feeds,<br>Establish guidelines and standard operating procedures for operation of hatcheries and breeder farms.<br>Establish guidelines for operation of hatcheries breeder farms,<br>Promote registration of hatcheries and breeder farms<br>Avail credit access to farmers and other actors in the value chain, |
| Policy related to land acquisition for chicken feed production. | Favorable policy for land acquisition for chicken feed production. |

## Interventions to achieve targets (RLI)

The proposed improvement of the traditional family chicken system via RLI transformation to the ITFC and CFC systems has two components: the first is to improve the productivity of indigenous chicken through improved breed selection, along with complementary health, feed and management interventions; and the second is that along with the health, feed and improved management interventions to introduce and distribute higher yielding dual

purpose tropical crossbreds. As well, reproductive wastage will be reduced by introducing artificial brooding facilities and incubators. Improvement of the traditional family chicken system can be practiced all over the country, in all livestock production zones (LRLA, MRMA and HRHA zones).

## ITFC and CFC impacts (RLI)

### Production impacts

As the result of the intervention, the number of indigenous hens in the traditional family system grows from 5.2 million in the base year to 7 million in 2021/22, a 34% increase and the number of the newly introduced dual purpose improved family crossbreed chicken grows to 68 thousand. This is a slow process that involves testing for adaptability.

Total chicken meat from the family system increases from 5.1 thousand tonnes in year 2016/17 to 9 thousand tonnes in 2021/22 a 77% increase. Similarly, over the same period egg production increases by 129%, i.e., from 83.4 million to 190.8 million.

### GDP impacts

As shown in table 45 below, as a result of the additional interventions, the GDP contribution from the improved family chicken systems increases from RWF 20,317 million in 2016/17 to 37,128 million in 2021/22, a 72% increase which is substantial growth over 5 years.

Table 45: Changes in GDP contribution of improved family chicken (RLI)

| Products | Chicken GDP 2016/17 (RWF millions) | Chicken GDP 2021/22 (RWF millions) | Change in % |
|---|---|---|---|
| ITFC meat | 13,266 | 20,475.5 | 54% |
| CFC meat | 3 | 336.8 | NA |
| **Total Family chicken meat** | 13,269 | 20,812.3 | 57% |
| ITFC Chicken eggs | 7,038 | 15,367 | 118% |
| CFC Chicken Eggs | 10 | 948 | NA |
| **Total Family chicken eggs** | 7,048 | 16,316 | 131% |
| **Total Family chicken meat and eggs** | 20,317 | 37,128 | 72% |

## *Specialized commercial chicken production (2016/17-2021/22)*

### SP Targets

Table 46: Number of chicken and chicken meat production in SP chicken subsystems (RLI)

| Chicken subsystem | **Unit** | **2016/17** | **2017/18** | **2018/19** | **2019/20** | **2020/21** | **2021/22** | % Change |
|---|---|---|---|---|---|---|---|---|
| SP layers | million | 0.77 | 0.89 | 1.02 | 1.18 | 1.35 | 1.55 | 101% |
| SP broilers | Million | 0.97 | 1.17 | 1.40 | 1.68 | 2.02 | 2.42 | 149% |
| Total SP chicken | million | 1.75 | 2.06 | 2.43 | 2.86 | 3.37 | 3.98 | 128% |
| SP layer | Tonnes | 648 | 745 | 857 | 986 | 1,134 | 1,304 | 101% |
| SP broiler | Tonnes | 9,986 | 11,983 | 14,380 | 17,256 | 20,707 | 24,848 | 149% |
| **Total** | **Tonnes** | 10,634 | 12,729 | 15,237 | 18,241 | 21,840 | 26,152 | 146% |

The number of chickens in the SP chicken layers subsystem grows from 0.77 million in 2016/17 to 1.5 million in 2021/22, a 101% increase. The SP chicken broilers subsystem grows from 0.97 million to 2.42 Million in the year 2021/22, a 149% increase.
Chicken meat production from SP chicken increases from 10,634 tonnes in 2016/17 to 26,152 tonnes in the year 2021/22, a 146% increase.

Table 47: Egg production from specialized layers (RLI)

| Chicken subsystem | **2016/17** | **2017/18** | **2018/19** | **2019/20** | **2020/21** | **2021/22** | |
|---|---|---|---|---|---|---|---|
| SP chicken egg million) | 160.3 | 184.3 | 212.0 | 243.7 | 280.3 | 322.3 | 101 % |

Egg production from specialized layers increases from 160.3 million in 2016/17 to 322.3 million in the year 2021/22, an increase of 101%.
The scale of operations or average number of commercial layers and broilers kept per farm and the number of farms will increase
Annual growth rate of layers and broilers over the coming 15 years will be 15% and 25%, respectively (Table 48).

Table 48: Commercial layer and broiler projected population at national level (RLI)

| Breed type | Base year (2016) | End of first five year (2021) | End of second five year (2026) | End of third five year (2031) |
|---|---|---|---|---|
| Layers population | 772,674 | 1,554,123 | 3,555,457 | 7,151,293 |
| Broiler population/ cycle | 974,543 | 2,424,976 | 6,034,116 | 18,414,662 |

## Key challenges and strategies related to the specialized layers and broilers system (SP system)

The specialized and family chicken systems face the same challenges as the family systems to improve and thus need the same or similar interventions to transform the industry. Refer to Table 44 above for the details **on the challenges and strategies**.

## Interventions to achieve targets

The interventions for SP chicken improvement involve increasing the number of chickens and thus the scale of operations and the volume of production from the specialized chicken farms i.e., SP chicken layers and SP chicken broilers. The major intervention proposed for the SP chicken layers and SP chicken broilers is increasing the number of birds and the number of specialized farm units using exotic pure bred chickens and modern industrial scale poultry management.

## SP chicken impacts (RLI)

### Production impacts

Chicken meat production from SP broilers increases from 10,634 tonnes in 2016/17 to 26,152 tonnes in the year 2021/22, a 146% increase.

Egg production from specialized layers increases from 160.3 million in 2016/17 to 322.3 million in the year 2021/22, an increase of 101%.

### GDP impacts

As Table 49 shows the GDP contribution of the specialized broiler system increases from the current RWF 7.2 billion to 17.3 billion (a 146% increase) and eggs from the specialized layers increases to 8.6 billion (an 101% increase) over the 5 year investment period.

Table 49: GDP contribution from commercial specialized chicken system 2016/17 and 2021/22 (RLI)

| GDP contributions | 2016/17 (RWF millions) | 2021/22 (RWF millions) | Change in % |
|---|---|---|---|
| SP Chicken meat GDP | 7,160.6 | 17,274 | 146% |
| SP Chicken eggs GDP | 4,284 | 8,617 | 101% |
| Total contribution | 11,444.60 | 25,891.00 | 126% |

The total GDP contribution of the SP chicken system (broilers and layers) increases from RWF 11.4 billion to 25.9 billion in five investment period, a 126% increase.

**Total production under the RLI scenario**

**Chicken meat**
The total chicken meat and egg production from the family and commercial specialized systems is presented in Table 50 below. It shows an increase from 15,715 tonnes in 2016/17 to 35,170 tonnes in 2021/22 for meat which constitutes a 124% increase over 5 years. The total egg production from the family and commercial specialized systems increases from 244 million to 513 million which is a 110% growth over 5 year.

Table 50: Total chicken meat and eggs production with additional investment RLI

| Products | Unit | 2016/17 | 2021/22 with case |
|---|---|---|---|
| Total chicken meat from the Family system | Tonnes | 5081 | 9018 |
| Total chicken meat from Commercial specialized system | Tonnes | 10,634 | 26,152 |
| **Total chicken meat production** | Tonnes | 15,715 | 35,170 |
| Total eggs Family system | millions | 83.4 | 190.8 |
| Total eggs from SP | millions | 160.3 | 322.3 |
| **Total eggs production** | millions | 243.7 | **513.1** |

**Total GDP**
Table 51: Total chicken meat and eggs production GDP contribution with additional investment RLI

| **Products** | **GDP 2016/17 (RWF millions)** | **GDP 2021/22 (RWF millions)** | **Change in %** |
|---|---|---|---|
| Family chicken meat and eggs contribution | 20,317 | 37,128.3 | 83% |
| Specialized chicken meat and eggs contribution | 11,444.6 | 25,890.24 | 126% |
| Total chicken GDP contribution | 31,762 | 63,019 | 98% |

Overall, with the RLI level of interventions the GDP contribution of the total chicken meat and eggs production is almost doubled, from the current RWF 31.8 billion to 63.0 billion over the 5 year investment period.

**Per capita chicken meat and eggs production RLI**
Table 52 below presents the per capita chicken meat and egg production in Rwanda comparing the baseline with 2021/22 under the additional investment scenario. Although the change in both meat and egg production is substantial, there remains a significant shortage of eggs to meet the FAO recommended requirement, which is 4.5 kg per day. This estimate is based on the projected human population in Rwanda for 2021/22, which is 13,252,272 (NISR 2014).

Table 52: Per capita availability of eggs and chicken meat RLI

| | 2016/17 | 2021/22 **"with case"** | % change |
|---|---|---|---|
| Per capita availability of eggs in kg | 1 | 1.9 | 90% |
| Per capita availability of chicken meat in kg | 1.19 | 2.65 | 123% |

The chicken investment is mainly to raise a large number of broilers and improved family chicken to produce enough chicken meat to close the gap between per capita meat production and the consumption level required to lead a healthy life.
Table 53 below shows the implications of the impact of the recommended level of investment (RLI) on production and GDP over 5 years as compared to the BAU case.

Table 53: Rwanda chicken meat production under BAU and RLI scenario (2021/22)

| **Livestock product** | **2016/17** | **2021/22 BAU** | **2021/22 With recommended case** | **% difference recommended/BAU** |
|---|---|---|---|---|
| Chicken meat in MT | 15,715 | 20,589 | 35,170 | 71% |
| Eggs in million | 243.6 | 369.7 | 513.1 | 39% |
| Chicken meat and eggs GDP in RWF | 31,762 | 43,606 | 63,019 | 45% |

As shown in table 53 above the increase in the production of chicken meat and eggs and their GDP contribution is substantial enough to justify the investment.
Table 54 below shows the required activities and implementation sequencing for the proposed interventions to transform the chicken industry, both family and specialized systems under the RLI scenario.

Table 54: Intervention activity timeline and sequencing: Gantt chart RLI

| **Investment interventions** | **Activity sequencing** | | | | |
|---|---|---|---|---|---|
| | **2017/18** | **2018/19** | **2019/20** | **2020/21** | **2021/22** |
| **Feeds interventions** | | | | | |
| Strengthen the existing feed processing plants through technology transfer, marketing extension to enable them operate at full capacity | | | | | |
| Establish 3 new chicken and pig feed processing plants each with 40,000 MT capacity | | | | | |
| Improve the capacities of chicken feed quality and biosafety (HACCP) control laboratories. | | | | | |
| Build capacity for Animal feed inspectors | | | | | |
| Land allocation for production of maize and soya beans and other raw materials for supplying the feed processing plants | | | | | |

| Investment interventions | Activity sequencing | | | | |
|---|---|---|---|---|---|
| | 2017/18 | 2018/19 | 2019/20 | 2020/21 | 2021/22 |
| Setting a procedure to facilitate importation additional raw materials in such as maize to supply the feed processing industries | | | | | |
| Build capacity of farmers on technologies and methods of formulating feed rations using locally available materials | | | | | |
| Promote the establishment of edible oil agro processing industries | | | | | |
| Demand/price and VC analysis for processed animal feed to inform feed pricing policy making and VC efficiencies | | | | | |
| **Animal Health interventions** | | | | | |
| Establish ND, FP, Gumboro vaccines production plant | | | | | |
| Establish a national bio safety guidelines for disease monitoring and controlling | | | | | |
| Strengthen the capacity for disease surveillance, quarantines and mass chicken vaccinations | | | | | |
| Rationalize public and private veterinary services | | | | | |
| Enforcing stricter disease controls on the importation of commercial replacement stock; | | | | | |
| Awareness creation among small producers on sanitation and housing | | | | | |
| **Genetics and breeding interventions** | | | | | |
| Identify and test suitable tropical dual purpose chicken crossbreeds to introduce in the family system | | | | | |
| Improve the genetic potentials of indigenous chicken breeds through selection | | | | | |
| Privatize, strengthen and upgrade the existing public chicken multiplication centers | | | | | |
| Establish 5 new private hatcheries and DOC multiplication centers with total capacities of producing 20 million | | | | | |

| Investment interventions | Activity sequencing | | | | |
|---|---|---|---|---|---|
| | 2017/18 | 2018/19 | 2019/20 | 2020/21 | 2021/22 |
| DOCs/year | | | | | |
| Establish 50 private mothering units and distribution centers for selected 4 weeks vaccinated chicken | | | | | |
| Put in place guidelines to monitor importation of DOC and fertile eggs | | | | | |
| Establish standard operating procedures of hatcheries and chicken breeder farms. | | | | | |
| Reduce reproductive wastage of brooding hens using artificial chicken brooding facilities and incubators | | | | | |
| **Marketing and extensions interventions** | | | | | |
| Establishment of private chicken slaughtering houses of a total annual capacity of slaughtering 10 million broilers along with modern chicken cutting and processing facilities and cold storage | | | | | |
| Establish 30 private municipal chicken slaughtering houses | | | | | |
| Establish one egg processing plant | | | | | |
| Promote establishment of Rwanda chicken traders associations; | | | | | |
| Build capacity and put in place strict biosafety systems and institutionalize mandatory biosafety and HACCP | | | | | |
| Promote consumption of exotic and hybrid chicken meat and egg in domestic households in Rwanda | | | | | |

Table 55:  Five-year Chicken meat and egg production improvement investment costs for RLI, the recommended investment scenario (2017/18 – 2021/22)

| **Investment interventions** | **Investment cost (000,000 or million RWF)** | | | | | **Total Budget (million RWF)** | **Budget Sources** |
|---|---|---|---|---|---|---|---|
| | **2017/18** | **2018/19** | **2019/20** | **2020/21** | **2021/22** | | |
| **Feeds interventions** | | | | | | | |
| Strengthen the existing feed processing plants through technology transfer, marketing extension to enable them operate at full capacity | 1,000 | 1,000 | | | | 2,000 | 10% public<br>90% private |
| Establish 3 new chicken feed processing plants each with 40,000 MT capacity | 1,500 | 2,500 | 2,000 | | | 5,000 | 100% private |
| Improve and upgrade chicken feed quality control laboratories. | 500 | | | | | | 20% public<br>80% private |
| Biosafety control (HACCP) and capacity building for Animal feed inspectors, and establishment of standard for chicken feed quality | 1,000 | 1,000 | | | | 2,000 | 50% public<br>50% private |
| Setting a procedure to facilitate importation of additional raw materials such as maize to supply the feed processing industries | 20 | | | | | 20 | 100% public |
| Build capacity of farmers on technologies and methods of formulating feed rations using locally available materials | 100 | 100 | | | | 200 | 60% public<br>40% private |
| Promote the establishment of edible oil agro processing industries | 50 | | | | | 50 | 50% public<br>50% private |

| Investment interventions | Investment cost (000,000 or million RWF) | | | | | Total Budget (million RWF) | Budget Sources |
|---|---|---|---|---|---|---|---|
| | 2017/18 | 2018/19 | 2019/20 | 2020/21 | 2021/22 | | |
| Demand/price and VC analysis for processed animal feed to inform feed pricing policy making and VC efficiencies | 90 | | | | | 90 | 100% public |
| **Total feed** | **4,260** | **4,600** | **1,000** | **0** | **0** | **9,860** | |
| **Animal Health interventions** | | | | | | | |
| Establish ND, FP, Gumboro vaccines production plant | 4,000 | 12,000 | | | | 16,000 | 100% public |
| Establish a national bio safety guidelines for disease monitoring and controlling | 100 | | | | | 100 | 100% public |
| Strengthen the capacity for disease surveillance, quarantines and mass chicken vaccinations | 1,500 | 1,000 | 1,000 | 1,000 | 1,000 | 5,500 | 100% public |
| Enforcing procedure and system for stricter disease controls on the importation of commercial replacement stock | 500 | 500 | | | | 1,000 | 100% public |
| Awareness creation among small producers on sanitation and housing | 100 | 100 | 100 | 100 | 100 | 500 | 60% public<br>40% private |
| **Total animal health** | 6,200 | 13,600 | 1,100 | 1,100 | 1,100 | 23,100 | |
| **Genetics and breeding interventions** | | | | | | | |
| Identify and test suitable tropical dual purpose chicken crossbreeds to introduce in the family system | 500 | 500 | | | | 1,000 | 60% public<br>40% private |

| Investment interventions | Investment cost (000,000 or million RWF) | | | | | Total Budget (million RWF) | Budget Sources |
|---|---|---|---|---|---|---|---|
| | 2017/18 | 2018/19 | 2019/20 | 2020/21 | 2021/22 | | |
| Improve the genetic potentials of indigenous chicken breeds through selection | 250 | 500 | 250 | | | 1,000 | 60% public<br>40% private |
| Privatize, strengthen and upgrade the existing public chicken multiplication center | 1,000 | 500 | | | | 1,500 | 15% public<br>85% private |
| Establish 5 new private hatcheries and DOC multiplication centers with total capacities of producing 20 million DOCs/year | 2,000 | 2,000 | 2,000 | | | 6,000 | 10% public<br>90% private |
| Establish 30 mini hatcheries | 900 | 450 | 450 | | | 1,800 | 100% private |
| Establish 50 private mothering units and distribution centers for selected 4 weeks vaccinated chicken | 220 | 660 | 220 | | | 1,100 | 100% private |
| Put in place guidelines to monitor importation of DOC and fertile eggs | 20 | | | | | 20 | 100% public |
| Establish standard operating procedures of hatcheries and chicken breeder farms. | 20 | | | | | 20 | 80% public<br>20% private |
| Reduce reproductive wastage of brooding hens using 5,000 artificial chicken brooding facilities and 3,000 artificial incubators/year | 100 | 200 | 200 | | | 500 | 10% public<br>90% private |
| **Total genetics** | **5010** | **4810** | **3120** | **0** | **0** | **12940** | |

| Investment interventions | Investment cost (000,000 or million RWF) | | | | | Total Budget (million RWF) | Budget Sources |
|---|---|---|---|---|---|---|---|
| | 2017/18 | 2018/19 | 2019/20 | 2020/21 | 2021/22 | | |
| **Marketing and extensions interventions** | | | | | | | |
| Establishment of 2 private chicken slaughtering houses of a total annual capacity of slaughtering 10 million broilers along with modern chicken cutting and processing facilities and cold storage | 1,750 | | 1,750 | | | 3500 | 100% private |
| Establish 30 private mini chicken slaughtering houses | 830 | 830 | 840 | | | 2.500 | 100% private |
| Establish one egg processing plant | | | | | 10,000 | 10,000 | 100% private |
| Promote establishment of Rwanda chicken traders associations; | 10 | 10 | | | | 20 | 100% public |
| Promote consumption of exotic and hybrid chicken meat and egg in domestic households in Rwanda | 500 | 500 | 500 | | | 1,500 | 50% public<br>50% private |
| **Total marketing** | **3,090** | **1,340** | **3,090** | **0** | **10,000** | **17,520** | |
| **Grand Total** | **18,560** | **24,350** | **8,310** | **1,100** | **11,100** | **63,420** | |

The total investment required over the first five years to implement the proposed interventions adds up to RWF 63 billion or USD 61 million. As shown in the Table 55 above the sources of the investment include both the private and public sector. The private sector invests 57% of the total required and the public sector 43%. Except the health service the other investment categories are dominantly invested by the private sector (more than 84%).This shows that the transformation of the chicken industry in Rwanda needs to be mainly driven by private sector investment.

Table 56:Total investment and recurrent costs for chicken meat and egg production VC development under RLI

| Investment category | Responsible actor (000 000 RWF) | | Total investment cost (million RWF) |
|---|---|---|---|
| | Public | Private | |
| Animal feeding | 1,555 | 8,305 | 9,860 |
| Animal health | 22,900 | 200 | 23,100 |
| Animal breeding and genetics | 2,111 | 10,829 | 12,940 |
| Marketing and value addition | 770 | 16,750 | 17,520 |
| **Total investment** | **27,336** | **36,084** | **63,420** |

Table 57: The percent contribution of public and private investments by investment areas for improving the chicken meat and egg production VC under RLI

| Key investment area | Proportion (%) by responsible actor | | Proportions of investment area |
|---|---|---|---|
| | Public | Private | |
| Animal feeding | 16% | 84% | 100% |
| Animal health | 99% | 1% | 100% |
| Animal breeding and genetics | 16% | 84% | 100% |
| Marketing and value addition | 4% | 96% | 100% |
| **Total investment** | 43% | 57% | 100% |

High production of chicken meat and eggs and value added to the national GDP is expected with the full implementation of the proposed RLI level of investment interventions. This is the recommended investment scenario in which sufficient resources are made available to transform the sector to achieve the national development objectives.

## Complimentary interventions and success requirements for Family (IFTC and CFC) and SP chicken systems

Feed is a critical factor to the success of the chicken industry. Hence, government should encourage the private sector to establish large-scale feed processing plants

Make land available for production of additional cereals for production of processed feed (maize and soybean) and facilitate importation to fill gaps

Facilitate construction of modern chicken slaughter and processing plants to process the huge increase in chicken meat production

Promote consumption of exotic chicken meat in domestic households and restaurants in Rwanda
Success in the specialized chicken (SP) and family crossbreed (CFC) systems depends on the effectiveness of the DOC production and distribution system. A well- functioning private and/or PPP DOC industry will be required for efficient production and distribution of DOCs to the specialized chicken farms and to small holders/enterprises which keep crossbreed family chicken.
Standardize feed testing labs to regulate feed quality (with periodic testing of processors compliance to set standards)
Standardize procedures for meat inspections at abattoirs, standardized mechanized slaughters, and processing facilities
Encourage private sector to invest in hatcheries and distribution of cross-bred DOCs and/or pullets,
A network which involves DOC multiplication centres, pullet out growers (mother units or hubs), individual smallholder farmers, women groups and cooperatives will be required to create a successful family chicken improvement scheme.
There is a need to put in place effective extension and animal health services (public or private) to meet the service needs (health, feed and management) of the coming millions of improved poultry family units.

## Medium Investment level scenario (MIL)

The medium investment level scenario (MLI) assumes a budget constraint to investing fully (as in the recommended case -- RLI). Only half of the RLI is made under the MLI, and the impact on production and GDP is expected to be limited. However, the total production and GDP impact would remain higher than the BAU scenario, which represents the current spending profile. See Tables 58 and 59 below.

**Key outcome assumptions for the medium investment scenario (MLI) for the family and specialized chicken systems are based on the interventions from the recommended level of investment (RLI), but scaled down to the 3 year RLI outcomes.**

The flock size in ITFC will increase from 1 to 3 improved hens
Reproductive wastage due to time spent in brooding will be reduced by 30%.
Eggs laid per hen/year will increase from 40 to 65 on average
Average weight of sold adult chicken increases from 1.4 kg to 1.55 kg.
Chicken mortality before marketing will drop from 50% to 30%.
The chickens will be supplemented with locally available feeds for up to 50% of their RLI requirements
With dual-purpose CFC the flock size is the minimum or 25 hens; with a production of 150 eggs/hen/year on average and weight at marketing of 2.5 kg
Adequate space for housing, better health services and feed supplementation is provided and delivered for dual purpose chicken
The major intervention proposed for the SP chicken layers and SP chicken broilers is increasing the number of birds and the number of specialized farm units under the 3$^{rd}$ year RLI scenario.

## Key challenges and interventions under MLI

Key challenges and interventions to achieve the targets under the MLI will remain the same as in the RLI for both family and specialized chicken systems

## Impacts under MLI scenario

Tables 58 and 59 below illustrate the production and GDP impacts under the MLI case comparing the MLI results with the base year or 2016/17 impacts and the BAU case scenario for 2021/22.

Table 58: Rwanda chicken meat production under BAU and MLI (2021/22)

| Livestock product | 2016/17 (base year) | 2021/22 BAU | 2021/22 With MLI case | % difference MLI/base year | % difference MLI/BAU |
|---|---|---|---|---|---|
| Chicken meat in MT | 15,715 | 20,589 | 27,826 | 77% | 35% |
| Eggs in million | 243.6 | 369.7 | 433.5 | 78% | 17% |

Table 59: Rwanda chicken meat value added or GDP impacts under BAU and MLI (2021/22)

| Livestock GDP | 2016/17 (base year) | 2021/22 BAU | 2021/22 With MLI case | % difference MLI/base year | % difference MLI/BAU |
|---|---|---|---|---|---|
| Chicken meat in million RWF | 20,430 | 27,278 | 31,284 | 53% | 15% |
| Eggs in million RWF | 11,332 | 16,328 | 19,090 | 68% | 17% |
| Total | 31,762 | 43,606 | 50,374 | 59% | 16% |

As shown in Tables 58 and 59 above, the chicken meat and eggs production and the value added by the sub sector under the MLI scenario is substantial when compared to the base year 2016/17 situation.  However, the chicken meat and eggs production shows an increase of 35 and 17% respectively compared to the BAU 2021/22 scenario. The total value added also increases by 16% compared to the BAU scenario. Although, there are some increases in production and value added, it is significantly far below what could be achieved under the recommended RLI scenario. The MLI impact on production and value added is not big enough to recommend MLI outright as the preferred investment. Hence, it requires further analysis to compute the Benefit-Cost ratio and the NPV to justify or reject the MLI scenario for chicken.

Table 60: Intervention activity timeline and sequencing: Gantt chart for MLI scenario

| Investment interventions | Activity sequencing | | | | |
|---|---|---|---|---|---|
| | 2017/18 | 2018/19 | 2019/20 | 2020/21 | 2021/22 |
| **Feeds interventions** | | | | | |
| Strengthen the existing feed processing plants through technology transfer, marketing extension to enable them operate at full capacity | | | | | |
| Establish 1 new chicken and pig feed processing plants each with 40,000 MT capacity | | | | | |
| Improve the capacities of chicken feed quality and biosafety (HACCP) control laboratories. | | | | | |
| Build capacity for Animal feed inspectors | | | | | |
| Land allocation for production of maize and soya beans and other raw materials for supplying the feed processing plants | | | | | |
| Setting a procedure to facilitate importation additional raw materials in such as maize to supply the feed processing industries | | | | | |
| Build capacity of farmers on technologies and methods of formulating feed rations using locally available materials | | | | | |
| Promote the establishment of edible oil agro processing industries | | | | | |
| Demand/price and VC analysis for processed animal feed to inform feed pricing policy making and VC efficiencies | | | | | |
| **Animal Health interventions** | | | | | |
| Establish ND, FP, Gumboro vaccines production plant | | | | | |
| Establish a national bio safety guidelines for disease monitoring and controlling | | | | | |
| Strengthen the capacity for disease surveillance, quarantines and mass chicken vaccinations | | | | | |
| Rationalize public and private veterinary services | | | | | |
| Enforcing stricter disease controls on the importation of commercial replacement | | | | | |

| Investment interventions | Activity sequencing | | | | |
|---|---|---|---|---|---|
| | 2017/18 | 2018/19 | 2019/20 | 2020/21 | 2021/22 |
| stock; | | | | | |
| Awareness creation among small producers on sanitation and housing | | | | | |
| **Genetics and breeding interventions** | | | | | |
| Identify and test suitable tropical dual purpose chicken crossbreeds to introduce in the family system | | | | | |
| Improve the genetic potentials of indigenous chicken breeds through selection | | | | | |
| Privatize, strengthen and upgrade the existing public chicken multiplication centers | | | | | |
| Establish 3 new private hatcheries and DOC multiplication centers with total capacities of producing 20 million DOCs/year | | | | | |
| Establish 15 mini hatcheries and 25 private mothering units and distribution centers for selected 4 weeks vaccinated chicken | | | | | |
| Put in place guidelines to monitor importation of DOC and fertile eggs | | | | | |
| Establish standard operating procedures of hatcheries and chicken breeder farms. | | | | | |
| Reduce reproductive wastage of brooding hens using artificial chicken brooding facilities and incubators | | | | | |
| **Marketing and extensions interventions** | | | | | |
| Establishment of 1 private chicken slaughtering house of a total annual capacity of slaughtering 10 million broilers along with modern chicken cutting and processing facilities and cold storage | | | | | |
| Establish 15 private municipal chicken slaughtering houses | | | | | |
| Promote establishment of Rwanda chicken traders associations; | | | | | |
| Build capacity and put in place strict biosafety systems and institutionalize | | | | | |

| Investment interventions | Activity sequencing | | | | |
| --- | --- | --- | --- | --- | --- |
| | 2017/18 | 2018/19 | 2019/20 | 2020/21 | 2021/22 |
| mandatory biosafety and HACCP | | | | | |
| Promote consumption of exotic and hybrid chicken meat and egg in domestic households in Rwanda | | | | | |

Table 61: Five-year Chicken meat and egg production improvement investment costs under medium investment scenario (2017/18 – 2021/22)

| **Investment interventions** | **Investment cost (000,000 RWF)** | | | | | **Total Budget** | **Budget Sources** |
|---|---|---|---|---|---|---|---|
| | **2017/18** | **2018/19** | **2019/20** | **2020/21** | **2021/22** | | |
| **Feeds interventions** | | | | | | | |
| Strengthen the existing feed processing plants through technology transfer, marketing extension to enable them operate at full capacity | 1,000 | 1,000 | | | | 2,000 | 10% public<br>90% private |
| Establish 1 new chicken feed processing plants each with 40,000 MT capacity | 2,000 | | | | | 2,000 | 100% private |
| Improve and upgrade chicken feed quality control laboratories. | 250 | | | | | 250 | 20% public<br>80% private |
| Biosafety control (HACCP) and capacity building for Animal feed inspectors, and establishment of standard for chicken feed quality | 1,000 | | | | | 1,000 | 50% public<br>50% private |
| Setting a procedure to facilitate importation of additional raw materials such as maize to supply the feed processing industries | 20 | | | | | 20 | 100% public |
| Build capacity of farmers on technologies and methods of formulating feed rations using locally available materials | 100 | | | | | 100 | 60% public<br>40% private |
| Promote the establishment of edible oil agro processing industries | 50 | | | | | 50 | 50% public<br>50% private |
| Demand/price and VC analysis for processed animal feed to inform feed pricing policy making and VC efficiencies | 90 | | | | | 90 | 100% public |
| **Total feed** | **4,510** | **1,000** | | | | **5,510** | |
| **Animal Health interventions** | | | | | | | |

| Investment interventions | Investment cost (000,000 RWF) | | | | | Total Budget | Budget Sources |
|---|---|---|---|---|---|---|---|
| | 2017/18 | 2018/19 | 2019/20 | 2020/21 | 2021/22 | | |
| Establish ND, FP, Gumboro vaccines production plant | 4,000 | | | | | 4000 | 100% public |
| Establish a national bio safety guidelines for disease monitoring and controlling | 100 | | | | | 100 | 100% public |
| Strengthen the capacity for disease surveillance, quarantines and mass chicken vaccinations | 1,500 | 1,000 | | | | 2,500 | 100% public |
| Enforcing procedure and system for stricter disease controls on the importation of commercial replacement stock | 500 | | | | | 500 | 100% public |
| Awareness creation among small producers on sanitation and housing | 100 | 100 | 100 | | | 300 | 60% public<br>40% private |
| **Total animal health** | **6,200** | **2,100** | **100** | | | **8,400** | |
| **Genetics and breeding interventions** | | | | | | | |
| Identify and test suitable tropical dual purpose chicken crossbreeds to introduce in the family system | 500 | | | | | 500 | 60% public<br>40% private |
| Improve the genetic potentials of indigenous chicken breeds through selection | 500 | | | | | 500 | 60% public<br>40% private |
| Privatize, strengthen and upgrade the existing public chicken multiplication center | 1,000 | | | | | 1,000 | 15% public<br>85% private |
| Establish 3 new private hatcheries and DOC multiplication centers with total capacities of producing 20 million DOCs/year | 2,000 | 2000 | | | | 4000 | 10% public<br>90% private |
| Establish 15 mini hatcheries | 900 | | | | | 900 | 100% private |
| Establish 25 private mothering units and distribution centers for selected 4 weeks | 220 | 330 | | | | 550 | 100% private |

| Investment interventions | Investment cost (000,000 RWF) | | | | | Total Budget | Budget Sources |
|---|---|---|---|---|---|---|---|
| | 2017/18 | 2018/19 | 2019/20 | 2020/21 | 2021/22 | | |
| vaccinated chicken | | | | | | | |
| Put in place guidelines to monitor importation of DOC and fertile eggs | 20 | | | | | 20 | 100% public |
| Establish standard operating procedures of hatcheries and chicken breeder farms. | 20 | | | | | 20 | 80% public<br>20% private |
| Reduce reproductive wastage of brooding hens using 25000 artificial chicken brooding facilities and 1500 artificial incubators/year | 100 | 150 | | | | 250 | 10% public<br>90% private |
| **Total genetics** | **5,260** | **2480** | **0** | **0** | **0** | **7,740** | |
| **Marketing and extensions interventions** | | | | | | | |
| Establishment of 1 private chicken slaughtering houses of a total annual capacity of slaughtering 10 million broilers along with modern chicken cutting and processing facilities and cold storage | 1,750 | | | | | 1750 | 100% private |
| Establish 15 private mini chicken slaughtering houses | 830 | 295 | | | | 1125 | 100% private |
| Establish one egg processing plant | | | | | | | 100% private |
| Promote establishment of Rwanda chicken traders associations; | 10 | 10 | | | | 20 | 100% public |
| Promote consumption of exotic and hybrid chicken meat and egg in domestic households in Rwanda | 500 | 250 | | | | 750 | 50% public<br>50% private |
| **Total marketing** | **3,090** | **555** | | | | **3,645** | |
| **Total investment** | **19,060** | **3,135** | **100** | | | **25,295** | |

As shown above in Table 61 above, in the MLI scenario the total investment will come down to RWF 25 billion or USD 30 million compared to RWF 51 billion or USD 61 million in the RLI scenario. Similar to the RLI, the sources of investment in the MLI case remain to be heavily private with a significant public contributions.

Complimentary interventions and success requirements for the MLI scenario are the same as for the RLI scenario.

## *Conclusions*

The chicken meat and egg production under the RLI scenario show substantial increments, 124% and 110% respectively compared to the base year. The RLI scenario would contribute significantly to fulfilling the national all meat requirement and would increase per capita egg consumption for Rwanda to help meet one of the Rwandan national development objectives of improving food and nutrition security. Under the RLI scenario, although the per capita egg consumption increases, a significant shortage continues to exist to meeting the FAO recommended requirement, which is 4.5 kg per person.

It must also be noted that the technical interventions alone will not bring the projected output unless the policy and institutional challenges are addressed.

Needed policies and strategies **–** to be enacted and implemented

- Livestock products pricing policy
- Animal breeding strategy
- Livestock identification & reporting system policy
- Livestock animal and product traceability policy

Regulations and standards

- Regulation of animal breeding
- Regulation of animal nutrition; standards on animal commercial feeds, crops residues, formulation of the feeds, honey, etc
- Regulation of livestock products pricing and standards on animal products grading (milk, meat, honey, eggs, wool, hides and skins)
- Standards on livestock infrastructure development (slaughterhouses, laboratories, processing factories, etc.)
- Standards on vet services and pharmaceuticals and supplies

Institutional framework

- Strengthening the functional linkages and collaboration of institutions from national level to grassroots

Under the MLI scenario, chicken meat and eggs is less by 21 and 16% respectively compared to the RLI. This would hinder the development effort of Rwanda to transform the sub-sector, postpone the achievement of closing the production and consumption gap for all meat and postpone increasing the per capita egg consumption of Rwandans.

Table 62: MLI and RLI production and GDP comparison

| Scenario Outcomes | Investment Scenarios | | | % difference MLI to RLI |
|---|---|---|---|---|
| | **BAU** | **RLI** | **MLI** | |
| Production 5th year Meat in MT | 20,589 | 35,170 | 27,826 | -21% |
| Eggs in million number | 369.7 | 513.1 | 433.5 | -16% |
| GDP 5th year | 43,606 | 63,019 | 50,374 | -20% |

The difference in the GDP contribution of the sub-sector between the BAU and MLI is only 16%. Moreover, compared to the RLI, the MLI shows a 20% drop in GDP contribution. It is recommended to do a financial feasibility of the MLI scenario before committing resources to this investment option.

# Pork value chain development roadmap

(2017/18-2021/22)

# Pork value chain development roadmap (2017/18-2021/22)

## *Vision*

The Rwanda pig industry becomes an efficiently functioning sub-sector with highly market-oriented farming and processing and dynamic marketing, to supply consumers with high quality and safe pig meat/pork, while operating in more sustainable ways, and contributing towards closing the national gap in meat production and consumption, improving household food and nutritional security, achieving income growth and poverty alleviation and contributing to growth in national income.

**Investment scenarios analyzed**

Three scenarios corresponding to three levels of investment in the pig sub-sector are examined for their impacts on production of pork meat and contributions to GDP to provide three investment choices for decision makers in Rwanda: the government, private investors and development partners (donors). These three scenarios are referred as BAU (Business As Usual), MLI (Medium Level of Investment) and RLI (Recommended Level of Investment). The BAU scenario analyzes the base case or presents the implications of the current level of investment for pig meat production and GDP contributions during the 5-year LMP roadmap period. RLI is the level of investment which could achieve the 5-year targets for the agreed upon national development objectives. In the MLI scenario the assumed level of investment is that of the RLI through the 3rd year of the 5-year LMP period (see Table 63 below) and could achieve the 3rd year RLI impact on the targets for pork production and contribution to GDP. The MLI scenario is tested to provide investors with the choice of a medium level of investment given they face an assumed budget constraint.

## *Overall Target under the recommended level of investment (RLI) scenario*

The overall target under RLI is to raise pig meat production from the current 19,945 tonnes in 2016/17 to 67,076 tonnes by 2021/22, an increase of 239% -- to be achieved through improving the family mixed pig system (IFP) and expanded commercial specialized piglet fattening (CSP).

## *Modernizing the semi-intensive mixed family pig production system (IFP)*

### Targets

Table 63: Number of sows and tonnes of pork in improved family mixed (IFP) pig subsystems (RLI scenario)

| Pig system | Unit | 2016/17 | 2017/18 | 2018/19 | 2019/20 | 2020/21 | 2021/22 | % Change |
|---|---|---|---|---|---|---|---|---|
| Semi intensive family mixed system (IFP) | Number | 252,960 | 278,256 | 306,081 | 336,689 | 370,358 | 407,394 | 61% |
| Semi intensive family mixed system (IFP) | Tonnes | 19,869 | 21,260 | 22,749 | 24,342 | 26,047 | 27,871 | 40% |

Source: LSIPT Livestock Sector Analysis (2017), Rwanda

Number of sows in family mixed (IFP) systems increases from 252,960 to 407,394, a 61% increase.

Pig meat production from the family mixed (IFP) systems increases from 19,869 tonnes to 27,871 tonnes a 40% increase.

## Key assumptions for improving the family pig sub-system under the RLI scenario

The number of sows per farm will increase from the current 1 to 6

Age at first service (in days) to decrease from 330 days to 250 days

Mortality rate of young animals before weaning to decrease from 13% to 10%

Mortality rate from weaning to marketing to decrease from 4% to 3%

Adult mortality rates to decrease from 3% to 2%

Cost of veterinary services to double

Percent of feed purchased to increase from the current 30% to 50%

Table 64: Key Challenges and Interventions related to the recommended level of investment (RLI) to improve family pig system (IFP)

| Challenges | Strategies to address the challenges |
|---|---|
| **Genetics** | |
| Lack of specialized commercial pig breeding farms to adequately supply well-bred high yielding pig stocks to pig farms | Establishing public and private sector specialized commercial pig breeding and multiplication farms with efficient distribution services |
| Low productivity of pigs in the family mixed system partly due to low genetic potential and inbreeding. | Importing new lines of improved high yielding pig breeds to avoid inbreeding and increase productivity<br>Extension and proper management and husbandry practices to lower the probability of inbreeding |
| **Animal Health** | |
| ASF is a permanent risk that could cause heavy mortality losses. FMD is also a serious health problem. | Strengthening surveillance, early detection/diagnosis<br>Pig Biosecurity policy guidelines for farmers (small and commercial), pig feed and meat processors<br>Support immunization measures FMD<br>Improving pig farm management practices to benefit producers.<br>Put in place an effective biosafety measures (HACCP)<br>Extensive pig farmers education on pig husbandry and health management |
| **Feeds challenges** | |

| Challenges | Strategies to address the challenges |
|---|---|
| Lack of supply of commercial processed feeds and other feeds | Establishment of private feed mills and feed processing plants<br>Expand private cereals and legumes production to supply feed processing plants.<br>Conduct research on alternative pig feeds to reduce completion of cereals for human consumption.<br>Build capacities of farmers to prepare feed ration using locally available materials |
| **Marketing and Processing challenges** | |
| Weak pig marketing arrangements | Developing the pig value chain to improve pig marketing, trading capacity and smallholder pig production by constructing pig markets, slaughter facilities/abattoirs and fresh pork marketing outlets<br>Strengthening Swine Producer Associations to innovate and actively participate in the value chain |
| lack of pig slaughter facilities/abattoirs, absence of cooling systems | |
| **Policy challenges** | |
| Lack of official pig marketing, transporting policies | Ensure Policy guidelines/regulations to organize pig marketing/trading system developed |
| Lack of policy for pig holding and slaughtering facilities | Create enabling policy environments for establishment of rural small-scale and urban large-scale slaughter facilities<br>Develop policy guidelines, standard operating procedures (SOP) and awareness **campaigns on slaughter facilities' hygiene** and food safety |

## Interventions to achieve targets in IFP systems

The family mixed system will be transformed with introduction of improved breeds, better animal health services and feeding. The semi intensive piglet fattening system is in its initial stage of development and will be scaled up by overcoming the breed, health, marketing and most importantly the feed problems. The genetic improvement involves import of adapted tropical and productive pig sows and boars for breeding and crossbredding and establishment of pig breeding and multiplication farms. The animal health intervention involves strengthening disease control, targeting the control and prevention of priority pig diseases (ASF, FMD)). Strengthening the biosecurity at farm level and at processing facilities is essential. The feed intervention involves strengthening capacity of private small-scale pig feed mills/processors with distribution networks to compound and distribute pig feeds to rural small-holder farmers and also strengthening the capacity of family pig keeping households to compound feed and supplement their herds with home formulated rations made from locally available feed materials.

### Impacts under RLI scenario

**Production and GDP impacts (IFP)**

As a result of the additional recommended level of investment, the total pig meat production from the family system (IFP) increases from 19,869 to 27,871 tonnes, a 40% increase, the GDP contribution of IFP increases from RWF 21,026 million in 2016/17 to RWF 41,877 million in 2021/22 which constitutes an increase of 99%.

Table 65: Pig meat production and GDP contribution family system (RLI)

| Family mixed pig system (IFP) | 2016/17 | 2021/22 | Change in % |
|---|---|---|---|
| Total Pig meat from the IFP (tonnes) | 19,869 | 27,871 | 40% |
| GDP contribution by the IFP in RWF | 21,026 | 41,877 | 99% |

## *Expanding Commercial Specialized Piglet fattening systems (CSP) RLI scenario*

In this operation piglets are fattened for the pork market. This system is in its infant stage. It is a newly introduced system which is expected to increase pig meat production greatly over time.

### Targets

Table 66: Population growth of piglets fattening and meat production RLI

| Pig subsystem | 2016/17 | 2017/18 | 2018/19 | 2019/20 | 2020/21 | 2021/22 | % change |
|---|---|---|---|---|---|---|---|
| Piglet numbers | 507 | 1,774 | 6,210 | 21,735 | 76,072 | 266,251 | NA |
| Piglet meat (tonnes) | 76 | 265 | 928 | 3,249 | 11,373 | 39,805 | NA |

**Source: LSIPT Livestock Sector Analysis (2016), Rwanda**

Number of fattened piglets in the commercial specialized pig fattening subsystem grows to 266,251.

Pig meat production from the commercial specialized pig fattening system reaches to 39,805 tonnes over 5 year period.

### Key assumption RLI

The number of pigs in the commercial specialized pig fattening system (CSP) will increase from about zero at the base year to about 266 thousand in year 5 and the number of piglet fattening farm units and piglet per unit farms will increase.

Health service and biosafety procedures will be established and fully functioning to control hazards and disease outbreaks such as ASF.

### Key Challenges and Strategies related to the commercial specialized pig (CSP) system under RLI

The key challenges and strategies related to commercial specialize pig (CSP) system is the same as for the family system (IFP)

## Interventions to achieve targets in commercial specialized family pig (CSP) systems RLI

The feed, health, genetics, marketing and policy interventions proposed for the family system also hold for the specialized piglet fattening system. Moreover, the total number of piglets and fattening units in the piglet fattening (CSP) system will be increased. A stricter biosafety system and HACCP will also be put in place along the value chain.

## Impacts under RLI

### Production impacts

The meat production from the piglet fattening reaches to 39,805 tons over 5 year period. Piglet fattening is a newly tested pork production system that barely exist in Rwanda currently. Piglet fattening farmers will buy piglets either as weaners or growers and fatten them for the domestic as well as export pork market

### GDP impacts

The GDP contribution of CSPS increases from the current RWF 46 million to 24,389 million in 5 year time.

### Total Pig Meat Production under RLI

The total pig meat production from the family and specialized piglet fattening systems increases by 239% over the 5 year period (2017 – 2022). This would be attributed to good animal productivity and higher production and the increasing number of intensive smallholder, medium-to-large scale farmers.

Table 67: Total pig meat production under RLI

| Products | Pig meat 2016/17 (in tonnes) | Pig meat 2021/22 (in tonnes) | Change in % |
|---|---|---|---|
| Total Pig meat from the Family mixed system | 19,869 | 27,871 | 40% |
| Total pig meat from Commercial specialized piglet fattening system | 76 | 39,805 | NA |
| Total Pig meat production | 19,945.00 | 67,676.00 | 239% |

**Source: LSIPT Livestock Sector Analysis (2016), Rwanda.**

### Total GDP contribution

Overall the GDP contribution of the total pig meat production could increase from RWF 21 billion to 66 billion in the 5-year LMP period, an increase of 214% from the base year contribution.

Table 68: Total GDP contribution Family (IFP) and Commercial/Specialized Pig (CSP) under RLI

| Products | Pig meat GDP 2016/17 (RWF millions) | Pig meat GDP 2021/22 (RWF millions) | Change in % |
|---|---|---|---|
| Family Pig meat contribution | 21,026 | 41,877 | 99% |
| Specialized pig meat contribution | 46 | 24,389 | NA |
| Total meat contribution | 21,072 | 66,266 | 214% |

**Source: LSIPT Livestock Sector Analysis (2016) Rwanda**

**Per capita pig meat production under RLI**

As the result of the additional RLI investment in the sector, the per capita pig meat production in Rwanda would increase from the current 1.5 kg to 5 kg. This represents a significant accomplishment made possible by the transformation of the sector in the 5-year LMP period.

Table 69: Activity Sequencing (Gantt chart) under RLI

| | **Type of investment** | **2017** | **2018** | **2019** | **2020** | **2021** |
|---|---|---|---|---|---|---|
| 1.0 | Animal Feeding | | | | | |
| 1.1 | Establish private pig feed processing plant | | | | | |
| 1.2 | Establishment of 10 private small-scale feed mills | | | | | |
| 1.3 | Expand private massive cereals and legumes production to supply feed processing plants | | | | | |
| 1.4 | Research on alternative pig feeds in terms of nutritive values, and feed conversion impacts on weight gain and meat quality | | | | | |
| 1.5 | Capacity building of family mixed pig farmers to formulate their own feed ration using locally available materials | | | | | |
| 2.0 | Animal Health | | | | | |
| 2.1 | Strengthen disease surveillance capacity | | | | | |
| 2.2 | Strength vet labs in knowledge, technologies, methods and facilities to test for early diagnosis of key pig diseases especially ASF | | | | | |
| 2.3 | Develop and strengthen capacity to control AFSV and transform the pig industry in the country | | | | | |
| 3.0 | Animal Breeding and Genetics | | | | | |
| 3.1 | Research on tropical adaptable productive pig sow and boar breeds | | | | | |
| 3.2 | Facilitate to establish private and public pig breeder and multiplication farms | | | | | |
| 3.3 | Establish Swine breeders Associations and build their capacity | | | | | |
| 4.0 | Marketing and Value Additions | | | | | |
| 4.1 | Establish new specialized piglet fattening farms | | | | | |
| 4.2 | Build capacity of Meat Inspectors | | | | | |
| 4.3 | Construct 30 pig marketing centers with slaughter facilities | | | | | |
| 4.4 | Construct 2 mechanized pig slaughters, processing plant, with cold-storage for marketing of chilled pig meat to domestic and exports markets | | | | | |
| 4.5 | Facilitate pig identification, registration and traceability | | | | | |
| 5.0 | Policy | | | | | |
| 5.1 | Develop official pig marketing, transporting policies and pig holding and slaughtering policy | | | | | |

Table 70: Five year pig/pork production improvement recommended level of investment costs scenario (2017/18 – 2021/22) RLI

| | Type of investment | Investment cost (RWF, 000,000) | | | | | TOTAL (RWF ,000,000) | Budget Source |
|---|---|---|---|---|---|---|---|---|
| | | 2017/18 | 2018/19 | 2019/20 | 2020/21 | 2021/22 | | |
| **1.0** | **Genetics** | | | | | | | |
| 1.1 | Establish 5 private pig breeding and multiplication farms | 100 | 200 | 100 | 100 | | 500 | Public 15% PPP 85% |
| 1.2 | Establish 5 public pig breeding and multiplication farms | 100 | 100 | 100 | 100 | 100 | 500 | Public 100% |
| | **Total genetics** | **200** | **300** | **200** | **200** | **100** | **1000** | |
| **2.0** | **Animal feed** | | | | | | | |
| 2.1 | Establish private chicken and pig feed processing plant | | | | | | | See chicken feed budget |
| 2.2 | Establishment of 10 private small-scale feed mills | 375 | | 375 | | | 750 | Private 100% |
| 2.3 | Expand private massive cereals and legumes production to supply feed processing plants | | | | | | | See chicken feed budget |
| 2.4 | Research on alternative pig feeds in terms of nutritive values, and feed conversion impacts on weight gain and meat quality | 200 | 200 | | | | 400 | 20%private & 80% public |
| | **Total animal feed** | **575** | **200** | **375** | | | **1150** | |
| **3.0** | **Animal health** | | | | | | | **See chicken AH budget** |
| | **Marketing** | | | | | | | |
| 3.1 | Pig identification, registration and traceability | 500 | 500 | | | | 1000 | Public 80% Private 20% |
| 3.2 | Establish 2 large-scale specialized investors' pig farms | 1,200 | | 1,000 | | | 2200 | Public 5% Private 95% |
| 3.3 | Establish 30 new commercial specialized pig farms for commercial pig production | 500 | 1000 | 1000 | 500 | | 3,000 | Public 5% Private 95% |
| 3.4 | Construct 30 pig marketing centers with slaughter facilities | 1000 | 1000 | 1000 | | | 3,000 | Public 10% Private 90% |

| | Type of investment | Investment cost (RWF, 000,000) | | | | | TOTAL (RWF ,000,000) | Budget Source |
|---|---|---|---|---|---|---|---|---|
| | | 2017/18 | 2018/19 | 2019/20 | 2020/21 | 2021/22 | | |
| 3.5 | Construct mechanized pig slaughters, processing plant, with cold-storage for marketing of chilled pig meat to domestic and exports markets | 500 | 1,500 | | | | 2,000 | Public 5%<br>Private 95% |
| | **TOTAL** | **4475** | **4500** | **3575** | **700** | **100** | **13,350** | **Pubic 18%**<br>**Private 82%** |

The potential high production increase and value added in the pig sub sector will depend on realizing the recommended level of investment and building adequate human and institutional capacity and political will to facilitate the implementation of the proposed interventions to transform the sector. The total cost of this investment situation which is characterized as the recommended level of investment (RLI) scenario adds up to RWF 13.4 billion in which 84% comes from the private sector and the remaining 16% from public sources, including Development Partners (DPs) (Tables 71 and 72).

Table 71:Total investment and recurrent costs for pig production VC development under RLI

| **Investment category** | **Responsible actor (000 000 RWF)** | | **Total investment cost (million RWF)** |
|---|---|---|---|
| | **Public** | **Private** | |
| Animal feeding | 80 | 1070 | 1150 |
| Animal breeding and genetics | **575** | **425** | 1000 |
| Marketing and value addition | **1460** | **9740** | 11200 |
| **Total investment** | 2115 | 11235 | 13350 |

Table 72: The percent contribution of public and private investments by investment areas for improving the pig production VC under RLI

| **Key investment area** | **Proportion (%) by responsible actor** | | **Proportions of investment area** |
|---|---|---|---|
| | **Public** | **Private** | |
| Animal feeding | 7% | 93% | 100% |
| Animal breeding and genetics | 58% | 43% | 100% |
| Marketing and value addition | 13% | 87% | 100% |
| **Total investment** | 16% | 84% | 100% |

## Complimentary interventions and success requirements for Family (IFP) and CSP pig production under the RLI scenario

Pig meat is the highest produced and consumed animal source food (ASF) in the world. It could play a major role in the future to help Rwanda meet the ASF requirements of its growing population. The following complimentary requirements, however, need to be met to ensure the effectiveness of the proposed LMP Pig roadmap interventions to help achieve sustainable transformation of the sub sector.

- Supplementary feeding increased in the family mixed system and achieved through adequate production and supply of quality commercial feeds
- Largescale private investment in massive cereal production under irrigation incentivized to ensure adequate feed input supply to more privately owned feed mills

- More efficient and effective biosafety, animal health services and productivity enhancement breeding services targeting the family mixed system and specialized commercial CSP production units
- Incentivizing the private sector to invest in large and medium scale specialized pig farming if they create demonstration effects and increase access to inputs for IFP farmers to modernize their pig operations
- Incentivizing the private sector to invest in pig slaughter houses and pork product processing plants if they provide access to private extension training and inputs for smallholders -- especially more and better feed, vet services, and genetics
- Strengthen pig producer and trader associations to assure better access to inputs and output markets

### Medium Level Investment (MLI) Scenario

If only half of the investment funds which are required for RLI are invested in the Pig sub sector, then the investment impact on production and GDP is expected to be somewhat limited. This scenario is called the medium level investment or MLI (see section 1.2). In the MLI scenario analysis carried out to produce the LMP Pig roadmap, the MLI is simulated by assuming the level of investment of the RLI through the 3rd year of the 5-year LMP period and the impact is based on production and contribution to GDP that could be achieved with this level of investment. Table 73 below shows the expected impact on production and GDP contribution of the sector under the medium investment level (MLI) scenario, as in the case of the RLI scenario the MLI results are evaluated by making comparisons with the BAU investment scenario, but also with the RLI results.
Under medium investment level scenario it is assumed that there will be a budget constraint to invest fully (as in the recommended case) and only half of the RLI would be made available to improve the sub sector.

### Key assumptions for improving the family pig sub-system (IFP) under the MLI scenario

- Under the medium investment level (MLI) scenario it is assumed that there will be a budget constraint to invest fully (as in the recommended investment level scenario) and only half of the RLI would be made available to improve the sub sector.
- The number of sows per farm will increase from the current 1 to 3
- Age at first service (days) to decrease from 330 days to 250 days
- Mortality rate of young animals before weaning to decrease from 13% to 10%
- Mortality rate for weaning to marketing to decrease from 4 to 3%
- Adult mortality rates to decrease from 3 to 2%
- Cost of veterinary service to double

Percent of feed purchased to increase from the current 30% in the semi-intensive family piggery to 40%

### Key challenges and interventions under MLI

Key challenges and interventions are the same as in the RLI scenario

### Interventions to achieve targets under the MLI

Interventions to achieve targets under the MLI are the same as in the RLI for both family and specialized pig systems

### Impacts under the MLI scenario

Table 73 below shows the impacts of the medium level investment (MLI) scenario on production and contribution to GDP.

The MLI impact on production and GDP is much less than in the RLI scenario (see Tables 71, 72, 73 and 74). The total production and GDP impact would remain significantly higher than the baseline year, i.e., 2016/17. Nevertheless, it shows 70% increment in pig meat production and 76% increment in GDP compared to the BAU scenario, which represents the current spending level.

Table 73: Rwanda total pig meat production and GDP contribution under BAU and MLI scenario (2021/22)

| Rwanda pig meat production and GDP | | | | | |
|---|---|---|---|---|---|
| Livestock product | 2016/17 Baseline | 2021/22 BAU | 2021/22 With MLI | % difference MLI/Baseline | % difference MLI/BAU |
| Pig meat in MT | 19,945 | 26,665 | 45,461 | 128% | 70% |
| GDP from Pig meat in RWF (million) | 21,072 | 28,184 | 49,659 | 136% | 76% |

Table 74 below shows the time sequencing in the implementation of the different intervention activities under the MLI scenario. The major intervention activities, feed, genetics, health, marketing... remain the same, but there would be changes in magnitude and timing of implementation.

Table 74: Activity sequencing Gantt chart under MLI

| | **Type of investment** | **2017** | **2018** | **2019** | **2020** | **2021** |
|---|---|---|---|---|---|---|
| **1.0** | **Animal Feeding** | | | | | |
| 1.1 | Establish private pig & chicken feed processing plant | | | | | |
| 1.2 | Establishment of 5 private small-scale feed mills | | | | | |
| 1.3 | Expand private massive cereals and legumes production to supply feed processing plants | | | | | |
| 1.4 | Research on alternative pig feeds in terms of nutritive values, and feed conversion impacts on weight gain and | | | | | |

| | Type of investment | 2017 | 2018 | 2019 | 2020 | 2021 |
|---|---|---|---|---|---|---|
| | meat quality | | | | | |
| 1.5 | Capacity building of family mixed pig farmers to formulate their own feed ration using locally available materials | | | | | |
| **2.0** | **Animal Health** | | | | | |
| 2.1 | Strengthen disease surveillance capacity | | | | | |
| 2.2 | Strength vet labs in knowledge, technologies, methods and facilities to test for early diagnosis of key pig diseases especially ASF | | | | | |
| 2.3 | Develop and strengthen capacity to control AFSV and transform the pig industry in the country | | | | | |
| **3.0** | **Animal Breeding and Genetics** | | | | | |
| 3.1 | Research on tropical adaptable productive pig sow and boar breeds | | | | | |
| 3.2 | Facilitate to establish private and public pig breeder and multiplication farms | | | | | |
| 3.3 | Establish Swine breeders Associations and build their capacity | | | | | |
| **4.0** | **Marketing and Value Additions** | | | | | |
| 4.1 | Establish new specialized piglet fattening farms | | | | | |
| 4.2 | Build capacity of Meat Inspectors | | | | | |
| 4.3 | Construct 15 pig marketing centers with slaughter facilities | | | | | |
| 4.4 | Construct 1 mechanized pig slaughters, processing plant, with cold-storage for marketing of chilled pig meat to domestic and exports markets | | | | | |
| 4.5 | Facilitate pig identification, registration and traceability | | | | | |
| **5.0** | **Policy** | | | | | |
| 5.1 | Develop official pig marketing and transporting policies and pig holding and slaughtering policy | | | | | |

Table 75: Five year pig/pork production improvement investment costs under medium investment scenario (2017/18 – 2021/22) MLI

| | Type of investment | Investment cost (RWF, 000,000) | | | | | TOTAL (RWF ,000,000) | Budget Source |
|---|---|---|---|---|---|---|---|---|
| | | 2017/18 | 2018/19 | 2019/20 | 2020/21 | 2021/22 | | |
| **1.0** | **Genetics** | | | | | | | |
| 1.1 | Establish 3 private pig breeding and multiplication farms | 100 | 200 | | | | 300 | Public 15% Private 85% |
| 1.2 | Establish 2 public pig breeding and multiplication farms | 100 | 100 | | | | 200 | Public 100% |
| | | **200** | **300** | | | | **500** | |
| **2.0** | **Animal feed** | | | | | | | |
| 2.1 | Establish private chicken and pig feed processing plant | | | | | | | See chicken feed budget |
| 2.2 | Establishment of 5 private small-scale feed mills | 180 | 195 | | | | 375 | Private 100% |
| 2.3 | Expand private massive cereals and legumes production to supply feed processing plants | | | | | | | See chicken feed budget |
| 2.4 | Research on alternative pig feeds | 200 | 200 | | | | 400 | 20% private & 80% public |
| | **Total animal feed** | **380** | **395** | | | | **775** | |
| **3.0** | **Animal health** | | | | | | | **See chicken health budget** |
| **4.0** | **Marketing** | | | | | | | |
| 4.1 | Pig identification, registration and traceability | 500 | | | | | 500 | Public 80% Private 20% |
| 4.2 | Establish 1 large-scale specialized investors' pig farms | 1,200 | | | | | 1,200 | Public 5% Private 95% |
| 4.3 | Establish 15 new commercial specialized pig farms for commercial pig production | 500 | 1000 | | | | 1,500 | Public 5% Private 95% |
| 4.4 | Construct 15 pig marketing centres with slaughter facilities | 1000 | 500 | | | | 1,500 | Public 10% Private 90% |
| | **Total marketing** | **3,200** | **1,500** | | | | **6,700** | |
| | **TOTAL investment** | **3,780** | **2,195** | | | | **5,975** | **21% pubic**<br>**79% private** |

Table 75 above shows that the investment cost for the MLI scenario adds up to about RWF 6 billion which is slightly less than half of the investment recommended to achieve the development targets for the RLI scenario. In the MLI scenario 21% of the investment comes from the public and 79% from the private sector. In both the MLI and RLI scenarios the development of the sub sector would be driven by private investment.
Table 76 below compares impacts of MLI and RLI on pig meat production and value added.

Table 76: MLI and RLI production and GDP comparison 2021/22

| **Scenario Outcomes** | **Investment Scenarios** | | | **% difference MLI/RLI** |
|---|---|---|---|---|
| | **BAU** | **Recommended** | **Medium** | |
| Production 5th year Pig Meat in MT | 26,665 | 67,676 | 45,461 | -33% |
| GDP 5th year | 28,184 | 66,266 | 49,659 | -25% |

Under the MLI scenario pig meat production drops down to 33% of the RLI impact level and GDP would be 25% of the RLI impact level. This constitutes a big difference between the two scenarios. The MLI impacts on pig meat production and GDP contribution are only 70 and 76% greater, respectively, than the impacts under the BAU scenario.

Complimentary interventions and success requirements for the medium investment scenario are the same as in the recommended investment (RLI) scenario

## *Conclusions*

The pig meat production and GDP impacts under the RLI scenario show substantial increments, 239% and 214% respectively, compared to the base year. The RLI would contribute significantly to fulfilling the national all meat requirement and would increase per capita pig meat consumption for Rwanda enough that it would help to meet the Rwandan national development objective of improving food and nutrition security.

It must also be noted that the technical interventions alone will not bring the projected output unless the policy and institutional challenges are addressed.

Needed policies and strategies – to be enacted and implemented

Livestock products pricing policy
Animal breeding strategy
Livestock identification & reporting system policy
Livestock animal and product traceability policy

Regulations and standards

Regulation of animal breeding
Regulation of animal nutrition; standards on animal commercial feeds, crops residues, formulation of the feeds, honey, etc

- Regulation of livestock products pricing and standards on animal products grading (milk, meat, honey, eggs, wool, hides and skins)
- Standards on livestock infrastructure development (slaughterhouses, laboratories, processing factories, etc.)
- Standards on vet services and pharmaceuticals and supplies

Institutional framework

Strengthening the functional linkages and collaboration of institutions from national level to grassroots

Under the MLI scenario, pig meat production and GDP contribution drop by 59 and 49% respectively compared to the RLI scenario. This shows the MLI would significantly slow down the efforts of Rwanda to modernize and transform the sub-sector and prevent Rwanda from closing the production and consumption gap for all meat during the 5-year period of the LMP.

Compared to the BAU, the MLI impact on pig meat production and GDP contribution show declines to only 70 and 76% respectively, indicating the MLI would not be warranted before conducting a thorough financial feasibility analysis, to calculate the benefit-cost ratio (BCR) and net present value (NPV). It is strongly recommended to do a financial feasibility analysis of the MLI scenario before committing the investment resources, no matter how great the budget constraint may be to committing to the RLI.
