# Supplementary material for: Genetic diversity and relationship of Bugesera and Rwamagana indigenous chicken populations with SASSO chickens using DArTseq SNPs
Source: PLoS One. 2025 Sep 12;20(9):e0331316. doi: 10.1371/journal.pone.0331316 (PMC12431124; doi:10.1371/journal.pone.0331316)
Supplement: S3 File — (PDF) [file pone.0331316.s007.pdf]

The Republic of Rwanda

NATIONAL INSTITUTE OF STATISTICS OF RWANDA

Rwanda

Integrated Household Living Conditions Survey

[EICV]

2013/2014

Thematic Report

CELEBRATING 2005 NISR'S 10 YEARS OF POLICY SUPPORT 2015 ANNIVERSARY

- Youth -

National Institute of Statistics of Rwanda

THE REPUBLIC OF RWANDA

NATIONAL INSTITUTE OF STATISTICS OF RWANDA

National Institute of Statistics of Rwanda

Integrated Household Living Conditions Survey

Enquête Intégrale sur les Conditions de Vie des Ménages

(EICV)

Thematic report

# - Youth -

March, 2016

Po Box 6139 kigali Rwanda
www.statistics.gov.rw
info@statistics.gov.rw

The EICV4 - Youth thematic Report, is produced based on the results of the Rwanda Integrated Household Living Conditions Survey – 2013/14 (EICV4) that was conducted by the National Institute of Statistics of Rwanda (NISR).

Additional information about the EICV4 - 2013/14, Youth thematic Report may be obtained from the NISR:

P.O. Box 6139, Kigali, Rwanda; Telephone: (250) 252 571 035
E-mail: info@statistics.gov.rw; Website: http://www.statistics.gov.rw.

Recommended citation:

National Institute of Statistics of Rwanda (NISR), EICV4 - Youth thematic Report,
March 2016.

ISBN: 978-99977-43-06-0

Foreword

The Government of Rwanda needs updated information for monitoring progress on poverty reduction programmes and policies as stipulated in the second Economic Development and Poverty Reduction Strategy (EDPRS2), the Millennium Development Goals (MDGs) and Vision 2020.

The 2013/14 EICV is a follow-up to the 2000/01, 2005/06 and 2010/11 EICV surveys. Each survey provides information on monetary poverty measured in consumption expenditure terms, but also provides complementary socio-economic information that facilitates understanding changes in households living conditions.

The 2013/14 EICV was implemented by the National Institute of Statistics of Rwanda (NISR), in collaboration with different stakeholders in the country.

Results of the 2013/14 EICV indicate substantial progress in poverty reduction and improvement in other socio-economic and demographic indicators in the last three years. The survey shows that poverty is at 39.1% as of 2013/14, down from 44.9% as was reported in 2010/11. During the same period, extreme poverty dropped from 24.1% to 16.3%.

Generally the progress is impressive. However challenges remain; many Rwandans are still poor and for many others living conditions still need to be improved especially in areas of education and employment.

I find these findings very informative; the report is an important vehicle for addressing poverty concerns and informing policy makers and other stakeholders where to intervene. We should stay on course.

I urge all stakeholders: government, researchers, partners and the general public to optimize the use of these findings.

Finally, I congratulate the National Institute of Statistics of Rwanda and all those who contributed in one way or another in this exercise, for the job well done.

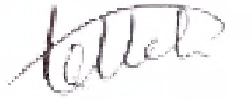

Claver Gatete
Minister of Finance and Economic Planning

Acknowledgements

While celebrating a decade since its establishment, the National Institute of Statistics of Rwanda (NISR) is honoured to present the results of EICV4 conducted in 2013/14.

Through the second National Strategy for the Development of Statistics (NSDS2), NISR has managed to increase the frequency of some surveys to provide timely and update statistics that will facilitate monitoring and evaluation of different policies and programmes at both national and international levels.

The frequency of EICV is now three years. This is an ambitious target that we are committed to achieve in collaboration with our stakeholders in order to support evidence-based decision and planning processes with more frequent and reliable statistics.

In this regard, we present our sincere appreciation to the Government of Rwanda for its support for statistics development in the country, the Ministry of Finance and Economic Planning, and other Government Ministries and Agencies for the facilitation that we received in this endeavour and in other similar efforts.

We express our gratitude to Development Partners that support statistics development in the country and especially EICV4; including: The African Development Bank (AfDB), World Bank, UK Aid, European Union, One UN and other UN agencies in the country. Their contribution was of immense importance to the effective accomplishments of the survey.

We also express our profound gratitude to the advisory team of national and international experts for their advice while constructing the survey, constructing the food basket and setting a new poverty line. Their technical advice contributed to the success of the analysis.

We would also like to thank the EICV management team for their effort throughout the planning and implementation stages of 2013/14 EICV; and also appreciate the valuable support provided by administrative and financial departments of the NISR. Their contribution allowed this exercise to be carried out smoothly.

Yusuf Murangwa
Director General, NISR

Methodological note for readers

Urban and rural classification in EICV3 and EICV4 data

In the preparation of the 2012 Rwanda Population and Housing Census (RPHC), the urban and rural classification of all villages has been updated. There were actually four codes defined for the 2012 Census: (1) urban, (2) rural, (3) peri-urban, and (4) semi-urban. Given the nature of each type of area, the NISR decided to define the urban domain as the combination of urban and semi-urban, and the rural domain as the combination of rural and peri-urban. In the fourth round of the Integrated Household Living Conditions Survey/Enquête Intégrale sur les Conditions de Vie des Ménages (EICV4), the sampling frame for the clusters was based on the 2012 Rwanda Population and Housing Census, and the corresponding new urban and rural classification was used.

At the time of EICV3 analysis, the urban and rural classification of the villages in the EICV3 data was based on the corresponding geographic designations from the previous Rwanda Population and Housing Census conducted in 2002. One reason for this approach was to ensure that the EICV3 urban and rural classification was consistent with that for the EICV2 sample, which was based on a sampling frame from the 2002 Census. For the 2002 Census each old "sector" was classified as urban or rural. Since Rwanda had been divided into new sector prior to the EICV3, it was necessary to use GIS databases to correctly classify the EICV3 sample villages by the 2002 urban–rural code.

In order to enable comparison of EICV4 and EICV3 estimates in urban and rural areas in this report, it has been necessary to apply the same urban/rural classification to data from both survey rounds. Instead of going back to the 2002 classification, it was decided to use the new classification established in 2012 for all tabulations by urban–rural location of the unit of analysis. This ensures that the current distribution of the population and population characteristics are correctly represented. Therefore, the EICV3 estimates for urban/rural areas presented in this report correspond to the new classification established in 2012.

In comparing the distribution of households and population by urban and rural domains within each province, EICV3 data used the urban–rural 2002 code with the corresponding distribution from the EICV4 data using the urban–rural 2012 variable; it was found that there are considerable differences. Estimates for urban and rural areas based on EICV3 data presented in this report may therefore differ from estimates presented in previous EICV3 reports, due to some villages having changed urban/rural status. The table below cross-tabulates the two urban–rural classification variables for the EICV3 sample of households.

Table 0.0: Classification of sample households (unweighted) in the EICV3 survey by 2002 and 2012 urban–rural classifications

	Urban (2002)	Rural (2002)	Total (2002)
**Urban (2012)**	1,357	705	2,062
**Rural (2012)**	792	11,454	12,246
**Total (2012)**	2,149	12,159	14,308

Source: EICV3; urban-rural classification from RPHC 2012.

Rounding of estimates

Estimates presented in the tables are shown rounded to one decimal place. To improve readability, estimates referred to in the interpretation of results have been rounded to the nearest integer, except for the discussion of relatively small percentages. Moreover, estimates of total population or total number of households are shown in tables **expressed in '000's. Due to the rounding, the sum of subpopulation totals (e.g. Provinces** or age groups) can be minimally different from the total population estimated at national level.

Consumption quintiles

The results are presented by quintile. Quintiles are developed by sorting the sample of households by annual consumption values, and then dividing the population into five equal shares. The 20% of individuals with the highest annual consumption are allocated to quintile 5, and the 20% of individuals with the lowest levels of annual consumption are allocated to quintile 1. The poorest households and their members are found in quintile 1 and the richest are found in quintile 5.

Consumption is used as a proxy for income, as is usual when estimating poverty. **Quintiles are a relative measure of individuals' consumption in comparison to the rest of** the population during a specific period. Therefore, comparisons between EICV3 and EICV4 are not informative about and are not comparable in terms of consumption levels because thresholds set to allocate population to the quintiles are not the same in different survey rounds.

Executive Summary

Background to the EICV4 and the EICV series

This report presents and discusses detailed results from EICV4. It focuses on youth and compares EICV4 and EICV3 in the areas of demography of youth, education and public ICT facilities, economic activity and migration.

The EICV4 survey was conducted over a period of 12 months between October 2013 and October 2014. .. Having taken place three years after the EICV3, which was conducted from October 2010 to October 2011, the EICV4 aimed to provide even more timely evidence to support policy-making and continuous evaluation of the EDPRS2. The EICV methodology has remained relatively unchanged since 2000/01. This allows calculating indicators in a consistent way for more than one survey round to investigate trends and inform the planning framework of the Economic Development and Poverty Reduction Strategy (EDPRS) as well as other development programs such as the Millennium Development Goals (MDGs), and Sustainable Development Goals (SDGs).

In this report, most of tables provide the results both from EICV3 and EICV4 and highlight trend over time, as well as different patterns across the provinces, urban and rural areas of Rwanda. Being one of seven thematic reports – Youth, Gender, Education, Environment and Natural Resources, Utilities and Amenities, Economic Activities, and Consumption Patterns- this report- seeks to inform and support the monitoring of EDPRS2 with data from the EICV4. ..

Before,

'youth' was defined in Rwanda as population aged 14 to 35 years until the new national youth policy of November 2015 has officially revised youth age groups and defined it as people aged 16 to 30 years. . This study remained as close as possible to the old age category for comparison purposes with EICV3 but also presented data for the following new age groups where necessary:

Old age groups

14–19 years
20–24 years
25–29 years
30–35 years

New age groups

16–19 years
20–24 years
25–30 years

The priority of the Government of Rwanda is around job creation and self-employment for young people in Rwanda. This report thus focuses on the education and employment situation of young people. It also looks at geographical mobility of young people as one of key factors in determining productivity and access to employment.

Demography

The total youth in Rwanda (aged between 14 and 35 as per the old definition of youth) is 4,474,000 people. These increased from 4,159,000 in 2010/11. However, according to the new policy by MYICT, the total youth between 16 to 30 years was 3,156,000 people according to EICV4 (2013/14).

Overall, 14–35 years old people make up 39% of the total population of Rwanda of all ages. The largest age group within the youth is 14–19 years, as reflected the age pyramid shown in figure 2.1. This group comprises 13.2% of the total population (all ages). The age group of 25–29 years comprises 8% of the total population.

The sex ratio of youth in Rwanda differs by 2 percentage points i.e. 49:51 in the age group of 14–19 but diverges through the old age groups. In 25–29 and 30–35 age groups, males make up 48% of the group and females 52%, which is almost similar to the national level sex ratio.

Education

Around 81% of the population aged 14–35 years know how to read and write. This varies from about 68% for those in the lowest quintile to 90% in the highest quintile. The youngest age groups have a higher literacy rate (85%) than the older age groups (74% for 30–35 years), reflecting the expansion of education in recent years.

10% of Rwandans aged 14–35 years are computer literate. The number is high in Kigali (27%) and among the wealthiest quintile (27%). There is a high difference between urban and rural areas in computer literacy rates. Only about 6% of rural youth are confident in using a computer compared to 27% in Kigali city.

Employment and economic activity

Employment and economic activity rates for young people (14–35) is lower than the one for all working age people (16 years+) which is about 87% especially in 14–19 age category. The overall employment rate for youth is 76% and most of those who are not active are students (16%). Employment rate for 25–35 years old is more than 93%.

The unemployment rate for male youth in Kigali in the seven days preceding the survey was 2.5% and 5.8% for female youth. Youth unemployment rate is less than 1% for all provinces.

Median hours worked by Rwandan youth are 30 hours per week. The mean is slightly high (34.9).

It is apparent that 59% of Rwandans aged 14–35 work less than 35 hours per week, and therefore, might be considered underemployed. About 12% work less than 10 hours per week, and 20% work about 51 hours or more per week.

Many young people (50%) are independent farmers as their main job. The next most common categories for main jobs for youth are wage non-farm work (24%), wage farm jobs (13%) and independent non-farm (11%).

The main pattern shows a decrease in the number of males in wage farming activities, from 16% for 14-19 years old people to just 8% for 30-35 years old people.

Among the youth working in wage employment, the majority (92%) is in private sector and 7% are in the public sector. Female youth are more likely to work in the public sector than male youth. 64% of young people work in agriculture, forestry and fishing as their main job. Another growing sector of main employment is trade (11%) for both male and female youth. In average, youth spend 19 hours a week on domestic duties, in addition to carrying out economic activities. Female youth spend more time on domestic duties than male youth. Females spent hours ranging from 12 to14 hours in cooking for the households in 2013/14.

Migration

On average, young people are more likely to migrate internally and outside the country than the rest of the population. 19% of 14–35 years old people had moved in the five years that preceded the study compared to 13% of all Rwandans. The rate is high (23%) in the 25–29 and 20-24 age groups. Here, males and females move at a similar rate.

The majority of Rwandans (59%) migrated internally due to family reasons while 22% migrated looking for job in 2013/14. Youth aged 14–19 years migrate a lot internally (73%) due to family reasons.

People who had moved in the five years that preceded the study were more likely to have moved because of work. The people who had moved because of work were about 37% in 2010/11 and 39% in 2013/14.

Table of Contents

Foreword i

Acknowledgements iii

Methodological note for readers v

Executive Summary vii

Table of Contents xi

List of Tables xii

List of figures xv

List of abbreviations xvi

Chapter 1: Introduction 1

Chapter 2: Demography 3

Chapter 3: Education 5

3.1. Literacy 5
3.2. Public ICT facilities 7

Chapter 4: Employment and economic activity 9

4.1. Poverty in Rwanda 16
4.2. Time Spent on domestic work 16

Chapter 5: Migration 19

Annex A. District disaggregation tables for youth population, EICV4 21

Annex B. Confidence intervals for selected indicators from EICV4 29

List of Tables

Table 0.0: Classification of sample households (unweighted) in the EICV3 survey by 2002 and 2012 urban–rural classifications vi
Table 2. 1: Youth population, (EICV4, EICV3) 3
Table 2.2: Sex ratio in youth (EICV4, EICV3) 4
Table 2.3: Percentage of youth by province, EICV4 4

Table 3. 1: Literacy rate (%) among population 14–35 years of age by province, urban/rural, and consumption quintiles (EICV4, EICV3) 5
Table 3.2: Percentage of youth (14-35) that have attended technical and vocational school (EICV4, EICV3) 6
Table 3.3: Computer literacy rate (%) among population aged 14–35 years by province, urban/rural, sex, age groups, and consumption quintiles (EICV4, EICV3) 6
Table 3.4: Usage rate of internet service facilities, EICV4 7

Table 4. 1: Distribution of youth by usual economic activity status, according to sex and age group (EICV4, EICV3) 9
Table 4.2: Distribution of youth by usual economic activity status, according to sex and province 10
Table 4.3: Median and mean hours worked in all jobs in the last seven days, by sex and age group (EICV4, EICV3) 10
Table 4.4: Hours worked in last seven days, by age group 11
Table 4.5: Main usual jobs of youth aged 14 to 35 years, by age group 12
Table 4. 6: Main usual jobs of youth aged 14-35 years, by province 12
Table 4.7: Youth in waged work by public and private sector, sex and age group 13
Table 4.8: Industry of main job by sex and by age group 14
Table 4.9: Distribution (%) of usually employed youth (14-35) by occupation group of main usual jobs, according to urban/rural and sex (EICV4) 15
Table 4.10: Proportion of young people below poverty line in 2013/14 16
Table 4.11: Proportion of young people in extreme poverty in 2013/14 16
Table 4.12: Hours (median) per week spent on domestic tasks, by age group and sex (EICV4, EICV3) 17
Table 4.13: Hours (median) per week spent by youth (14-35) on domestic tasks, by province and sex 17

Table 5. 1: Migration in last five years (internal and outside the country) (EICV4, EICV3) 19
Table 5.2: People who have ever moved internally, by sex, age, and reason (EICV4, EICV3) 19
Table 5.3: People who have moved internally in the last five years, by sex, age, and reason (EICV4, EICV3) 20

Table A. 1: Youth population by district, EICV4, EICV3 (000s, %)....21
Table A.2: Youth population by district, Male, EICV4, EICV3 (000s, %)....23
Table A.3: Youth population by district, Female, EICV4, EICV3 (000s, %)....26

Table B. 1: Percentage of total young population aged 14 to 35 years who have never been to school, by domain....29
Table B.2: Percentage of total young population aged 14 to 35 years who did not complete primary, by domain....30
Table B.3: Percentage of total young population age 14 to 35 years who completed primary, by domain....31
Table B.4: Literacy rate for young male population age 14 to 35 years, by domain....32
Table B.5: Literacy rate for young female population age 14 to 35 years, by domain....33

List of figures

Figure 2. 1: Percentage distribution of population by age groups and sex, EICV4 --------- 3

List of abbreviations

EDPRS	:	Economic Development and Poverty Reduction Strategy
EICV	:	Enquête Intégrale sur les Conditions de Vie des Ménages (Integrated Household Living conditions Survey)
HH	:	Household
ICT	:	Information and Communication Technology
ILO	:	International Labour Organization
MYICT	:	Ministry of Youth, Information and Communication Technology
IPRC	:	Integrated Polytechnic Regional Center
VTC	:	Vocational Technical Centers
NISR	:	National Institute of Statistics of Rwanda
RWF	:	Rwandan Franc

Chapter 1: Introduction

The second Economic Development and Poverty Reduction Strategy (EDPRS2: 2013-2018) **stems from Rwanda's Vision 2020 and guides medium term actions that will lead to the achievement of the Vision's goals. The main goal of the strategy is to speed up Rwanda's** progress towards a middle-income status country status and to a better life of Rwandans.

The evidence collected through the EICV4 fieldwork which was carried out by the NISR between October 2013 and October 2014 provided a key input into the mid-term evaluation of the EDPRS2. There are three EICV4 reports that were published by the NISR in September 2015: a Main Indicators Report, Rwanda Poverty Profile Report, and Social Protection/VUP Report.

This report is one of seven (7) thematic reports – Youth, Gender, Education, Environment and Natural Resources, Utilities and Amenities, Economic Activities, and Consumption Patterns, which will play key role in the mid-term evaluation of EDPRS2. The focus of the study being on the situation of the youth in Rwanda is about geographical mobility, education, economic activities, as well as demographic features of young people in Rwanda.

Before, 'youth' was defined in Rwanda as population aged 14 to 35 years until the new national youth policy of November 2015 has officially revised youth age groups and defined it as people aged 16 to 30 years.

. In this report, the definition of youth contained in EICV3 (from 14 to 35 years) was maintained for better comparison and the new definition (from 16 to 30 years) was also considered to get the related trends. The following age groups were used:

Old age groups

14–19 years
20–24 years
25–29 years
30–35 years

New age groups

16–19 years
20–24 years
25–30 years

Chapter 2: Demography

The total youth population in the age group of 14-35 years in Rwanda was 4,474,000 in 2013/14. This youth population increased from 4,159,000 in 2010/11. According to the new age group classification (16-30 years), the total youth population was found to be 3,156,000 people according to EICV4.

Figure 2. 1: Percentage distribution of population by age groups and sex, EICV4

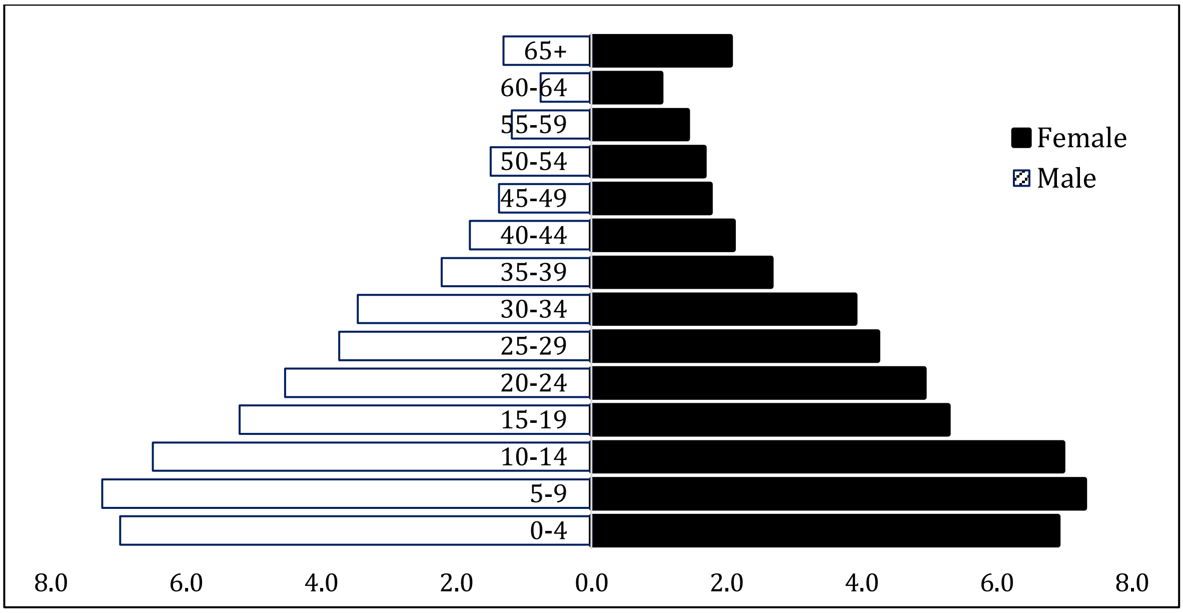

Considering the old definition of youth (14-35 years), the study reveals that the proportion of youth increased slightly since EICV3. However, there has been a slight decrease in the proportion of youth in 14–19 age group, from 14% (EICV3) to 13% (EICV4).Overall, people aged between 14 and 35 years make 39% of the total population of Rwanda of all ages in EICV4. Table 2.1 points out that the largest age group within the youth is 14–19 years, representing 13% of the total population (all ages), while the lowest is 25–29 years (8%).

Table 2. 1: Youth population, (EICV4, EICV3)

EICV4	Male (000s)	Female(000)	Total(000)	Percent
14-19	739	769	1507	13.2
20-24	519	563	1082	9.5
25-29	427	484	911	8.0
30-35	457	517	974	8.5
**Total**	2,142	2,332	4,474	39.1
**Total population all ages**	**5,465**	**5,967**	**11,432**	**100**

Source: EICV4

EICV3	Male (000s)	Female(000)	Total(000)	Percent
14-19	740	751	1491	13.9
20-24	479	547	1026	9.5
25-29	408	477	885	8.2
30-35	344	413	757	7.0
**Total**	1,971	2,188	4,159	38.6
**Total population all ages**	**5,105**	**5,657**	**10,762**	**100**

Source: EICV3

As Table 2.2 illustrates, the sex ratio of youth in Rwanda in the age group of 14–19 is 49% for males against 51% for females. Overall, the sex ratio of youth in Rwanda is 49% for males versus 52% for females.

Table 2.2: Sex ratio in youth (EICV4, EICV3)

EICV4	Male	Female	Total(000)
14-19	49.0	51.0	1,507
20-24	48.0	52.1	1,082
25-29	46.9	53.1	911
30-35	46.9	53.1	974
**Total**	**47.8**	**52.2**	**4,474**

Source: EICV4

EICV3	Male	Female	Total(000)
16-19	49.1	50.9	963
20-24	48.0	52.0	1,082
25-30	47.1	52.9	1,112
**Total**	**48.0**	**52.0**	**3,156**

Source: EICV3 the provinces with the largest number of youth are Eastern Province (25% of all youth) and Western Province (23%). Kigali City has the smallest number of youth (14%). The table 2.3 illustrates the distribution of youth by provinces and age groups.

Table 2.3: Percentage of youth by province, EICV4

EICV4	Percentage of youth by province					
	Kigali City	Southern Province	Western Province	Northern Province	Eastern Province	All Rwanda
14-19	11.83	22.94	22.63	16.91	25.69	100
20-24	15.3	20.15	23.43	14.84	26.28	100
25-29	17.03	20.78	22.14	15.71	24.34	100
30-35	14.05	22.78	22.72	15.01	25.44	100
Total 14-35	14.21	21.79	22.74	15.75	25.5	100
**Total (14-35) (000s)**	**636**	**975**	**1,018**	**705**	**1,141**	**4,474**

Source: EICV4

EICV4	Percentage of youth by province					
	Kigali City	Southern Province	Western Province	Northern Province	Eastern Province	All Rwanda
16-19	13,03	22,66	22,65	16,75	24,91	100
20-24	15,3	20	23,43	14,84	26,28	100
25-30	16,52	21	22,09	15,53	25,0	100
Total 16-30	15,04	21,17	22,72	15,67	25,41	100
**Total (16-30) (000s)**	**475**	**668**	**717**	**494**	**802**	**3 156**

Source: EICV3

Chapter 3: Education

3.1. Literacy

Table 3.1 shows that, 81.2% of the population aged 14–35 know how to read and write. This varies from 67% for those in the lowest quintile to 90% for those in the highest quintile. Overall, literacy rates are similar for males and females, with higher rate in urban areas (91%) compared to rural areas (79%). A small increase has been noticed at national level, from 80% in 2010/11 to 81% in 2013/14.

Table 3. 1: Literacy rate (%) among population 14–35 years of age by province, urban/rural, and consumption quintiles (EICV4, EICV3)

EICV4	Male	Female	Total
**All Rwanda**	81.2	81.7	**81.3**
**Urban/rural**			
Urban	92.3	90.8	**91.2**
Rural	78.8	78.8	**78.7**
**Province**			
Kigali City	93.5	91.4	**92.4**
Southern Province	77.8	82.1	**79.9**
Western Province	80.2	77.9	**79.0**
Northern Province	82.6	82.6	**82.7**
Eastern Province	79.2	77.5	**77.9**
**Quintile**			
Q1	69.3	67.0	**67.5**
Q2	78.7	76.8	**77.5**
Q3	80.0	81.0	**80.4**
Q4	84.2	85.0	**84.5**
Q5	89	91.6	**90**

Source: EICV4

EICV3	Male	Female	Total
**All Rwanda**	80.6	79.8	**80.2**
**Urban/rural**			
Urban	88.8	87.9	**89.0**
Rural	78.8	78.1	**78.3**
**Province**			
Kigali City	90.5	89.5	**91.0**
Southern Province	75.2	78.5	**77.3**
Western Province	81.1	77.1	**79.2**
Northern Province	81.9	81.1	**80.8**
Eastern Province	79.0	77.8	**78.4**
**Quintile**			
Q1	69.1	68.3	**68.1**
Q2	74.9	74.2	**75.2**
Q3	79.4	78.4	**79.1**
Q4	82.9	82.8	**83.1**
Q5	89	90.3	**90**

Source: EICV3

Of all Rwandans aged 14–35 years, about 3% had attended technical or vocational school in 2013/14 compared to 2% in 2010/11. Kigali city had a high percentage (6%) of youth who had attended technical or vocational school and wealthier quintiles dominate in vocational trainings (see Table 3.2).

Table 3.2: Percentage of youth (14-35) that have attended technical and vocational school (EICV4, EICV3)

	EICV 4	EICV 3
**All Rwanda**	3.3	2.1
**Urban/rural**		
Urban	5.7	2.4
Rural	3.6	1.9
**Province**		
Kigali City	5.9	1.9
Southern Province	3.7	3.3
Western Province	3.1	2.4
Northern Province	4.2	1.2
Eastern Province	4.0	1.3
**Sex**		
Male	4.9	2.4
Female	3.3	1.8
**Age groups**		
14–19	3.3	1.3
20–24	5.4	2.6
25–29	3.5	11.3
30-35	3.1	20.8
16-30	4.1	2.2
**Quintile**		
Q1	2.3	2.6
Q2	3.0	0.8
Q3	3.9	3.2
Q4	4.5	1.6
Q5	5.4	2.2

Source: EICV4, EICV3. Table 3.3 shows that 10.3% of the population aged 14–35 were computer literate in 2013/14. Male were reported to be more computer literate (12%) compared to females (9%). Among provinces, Kigali city had the highest percentage of computer literate people (27%) in 2013/14.

Table 3.3: Computer literacy rate (%) among population aged 14–35 years by province, urban/rural, sex, age groups, and consumption quintiles (EICV4, EICV3)

	EICV 4	EICV 3
**All Rwanda**	10.3	6.5
**Urban/rural**		
Urban	28.4	22.0
Rural	5.9	2.9
**Province**		
Kigali City	27.2	21.5
Southern Province	7.9	3.9
Western Province	7.9	4.1
Northern Province	9.3	6.2
Eastern Province	7.7	4.0
**Sex**		
Male	11.8	8.0
Female	9.0	5.2

	EICV 4	EICV 3
**Age groups**		
14–19	5.3	3.6
20–24	15.8	9.5
25–29	14.4	8.4
30-35	8.3	5.9
**Quintile**		
Q1	2.3	0.7
Q2	3.1	1.0
Q3	4.7	2.0
Q4	7.6	3.6
Q5	27.4	19.3

Source: EICV4, EICV3.

3.2. Public ICT facilities

At the national level, 6% of young population (14-35) used public internet service facility, 49% never used it at all in 2013/14, and 45% were not aware of the service. People in Kigali were most likely to use internet facilities compared to other provinces as shown by Table 3.4.

Table 3.4: Usage rate of internet service facilities, EICV4

Province	Not at all	Sometimes	Often	Use Regularly	Internet use	Not aware of the service	Total
**All Rwanda**	**49.1**	**3.9**	**1.2**	**0.9**	**6**	**44.8**	**100**
Kigali City	72.7	9.9	3.3	4.6	**17.8**	9.6	100
Southern Province	40.5	2.3	0.8	0.9	**4**	55.6	100
Western Province	50.1	3.6	0.9	0.4	**4.9**	45.1	100
Northern Province	50.0	3.9	1.4	0.4	**5.7**	44.3	100
Eastern Province	44.9	3.0	0.9	0.2	**4.1**	51.0	100
**Age-group**							
14-19	50.6	5.3	1.7	1.0	**8**	41.4	100
20-24	52.6	6.7	2.4	1.7	**10.8**	36.6	100
25-29	53.2	5.2	1.8	1.9	**8.9**	37.9	100
30-35	50.8	3.6	1.1	1.2	**5.9**	43.3	100
**Sex**							
Male	49.4	4.0	1.3	1.0	**6.3**	44.3	100
Female	48.8	3.9	1.2	0.8	**5.9**	45.4	100

Source: EICV4

Chapter 4: Employment and economic activity

According to EICV4 employment and economic activity rate for young people (14–35) was found lower than the rate of all working age people (16 years+) which was about 87%, especially in 14–19 age category. The overall proportion of youth who is unemployed was 76%. Most of the people who were not active were students (16%). The employment rate for people in 25–35 years age category were more than 93% as portrayed in (Table 4.1).

Table 4. 1: Distribution of youth by usual economic activity status, according to sex and age group (EICV4, EICV3)

EICV4	14-19		20-24		25-29		30-35		Total		
	Male	Female	Male	Female	Male	Female	Male	Female	Male	Female	Total
Employed	48.9	46.8	81.4	80.4	94.7	94.9	93.1	98.4	76.5	75.6	76.1
Unemployed	0.5	0.3	0.9	2.2	0.9	1	2.3	0.4	0.7	1.3	1
Student	34	35.8	16.3	13.9	3.9	3.2	2	0.4	16.4	15.6	16
Domestic	2.1	3	0.4	2.3	0	0.1	1.8	0.1	0.8	2.2	1.6
Disability	0.5	0.4	0.7	1	0.3	0.6	0.7	0.3	0.5	0.7	0.6
Other	13.9	13.6	0.3	0.2	0.2	0.2	0	0.4	5	4.5	4.8
**Total**	**100**	**100**	**100**	**100**	**100**	**100**	**100**	**100**	**100**	**100**	**100**
Usually active	60.3	57.3	89.8	88.8	98	97.3	99.4	98.7	86.6	84.8	85.7
Inactive	39.7	42.7	10.2	11.2	2	2.7	0.6	1.3	13.4	15.2	14.3
**Total**	**100**	**100**	**100**	**100**	**100**	**100**	**100**	**100**	**100**	**100**	**100**

Source: EICV4

EICV3	14-19		20-24		25-29		30-35		Total		
	Male	Female	Male	Female	Male	Female	Male	Female	Male	Female	Total
Employed	37.9	39	77.5	80.6	94.7	94.4	97.9	97.7	69.8	72.5	71.2
Unemployed	0.3	0.3	1.6	1.7	0.9	1.9	0.7	0.8	0.8	1.1	1
Student	57.9	58	19.8	15.5	3.9	2.3	0.3	0.3	27.4	24.3	25.7
Domestic	2.4	2.2	0.5	1.6	0	0.9	0	1	1	1.5	1.3
Disability	0.7	0.4	0.6	0.5	0.3	0.4	0.4	0.2	0.5	0.4	0.5
Other	0.8	0.6	0.1	0	0.2	0.1	0.6	0.1	0.5	0.2	0.3
**Total**	**100**	**100**	**100**	**100**	**100**	**100**	**100**	**100**	**100**	**100**	**100**
Usually active	38.2	39.2	79	82.3	95.6	96.3	98.6	98.5	70.6	73.6	72.2
Inactive	61.7	60.8	20.9	17.7	4.4	3.7	1.4	1.5	29.4	26.4	27.8
**Total**	**100**	**100**	**100**	**100**	**100**	**100**	**100**	**100**	**100**	**100**	**100**

Source: EICV3

Youth unemployment rate is generally low in Rwanda. This is because unemployment by ILO definition is underestimated in countries without extensive social safety nets. To be classified as unemployed, someone must be working less than one hour per week and also actively looking for work. Therefore, a more informative indicator in countries like Rwanda is underemployment, which looks at people working less hours but who would like to be working more hours if work was available.

Unemployment rate according to ILO definition is only significant for youth in Kigali. The unemployment rate for male youth in Kigali during seven days that preceded the study was about 3% while the unemployment rate for female youth was 6%. Youth unemployment rate was less than 1% for all provinces.

Table 4.2 shows that the rate of young people whose main activity is domestic duties was significantly higher for females in Kigali (8%) than males ().

Table 4.2: Distribution of youth by usual economic activity status, according to sex and province

EICV4		Employed	Unemployed	Student	Domestic	Disability	Other	Total	Usually	Inactive	Total
**Kigali city**	**Male**	72.3	2.5	20.3	0.9	0.4	3.6	**100**	74.8	25.2	**100**
	**Female**	63.1	5.8	18.3	7.7	1	4	**100**	69	31	**100**
**Southern Province**	**Male**	73.7	0.4	17.9	1.2	0.8	6.1	**100**	74.1	25.9	**100**
	**Female**	73.4	0.4	18.1	1.6	0.8	5.6	**100**	73.8	26.2	**100**
**Western Province**	**Male**	78.9	0.5	15.4	0.8	0.5	3.9	**100**	79.4	20.6	**100**
	**Female**	81	0.9	13.2	1.1	0.6	3.3	**100**	81.9	18.1	**100**
**Northern Province**	**Male**	80.7	0.3	12.7	0.5	0.7	5.1	**100**	81	19	**100**
	**Female**	82.1	0.4	12.1	0.6	0.5	4.3	**100**	82.5	17.5	**100**
**Eastern Province**	**Male**	76.9	0.4	16	0.8	0.4	5.6	**100**	77.3	22.7	**100**
	**Female**	75.4	0.5	16.4	1.7	0.7	5.3	**100**	75.9	24.1	**100**
**Total**	**Male**	76.5	0.7	16.4	0.8	0.5	5	**100**	77.3	22.7	**100**
	**Female**	75.6	1.3	15.6	2.2	0.7	4.5	**100**	76.9	23.1	**100**
	**All**	76.1	1	16	1.6	0.6	4.8	**100**	77.1	22.9	**100**

Source: EICV3

Table 4.3 indicates that median hours worked by Rwandan youth are 30 hours per week. The mean is slightly high at 34%. Findings have found that males work more hours than females across all age groups of youth.

Table 4.3: Median and mean hours worked in all jobs in the last seven days, by sex and age group (EICV4, EICV3)

Age group EICV4	Hours	Sex		
		Male	Female	Total
14-19	Mean	31.8	30.5	31.1
	Median	27	25	25
20-24	Mean	39.1	30.5	34.7
	Median	35	26	30
25-29	Mean	41.9	31.4	36.4
	Median	37	30	32
30-35	Mean	41.2	31.5	36.1
	Median	38	30	32
All 14-35	Mean	39.1	31.1	34.9
	Median	35	28	30
**All 16-30**	**Mean**	**38.2**	**30.6**	**34.3**
	**Median**	**35**	**27**	**30**

Source: EICV4

Age group EICV3	Hours	Sex		
		Male	Female	Total
14-19	Mean	31.8	29.7	30.7
	Median	25	23	24
20-24	Mean	34.3	25.5	29.5
	Median	30	21	24
25-29	Mean	33.2	23.7	28.2
	Median	28	20	24
30-35	Mean	33.8	24	28.6
	Median	28	20	24
All 14-35	Mean	33.4	25.2	29.1
	Median	28	21	24
**All 16-30**	**Mean**	**33.2**	**25.4**	**29.1**
	**Median**	**28**	**21**	**24**

Source: EICV3

Looking at the distribution of hours worked in detail, table 4.4 shows that 60% of Rwandans aged 14‒35 years work less than 35 hours per week, and therefore might be considered underemployed. About 14% work less than 10 hours per week, and 20% work r 51 hours or more per week.

Table 4.4: Hours worked in last seven days, by age group

EICV4	14-19	20-24	25-29	30-35	Total 14-35	Total 16-30
1-5	7.1	4.5	3.5	2.8	4.2	4.6
6 -10	12.2	8.5	7	7.1	8.3	8.9
11-15	10.4	8.8	7	7.4	8.2	8.4
16-20	10.8	10.4	9.2	8.8	9.7	10
21-25	9.7	10	10.3	10.4	10.2	10.1
26-30	11.2	11.8	12	12.2	11.9	11.8
31-35	4.7	5.8	6.5	6.6	6	5.9
36-40	7.9	9.4	10.4	10.7	9.8	9.4
41-45	3.8	4.7	5.8	6.7	5.4	5
46-50	4.4	5.3	6.4	6.8	5.9	5.6
51+	17.7	20.7	21.9	20.6	20.5	20.3
**Total**	**100**	**100**	**100**	**100**	**100**	**100**

Source: EICV4

EICV3	14-19	20-24	25-29	30-35	Total 14-35	Total 16-30
1-5	7.9	5.9	5.1	3.8	5.8	5.8
6 -10	10.1	8.1	7.9	9.4	8.5	8.5
11-15	9.4	8.9	9.3	7.4	9	9
16-20	9.9	9.5	11.6	10.8	10.5	10.5
21-25	8.6	11.5	11.6	11.3	11	11
26-30	9.4	11.2	11.6	11.4	11	11
31-35	7.4	8.1	8.4	7.7	8.1	8.1
36-40	6.5	8	8.7	9	8.1	8.1
41-45	4.9	5.3	5.2	6.5	5.3	5.3
46-50	3.9	5.1	4.7	5.9	4.8	4.8
51+	22	18.4	15.9	16.8	18	18
**Total**	**100**	**100**	**100**	**100**	**100**	**100**

Source: EICV3

The findings of the study indicate t About 50% of young people are independent farmers as their main job,24% are waged non-farm workers, 13% have wage farm jobs and about 11% as

independent non-farmers. Table 4.5 indicates that the main pattern is a decrease in the number of males in wage farming activities, from 16% for 14-19 years category to just 8% for 30 - 35 years category. In contrast, more than half of females remain in independent farming activities from the age of 14 to the age of 35. Overall, this means that males have a much higher chance of leaving farming as their primary job as they get older than females.

Table 4.5: Main usual jobs of youth aged 14 to 35 years, by age group

Main usual jobs: EICV4	14-19		20-24		25-29		30-35		Total (14-35)			16-30
	Male	Female	Male	Female	Male	Female	Male	Female	Male	Female	Total	
Wage Farm	15.9	12.9	12.7	14.7	9.6	15.2	8.3	16	11.4	14.9	13.2	13
Wage Non farm	28.1	20.2	35.9	15.9	39.1	13.6	36.2	10.1	35	14.5	24.4	26
Independent farmer	48	58.7	38.9	57.3	37.3	57	43.3	59.9	41.7	58.3	50.3	49
Independent non farmer	6	6	11.3	9.8	13.3	12.6	12.1	12.3	10.8	10.5	10.6	10
Unpaid non farmer	2	1	1.1	2	0.4	1.4	0.2	1.3	0.7	1.5	1.2	1
Not stated	0.6	0.6	0.3	0.3	0.3	0.1	0	0.3	0.3	0.3	0.3	0
Total	100	100	100	100	100	100	100	100	100	100	100	100
**14-35 (000s)**	**360**	**359**	**421**	**452**	**405**	**449**	**449**	**500**	**1,636**	**1,761**	**3,398**	**1,249**

Source: EICV4

Main usual jobs: EICV3	14-19		20-24		25-29		30-35		Total (14-35)		
	Male	Female	Male	Female	Male	Female	Male	Female	Male	Female	Total
Wage Farm	14.8	9.1	12.1	11.8	9.5	10.6	9.6	10.1	11.3	10.5	10.9
Wage Non farm	28.5	20.1	33.3	13.3	33.3	9.7	31.3	7.6	31.9	12.1	21.3
Independent farmer	44.3	55	42.4	63.7	42.8	67.4	42.6	68.6	43	64.4	54.4
Independent non farmer	7.1	7.3	10.2	7.8	13.4	9.3	15.6	10.4	11.8	8.8	10.2
Unpaid non farmer	1.8	2	0.9	1.5	0.7	2.2	0.6	2.4	0.9	2	1.5
Not stated	3.5	6.5	1.1	1.9	0.3	0.9	0.4	0.9	1.2	2.2	1.7
Total	100	100	100	100	100	100	100	100	100	100	100
**Total working youth (000s)**	**280**	**292**	**371**	**441**	**386**	**451**	**337**	**403**	**1,375**	**1,586**	**2,961**

Source: EICV3

Across provinces, Kigali city has a high number of youth, both males (72%) and females (52%) in wage non- farm jobs in 2013/14 and there were remarkable differences in all provinces whereby males dominated in wage non- farm jobs (Table 4.6).

Table 4. 6: Main usual jobs of youth aged 14-35 years, by province

EICV4	Kigali city		Southern Province		Western Province		Northern Province		Eastern Province		Total (14-35)		
	Male	Female	Male	Female	Male	Female	Male	Female	Male	Female	Male	Female	All
Wage Farm	2.3	3.7	12	14.2	12.7	17.1	12	17.9	14.2	16.2	11.4	14.9	13.2
Wage Non farm	71.7	52.1	31.4	12.1	33.4	9	28.3	8.1	24.6	9.1	35	14.5	24.4
Independent	6.7	15.7	47.3	63.8	43.1	60.9	50.7	67.2	48.6	64.8	41.7	58.3	50.3

EICV4	Kigali city		Southern Province		Western Province		Northern Province		Eastern Province		Total (14-35)		
	Male	Female	Male	Female	Male	Female	Male	Female	Male	Female	Male	Female	All
farmer													
Independent non farmer	18.5	24	8.4	8.5	9.1	10.9	8.6	6.1	11.6	8.3	10.8	10.5	10.6
Unpaid non farmer	0.8	3.8	0.7	1.3	1	1.7	0.3	0.5	0.8	1.2	0.7	1.5	1.2
Not stated	0.1	0.7	0.3	0.1	0.7	0.4	0.1	0.2	0.2	0.4	0.3	0.3	0.3
**Total**	**100**	**100**	**100**	**100**	**100**	**100**	**100**	**100**	**100**	**100**	**100**	**100**	**100**

Source: EICV4

EICV3	Kigali city		Southern Province		Western Province		Northern Province		Eastern Province		Total (14-35)		
	Male	Female	Male	Female	Male	Female	Male	Female	Male	Female	Male	Female	All
Wage Farm	3.6	5.1	11.9	10.7	15	14	12	9.9	10.5	9.7	11.3	10.5	10.9
Wage Non farm	67.9	48.9	25.8	8.8	32	8.1	29.8	7.4	19.9	7.1	31.9	12.1	21.3
Independent farmer	7.1	21.1	49.2	72.7	40.5	60.6	46.4	71.7	55.6	73.5	43	64.4	54.4
Independent non farmer	18.7	18.2	11.9	4.7	10.1	13.1	9.6	6.4	11.4	6	11.8	8.8	10.2
Unpaid non farmer	1.7	4.5	0.7	1.4	0.9	2.1	0.3	0.6	1.3	2.6	0.9	2	1.5
Not stated	0.9	2.2	0.4	1.6	1.4	2	1.9	4	1.2	1.2	1.2	2.2	1.7
**Total**	**100**	**100**	**100**	**100**	**100**	**100**	**100**	**100**	**100**	**100**	**100**	**100**	**100**

Source: EICV3

Among the youth working in waged employment, the majority (92%) is in the private sector while 7% are in the public sector as indicated in Table 4.7.

Table 4.7: Youth in waged work by public and private sector, sex and age group

EICV4	14-19		20-24		25-29		30-35		Total (14-35)			16-30
	Male	Female	Male	Female	Male	Female	Male	Female	Male	Female	Total	
Public	0.9	0.5	2.9	3.5	8.4	11.3	13.7	13.7	6.8	7.4	7	6.4
Private	98.5	99.5	96.6	94.9	90.4	87.1	85.2	84.9	92.4	91.4	92	92.5
Others	0.6	0	0.4	1.6	1.2	1.6	1.2	1.5	0.9	1.2	1	1.1
**Total**	**100**	**100**	**100**	**100**	**100**	**100**	**100**	**100**	**100**	**100**	**100**	**100**

Source: EICV4

EICV3	14-19		20-24		25-29		30-35		Total (14-35)		
	Male	Female	Male	Female	Male	Female	Male	Female	Male	Female	Total
Public	1.2	0.2	4.3	5.7	9.8	11.5	15.2	13.2	7.7	7.4	7.6
Private	97.9	98.5	94.4	93.7	88.2	86.9	82.5	84.5	90.6	91.3	90.9
Others	0.9	1.3	1	1	2	1.6	2.2	2.3	1.6	1.4	1.5
**Total**	**100**	**100**	**100**	**100**	**100**	**100**	**100**	**100**	**100**	**100**	**100**

Source: EICV3

64% of young people work in agriculture, forestry and fishing as their main job. For males, this proportion decreases for older age groups, from 64% (14–19 years old) to 52% (30–35 years old). This pattern reverses for female as the proportion increases for old age groups, from 72% (14–19 years old) to 76% (30–35 years old).

Another growing sector of main employment according to EICV4 is trade (11% overall) for both males and females. Construction and transport are also growing sectors of employment for young males (Table 4.8).

Table 4.8: Industry of main job by sex and by age group

EICV4	Male				Female					Total
	14-19	20-24	25-29	30-35	14-19	20-24	25-29	30-35	Total (14-35)	16-30
Agriculture, Forestry, and Fishing	63.8	52.7	47.9	52.4	72.1	72.4	72.4	76.2	64.0	61
Mining and Quarrying	1.9	2.8	2.8	2.2	0.1	0.4	0.1	0.3	1.3	1.6
Manufacturing	1.2	2.8	3.3	3.4	0.8	1.7	2.1	1.1	2.1	2.1
Electricity, Gas and Air Condition	0.0	0.2	0.2	0.4	0.0	0.0	0.1	0.0	0.1	0.1
Water Supply, Gas and Remediation	0.0	0.1	0.2	0.4	0.0	0.2	0.0	0.0	0.1	0.1
Construction	5.5	9.4	9.2	9.5	2.3	2.0	1.3	0.7	4.9	6.1
Wholesale and Retail Trade, Repair	9.3	12.2	11.5	10.7	7.7	10.1	12.1	11.8	10.8	11.5
Transportation and Storage	2.1	6.4	7.7	6.2	0.2	0.3	0.4	0.2	2.9	3.1
Accommodation and Food Service Activity	0.7	0.7	1.5	0.9	0.5	0.8	1.1	0.5	0.8	0.8
Information and Communication	0.0	0.4	0.5	0.3	0.0	0.0	0.4	0.1	0.2	0.3
Financial and Insurance Activities	0.0	0.2	0.7	0.6	0.0	0.4	0.6	0.3	0.4	0.4
Real Estate Activities	0.0	0.0	0.0	0.0	0.0	0.0	0.0	0.0	0.0	0.3
Professional, Scientific, and	0.0	0.2	0.9	0.7	0.1	0.3	0.3	0.4	0.4	0.9

EICV4	Male				Female					Total
	14-19	20-24	25-29	30-35	14-19	20-24	25-29	30-35	Total (14-35)	16-30
Technical										
Administrative and Support Service	0.1	0.8	2.0	2.2	0.1	0.7	0.5	0.3	0.9	0.6
Public Administration and Defense	0.1	0.2	0.8	2.2	0.0	0.4	0.6	0.8	0.7	1.5
Education	0.2	0.9	3.3	2.3	0.1	0.9	2.1	2.3	1.6	0.6
Human Health and Social Work Activity	0.1	0.2	0.8	1.3	0.0	0.5	1.3	1.9	0.8	0.5
Arts, Entertainment and Recreation	0.2	0.7	0.9	0.4	0.2	0.4	0.5	0.4	0.5	1.7
Other Service Activities	1.3	1.7	2.8	1.9	0.4	1.8	1.6	1.2	1.6	6.5
Activities of Households as Employer	13.1	7.2	2.6	1.6	15.2	6.7	2.1	1.2	5.8	0.2
Activities of Extraterritorial Organ	0.1	0.1	0.4	0.5	0.1	0.1	0.3	0.2	0.2	0.2
**Total**	**100**	**100**	**100**	**100**	**100**	**100**	**100**	**100**	**100**	**100**

Source: EICV4. **Note**: Job classification used in EICV4 is different from EICV3

Table 4.9 illustrates that 48% of youth work in skilled agriculture, forestry and fishery workers; 29% of them are involved in elementary occupations while 12% of youth are services and sales workers.

Table 4.9: Distribution (%) of usually employed youth (14-35) by occupation group of main usual jobs, according to urban/rural and sex (EICV4)

**EICV4**	**All Rwanda**	**Urban/Rural**		**Sex**	
**Occupation group of main usual job (ISCO 1 digit)**		**Urban**	**Rural**	**Male**	**Female**
Managers	0.3	1.2	0.1	0.5	0.2
Professionals	2.4	7.3	1.4	2.8	2.1
Technical and associated Professionals	0.6	2	0.3	0.7	0.4
Clerical support workers	0.4	1.7	0.1	0.4	0.4
Services and sales workers	12.4	25.6	9.5	13.4	11.4
Skilled agriculture, forestry and fishery workers	47.6	12.6	55.1	35.9	58.5
Craft and related trade workers	3.9	8.4	2.9	6.2	1.8
Plant and machine operators and assemblers	1.5	3.9	1	2.9	0.2
Elementary occupation	28.6	34.8	27.3	35	22.6
Other	0.2	0.5	0.2	0.5	0
Missing information	2.1	2.1	2.1	1.7	2.4
Total	100	100	100	100	100
**Usually employed youth (000s)**	**3,399**	**625**	**2,774**	**1,637**	**1,762**

Source: EICV4.

4.1. Poverty in Rwanda

Poverty in Rwanda is measured by household consumption per adult equivalent member. The value of consumption is measured at the household level, and divided by the number of people in the household (with an adjustment for children). As it is done in most countries, a line is set based on what is considered to be required for a basic minimum level of consumption and below this line people are considered to be in poverty. The poverty line in Rwanda is RWF 159,375 per adult equivalent per year (in January 2014 prices), that is the share of the population that cannot afford to buy a basic basket of goods (food and non-food).While food poverty line is RWF 105,064 per adult equivalent per year (in January 2014 prices).

Overall, about 33% of young people live in households that are below poverty line, which is slightly below the national average for all ages (39%). The poverty rate for young age group of 14–19 years is similar to the national average of 39%. In older age groups, Females are more likely to be poorer than males (Table 4.10).

Table 4.10: Proportion of young people below poverty line in 2013/14

Below poverty line				
	**Male**	**Female**	**Overall**	**Total(000s)**
14-19	39.4	38.8	39.1	1,507
20-24	26.6	28.0	27.3	1,082
25-29	21.9	28.8	25.6	911
30-35	30.8	40.2	35.8	974
**Total (14-35)**	**31.0**	**34.5**	**32.8**	**4,474**

Source: EICV4.

People who cannot afford buying minimum basket of food needed to be healthy are considered to be extremely poor. The rate of extreme poverty among youth (14-35) is 13% compared to the 16% at the national level for all ages. It is clear in table 4.11, that females were much more poor (14%) compared to males (12%) in 2013/14. However, they were well off in all age groups than males except at the age group of 14-19 years.

Table 4.11: Proportion of young people in extreme poverty in 2013/14

Extreme poverty				
	**Male**	**Female**	**Overall**	**Total(000s)**
14-19	17.0	16.7	16.8	1,507
20-24	9.3	9.9	9.6	1,082
25-29	7.1	10.9	9.1	911
30-35	11.1	16.7	14.0	974
**Total(14-35)**	**11.9**	**13.8**	**12.9**	**4,474**

Source: EICV4.

4.2. Time Spent on domestic work

The survey asked all individuals in the households (at least aged six years and above) about the time spent on domestic work. On average, youth spend 19 hours a week on domestic duties, in addition to carrying out economic activities. Some of the domestic tasks qualify as economic activity according to the ILO, including collecting water, firewood and fodder for animals.

Findings have shown that female young people spend much longer time on domestic duties than males. However, as the age for **male's** increases, they do less domestic activities; while it is a reverse for females where 12-14 hours a week were spent in cooking for the households in 2013/14(see Table 4.12).

Table 4.12: Hours (median) per week spent on domestic tasks, by age group and sex (EICV4, EICV3)

Number of hours spent in last 7 days: EICV4	Male				Female				Total (14-35)			
	14-19	20-24	25-29	30-35	14-19	20-24	25-29	30-35	14-19	20-24	25-29	30-35
Fetch water for the household	2	2	1	1	3	2	2	2	3	2	2	2
Forage for firewood	3	2	2	2	3	3	3	2	3	2	2	2
Searching for fodder or grazing	6	6	7	7	3	4	5	6	4	4	6	7
Go to the market for the household for shopping	1	1	2	2	2	2	2	2	2	2	2	2
Cook for the household	4	3	4	3	7	12	14	14	7	10	14	14
Other household chores	1	2	2	2	3	4	6	6	2	3	4	4
**Total**	**8**	**4**	**3**	**4**	**16**	**21**	**29**	**29**	**12**	**10**	**15**	**17**

Source: EICV4

Number of hours spent in last 7 days: EICV3	Male				Female				Total (14-35)			
	14-19	20-24	25-29	30-35	14-19	20-24	25-29	30-35	14-19	20-24	25-29	30-35
Fetch water for the household	3	3	2	2	4	3	3	3	4	3	3	3
Forage for firewood	7	7	7	7	4	4	6	6	5	5	7	7
Searching for fodder or grazing	3	2	2	2	3	3	3	2	3	2	2	2
Go to the market for the household for shopping	2	2	2	2	3	3	3	3	3	3	3	3
Cook for the household	4	5	3	3	7	12	14	14	7	11	14	14
Other household chores	2	2	2	2	4	6	7	7	3	4	5	5
**Total**	**11**	**5**	**5**	**4**	**19**	**24**	**31**	**30**	**15**	**14**	**18**	**18**

Source: EICV3

In all provinces, except in Kigali City and Eastern provinces, females spend more hours (10 hours) cooking for the household while males spend more hours (7 hours) searching for fodder or grazing.

Table 4.13: Hours (median) per week spent by youth (14-35) on domestic tasks, by province and sex

	Male					Female				
**EICV4**	**Kigali City**	**Southern Province**	**Western Province**	**Northern Province**	**Eastern Province**	**Kigali City**	**Southern Province**	**Western Province**	**Northern Province**	**Eastern Province**
Fetch water for the household	2	2	2	2	2	3	2	2	2	3
Forage for firewood	3	2	2	2	2	3	3	3	2	3
Searching for fodder or grazing	6	7	7	7	6	4	4	5	5	3

	Male					Female				
**EICV4**	**Kigali City**	**Southern Province**	**Western Province**	**Northern Province**	**Eastern Province**	**Kigali City**	**Southern Province**	**Western Province**	**Northern Province**	**Eastern Province**
Go to the market for the household for shopping	1	2	2	2	1	2	2	2	2	2
Cook for the household	4	3	4	4	3	8	10	10	10	12
Other household chores	2	1	2	1	2	4	3	3	3	4
**Total**	**5**	**9**	**8**	**10**	**9**	**5**	**9**	**8**	**10**	**9**

Source: EICV4

	Male					Female				
**EICV3**	**Kigali City**	**Southern Province**	**Western Province**	**Northern Province**	**Eastern Province**	**Kigali City**	**Southern Province**	**Western Province**	**Northern Province**	**Eastern Province**
Fetch water for the household	3	3	3	2	3	3	4	3	3	3
Forage for firewood	6	7	7	6	7	3	6	6	4	4
Searching for fodder or grazing	2	3	3	2	3	2	4	3	2	3
Go to the market for the household for shopping	2	3	3	3	2	2	2	3	3	2
Cook for the household	6	4	4	3	3	13	10	11	8	11
Other household chores	2	2	2	2	2	7	3	4	3	5
**Total**	**7**	**12**	**11**	**9**	**10**	**7**	**12**	**11**	**9**	**10**

Source: EICV3

Chapter 5: Migration

Internal migration between districts is a potential labour force market as it allows people to go where jobs are and seek out best opportunities. Table 5.1 shows that on average, young people are more likely to migrate internally and outside the country than the rest of the population. 19% for 14–35 years had moved during the five years that preceded the study compared to 13% for all Rwandans. The rate is highest (23%) in the age groups of 25–29 and 20-24. Findings show that males and females move at a similar rate (Table 5.1).

Table 5. 1: Migration in last five years (internal and outside the country) (EICV4, EICV3)

EICV4	Migrants(000s)	population(000s)	Migrants as % of population
All Rwanda	1,459	11,432	13
14-19	214	1,507	14
20-24	247	1,082	23
25-29	207	911	23
30-35	163	974	17
Male 14-35	397	2,142	19
Female 14-35	435	2,332	19
**All 14–35**	**832**	**4,474**	**19**

Source: EICV4

EICV3	Migrants(000s)	population(000s)	Migrants as % of population
All Rwanda	1,151	10,762	11
14-19	182	1,491	12
20-24	194	1,026	19
25-29	174	885	20
30-35	113	757	15
Male 14-35	315	1,971	16
Female 14-35	349	2,188	16
**All 14–35**	**663**	**4,159**	**16**

Source: EICV3

In 2013/14, according to Table 5.2, the majority of young Rwandans migrated internally due to family reasons (59%) and looking for work (22%). The pattern of internal migration is similar for youth (14-35). Youth aged 14–19 years migrated a lot internally (73%) due to family reasons.

Table 5.2: People who have ever moved internally, by sex, age, and reason (EICV4, EICV3)

EICV4	work	Family	studies	Disasters	lack of land	Return home	Others	Total	Migrants (000s)
**All Rwanda**	**21.9**	**59.1**	**2.5**	**1.3**	**6.4**	**5.5**	**3.4**	**100**	**1356**
14-19	21.5	72.6	4.2	0.2	0.0	0.9	0.6	100	201
20-24	29.7	55.7	6.0	0.7	1.5	4.1	2.4	100	235
25-29	32.0	51.4	4.3	0.8	3.8	4.4	3.3	100	195
30-35	33.2	46.4	2.1	1.6	6.7	6.3	3.8	100	154
Male 14-35	13.5	17.3	1.6	0.5	1.1	9.3	56.7	100	371
Female 14-35	6.0	24.8	1.2	0.3	0.9	9.3	57.5	100	414
**All 14–35**	**9.6**	**21.2**	**1.4**	**0.4**	**1.0**	**9.3**	**57.1**	**100**	**785**

Source: EICV4

EICV3	work	Family	studies	Disasters	lack of land	Return home	Others	Total	Migrants (000s)
**All Rwanda**	**20**	**58**	**3**	**2**	**8**	**6**	**3**	**100**	**2,204**
14-19	19.6	68.9	3.9	1.6	0.5	4.5	1.1	100	294
20-24	27.6	52.3	6.3	1.4	1.9	8.6	1.9	100	292
25-29	32.5	49.5	3.8	1.0	5.7	5.5	2.0	100	301
30-35	29.8	47.8	1.9	1.5	11.2	3.4	4.3	100	248
Male 14-35	38.0	42.9	4.0	1.1	5.0	6.3	2.6	100	541
Female 14-35	17.5	65.8	4.1	1.6	4.2	4.9	1.9	100	593
**All 14–35**	**27.3**	**54.9**	**4.1**	**1.4**	**4.6**	**5.6**	**2.3**	**100**	**1,135**

Source: EICV3

Young people who had moved within the five years that preceded the study were more likely to move for work than previously. These were about 37% in 2010/11 and about 39% in 2013/14 as shown in Table 5.3.

Table 5.3: People who have moved internally in the last five years, by sex, age, and reason (EICV4, EICV3)

EICV4	work	Family	studies	Disasters	lack of land	Return home	Others	Total	Migrants (000s)
All Rwanda	26.7	60.0	3.7	0.4	4.9	0.4	3.9	100	1,356
14-19	34.5	57.3	6.5	0.1	0.0	0.6	1.0	100	201
20-24	41.2	45.5	7.8	0.0	1.4	0.6	3.5	100	235
25-29	39.2	46.2	5.7	0.3	3.9	0.3	4.4	100	195
30-35	39.0	44.3	1.3	1.2	7.6	0.2	6.4	100	154
Male 14-35	51.9	33.4	6.4	0.4	3.2	0.4	4.4	100	371
Female 14-35	26.7	61.9	5.0	0.3	2.6	0.5	3.0	100	414
**All 14–35**	**38.6**	**48.5**	**5.7**	**0.3**	**2.9**	**0.5**	**3.7**	**100**	**785**

Source: EICV4

EICV3	work	Family	studies	Disasters	lack of land	Return home	Others	Total	Migrants (000s)
**All Rwanda**	**25.3**	**58.7**	**4.1**	**0.4**	**7.6**	**0.6**	**3.3**	**100**	**1,063**
14-19	33.4	58.3	6	0.2	0.5	0.8	0.9	100	168
20-24	38.3	48.1	8.5	0.1	2.3	0.4	2.4	100	182
25-29	39.7	45.3	4.1	0.3	7.8	0.5	2.5	100	165
30-35	34.4	41	1.6	0.6	15.5	0.7	6.2	100	105
Male 14-35	49.3	35.7	5.4	0.3	5.6	0.6	3.1	100	290
Female 14-35	25.5	60.6	5.5	0.2	5.4	0.5	2.2	100	330
**All 14–35**	36.7	48.9	5.5	0.2	5.5	0.6	2.6	100	620

Source: EICV3

Annex A. District disaggregation tables for youth population, EICV4

Table A. 1: Youth population by district, EICV4, EICV3 (000s, %)

EICV4	14–19 years	20–24 years	25–29 years	30–35 years	Non-Youth	Total population
**All Rwanda**	**1,507,260**	**1,081,568**	**911,374**	**974,279**	**6,957,831**	**11,507,260**
	**13.2**	**9.5**	**8.0**	**8.5**	**60.9**	**100**
Nyarugenge	47	41	38	35	165	326
	14.4	12.5	11.7	10.8	50.6	100
Gasabo	88	81	76	61	340	645
	13.7	12.5	11.7	9.5	52.6	100
Kicukiro	43	44	42	41	179	348
	12.4	12.6	12.0	11.7	51.4	100
Nyanza	41	32	25	31	216	345
	11.9	9.2	7.2	9.0	62.7	100
Gisagara	43	30	22	27	224	346
	12.4	8.7	6.3	7.7	64.8	100
Nyaruguru	42	27	16	20	185	290
	14.6	9.2	5.4	6.9	63.9	100
Huye	48	31	28	29	215	352
	13.8	8.8	8.0	8.4	61.1	100
Nyamagabe	49	26	23	27	211	335
	14.6	7.8	7.0	7.9	62.8	100
Ruhango	45	23	23	30	220	341
	13.2	6.6	6.7	8.9	64.6	100
Muhanga	37	25	28	29	185	305
	12.2	8.2	9.3	9.5	60.7	100
Kamonyi	40	24	24	29	223	340
	11.8	7.2	7.1	8.5	65.5	100
Karongi	45	33	25	25	214	343
	13.1	9.6	7.4	7.4	62.5	100
Rutsiro	44	29	28	31	202	333
	13.2	8.6	8.3	9.3	60.6	100
Rubavu	59	45	35	36	278	453
	13.1	10.0	7.7	7.9	61.3	100
Nyabihu	40	30	27	22	180	299
	13.2	10.1	9.0	7.4	60.3	100
Ngororero	42	30	27	32	230	362
	11.6	8.4	7.5	8.9	63.6	100
Rusizi	60	46	29	41	270	446
	13.4	10.2	6.6	9.3	60.6	100
Nyamasheke	52	41	30	34	254	410
	12.7	9.9	7.4	8.2	61.8	100
Rulindo	43	25	23	29	189	309
	14.1	8.1	7.3	9.3	61.2	100
Gakenke	46	30	34	29	214	354
	13.1	8.4	9.7	8.3	60.6	100
Musanze	56	37	35	31	236	395
	14.3	9.3	9.0	7.8	59.7	100
Burera	48	32	25	31	215	352
	13.8	9.0	7.2	8.7	61.3	100
Gicumbi	60	37	25	27	244	394
	15.3	9.5	6.4	6.9	61.9	100
Rwamagana	50	39	26	34	245	393
	12.6	9.9	6.5	8.6	62.3	100
Nyagatare	85	46	40	37	331	538

EICV4	14–19 years	20–24 years	25–29 years	30–35 years	Non-Youth	Total population
	15.7	8.5	7.4	6.9	61.5	100
Gatsibo	60	49	32	34	302	477
	12.7	10.3	6.8	7.0	63.2	100
Kayonza	51	40	31	33	249	404
	12.7	9.9	7.8	8.1	61.6	100
Kirehe	49	38	30	30	237	384
	12.9	9.9	7.8	7.8	61.7	100
Ngoma	43	37	27	36	239	382
	11.3	9.8	7.0	9.4	62.6	100
Bugesera	49	35	36	45	266	431
	11.3	8.1	8.3	10.4	61.8	100

Source: EICV4

EICV3	14–19 years	20–24 years	25–29 years	30–35 years	Non-youth	Total population
**All Rwanda**	**1,491**	**1,026**	**885**	**757**	**6,603**	**10,762**
	**13.9**	**9.5**	**8.2**	**7.0**	**61.4**	**100**
Nyarugenge	41	36	35	28	143	282
	14.4	12.7	12.5	9.9	50.5	100
Gasabo	61	59	46	45	264	476
	12.9	12.5	9.7	9.5	55.4	100
Kicukiro	35	38	40	32	156	301
	11.6	12.7	13.2	10.5	52.0	100
Nyanza	40	26	23	18	200	307
	13.1	8.4	7.5	5.8	65.1	100
Gisagara	45	28	27	27	211	337
	13.2	8.2	8.0	7.9	62.7	100
Nyaruguru	48	26	22	18	190	304
	15.9	8.4	7.3	5.8	62.6	100
Huye	42	31	24	21	201	319
	13.2	9.7	7.5	6.5	63.2	100
Nyamagabe	47	27	24	18	214	330
	14.1	8.3	7.2	5.4	64.9	100
Ruhango	39	26	28	19	193	304
	12.8	8.5	9.1	6.2	63.4	100
Muhanga	39	26	22	24	186	297
	13.3	8.8	7.3	8.0	62.6	100
Kamonyi	44	26	26	22	211	329
	13.4	8.0	7.8	6.8	64.0	100
Karongi	53	33	24	23	221	354
	15.1	9.2	6.7	6.5	62.5	100
Rutsiro	41	27	29	26	203	326
	12.5	8.4	8.9	8.1	62.1	100
Rubavu	58	41	33	32	259	423
	13.7	9.8	7.7	7.5	61.3	100
Nyabihu	50	37	22	21	199	331
	15.3	11.3	6.6	6.5	60.4	100
Ngororero	43	30	29	24	216	341
	12.5	8.7	8.6	6.9	63.3	100
Rusizi	68	43	27	26	254	417
	16.2	10.3	6.4	6.1	60.9	100
Nyamasheke	58	35	34	27	240	393
	14.8	8.9	8.7	6.8	60.9	100
Rulindo	40	24	22	23	184	294
	13.5	8.3	7.6	7.9	62.7	100

EICV3	14–19 years	20–24 years	25–29 years	30–35 years	Non-youth	Total population
Gakenke	40	32	36	27	210	345
	11.7	9.3	10.4	7.7	60.9	100
Musanze	58	40	39	26	253	416
	13.9	9.6	9.5	6.2	60.8	100
Burera	53	34	21	21	225	354
	14.9	9.5	6.1	5.9	63.6	100
Gicumbi	91	66	46	30	340	572
	15.8	11.6	8.0	5.2	59.4	100
Rwamagana	44	33	23	26	193	318
	13.7	10.2	7.3	8.1	60.6	100
Nyagatare	59	36	30	28	270	424
	14.0	8.6	7.1	6.6	63.7	100
Gatsibo	68	44	40	30	310	491
	13.9	8.9	8.1	6.0	63.1	100
Kayonza	46	30	27	24	205	332
	13.9	9.1	8.0	7.1	61.9	100
Kirehe	44	32	30	20	203	329
	13.4	9.6	9.1	6.2	61.8	100
Ngoma	43	29	25	25	201	323
	13.4	8.8	7.7	7.7	62.4	100
Bugesera	52	32	32	30	245	391
	13.4	8.1	8.1	7.7	62.7	100

Source: EICV3

Table A.2: Youth population by district, Male, EICV4, EICV3 (000s, %)

EICV4	14–19 years	20–24 years	25–29 years	30–35 years	Non-youth	Total population
**All Rwanda**	**738,748**	**518,651**	**427,370**	**457,272**	**3,323,180**	**5,465,222**
	**13.5**	**9.5**	**7.8**	**8.4**	**60.8**	**100**
Nyarugenge	21	20	21	20	85	166
	12.4	11.9	12.4	12.1	51.2	100
Gasabo	43	37	38	32	170	319
	13.3	11.6	11.8	10.1	53.16	100
Kicukiro	18	20	20	20	83	161
	11.4	12.7	12.4	12.2	51.3	100
Nyanza	21	15	12	15	98	162
	13.3	9.6	7.2	9.4	60.6	100
Gisagara	23	14	11	12	103	163
	14.4	8.5	6.8	7.4	62.9	100
Nyaruguru	22	12	6	11	88	139
	15.7	8.6	4.3	8.2	63.2	100
Huye	23	18	11	16	101	169
	13.7	10.6	6.7	9.3	59.7	100
Nyamagabe	24	12	12	11	98	157
	15.4	7.7	7.5	7.2	62.3	100
Ruhango	23	11	10	15	105	164
	14.0	6.8	6.4	9.2	63.6	100
Muhanga	18	12	14	13	87	144
	12.6	8.4	9.6	8.9	60.5	100
Kamonyi	20	11	11	13	106	161
	12.3	7.1	6.9	8.1	65.6	100
Karongi	20	16	11	11	104	162
	12.6	10.1	6.5	7.0	63.7	100
Rutsiro	22	14	12	13	96	157

EICV4	14–19 years	20–24 years	25–29 years	30–35 years	Non-youth	Total population
	13.9	8.9	7.8	8.2	61.3	100
Rubavu	29	21	14	19	135	217
	13.1	9.8	6.6	8.6	61.9	100
Nyabihu	18	14	13	9	89	143
	12.3	10.0	9.1	6.6	62.0	100
Ngororero	20	11	12	14	114	172
	11.9	6.4	7.2	8.3	66.1	100
Rusizi	29	22	14	19	127	211
	13.7	10.4	6.7	8.8	60.3	100
Nyamasheke	25	18	13	14	117	187
	13.2	9.5	6.9	7.7	62.8	100
Rulindo	22	12	11	13	88	146
	15.3	8.2	7.3	8.8	60.5	100
Gakenke	21	14	15	13	107	170
	12.4	8.4	8.7	7.6	62.8	100
Musanze	27	18	18	12	109	184
	14.6	9.9	9.7	6.7	59.0	100
Burera	25	14	12	16	103	170
	14.8	8.5	7.0	9.2	60.5	100
Gicumbi	31	18	12	13	117	191
	16.2	9.6	6.2	6.9	61.1	100
Rwamagana	21	19	11	15	114	180
	11.5	10.5	6.25	8.26	63.5	100
Nyagatare	44	26	20	16	155	261
	17	10.1	7.6	6.0	59.4	100
Gatsibo	30	24	17	15	150	235
	12.7	10.0	7.1	6.4	63.8	100
Kayonza	27	20	16	15	120	199
	13.5	10.1	8.2	7.7	60.6	100
Kirehe	23	21	13	14	116	187
	12.3	11.4	7.0	7.4	61.8	100
Ngoma	24	16	12	17	113	182
	13.1	8.8	6.6	9.5	62.1	100
Bugesera	25	16	16	20	129	206
	12.0	7.6	7.9	10.0	62.6	100

Source: EICV4

EICV3	14–19 years	20–24 years	25–29 years	30–35 years	Non-youth	Total population
**All Rwanda**	**739,645**	**479,434**	**407,886**	**344,369**	**3,133,610**	**5,104,945**
	**14.5**	**9.4**	**8.0**	**6.8**	**61.4**	**100**
Nyarugenge	16	16	18	16	69	135
	12.0	11.8	13.4	12.0	50.8	100
Gasabo	27	28	23	23	130	231
	11.7	12.1	9.9	10.1	56.3	100
Kicukiro	15	19	21	16	79	151
	10.1	12.5	14.3	10.8	52.4	100
Nyanza	20	14	12	6	92	145
	13.5	9.7	8.4	4.5	63.9	100
Gisagara	23	14	13	12	100	162
	14.4	8.5	7.9	7.3	62.0	100
Nyaruguru	26	13	11	8	87	144
	17.8	8.9	7.4	5.5	60.4	100
Huye	20	15	11	10	92	147
	13.4	10.1	7.8	6.6	62.1	100

EICV3	14–19 years	20–24 years	25–29 years	30–35 years	Non-youth	Total population
Nyamagabe	23	14	10	9	101	156
	14.7	8.7	6.4	5.7	64.5	100
Ruhango	19	13	13	8	88	141
	13.7	9.1	9.4	5.5	62.3	100
Muhanga	19	13	9	10	87	137
	13.6	9.3	6.5	7.0	63.7	100
Kamonyi	22	14	11	11	100	159
	13.9	8.9	7.1	6.9	63.4	100
Karongi	27	17	10	10	101	165
	16.3	10.1	6.2	5.8	61.6	100
Rutsiro	22	12	11	12	98	155
	14.1	7.5	7.4	7.8	63.3	100
Rubavu	29	17	14	15	119	194
	15.1	9.0	7.1	7.7	61.1	100
Nyabihu	25	17	10	9	96	157
	16.2	10.6	6.5	6.0	60.8	100
Ngororero	20	14	12	11	106	163
	12	8.6	7.6	6.6	65.3	100
Rusizi	34	21	12	11	120	198
	17.3	10.5	6.0	5.5	60.7	100
Nyamasheke	29	13	15	11	108	177
	16.1	7.3	8.7	6.4	61.4	100
Rulindo	17	10	10	10	92	139
	12.1	7.3	7.0	7.1	66.6	100
Gakenke	20	15	15	11	99	160
	12.4	9.4	9.3	7.1	61.8	100
Musanze	28	19	16	13	115	191
	14.7	9.9	8.6	6.6	60.2	100
Burera	27	16	10	9	104	167
	16.2	9.8	5.9	5.7	62.5	100
Gicumbi	42	29	25	14	164	274
	15.2	10.7	9.1	5.1	59.9	100
Rwamagana	22	18	11	12	89	152
	14.6	12.2	7.2	7.9	58.2	100
Nyagatare	33	17	12	12	135	209
	15.7	8.3	5.6	5.8	64.5	100
Gatsibo	39	18	19	12	148	236
	16.4	7.7	8.1	5.2	62.6	100
Kayonza	23	13	13	11	98	158
	14.7	8.5	8.2	6.7	62.1	100
Kirehe	24	15	14	8	100	162
	15.0	9.3	8.5	5.2	62.0	100
Ngoma	24	13	10	12	93	152
	15.5	8.5	6.7	8.0	61.3	100
Bugesera	26	13	16	12	123	190
	13.8	7.1	8.4	6.2	64.6	100

Source: EICV3

Table A.3: Youth population by district, Female, EICV4, EICV3 (000s, %)

EICV4	14–19 years	20–24 years	25–29 years	30–35 years	Non-youth	Total population
**All Rwanda**	**768,512**	**562,917**	**484,004**	**517,007**	**3,634,651**	**5,967,091**
	**12.9**	**9.4**	**8.1**	**8.7**	**60.9**	**100**
Nyarugenge	26	21	17	15	80	160
	16	13	11	10	50	100
Gasabo	46	44	38	29	170	326
	14.1	13.4	11.6	8.9	52.1	100
Kicukiro	25	23	22	21	96	187
	13.2	12.6	11.6	11.2	51.4	100
Nyanza	20	16	13	16	118	183
	10.8	8.9	7.1	8.7	64.5	100
Gisagara	19	16	11	15	122	183
	10.6	8.9	5.9	8.0	66.5	100
Nyaruguru	20	15	10	9	98	151
	13.5	9.8	6.4	5.7	64.6	100
Huye	25	13	17	14	114	183
	14	7	9	7	62	100
Nyamagabe	25	14	12	15	113	178
	13.8	7.9	6.5	8.6	63.2	100
Ruhango	22	11	12	15	115	176
	12.4	6.4	7.1	8.6	65.5	100
Muhanga	19	13	15	16	99	162
	11.9	8.1	9.0	10.1	61.0	100
Kamonyi	20	13	13	16	117	179
	11.3	7.3	7.3	8.7	65.3	100
Karongi	24	16	15	14	111	180
	13.5	9.1	8.2	7.8	61.5	100
Rutsiro	22	15	16	18	106	176
	12.6	8.4	8.8	10.2	60.0	100
Rubavu	31	24	21	17	143	236
	13.1	10.2	8.8	7.2	60.8	100
Nyabihu	22	16	14	13	92	156
	14.1	10.1	8.8	8.2	58.8	100
Ngororero	21	19	15	18	117	190
	11.2	10.2	7.8	9.5	61.4	100
Rusizi	31	24	15	23	143	235
	13.0	10.1	6.4	9.6	60.8	100
Nyamasheke	27	23	18	19	137	224
	12.2	10.3	7.9	8.6	61.0	100
Rulindo	21	13	12	16	101	163
	12.9	8.1	7.3	9.8	61.9	100
Gakenke	25	15	19	16	107	183
	13.6	8.4	10.6	8.8	58.5	100
Musanze	29	18	18	18	127	210
	14	8.7	8.4	8.7	60.2	100
Burera	23	17	14	15	112	181
	12.8	9.6	7.5	8.2	62.0	100
Gicumbi	30	19	14	14	128	204
	14.6	9.3	6.7	6.8	62.6	100
Rwamagana	29	20	14	19	131	214
	13.5	9.5	6.8	8.9	61.3	100
Nyagatare	40	19	20	21	176	277
	14.5	7.0	7.3	7.7	63.5	100
Gatsibo	31	26	16	19	152	242

EICV4	14–19 years	20–24 years	25–29 years	30–35 years	Non-youth	Total population
	12.6	10.7	6.5	7.7	62.6	100
Kayonza	24	20	15	17	128	205
	11.9	9.7	7.4	8.5	62.7	100
Kirehe	26	17	17	16	121	197
	13.4	8.4	8.5	8.2	61.5	100
Ngoma	19	21	15	19	126	200
	9.6	10.7	7.4	9.3	63.0	100
Bugesera	24	19	20	24	137	225
	10.8	8.6	8.7	10.9	61.1	100

Source: EICV4

EICV3	14–19 years	20–24 years	25–29 years	30–35 years	Non-youth	Total population
**All Rwanda**	**751,066**	**546,722**	**477,322**	**412,539**	**3,469,490**	**5,657,139**
	**13.3**	**9.7**	**8.4**	**7.3**	**61.3**	**100**
Nyarugenge	25	20	17	12	74	147
	16.6	13.5	11.7	8.0	50.2	100
Gasabo	34	31	24	22	134	246
	14.0	12.8	9.6	9.0	54.6	100
Kicukiro	20	19	18	15	77	150
	13.1	12.9	12.2	10.2	51.6	100
Nyanza	21	12	11	11	107	162
	12.8	7.2	6.8	7.0	66.2	100
Gisagara	21	14	14	15	111	175
	12.2	8.0	8.2	8.4	63.3	100
Nyaruguru	23	13	12	10	104	160
	14.2	8.0	7.2	6.0	64.7	100
Huye	22	16	12	11	110	172
	13.0	9.3	7.2	6.4	64.1	100
Nyamagabe	24	14	14	9	114	174
	13.6	7.9	8.0	5.2	65.3	100
Ruhango	20	13	15	11	105	163
	12.0	8.1	8.9	6.7	64.4	100
Muhanga	21	13	13	14	99	160
	13.0	8.4	8.1	8.8	61.7	100
Kamonyi	22	12	15	11	110	171
	12.9	7.3	8.6	6.7	64.6	100
Karongi	27	16	13	14	120	190
	14	8.4	7.1	7.2	63.3	100
Rutsiro	19	16	18	14	104	171
	11.1	9.2	10.3	8.4	61.0	100
Rubavu	29	24	19	17	141	229
	12.6	10.4	8.3	7.3	61.5	100
Nyabihu	25	21	11	12	104	173
	14.5	12.0	6.6	6.9	60.0	100
Ngororero	23	16	17	13	110	179
	13.0	8.8	9.6	7.2	61.4	100
Rusizi	33	22	15	15	134	219
	15.2	10.2	6.8	6.7	61.2	100
Nyamasheke	30	22	19	15	131	217
	13.6	10.1	8.7	7.0	60.5	100
Rulindo	23	14	13	13	92	155
	14.7	9.2	8.2	8.6	59.3	100
Gakenke	20	17	21	15	111	185

EICV3	14–19 years	20–24 years	25–29 years	30–35 years	Non-youth	Total population
	11.1	9.3	11.3	8.3	60.1	100
Musanze	30	21	23	13	138	225
	13.2	9.3	10.3	5.9	61.3	100
Burera	26	17	12	12	121	187
	13.7	9.2	6.3	6.2	64.7	100
Gicumbi	49	37	21	16	175	298
	16.5	12.4	7.1	5.2	58.9	100
Rwamagana	22	14	12	14	104	166
	13.0	8.5	7.4	8.4	62.8	100
Nyagatare	26	19	18	16	136	215
	12.3	8.9	8.5	7.4	62.9	100
Gatsibo	29	26	20	17	163	255
	11.5	10.1	8.0	6.8	63.7	100
Kayonza	23	17	14	13	107	174
	13.2	9.6	7.9	7.5	61.7	100
Kirehe	20	16	16	12	103	167
	11.8	9.9	9.6	7.1	61.6	100
Ngoma	20	16	15	13	108	171
	11.6	9.2	8.6	7.3	63.4	100
Bugesera	26	18	16	19	122	201
	13.0	9.1	7.8	9.2	60.9	100

Source: EICV3

Annex B. Confidence intervals for selected indicators from EICV4

Table B. 1: Percentage of total young population aged 14 to 35 years who have never been to school, by domain

Domain	Estimate (%)	Standard Error	95% Confidence Interval		CV	Design Effect	No. of observations
			Lower	Upper			
**All Rwanda**	5.9	0.2	5.6	6.3	2.947	1.4	25,743
**Urban/rural**							
Urban	3.0	0.30	2.4	3.6	9.909	1.7	5,062
Rural	6.7	0.21	6.3	7.1	3.071	1.4	20,681
**Province**							
Kigali City	2.2	0.29	1.6	2.8	13.169	1.4	3,006
Southern Province	5.9	0.33	5.3	6.6	5.510	1.1	6,332
Western Province	7.8	0.43	7.0	8.7	5.511	1.5	6,158
Northern Province	5.3	0.40	4.5	6.1	7.655	1.3	4,323
Eastern Province	6.8	0.39	6.0	7.5	5.827	1.6	5,924
**Age (in years)**							
14-19	1.7	0.15	1.4	2.0	8.878	1.2	8,701
20-24	4.5	0.29	3.9	5.0	6.405	1.2	6,185
25-29	7.9	0.38	7.1	8.6	4.774	1.0	5,225
30-35	12.3	0.50	11.4	13.3	4.067	1.3	5,632
**Quintile**							
Q1	9.6	0.51	8.6	10.6	5.268	1.2	4,169
Q2	7.3	0.41	6.5	8.1	5.677	1.1	4,655
Q3	6.3	0.40	5.6	7.1	6.242	1.3	4,923
Q4	4.8	0.32	4.2	5.4	6.726	1.2	5,367
Q5	3.4	0.26	2.9	3.9	7.404	1.3	6,628
**Districts**							
Nyarugenge	2.4	0.50	1.4	3.4	21.227	1.0	1,007
Gasabo	2.0	0.42	1.2	2.8	20.991	1.6	964
Kicukiro	2.4	0.61	1.2	3.6	25.541	1.5	1,035
Nyanza	7.2	1.14	4.9	9.4	15.951	1.5	809
Gisagara	9.2	1.22	6.8	11.6	13.224	1.2	757
Nyaruguru	7.8	0.94	5.9	9.6	12.046	0.7	845
Huye	5.5	0.88	3.8	7.2	16.108	1.2	836
Nyamagabe	4.6	0.76	3.1	6.1	16.461	0.9	822
Ruhango	6.8	0.90	5.1	8.6	13.196	0.9	757
Muhanga	3.3	0.67	1.9	4.6	20.630	1.0	790
Kamonyi	3.3	0.67	2.0	4.6	20.358	1.0	716
Karongi	4.6	0.71	3.2	6.0	15.317	0.8	832
Rutsiro	11.2	1.28	8.7	13.7	11.432	1.2	870
Rubavu	10.4	1.29	7.9	13.0	12.364	1.8	924
Nyabihu	8.6	1.03	6.6	10.6	12.052	0.9	898
Ngororero	8.2	1.33	5.6	10.8	16.300	1.8	790
Rusizi	5.8	1.20	3.4	8.1	20.679	2.7	965

Domain	Estimate (%)	Standard Error	95% Confidence Interval		CV	Design Effect	No. of observations
			Lower	Upper			
Nyamasheke	6.1	0.77	4.6	7.6	12.630	0.9	879
Rulindo	4.8	0.87	3.1	6.5	18.184	1.1	843
Gakenke	4.5	0.77	2.9	6.0	17.269	1.1	843
Musanze	4.6	0.83	3.0	6.2	18.085	1.4	902
Burera	7.6	1.18	5.2	9.9	15.675	1.6	886
Gicumbi	5.0	0.82	3.4	6.6	16.356	1.2	849
Rwamagana	4.3	0.95	2.4	6.2	22.063	1.9	813
Nyagatare	6.5	0.84	4.8	8.1	12.974	1.4	894
Gatsibo	6.4	1.08	4.3	8.6	16.822	2.0	800
Kayonza	6.6	0.95	4.8	8.5	14.246	1.3	868
Kirehe	8.9	1.22	6.6	11.3	13.627	1.5	840
Ngoma	7.9	1.24	5.4	10.3	15.699	1.7	854
Bugesera	6.9	0.93	5.0	8.7	13.572	1.3	855

Source: EICV4

Table B.2: Percentage of total young population aged 14 to 35 years who did not complete primary, by domain

Domain	Estimate (%)	Standard Error	95% Confidence Interval		CV	Design Effect	No. of observations
			Lower	Upper			
**All Rwanda**	50.2	0.5	2.6	1.6	0.999	2.6	25,743
**Urban/rural**							
Urban	31.1	1.05	29.1	33.2	3.363	2.8	5,062
Rural	55.3	0.48	54.4	56.3	0.870	1.9	20,681
**Province**							
Kigali City	32.5	1.60	29.3	35.6	4.938	4.3	3,006
Southern Province	52.0	0.96	50.1	53.8	1.840	2.1	6,332
Western Province	54.6	0.97	52.7	56.5	1.770	2.2	6,158
Northern Province	51.0	1.13	48.8	53.3	2.213	2.1	4,323
Eastern Province	54.2	1.02	52.2	56.2	1.876	2.7	5,924
**Age (in years)**							
14-19	56.3	0.68	54.9	57.6	1.211	1.6	8,701
20-24	41.2	0.81	39.7	42.8	1.973	1.7	6,185
25-29	51.9	0.86	50.2	53.6	1.661	1.6	5,225
30-35	49.3	0.82	47.7	50.9	1.659	1.5	5,632
**Quintile**							
Q1	66.7	0.84	65.0	68.3	1.264	1.3	4,169
Q2	59.5	0.89	57.8	61.3	1.486	1.5	4,655
Q3	55.1	0.87	53.4	56.9	1.575	1.5	4,923
Q4	48.6	0.87	46.9	50.3	1.798	1.6	5,367
Q5	31.8	0.76	30.3	33.3	2.398	1.8	6,628
**Districts**							
Nyarugenge	28.6	2.45	23.8	33.4	8.559	2.7	1,007
Gasabo	36.6	2.95	30.9	42.4	8.054	6.6	964
Kicukiro	28.6	1.87	25.0	32.3	6.516	1.7	1,035
Nyanza	51.2	2.71	45.9	56.5	5.287	2.2	809
Gisagara	58.8	2.19	54.5	63.0	3.720	1.4	757
Nyaruguru	54.8	2.53	49.9	59.8	4.606	1.5	845
Huye	46.9	2.65	41.6	52.1	5.665	2.2	836
Nyamagabe	55.1	2.35	50.5	59.7	4.273	1.6	822

Domain	Estimate (%)	Standard Error	95% Confidence Interval		CV	Design Effect	No. of observations
			Lower	Upper			
Ruhango	49.8	2.53	44.8	54.8	5.089	1.8	757
Muhanga	47.8	3.39	41.1	54.4	7.092	3.2	790
Kamonyi	52.4	2.77	46.9	57.8	5.295	2.1	716
Karongi	55.4	2.11	51.3	59.5	3.803	1.3	832
Rutsiro	57.7	1.82	54.1	61.3	3.156	1.0	870
Rubavu	48.8	3.32	42.3	55.3	6.797	4.4	924
Nyabihu	53.5	2.43	48.7	58.2	4.535	1.6	898
Ngororero	58.4	2.53	53.5	63.4	4.327	2.0	790
Rusizi	51.9	2.37	47.2	56.5	4.562	2.3	965
Nyamasheke	58.5	2.18	54.3	62.8	3.725	1.8	879
Rulindo	45.1	2.44	40.3	49.9	5.418	1.7	843
Gakenke	45.3	2.28	40.8	49.8	5.042	1.7	843
Musanze	55.5	2.97	49.7	61.3	5.349	3.3	902
Burera	57.3	2.30	52.8	61.8	4.013	1.7	886
Gicumbi	50.6	2.36	46.0	55.2	4.667	1.9	849
Rwamagana	47.9	2.39	43.2	52.6	4.993	2.0	813
Nyagatare	55.5	2.63	50.4	60.7	4.733	3.3	894
Gatsibo	53.0	2.37	48.3	57.6	4.466	2.3	800
Kayonza	56.5	2.67	51.3	61.7	4.732	2.6	868
Kirehe	54.2	2.67	48.9	59.4	4.924	2.4	840
Ngoma	58.3	2.45	53.4	63.1	4.213	2.0	854
Bugesera	53.9	3.13	47.8	60.0	5.811	3.7	855

Source: EICV4

Table B.3: Percentage of total young population age 14 to 35 years who completed primary, by domain

Domain	Estimate (%)	Standard Error	95% Confidence Interval		CV	Design Effect	No. of observations
			Lower	Upper			
**All Rwanda**	43.7	0.5	42.6	44.7	1.244	3.1	25,743
**Urban/rural**							
Urban	65.7	1.13	63.5	67.9	1.721	3.1	5,062
Rural	37.8	0.52	36.8	38.8	1.370	2.3	20,681
**Province**							
Kigali City	65.3	1.62	62.1	68.4	2.483	4.2	3,006
Southern Province	42.0	1.03	39.9	44.0	2.459	2.5	6,332
Western Province	37.3	1.11	35.1	39.5	2.988	3.1	6,158
Northern Province	43.6	1.22	41.2	46.0	2.793	2.4	4,323
Eastern Province	38.8	1.10	36.7	41.0	2.843	3.4	5,924
**Age (in years)**							
14-19	41.9	0.69	40.5	43.2	1.636	1.7	8,701
20-24	54.1	0.85	52.4	55.8	1.574	1.8	6,185
25-29	40.0	0.90	38.2	41.7	2.255	1.8	5,225
30-35	38.4	0.85	36.7	40.1	2.217	1.7	5,632
**Quintile**							
Q1	23.5	0.83	1.6	1.3	3.516	1.6	4,169
Q2	33.1	0.89	1.6	1.3	2.683	1.6	4,655
Q3	38.3	0.90	1.7	1.3	2.353	1.7	4,923
Q4	46.5	0.92	1.8	1.4	1.988	1.8	5,367
Q5	64.6	0.81	2.0	1.4	1.261	2.0	6,628
**Districts**							
Nyarugenge	69.0	2.54	64.0	74.0	3.676	2.8	1,007
Gasabo	61.4	2.95	55.6	67.1	4.800	6.4	964

Domain	Estimate (%)	Standard Error	95% Confidence Interval		CV	Design Effect	No. of observations
			Lower	Upper			
Kicukiro	68.8	2.01	64.8	72.7	2.922	1.8	1,035
Nyanza	41.1	3.06	35.1	47.1	7.432	2.9	809
Gisagara	32.1	2.37	27.4	36.7	7.393	1.8	757
Nyaruguru	37.4	2.72	32.0	42.7	7.288	1.9	845
Huye	47.7	2.89	42.0	53.3	6.062	2.6	836
Nyamagabe	40.3	2.50	35.4	45.2	6.217	1.9	822
Ruhango	42.8	2.79	37.4	48.3	6.513	2.2	757
Muhanga	49.0	3.51	42.1	55.9	7.175	3.4	790
Kamonyi	44.3	2.88	38.7	50.0	6.496	2.3	716
Karongi	40.0	2.12	35.8	44.1	5.300	1.4	832
Rutsiro	30.6	2.18	26.3	34.9	7.142	1.7	870
Rubavu	40.8	3.91	33.1	48.5	9.594	6.4	924
Nyabihu	37.5	2.73	32.1	42.9	7.291	2.2	898
Ngororero	33.4	3.02	27.5	39.3	9.055	3.1	790
Rusizi	41.5	2.85	35.9	47.1	6.873	3.4	965
Nyamasheke	35.2	2.23	30.9	39.6	6.326	2.0	879
Rulindo	50.0	2.51	45.1	54.9	5.022	1.7	843
Gakenke	50.2	2.47	45.4	55.1	4.916	2.0	843
Musanze	39.7	3.18	33.4	45.9	8.011	3.9	902
Burera	35.1	2.60	30.0	40.2	7.398	2.3	886
Gicumbi	44.4	2.60	39.3	49.5	5.862	2.4	849
Rwamagana	47.8	2.72	42.5	53.1	5.681	2.5	813
Nyagatare	37.8	2.75	32.5	43.2	7.256	3.8	894
Gatsibo	40.5	2.50	35.5	45.4	6.190	2.6	800
Kayonza	36.8	2.77	31.3	42.2	7.534	2.9	868
Kirehe	36.3	3.14	30.2	42.5	8.641	3.6	840
Ngoma	33.5	2.56	28.5	38.6	7.647	2.4	854
Bugesera	39.1	3.29	32.7	45.6	8.399	4.3	855

Source: EICV4

Table B.4: Literacy rate for young male population age 14 to 35 years, by domain

Domain	Estimate (%)	Standard Error	95% Confidence Interval		CV	Design Effect	No. of observations
			Lower	Upper			
**All Rwanda**	81.7	0.4	80.9	82.5	0.503	1.4	12,311
**Urban/rural**							
Urban	92.3	0.67	91.0	93.6	0.722	1.7	2,449
Rural	78.8	0.47	77.9	79.7	0.593	1.3	9,862
**Province**							
Kigali City	93.5	0.86	91.8	95.2	0.922	2.2	1455
Southern Province	77.8	0.86	76.2	79.5	1.106	1.2	3081
Western Province	80.2	0.89	78.4	81.9	1.108	1.3	2843
Northern Province	82.6	0.96	80.7	84.5	1.162	1.3	2071
Eastern Province	79.2	0.87	77.5	80.9	1.104	1.5	2861
**Age (in years)**							
14-19	81.7	0.65	80.5	83.0	0.795	1.2	4,276
20-24	85.6	0.70	84.3	87.0	0.822	1.2	2,956
25-29	80.8	0.84	79.1	82.4	1.041	1.1	2,440
30-35	78.1	0.84	76.4	79.8	1.079	1.1	2,639
**Quintile**							
Q1	69.3	1.20	66.9	71.6	1.729	1.2	1,853
Q2	78.7	0.93	76.9	80.5	1.185	1.1	2,125

Domain	Estimate (%)	Standard Error	95% Confidence Interval		CV	Design Effect	No. of observations
			Lower	Upper			
Q3	80.0	0.89	78.2	81.7	1.107	1.1	2,348
Q4	84.2	0.76	82.7	85.7	0.902	1.1	2,636
Q5	89.4	0.59	88.2	90.6	0.662	1.3	3,348
**Districts**							
Nyarugenge	91.9	1.70	88.6	95.3	1.852	1.8	504
Gasabo	94.0	1.37	91.3	96.7	1.458	2.9	470
Kicukiro	94.1	1.24	91.7	96.6	1.320	1.3	481
Nyanza	79.7	2.54	74.7	84.7	3.193	1.5	399
Gisagara	73.8	2.40	69.1	78.5	3.251	1.0	379
Nyaruguru	74.4	2.25	70.0	78.8	3.031	0.8	409
Huye	77.9	2.73	72.6	83.3	3.499	1.7	416
Nyamagabe	81.4	2.10	77.3	85.6	2.584	1.0	390
Ruhango	75.5	2.10	71.3	79.6	2.779	0.8	375
Muhanga	83.0	2.60	77.9	88.1	3.138	1.6	375
Kamonyi	76.6	2.34	72.1	81.2	3.050	1.0	338
Karongi	79.1	2.16	74.9	83.4	2.736	1.0	381
Rutsiro	80.5	2.26	76.1	84.9	2.808	1.1	402
Rubavu	85.2	2.05	81.1	89.2	2.412	1.6	438
Nyabihu	81.0	2.08	76.9	85.1	2.575	0.9	412
Ngororero	70.6	2.87	65.0	76.2	4.063	1.3	350
Rusizi	81.9	2.44	77.1	86.7	2.987	1.9	469
Nyamasheke	80.2	2.11	76.0	84.3	2.631	1.1	391
Rulindo	82.9	2.39	78.2	87.6	2.884	1.3	405
Gakenke	82.3	2.28	77.8	86.7	2.767	1.3	383
Musanze	82.6	2.09	78.5	86.7	2.535	1.3	425
Burera	80.9	2.13	76.7	85.1	2.628	1.1	440
Gicumbi	84.2	1.87	80.5	87.8	2.224	1.1	418
Rwamagana	82.0	1.91	78.2	85.7	2.330	0.9	359
Nyagatare	84.4	1.96	80.5	88.2	2.326	1.8	459
Gatsibo	81.4	2.18	77.1	85.6	2.677	1.5	384
Kayonza	82.6	2.50	77.7	87.5	3.030	2.0	440
Kirehe	79.1	2.62	73.9	84.2	3.311	1.7	410
Ngoma	61.2	2.94	55.4	66.9	4.805	1.4	412
Bugesera	79.9	1.90	76.2	83.6	2.374	1.0	397

Source: EICV4

Table B.5: Literacy rate for young female population age 14 to 35 years, by domain

Domain	Estimate (%)	Standard Error	95% Confidence Interval		CV	Design Effect	No. of observations
			Lower	Upper			
**All Rwanda**	81.3	0.4	80.5	82.2	0.518	1.6	13,432
**Urban/rural**							
Urban	90.8	0.71	89.4	92.2	0.777	1.7	2,613
Rural	78.8	0.48	77.9	79.8	0.615	1.5	10,819
**Province**							
Kigali City	91.4	0.84	89.8	93.1	0.923	1.7	1,551
Southern Province	82.1	0.84	80.4	83.7	1.025	1.4	3,251
Western Province	77.9	1.00	75.9	79.9	1.287	1.8	3,315
Northern Province	82.6	0.95	80.7	84.5	1.150	1.3	2,252
Eastern Province	77.5	0.90	75.7	79.3	1.166	1.6	3,063
**Age (in years**							
14-19	88.2	0.56	87.1	89.3	0.633	1.3	4,425
20-24	84.8	0.68	83.5	86.1	0.800	1.2	3,229
25-29	75.9	0.85	74.3	77.6	1.124	1.1	2,785

Domain	Estimate (%)	Standard Error	95% Confidence Interval		CV	Design Effect	No. of observations
			Lower	Upper			
30-35	72.3	0.90	70.5	74.1	1.249	1.2	2,993
**Quintile**							
Q1	67.0	1.13	64.7	69.2	1.682	1.3	2,316
Q2	76.8	0.95	74.9	78.6	1.234	1.2	2,530
Q3	81.0	0.84	79.3	82.6	1.041	1.2	2,575
Q4	85.0	0.76	83.5	86.5	0.896	1.3	2,731
Q5	91.6	0.52	90.6	92.6	0.565	1.2	3,280
**Districts**							
Nyarugenge	90.3	1.48	87.3	93.2	1.639	1.1	503
Gasabo	91.9	1.35	89.3	94.6	1.465	2.2	494
Kicukiro	91.5	1.45	88.7	94.4	1.584	1.4	554
Nyanza	80.6	2.61	75.5	85.7	3.235	1.6	410
Gisagara	77.3	2.98	71.4	83.1	3.855	1.8	378
Nyaruguru	77.8	2.14	73.6	82.0	2.754	0.8	436
Huye	86.3	2.09	82.2	90.4	2.421	1.5	420
Nyamagabe	83.9	1.95	80.1	87.7	2.318	1.1	432
Ruhango	81.7	2.66	76.5	87.0	3.260	1.7	382
Muhanga	85.8	2.15	81.6	90.0	2.500	1.4	415
Kamonyi	81.8	2.21	77.4	86.1	2.700	1.2	378
Karongi	82.3	1.84	78.6	85.9	2.241	0.9	451
Rutsiro	75.1	2.73	69.8	80.5	3.631	1.6	468
Rubavu	73.3	3.08	67.2	79.3	4.209	2.6	486
Nyabihu	81.5	2.28	77.0	86.0	2.795	1.3	486
Ngororero	74.5	3.30	68.0	80.9	4.437	2.4	440
Rusizi	81.2	2.45	76.4	86.0	3.019	2.1	496
Nyamasheke	78.2	2.14	74.0	82.4	2.737	1.4	488
Rulindo	86.8	1.98	82.9	90.7	2.276	1.2	438
Gakenke	85.6	1.97	81.8	89.5	2.306	1.4	460
Musanze	81.3	2.04	77.3	85.3	2.511	1.3	477
Burera	76.1	2.37	71.5	80.8	3.117	1.2	446
Gicumbi	83.5	2.15	79.3	87.7	2.580	1.5	431
Rwamagana	87.5	1.25	85.0	89.9	1.435	0.7	454
Nyagatare	75.0	2.59	69.9	80.1	3.453	2.1	435
Gatsibo	80.3	1.99	76.4	84.2	2.482	1.3	416
Kayonza	81.3	1.88	77.6	85.0	2.306	1.0	428
Kirehe	74.6	2.94	68.8	80.4	3.940	2.0	430
Ngoma	59.3	3.09	53.2	65.4	5.218	1.7	442
Bugesera	82.6	2.13	78.4	86.8	2.584	1.6	458

Source: EICV4

EICV4 Staff

EICV4 National Coordination

Yusuf Murangwa

EICV4 Technical Coordination

Dominique Habimana

EICV4 Field Coordination

Juvenal Munyarugerero

EICV4 Youth analysis

- Nicolas MWIZERWA, Statistician -NISR

EICV4 data processing

- Mario Vaisman
- Juste Nitiema

EICV4 Proof reading, designing and copy-editing

- Jean Claude NYIRIMANZI
- Ruben MUHAYITETO
- Denise UWAMARIYA
- Dr Joseph NSENGIYUMVA

EICV4 – Fieldwork Supervision

- Mugabo Jean
- Kamana Roger
- Mwizerwa Nicolas
- Nzabonimpa Jean Claude
- Segahwege Astrid
- Serugendo Jean Baptiste

EICV4 Staff - Centre Zone

Province	Number of Center Staff
**Kigali City Zone**	
Nyarugenge	7
Gasabo	7
Kicukiro	7
**Southern Zone**	
Nyanza	6
Gisagara	6
Nyaruguru	6
Huye	6
Nyamagabe	6
Ruhango	6
Muhanga	6
Kamonyi	6
**Nothern Zone**	
Rulindo	6
Gakenke	6
Musanze	6
Burera	6
Gicumbi	6

Province	Number of Center Staff
**Western Zone**	
Karongi	6
Rutsiro	6
Rubavu	6
Nyabihu	6
Ngororero	6
Rusizi	6
Nyamasheke	6
**Eastern Zone**	
Rwamagana	6
Nyagatare	6
Gatsibo	6
Kayonza	6
Kirehe	6
Ngoma	6
Bugesera	6

EICV4 VUP

Zones	Number EICV 4 VUP Staff
Kigali City Zone	10
Southern Zone	7
Western Zone	7
Nothern Zone	6
Eastern Zone	7

EICV4 Panel

Zones	Number of EICV 4 PANEL Staff
Kigali City Zone	3
Southern Zone	3
Western Zone	3
Nothern Zone	3
Eastern Zone	3
