# Supplementary material for: Genetic diversity and relationship of Bugesera and Rwamagana indigenous chicken populations with SASSO chickens using DArTseq SNPs
Source: PLoS One. 2025 Sep 12;20(9):e0331316. doi: 10.1371/journal.pone.0331316 (PMC12431124; doi:10.1371/journal.pone.0331316)
Supplement: S4 File — (PDF) [file pone.0331316.s008.pdf]

The Republic of Rwanda

AGRICULTURAL HOUSEHOLD SURVEY 2020

REPORT

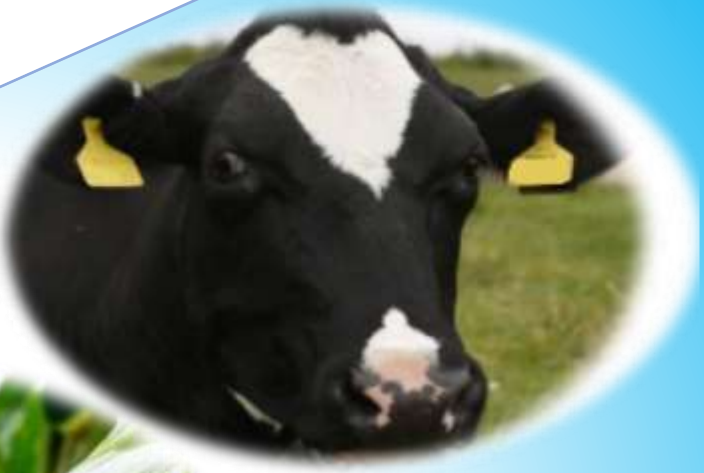

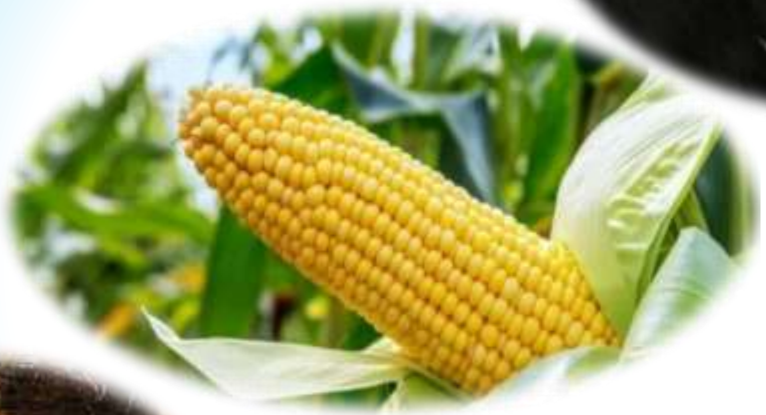

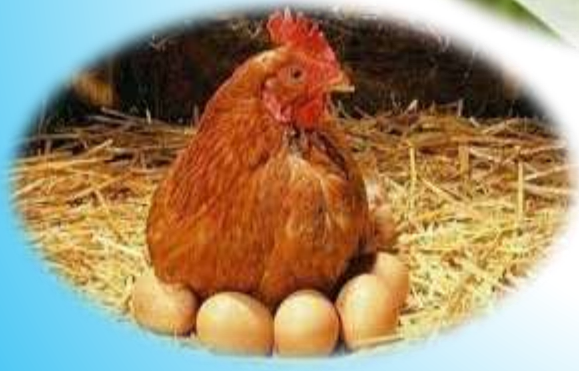

December 2021

The Republic of Rwanda

NATIONAL INSTITUTE OF STATISTICS OF RWANDA

AGRICULTURAL HOUSEHOLD SURVEY 2020

REPORT

December, 2021

December 2021

© National Institute of Statistics of Rwanda, Agricultural Household Survey 2020

Rwanda AHS 2020 report is available online at: www.statistics.gov.rw
Additional information not available in the Agricultural Household Survey 2020 report may be obtained from the NISR office in the Economic statistics unit.

Po. Box 6139 Kigali Rwanda ;
Website: www.statistics.gov.rw
Email : info@statistics.gov.rw

Recommended citation:

National Institute of Statistics of Rwanda (NISR), Agricultural Household Survey 2020 report, December 2021

FOREWORD

For the second time, the National Institute of Statistics of Rwanda (NISR) in cooperation with the Ministry of Agriculture and Animal Resources (MINAGRI) conducted Agricultural Household Survey (AHS) from September to October 2020. This survey was designed to collect statistical data on the agriculture sector which is not fully covered in Seasonal Agricultural Survey.

This report is an important source of information on the current agriculture situation in Rwandan households. It will be used in the monitoring of Rwanda agriculture policies and programs and will help to assist in addressing key agricultural issues.

Special recognition is given to the NISR department of economic statistics and agriculture and environmental statistics team, for their effort in conducting this survey. NISR would like to thank the international sampling consultant David J. Megill and the consultant in sample frame design Francisco Javier Gallego for their technical assistance that enabled them to conduct this survey. Special acknowledgements go to field enumerators and respondents for their contributions. NISR also expresses its gratitude to other partners who contributed with great effort in one way or another, for the great job done.

NISR invites policy makers, partners, the public, researchers, and all data users to value and use optimally the findings of this survey to contribute to Rwandan Agriculture development.

MURANGWA Yusuf
Director General, NISR

EXECUTIVE SUMMARY

This report presents the results of the Agricultural Household Survey carried out from 6th September to 8th October 2020. The survey covered 900 enumeration areas (villages), distributed across 30 districts of Rwanda, in which 10,666 sample households were selected. The information enclosed in this report covers agricultural activities done in the 2019/2020 agricultural year.

This survey gathered information at the household level about basic agriculture indicators related to demographic household characteristics, farm characteristics, livelihood activities, crop information, livestock production, level of inputs use, agricultural practices, extension services, level of implementation of agricultural programs, the financial aspect of agricultural households, and other agriculture-related indicators. The information on crop production and productivity was not captured here as it is well recorded in the Seasonal Agricultural Survey (SAS) also conducted by NISR.

This report provides results of AHS 2020 discussing different patterns across provinces and districts of Rwanda. Results have been also disaggregated by gender (male and female or male and female-headed households) to explore gender-related aspects in key social and economic characteristics of agricultural households in Rwanda.

Agricultural households' figures

An agricultural household is defined as a household with at least one member practicing agricultural activities (either crop or livestock production) that are taken as one of the sources of family income. In other words, it is a household that derives part of the income from agriculture, even when this is the smallest portion of the family earnings.

According to AHS2020 findings, the estimated number of agricultural households is 2.3 million, equivalent to 80.1 percent of total country households. In comparison to AHS 2017 findings, it is revealed that even though there was an increase in number of agricultural households from 2.1 million in 2017 to 2.3 million in 2020, the percentage of agricultural households remained almost the same (80.2 percent in 2017 versus 80.1 percent in 2020). Results show further that, 86.3 percent of agricultural households practice agriculture as the main livelihood activity, while the rest rely mostly on non-agricultural activities but performed crop/livestock production as another income-generating activity. Looking at different agricultural activities, in the agricultural year 2019/2020, 78.1 percent of households were engaged in crop production and 61.3 percent of households were engaged in livestock.

Demographic characteristics of agricultural households

Out of total agricultural households, 71.8 percent are headed by males. 65 percent of females heading agricultural households are widows. 71.6 percent of agricultural household heads are married, 19.7 percent are widowed, 5.3 percent are divorced while the rest are single.

In the 2019/2020 agricultural year, the average household size is 4.5 persons. The total population living in agricultural households was estimated to be 10.5 million, of whom 47.5 percent of household members were males and 52.5 percent were females.

Farmer's profile

A farmer is defined as a person who is engaged in agriculture by growing crops or raising livestock on his/her own or rented land to sustain himself or his/her family or for commercial purposes.

Results indicated that 3.8 million adult populations were engaged in agricultural activities (growing crops/and or livestock) including 2.1 million females representing a share of 56.6% of total farmers. In comparison to AHS 2017, the number of farmers decreased by 1.1 percent.

The majority of farmers[1] are old, less educated, and live in rural areas. The involvement of youth in agriculture is low, only a quarter of farmers (26.6percent) are between 16 and 30 years old.

Access and use of land

Results show that 87.6 percent of agricultural households, own cultivation land. Even though the big share of agricultural households has their own land, 49.5 percent rented agricultural land. Out of those who rented agricultural land, 37.1 percent rented agricultural land for the purpose of complementing their own land.

In regards to land use, 97.8 percent of agricultural households used the land for crop production, while 11.2 percent used the land for pasture (fodder crop cultivation or grazing). Besides, 18.8 percent of agricultural households have land used for forest plantation.

Farm structure

A household farm, also called land holding, is a collection of all parcels operated by a household, both owned and rented land.

[1] A farmer is referred to any adult person aged 16 or above involved in his/her own or joint agricultural activity such as crop production or livestock rearing during 2019/2020 the agricultural year.

Results show that 77.2 percent of agricultural households operate on farm with less than 0.5 hectares of size, 13.6 percent on farm ranging from 0.5 to 1 hectare(excl.) of size, 8.7 percent on farm ranging from 1 to 5 hectares (excl.) of size, while 0.4 percent operates on farm with size equivalent to five hectares and above.

Crops grown

In 2019/2020 agricultural year, 90.3 percent of agricultural households engaged in crop production grew legumes and pulses, cereals were grown by 76 percent, tubers and roots by 72.1 percent, banana by 40.2 percent and vegetables by 14 percent of agricultural households engaged in crop production.

Use of agricultural inputs

AHS 2020 findings show that 44.6 percent of agricultural households used improved seeds, 83.7 percent used organic fertilizers, 39.1 percent used inorganic fertilizers while 26.8 percent used pesticides. Most of the agricultural households used inorganic fertilizers sourced from Agro-dealers.

Agricultural practices

Findings show that 83.8 percent of agricultural households protected their land against erosion and 46.2 percent planted agroforestry trees in their plots. Only 14.6 percent of agricultural households practiced irrigation. Mechanical equipment was used by 0.1 percent of agricultural households.

Agriculture extension services

Empowering farmers with modern knowledge and farming practices leads to an increase in farm productivity, income, and welfare of their families as well. Results show that in the 2019/2020 agricultural year 65.0 percent of agricultural households received extension services.

In regard to services received, 55.8 percent of them received good agriculture practices information, followed by 27.1 percent who received knowledge on erosion control measures 15.5 percent gained skills on horticulture skills and 13.4 percent gained skills on pest management. In addition, 13.8 percent of extension receivers gained knowledge of using Smart Nkunganire System (SNS) while weather and climate information was received by 11.9 percent.

In regard to community membership, 12.6 percent of households belong to agricultural cooperatives, 20.7 percent of agricultural households have at least one member belonging to Twigire Muhinzi groups, whereas only 11.6 percent of agricultural households have at least one member belong to Farmer Field School (FFS).

Agricultural and social protection programs

AHS 2020 findings reveal that 24.5 percent of households performed their crop production activities in land consolidated sites, 35.6 percent of households have a kitchen garden while 1.0 percent of agricultural households acquired agriculture insurance. Besides, 4.1 percent of agricultural households received a cow from Girinka program and 4.3 percent of agricultural households received small livestock as part of the social protection scheme in 2020 year.

Saving and credits

Access to savings, credit and funds for agricultural households has a major contribution to the development especially in terms of getting agricultural inputs that increase production. Countrywide 58.1 percent of all agricultural households have a bank account. 68.5 percent of agricultural households belong to informal saving groups (tontine). 68.1 percent of all agricultural households reportedly managed to save money in either formal or informal financial institutions.

Additionally, 38.7 percent of all agricultural households had applied for a loan in 2020. Out of total agricultural households who applied for a loan, 70.1 percent of applications were destined to tontines. Received funds account for 2.8 percent of all agricultural households including money (received by 15.9 percent of agricultural households who received support), agriculture materials/ tools (received by 37.2 percent), and post-harvest tools (received by 0.7 percent) among others.

Livestock

Countrywide, 1.9 million agricultural households reportedly reared livestock. In regards to livestock distribution, 53.4 percent of households reared cattle, 37.6 percent reared goats, 33.7 percent reared pigs, 31.3 percent reared chickens, 9.9 percent reared sheep and 8.6 percent reared rabbits.

In regard to the estimates on livestock numbers including the livestock reared by households and large-scale farmers (individuals, cooperatives/associations, companies, and institutions), results show that, the overall livestock heads estimations are as follows: 1.6 million cattle, 1.7 million goats, 0.4 million sheep, 1.2 million pigs, 3.9 million chickens ,and 0.7 million rabbits.

Table 1: Summary of AHS 2020 results

No	Agricultural Households Survey (AHS) Indicator	2017	2020
1	Percentage of agricultural households over the total number of households in Rwanda	80.2	80.1
2	Estimated number of agricultural households in millions	2.1	2.3
3	Percentage of agricultural households headed by females	27.8	28.2
4	Percentage of agricultural households headed by males	72.2	71.8
5	Percentage of agricultural households engaged in crop production	78.6	78.1
6	Percentage of households engaged in livestock	62.6	61.3
7	Percentage of agricultural households whose main activity is both crop production and livestock	76.4	74.0
8	Estimated number of farmers in millions	3.8	3.8
9	Percentage of male farmers	40.2	43.43
10	Percentage of female farmers	59.8	56.57
11	Average agricultural household size	4.5	4.5
12	Average farm size in hectares		0.4
13	Percentage of agricultural households below 0.5ha		77.2
14	Percentage of agricultural households who used their own land for cultivation		87.6
15	Percentage of agricultural households who used rented land for cultivation		49.5
	**Crops**		
16	Percentage of agricultural households producing maize	56.8	67.7
17	Percentage of agricultural households producing paddy rice	3.1	3.2
18	Percentage of agricultural households producing sorghum	25.9	20.9
19	Percentage of agricultural households producing wheat	3.2	3.6
20	Percentage of agricultural households producing beans	92.8	88.6
21	Percentage of agricultural households producing peas	4.2	5.8
22	Percentage of agricultural households producing soybean	11.6	12.3
23	Percentage of agricultural households producing groundnuts	9.3	6.3
24	Percentage of agricultural households producing sweet potato	35.5	44.7
25	Percentage of agricultural households producing Irish potato	13.7	15.5
26	Percentage of agricultural households producing cassava	26.9	45.7
	**Use of inputs**		
28	Percentage of agricultural households who use improved seeds	43.8	44.6
29	Percentage of agricultural households who used organic fertilizer	81	83.7
30	Percentage of agricultural households who used inorganic fertilizer	36.6	39.1
31	Percentage of agricultural households who used pesticides	25.3	26.8
	**Agricultural practices**		
33	Percentage of agricultural households who practice irrigation	10.1	14.6
34	Percentage of agricultural households who practice erosion control measures	65.7	83.8
35	Percentage of agricultural households who planted agroforestry trees in their plots		46.2
36	Percentage of households who used mechanical equipment used in cultivation		0.1
37	Percentage of agricultural household who stored crop produces after harvest		62.8
38	Percentage of agricultural households with at least one member belongs to agricultural cooperative or association	12.5	12.5
39	Percentage of agricultural households with at least one member received an agricultural extension		65.0
40	Percentage of agricultural households with at least one member belongs to Twigire muhinzi/mworozi group	13	**20.7**
41	Percentage of agricultural households with at least one member belongs to Farmer Field School		**11.6**
	**Agriculture policies/programs**		
42	Percentage of agricultural households who have got contract farming	4.2	3.3
43	Percentage of agricultural households with agriculture insurance		1
44	Percentage of agricultural households who have received a cow from GIRINKA Program	13	4.1
45	Percentage of agricultural households who have received small livestock		4.3
46	Percentage of agricultural households who had a kitchen garden	44.4	36.3
47	Percentage of agricultural households operating in land consolidated site		24.5

No	Agricultural Households Survey (AHS) Indicator	2017	2020
	**Savings and credits**		
48	Percentage of agricultural households with at least one member had a bank account	49.6	58.1
49	Percentage of farmers with bank account		37.9
50	Percentage of agricultural households with at least one member belongs to tontine/solidarity fund		68.5
51	Percentage of agricultural HHs with at least one member who made savings		68.1
52	Percentage of farmers who made savings		48.8
53	Percentage of agricultural households with at least one member requested loan		38.7
	**Livestock reared**		
54	Percentage of cattle owners out of total households rearing livestock	61.0	53.4
55	Percentage of goat owners out of total households rearing livestock	53.6	37.6
56	Percentage of sheep owners out of total households rearing livestock	18.1	9.9
57	Percentage of pig owners out of total households rearing livestock	30.6	33.7
58	Percentage of chicken owners out of total households rearing livestock	33.7	31.3
59	Percentage of rabbit owners out of total households rearing livestock	15	8.6
60	Percentage of agricultural households who did beekeeping		2.6

TABLE OF CONTENT

FOREWORD ii
EXECUTIVE SUMMARY iii
TABLE OF CONTENT ix
LIST OF TABLES x
LIST OF FIGURES xiii
ACRONYMS AND ABBREVIATIONS xiv
CHAPTER 1: INTRODUCTION 1
1.1. Background 1
1.2. Sampling process 1
1.3. Weights calculation 3
1.4. Data collection 4
1.5. Survey instruments 5
CHAPTER 2: DEMOGRAPHICS AND LIVELIHOOD STRATEGIES 6
2.1. Agricultural households' numbers 6
2.2. Agricultural households' profile 7
2.3. Farmer's profile 10
2.4. Main livelihood activities of agricultural households 12
CHAPTER 3: AGRICULTURAL LAND ACQUISITION 14
3.1. Access to agricultural land 14
3.2. Farm size 15
3.3. Right to land 15
CHAPTER 4: CROPS AND FARMING PRACTICES 16
4.1. Crop produced in 2019/2020 agricultural year 16
4.2. Use of agricultural inputs 18
4.3. Agricultural practices 19
4.4. Post-harvest 21
CHAPTER 5: AGRICULTURE EXTENSION SERVICES 24
5.1 Communication asset ownership 24
5.2. Farmers' community groups membership 27
CHAPTER 6: AGRICULTURE POLICIES AND PROGRAMS 29
6.1 Land consolidation 29
6.2. Operating in Kitchen gardens 30
6.3. Agriculture insurance programs 30
6.4. Market linkage (contract farming and agribusiness) 31
6.5. Social protection programs 31
CHAPTER 7 : FINANCIAL SERVICES AND AGRICULTURAL SUPPORT 34
7.1. Savings 35
7.2. Access to loan 36
7.3. Agricultural funds/support 38
CHAPTER 8: LIVESTOCK 39
8.1 Livestock numbers 39
8.2. Livestock products 41
8.3 Animal Heath 44

LIST OF TABLES

Table 1: Summary of AHS 2020 results vii
Table 2: Sampled enumeration areas(EAs) per district 2
Table 3: Percentage of households per agricultural activities, by province 6
Table 4: Percentage of agricultural households, by province and agricultural activity types (gender-disaggregated) 7
Table 5: Percentage of agricultural-household heads by marital status and province (gender-disaggregated) 7
Table 6: Agricultural households population by age group and province in 2020 7
Table 7: Percentage of agricultural households' population aged 16 and above by sex, education level and province 8
Table 8: Demographic characteristics of Agricultural household members 9
Table 9: Number of farmers by province and agricultural activity type (gender-disaggregated) . 10
Table 10: Farmers demographic characteristics (in percentage) 11
Table 11: Distribution of farmers by sex, age group and province (in percentage) 11
Table 12: Distribution of farmers by sex, education level and province (in percentage) 12
Table 13: Percentage of agricultural households practicing other livelihood activities that complement agriculture, by province 13
Table 14: Percentage of agricultural households who accessed agricultural land by land ownership and province 14
Table 15: Percentage of agricultural households by land use type and province 14
Table 16: Percentage of agricultural households accessing land by farm size categories and province 15
Table 17: Percentage of farmers with right to land 15
Table 18: Percentage of households producing staple crops by crop type and province 16
Table 19: Percentage of households producing major vegetable crops by vegetable type and province 17
Table 20: Percentage of households producing fruits by province 17
Table 21: Percentage of households per types of fruits produced by province 17
Table 22: Percentage of agricultural households per different agricultural inputs used by province 18
Table 23: Percentage of households who used improved seeds by province and important crop type 18
Table 24: Percentage of households who used inorganic fertilizers by province and source of fertilizer 19
Table 25: Percentage of agricultural households per different agricultural practices 19
Table 26: Percentage of agricultural households by types of erosion control measures 20
Table 27: Percentage of agricultural households who irrigated land by irrigation techniques and province 20
Table 28: Source of water used for irrigation (percentage) by province 20
Table 29: Percentage of agricultural households who stored crop produce by storage length and province 22
Table 30: Percentage of agricultural households who stored crop produce by crop type and province 22
Table 31: Percentage of agricultural households who stored crop produce by type of storage facility and province 23
Table 32: Percentage of agricultural households owning communication assets 24
Table 33: Percentage of agricultural households who received extension services 25
Table 34: Percentage of agricultural households by type and source of extension services 26
Table 35: Percentage of agricultural households by type of cooperatives 27
Table 36: Percentage of agricultural households who belong the community groups by province 28

Table 37: Percentage of HHs who benefited from Girinka program by province and type of providers 31
Table 38: Percentage of households who benefited from the small stock program by type of small livestock 32
Table 39: Percentage of agricultural households/farmers having bank account by province 34
Table 40: Percentage of agricultural households by type of financial institutions in which they have a bank account and by province 34
Table 41: Percentage of agricultural households/farmers who did savings by province 35
Table 42: Percentage of agricultural households by province and type of financial institutions (formal or informal) in which they made savings 36
Table 43: Percentage of agricultural households/farmers who requested loan by province 36
Table 44: Percentage of agricultural households by province and source of requested loan 36
Table 45: Percentage of agricultural households who received any support by support/fund type and province 38
Table 46: Percentage of agricultural households who received any support by the source of support/fund and province 38
Table 47: Percentage of households raising different types of livestock by province and sex of household head 39
Table 48: Number of livestock raised, by type and province 40
Table 49: Number of cattle that were reared by agricultural households on the day of the interview, by breeds, sex age and province 40
Table 50: Number of goats that were reared by agricultural households on the day of the interview, by sex age and province 41
Table 51: Number of sheep that were reared by agricultural households on the day of the interview, by sex age and province 41
Table 52: Number of pigs that were reared by agricultural households on the day of the interview, by sex age and province 41
Table 53: Monthly lactating cows per Province, 2019/20 Agricultural Year 41
Table 54: Average milk production in litters per cow per day by province 42
Table 55: Milk utilisation (in %) 42
Table 56: Average farm gate milk price, year 2019/20 (Frw/litre) 42
Table 57: Annual Egg production (number) per province, 2019/20 Agricultural Year 43
Table 58: Annual honey production [in Kg] and usage during 2019/2020agricultural year 43
Table 59: Animal Heath and reproduction 44
Table 60: Animal feeding practices by livestock type (%) 44
Table 61: Number of animals born, purchased, sold or consumed by households 45
Table 62: Socio-demographic characteristics of agricultural households by district 47
Table 63: Percentage of households who engaged in different agricultural activities during the 2019/2020 agricultural year by district 48
Table 64: Percentage of agricultural households by district and agricultural activity types during 2019/2020 agricultural year 49
Table 65: Number of farmers by District and agricultural activity type 50
Table 66: Socio-demographic characteristics of farmers by district (in percentage) 51
Table 67: Percentage of agricultural households who accessed land by and District and land use type 52
Table 68: Percentage of agriculture households accessing land by District, farm category 53
Table 69: Percentage of crop-producing households by district and crop type 54
Table 70: Percentage of households producing vegetable crops by District and crop type 55
Table 71: Percentage of crop-producing households who used different agricultural inputs by District 56
Table 72: Use of agricultural practices among agricultural households (%) 57
Table 73: Percentage of agricultural households who stored harvested crop produces by storage length and District 58

Table 74: Percentage of agricultural households who own communication assets by District....59
Table 75: Percentage of agricultural households who received extension services by extension type, and district....60
Table 76: Percentage of agricultural households with at least one member who joined community groups by District....61
Table 77: Percentage of agricultural HHs with at least one member having a bank account......62
Table 78: Percentage of agricultural households/farmers who did savings by District................63

LIST OF FIGURES

Figure 1: Distribution of agricultural households' members by size 8
Figure 2: Percentage of agricultural household population by age-group (from 16 years and above) per education attainment level 9
Figure 3: Percentage of agricultural households' population by level of education 10
Figure 4: Percentage of farmers by age-group (from 16 years and above) per education attainment level 12
Figure 5: Percentage of agricultural households practicing agriculture as main livelihood activity, by province 13
Figure 6: Reasons for not using mechanical equipment 21
Figure 7: Percentage of agricultural households who stored crop produce by storage length 21
Figure 8: Motivations of joining the agricultural cooperative 28
Figure 9: Percentage of agricultural households operating in land consolidated site by province 29
Figure 10: Percentage of cultivated land under land consolidation 29
Figure 11: Percentage of households with kitchen garden by province 30
Figure 12: Percentage of agricultural households with agriculture insurance 30
Figure 13: Percentage of agricultural households with contract farming 31
Figure 14:Reasons for abandonment of rearing cow from Girinka program 32
Figure 15: Percentage of HHs by source of small livestock received 33
Figure 16: Percentage of agricultural households who have at least one member belonging to tontine/solidarity fund(ikimina) by province 35
Figure 17: Main reasons for rejecting loans 37
Figure 18: Planned use of rejected loans 37
Figure 19: Percentage of agricultural households who received any support/fund for grant for agricultural purpose by province 38

ACRONYMS AND ABBREVIATIONS

%: Percentage
EAs: Enumeration Areas
AHS: Agriculture Household Survey
CAPI: Computer-Assisted Personal Interviewing
CSPro: Census and Survey Processing System
FAO: Food and Agriculture Organization
GIS: Geographical Information System
HH: Household
HHH: Household Head
Kg: Kilograms
LSF: Large Scale Farmers
MINAGRI: Ministry of Agriculture and Animal Resources
NAEB: National Agriculture Export Board
NGO: Non-Government Organization
NISR: National Institute of Statistics of Rwanda
PPS: Probability Proportional to Size
RAB: Rwanda Agriculture Board
RGPHC-2012: 2012 Rwanda General Population and Housing Census
SACCO: Savings and Credit Cooperative Organization.
SAS: Seasonal Agriculture Survey
VUP: Vision Umurenge Program

CHAPTER 1: INTRODUCTION

1.1. Background

The National Institute of Statistics of Rwanda (NISR) in cooperation with the Ministry of Agriculture and Animal Resources (MINAGRI) conducted Agricultural Household Survey (AHS) for the second time. The data collection took place from September to October 2020. This survey was designed to collect statistical data on the agriculture sector which is not fully covered in Seasonal Agricultural Survey. NISR has committed to conducting this survey every three years.

This survey gathered information at the household level about basic agriculture indicators related to demographic household characteristics, farm characteristics, livelihood activities, crop information, livestock production, level of inputs use, agricultural practices, extension services, level of implementation of agricultural programs, the financial aspect of agricultural households, and other agriculture-related indicators.

1.2. Sampling process

To ensure a good representation of data at the district level, the survey was conducted using a sample size of 900 Enumeration areas (EAs). A sample frame used was composed of a list of enumeration areas (EAs) retrieved from the 2012 Rwanda General Population and Housing Census (RGPHC-2012). A stratified two-stages sample design was used.

The first stage focused on a stratified sample of enumeration areas from the latest RGPHC-2012. Given that rural areas are dominated by many households practicing agriculture, the frame of EAs was sorted by urban and rural areas within districts. This provides an implicit stratification of the households by urban and rural areas. Out of 900 EAs, 860 were selected from rural EAs while the rest was selected from urban EAs, where there are fewer but not negligible numbers of households practicing agriculture.

To ensure adequate geographical distribution of the sample and given that the results were analysed up to district level, the sample of 860 rural EAs was allocated equally among 30 districts, while a sample of 40 urban EAs was allocated proportionally throughout the country. At this first stage, the sampled EAs in each district were selected systematically with a probability proportional to size (PPS) measured in terms of the total number of households in each EA from the RGPHC-2012.

The second stage looked at a random selection of a fixed number of 12 households who only did agriculture in each sampled EA. 12 households were selected from an updated number of households listed in each sampled EA, in the first phase of AHS 2020 data collection.

It is important to note that there are EAs with less than 12 agricultural households and these have been directly taken as a sample at the second stage. Finally, the sampling process conveyed a total sample of 10,666 agricultural households.

Table 2: Sampled enumeration areas (EAs) per district

District	Number of sampled EAs	Percentage of sample share at national level	Listed households	Sampled agricultural households
Kigali	120	13.3	31,300	1,340
Nyarugenge	37	4.1	9,222	412
Gasabo	44	4.9	11,692	491
Kicukiro	39	4.3	10,386	437
South	225	25	44,831	2,693
Nyanza	28	3.1	6,296	334
Gisagara	28	3.1	4,919	335
Nyaruguru	28	3.1	5,348	335
Huye	28	3.1	4,765	335
Nyamagabe	28	3.1	4,969	336
Ruhango	28	3.1	4,662	335
Muhanga	28	3.1	6,711	335
Kamonyi	29	3.2	7,161	348
West	205	22.8	36,097	2,447
Karongi	28	3.1	4,256	336
Rutsiro	28	3.1	4,766	336
Rubavu	30	3.3	6,229	350
Nyabihu	28	3.1	4,440	335
Ngororero	30	3.3	5,706	360
Rusizi	31	3.4	5,550	370
Nyamasheke	30	3.3	5,150	360
North	146	16.2	25,960	1,750
Rulindo	28	3.1	5,195	335
Gakenke	30	3.3	4,341	360
Musanze	30	3.3	6,907	360
Burera	28	3.1	4,450	336
Gicumbi	30	3.3	5,067	359
East	204	22.7	42,174	2,436
Rwamagana	28	3.1	6,437	334
Nyagatare	30	3.3	6,733	358
Gatsibo	30	3.3	6,812	359
Kayonza	28	3.1	6,104	335
Kirehe	28	3.1	3,934	332
Ngoma	30	3.3	5,601	359
Bugesera	30	3.3	6,553	359
Rwanda	900	100	180,362	10,666

Source: NISR, AHS 2020

Moreover, a list large scale farmers (LSF) for livestock was added to the sampled agricultural households to have a complete coverage of livestock numbers in the country.

As consensus, a large-scale farmer was defined as any individual person, institution, company, association or cooperative rearing at least 20 cattle, 40 pigs, 100 goats/sheep or 100 pigs, or 500 chicken was considered. For beekeeping, an LSF selected was the one with 10 bee hives. A total of two thousand and three hundred forty-five (2,345) large scale farmers were identified and visited in all districts.

These includes 1,143 large scale farmers for cattle, 22 for goats, 3 for sheep, 165 for pigs, 288 for chicken, 7 for rabbits, 567 for beekeeping, and 150 rearing various types of livestock.

1.3. Weights calculation

The weight of a given sample-households is the inverse of the probability of selection of households. This probability is computed as the product of two probabilities.

The first probability is the probability of selection of the enumeration area (EA) h, where the sample households reside, and it was calculated as follows:

$$\boldsymbol{p_{1hi}} = \frac{\boldsymbol{n_h \times M_{hi}}}{\boldsymbol{M_h}}$$

where:
p_{1hi} = First sampling probability of the i-th sample EA for AHS in district h
n_h = Number of sampled EAs selected in district h
M_{hi} = Total number of households in the i-th sampled EA in district h from the 2012 Rwanda Population and Housing Census frame
M_h = Total number of households in district h in the 2012 Rwanda Population and Housing Census frame

The second probability is the probability of selection of the agricultural household within the i-th sampled enumeration area in the district h, and was calculated as follow:

$$\boldsymbol{p_{2hi}} = \frac{\boldsymbol{m_{hi}}}{\boldsymbol{M'_{hi}}}$$

Where:
p_{2hi}= Second sampling probability of selection of an agricultural household in the i-th sampled EA in a district h
m_{hi} = Number of sample agricultural households selected in the i-th sampled EA in district h
M'_{hi} =Total number of agricultural households listed in the i-th sampled EA in district h

At the second stage, 12 sample agricultural households were selected systematically with equal probability from the listing for each sample EA.

The overall probability of selecting an agricultural household in an i-th sampled EAs is the product of the two probabilities and is expressed as follows:

$$\boldsymbol{p_{hi}} = \frac{\boldsymbol{n_h \times M_{hi}}}{\boldsymbol{M_h}} \times \frac{\boldsymbol{m_{hi}}}{\boldsymbol{M'_{hi}}}$$

The overall household weight for the AHS sample household is the inverse of the overall probability

$$\boldsymbol{W_{hi}} = \frac{\mathbf{1}}{\boldsymbol{p_{hi}}} = \frac{\boldsymbol{M_h} \times \boldsymbol{M'_{hi}}}{\boldsymbol{n_h} \times \boldsymbol{M_{hi}} \times \boldsymbol{m_{hi}}}$$

Following the AHS 2020 data collection, it was necessary to adjust the basic weights to account for non-interviews, as follows:

$$W'_{hi} = W_{hi} \times \frac{m_{hi}}{m'_{hi}}$$

where:

m'hi = number of sample agricultural households with completed AHS interviews in the i-th sample EA in district h

1.4. Data collection

Data collection for agricultural household survey 2020 was conducted into two separate phases:

- The first phase consisted of listing all households in sampled enumeration areas. The listing exercise aims to identify households engaged in cropping or/and livestock activities during the 2019/2020 agricultural year. Based on noticeable exception on the production of fruits, during listing special questions on fruits was introduced to all listed households since both agricultural and non-agricultural households can grow fruits. An agricultural household was defined as a household whose one of its sources of income is derived from agricultural production (crop production and/or livestock).
- The second phase was dedicated to interviewing the selected agricultural households. During this time, a well-structured household survey questionnaire was used to gather all information on agricultural activities done during the agricultural year 2019/2020 starting from September 2019 to August 2020. The questionnaire was administered to a whoever member of the household well informed on agricultural activities, in most cases the household head.
- Data collection started on 6th September 2020 and ended on 8th October 2020. In this survey Computer-Assisted-Personal Interviewing (CAPI) technique was used to improve the speed of delivery and quality of data. The survey employed 186 field workers including 152 enumerators, 31 team leaders and 3 editors, while 25 supervisors assisted in field data quality assurance.

1.5. Survey instruments

The questionnaire was designed in CSPro software and android tablets were used to facilitate electronic data collection. The survey questionnaire was designed with a common set of core modules on household composition, household members' characteristics, land use and ownership, crops planted during the agricultural year 2019/2020, agriculture extension services, agricultural programs, access to savings and credits, access to inputs, livestock numbers, livestock production (milk, eggs and honey) and other agricultural related information. Moreover, GPS was used for locating sampled households at the same time used as a monitoring tool for field staff.

CHAPTER 2: DEMOGRAPHICS AND LIVELIHOOD STRATEGIES

This section presents thoroughly the findings of AHS 2020 related to demographic household characteristics, farm characteristics, livelihood activities, major crop and vegetables grown, fruits production and use. The survey provide data on livestock numbers, changes in inventory, and livestock products. It also provides the status on the level of inputs use, agricultural practices, extension services, level of implementation of agricultural programs, the financial aspect of agricultural households, and other agriculture-related indicators.

2.1. Agricultural households' numbers

Table 3 shows that during the 2019/2020 agricultural year, 80.1 percent of households are agricultural (engage in either crop or livestock production), which is a slight decline since AHS2017, where agricultural households were 80.2 percent but households who did agriculture as their main livelihood activity were 69.2 percent in AHS 2020. Taking into consideration the type of agricultural activity, 78.1 percent of all households in Rwanda (a decrease of 0.5 percent points compared to AHS 2017) were engaged in crop production, whereas 61.3 percent of households (a decrease of 1.3 percent points compared to AHS 2017) were engaged in livestock. Though there is a decrease in the percentage of agricultural households, the corresponding number increased to 2.3 million agricultural households in 2020 from 2.1 million in 2017.

Table 3: Percentage of households per agricultural activities, by province

	AHS 2020					AHS 2017			
	Percentage of households engaged in				Total estimated number of households (,000)	Percentage of households engaged in			Total estimated number of households (,000)
			production of				Production of		
Province	Any agriculture activity	Agriculture as main livelihood activity	Crops	livestock		Any agriculture activity	Crops	livestock	
Kigali	23.9	15.0	21.4	14.7	361	28.6	26.3	16.1	328
South	89.3	81.1	87.6	71.4	707	88.5	87.3	72.0	652
West	86.8	69.2	84.1	68.0	624	86.1	83.0	68.7	533
North	91.2	80.1	90.2	73.9	476	90.0	89.1	72.8	474
East	86.1	77.2	84.0	60.8	729	85.6	84.3	64.2	711
Rwanda	80.1	69.2	78.1	61.3	2,898	80.2	78.6	62.6	2,698

Source: NISR, AHS 2020

A household may be engaged in crops only or livestock only or in both agricultural activities. Table 4 shows that out of total household who engaged in agriculture, 23.5 percent of them practiced solely crop production, 2.5 percent were exclusively in livestock, while 74.0 percent did crop production in a combination of livestock rearing. This shows that most of households who engage in agriculture mostly combine growing crops and rearing livestock, a good indicator of access to organic manure.

Table 4: Percentage of agricultural households, by province and agricultural activity types (gender-disaggregated)

	Percentage of agricultural households who did			Total agricultural households (,000s)
	crop production only	livestock production only	both crop and livestock production	
Rwanda	23.5	2.5	74.0	2,322
By Province				
Kigali	38.6	10.4	51.0	86
South	20.1	1.9	78.0	631
West	21.7	3.1	75.2	542
North	19.1	1.1	79.9	435
East	29.4	2.4	68.1	628
By HHH sex				
Male-headed HHs	18.1	2.4	79.6	1,667
Female-headed HHs	24.6	2.3	73.2	655

Source: NISR, AHS 2020

2.2. Agricultural households' profile

Table 5 shows that 71.8 percent of agricultural households are headed by males. Among agricultural households' heads, 71.6 percent are married, 19.7 percent are widowed, 5.3 percent are divorced while the rest are single.

Table 5: Percentage of agricultural-household heads by marital status and province (gender-disaggregated)

	Marital status				
	Single	Married	Widowed	Divorced	Total
Rwanda	3.3	71.6	19.7	5.3	100.0
By Province					
Kigali	5.9	73.9	15.3	5.0	100.0
South	3.0	69.3	21.3	6.3	100.0
West	3.9	72.3	19.6	4.2	100.0
North	3.4	73.6	19.1	4.0	100.0
East	2.8	71.7	19.2	6.3	100.0
By HHH sex					
Male heads	2.2	94.4	1.9	1.5	100.0
Female heads	6.2	13.7	65.0	15.1	100.0

Source: NISR, AHS 2020

Table 6 shows that the total population living in agricultural households was estimated at 10.5 million, of whom 52.5 percent were females. Rwandan agricultural household population is largely characterized by a young population whereby 68.0 percent are in the age of 30 years and below.

Table 6: Agricultural households population by age group and province in 2020

	Province (,000)					Rwanda (,000)	
Age group	Kigali	South	West	North	East	Number	Percent
People below 16 years	175	1,140	1,117	760	1,198	4,389	41.9
People from 16 to 30 years	102	703	671	511	750	2,738	26.1
People from 31 to 64 years	118	792	679	520	772	2,880	27.5
People from 65 years & above	10	140	113	100	116	479	4.6
Rwanda	405	2,775	2,579	1,891	2,836	10,486	100.0

Source: NISR, AHS 2020

Table 8 shows that the average number of household members is 4.5, while figure 1 shows the distribution of agricultural household membership where 54.2 percent of agricultural households are composed of 3 to 5 members.

Figure 1: Distribution of agricultural households' members by size

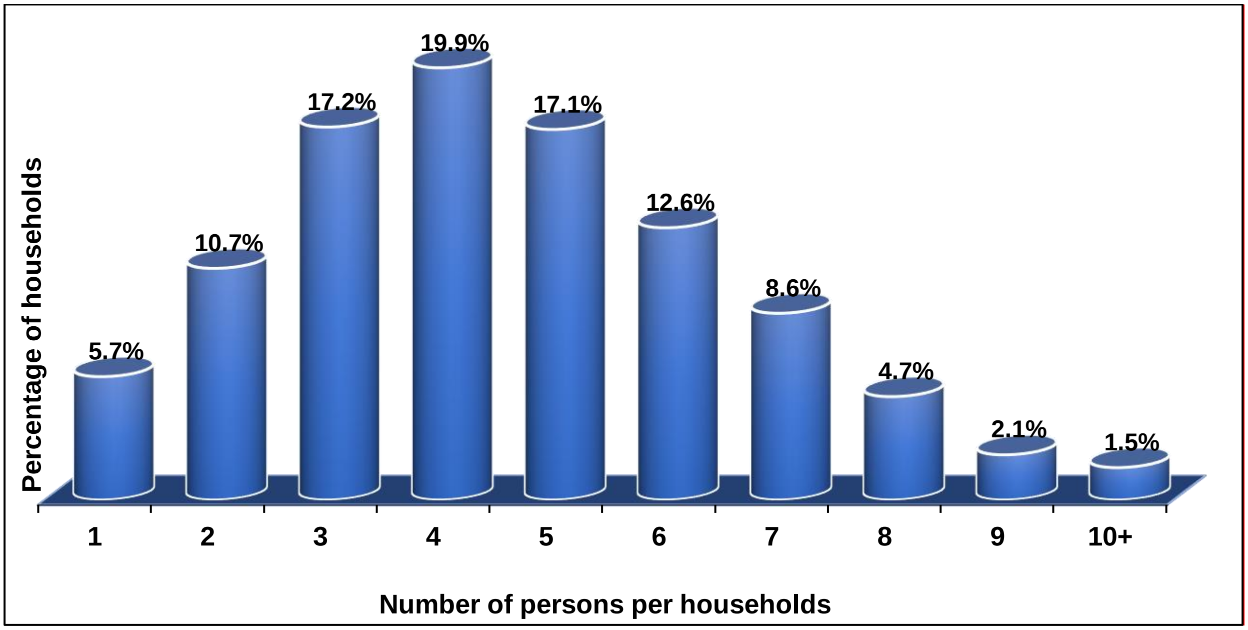

Source: NISR, AHS 2020

Table 7: Percentage of agricultural households' population aged 16 and above by sex, education level and province

Province		Kigali	South	West	North	East	Rwanda
Male	Primary	52.7	63.8	61.4	63.8	60.5	61.9
	Secondary	31.9	21.2	24.7	20.9	23.7	23.1
	University	8.6	2.7	2.6	3.4	2.9	3.1
	No education	6.8	12.4	11.4	12	12.9	12
Female	Primary	52.7	58.1	55.5	57.9	55.8	56.6
	Secondary	29.8	22.6	23.3	20.4	23.2	22.8
	University	6.7	1.9	1.6	1.2	1.4	1.7
	No education	10.8	17.4	19.6	20.5	19.7	18.9
Both males and females	Primary	52.7	60.7	58.2	60.5	58	59
	Secondary	30.8	21.9	24	20.6	23.4	22.9
	University	7.6	2.3	2	2.2	2.1	2.4
	No education	8.9	15.1	15.9	16.7	16.5	15.7
Number of agricultural households' population aged 16 years and above (,000)		230	1,635	1,463	1,131	1,639	6,097

Source: NISR, AHS 2020

Table 7 shows that 59.0 percent of the agricultural population at working age (16 years and above) attained primary education, followed 22.9 percent who attained secondary education, no education by 15.7, while university was attained by 2.4 percent

Figure 3 presents the education attainment of the agricultural household population by age group. Overall, a higher percentage of agriculture household population at working age (53.0 percent) with primary school level fall into the age group of 31 to 64 years old.

For the secondary school level, the large proportion of agriculture household population at working age (78.1 percent) belong to the age group of 16-30 years old. While for university attainment a large number is found in the age group of 31 to 64 years (50.1 percent). People with no education appeared most in the age group of 65 years old and above (63.3 percent).

Figure 2: Percentage of agricultural household population by age-group (from 16 years and above) per education attainment level

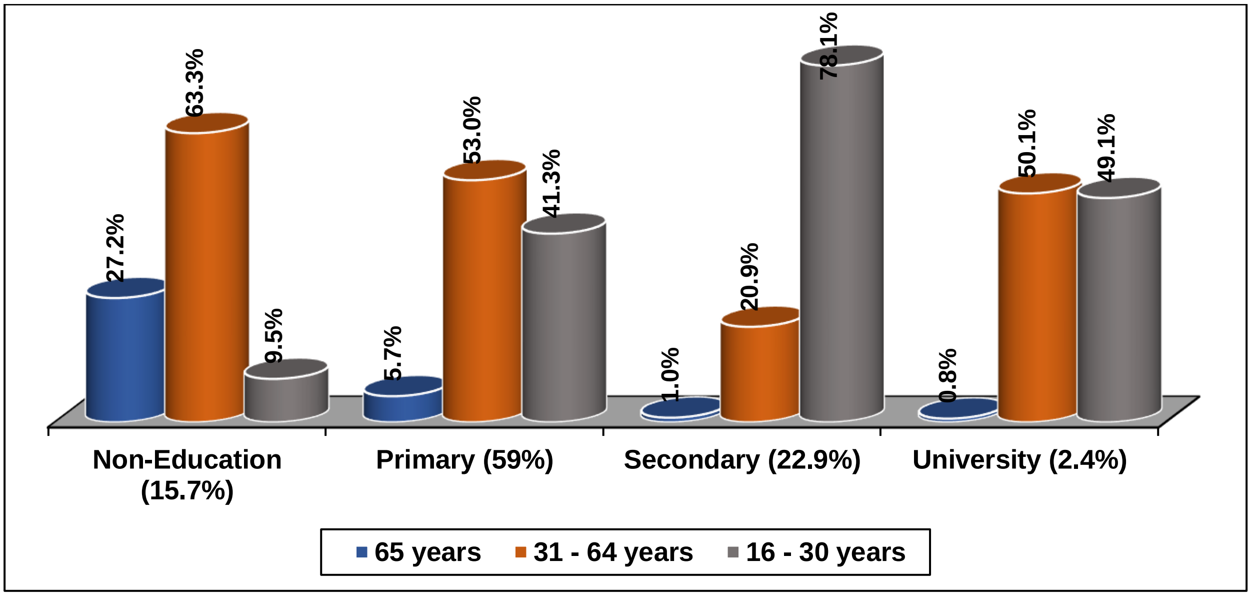

Source: NISR, AHS 2020

Table 8 shows that 69.1 percent of the agricultural population attained primary education, followed 18.3 percent who attained secondary education, no education by 11.0, while university was attained by 1.6 percent as displayed on figure 3

Table 8: Demographic characteristics of Agricultural household members

	By province					
Characteristic	Kigali	South	West	North	East	Rwanda
Average agricultural household size	4.7	4.4	4.8	4.3	4.5	4.5
Household heads by sex (%)						
Male-headed households	75.9	69.4	71.3	73.5	73	71.8
Female-headed households	24.2	30.6	28.7	26.5	27.1	28.2
Agricultural household members by sex (%)						
Male	48.2	48.0	47.3	46.7	47.8	47.5
Female	51.8	52.0	52.7	53.3	52.2	52.5
Agricultural household members by age group (%)						
Below 16 years	43.2	41.1	43.3	40.2	42.2	41.9
16 to 30 years	25.3	25.3	26.0	27.0	26.5	26.1
31 to 64 years	29.1	28.5	26.3	27.5	27.2	27.5
65 years and above	2.4	5.1	4.4	5.3	4.1	4.6
Agricultural households' members' Education attained (%)						
No education	6.2	10.5	10.9	11.8	11.7	11.0
Primary	64.1	70.2	69.1	69.3	68.5	69.1
Secondary	24.6	17.8	18.7	17.3	18.4	18.3
University	5.1	1.6	1.4	1.5	1.4	1.6

Source: NISR, AHS 2020

Figure 3: Percentage of agricultural households' population by level of education

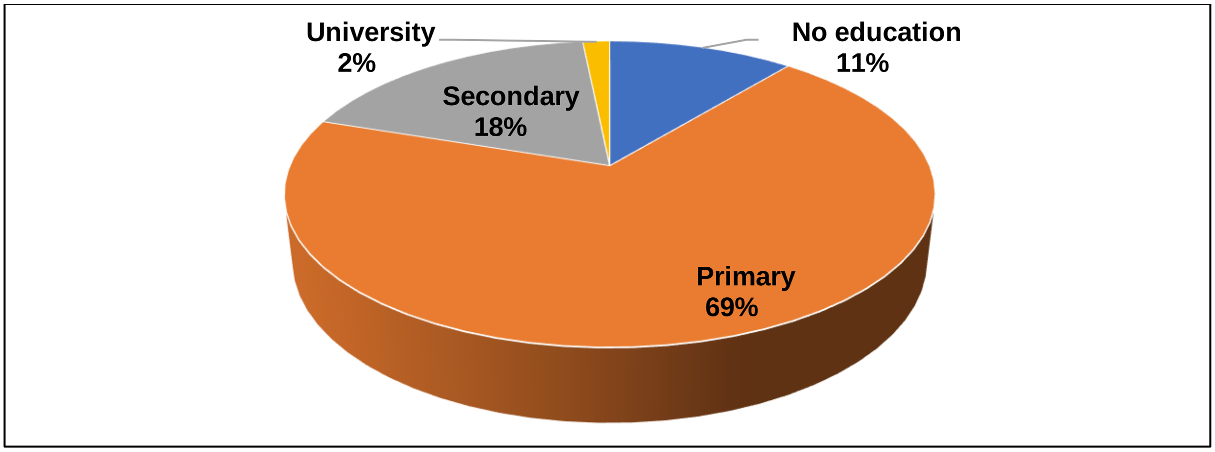

Source: NISR, AHS 2020

2.3. Farmer's profile

Results of AHS 2020 show that, there are 3.8 million regarded as farmers, of whom 2.8 million practice agriculture as the main activity. The proportion of farmers reduced by 1.1 percent when compared to AHS 2017.

Table 9: Number of farmers by province and agricultural activity type (gender-disaggregated)

	Agricultural activity type				Total farmers
	Farmers practicing agriculture as main activity		Farmers practicing agriculture as a second activity		
	Number (,000)	%	Number (,000)	%	Number (,000)
Rwanda	2,816	73.5	1,016	26.5	3,832
By Province					
Kigali	78	61.8	48	38.2	126
South	767	74.6	261	25.4	1,028
West	622	72.9	231	27.1	852
North	587	77.1	174	22.9	761
East	763	71.7	302	28.3	1,065
By sex					
Male	1,131	67.9	534	32.1	1,665
Female	1,686	77.8	482	22.2	2,168

Source: NISR, AHS 2020

Table 10 shows a higher percentage of female farmers, 56.6 percent, than males, 43.4 percent. The predominance of female farmers was also recorded in the 2017 agricultural household survey but it decreased by 3.2 percent when compared to AHS 2020 results.

Table 10: Farmers' demographic characteristics (in percentage)

Characteristic	By province Kigali	South	West	North	East	Rwanda
% of farmers out of total agricultural working population	54.9	62.9	58.3	67.3	65.0	62.9
Percentage of farmers by sex						
Male	45.6	43.0	43.9	42.0	44.3	43.4
Female	54.4	57.0	56.1	58.1	55.7	56.6
Percentage of farmers by age group						
16 to 30 years	24.9	22.9	25.4	29.8	29.0	26.6
31 to 64 years	69.9	66.9	64.8	59.8	62.9	64.0
65 years and above	5.2	10.1	9.7	10.4	8.1	9.4
Percentage of farmers by education						
No education	12.0	19.2	20.6	20.2	20.3	19.8
Primary	63.4	65.5	63.6	65.6	63.5	64.5
Secondary	20.3	13.8	14.3	12.8	14.7	14.2
University	4.3	1.6	1.5	1.5	1.5	1.6

Source: NISR, AHS 2020

Table 10 also shows that on overall 64.5 percent of farmers have attained primary school, followed by 19.8 percent who have no level of education, 14.2 percent with secondary school level of education and 1.6 percent with university level of education.

Table 11: Distribution of farmers by sex, age group and province (in percentage)

Province		Kigali	South	West	North	East	Rwanda
Males	16 to 30 years	23.9	24	27.2	29.5	28.5	27
	31 to 64 years	70.7	67.5	63.8	61.1	64.1	64.6
	65 years and above	5.4	8.5	9	9.5	7.4	8.4
	Total	100	100	100	100	100	100
Females	16 to 30 years	25.8	22.1	24.1	30.1	29.4	26.3
	31 to 64 years	69.2	66.5	65.6	58.8	62	63.6
	65 years and above	5.0	11.3	10.3	11.1	8.7	10.1
	Total	100	100	100	100	100	100
Both males and females	16 to 30 years	24.9	22.9	25.4	29.8	29	26.6
	31 to 64 years	69.9	66.9	64.8	59.8	62.9	64
	65 years and above	5.2	10.1	9.7	10.4	8.1	9.4
	Total	100	100	100	100	100	100
Total number of farmers (,000)		126	1,028	852	761	1,065	3,832

Source: NISR, AHS 2020

Figure 4 represents farmer's level of education by age group. 66.6 percent of farmers at working age who have attained primary schools fall into the age group of 31 to 64 years old. This ratio attains 79.5 percent for farmers who attained university level and 69.2 percent for farmers with no education. However, for secondary school level, 54.8 percent belong to the age group of 16 to 30 years. Details about farmer's education attainment are found in table 12.

Figure 2: Percentage of farmers by age-group (from 16 years and above) per education attainment level

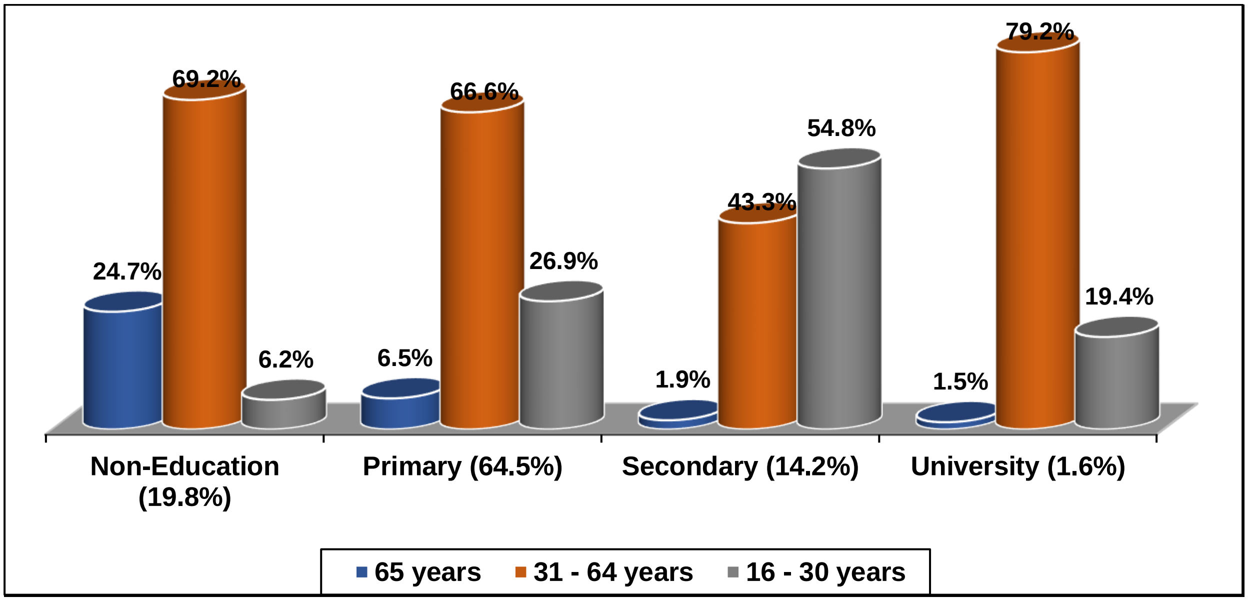

Source: NISR, AHS 2020

Table 12: Distribution of farmers by sex, education level and province (in percentage)

Sex	Level of education	Provinces					Rwanda
		Kigali	South	West	North	East	**Rwanda**
Males	Primary	61.7	67.9	68.5	70.1	65.7	**67.6**
	Secondary	22.7	14.1	15	13	15.5	**14.8**
	University	5.3	1.8	2	2.5	2.2	**2.2**
	No education	10.3	16.2	14.5	14.4	16.6	**15.4**
	Total	100	100	100	100	100	**100**
Females	Primary	64.8	63.6	59.8	62.3	61.7	**62**
	Secondary	18.3	13.5	13.7	12.6	14	**13.7**
	University	3.5	1.4	1.1	0.9	1	**1.2**
	No education	13.5	21.5	25.4	24.3	23.3	**23.2**
	Total	100	100	100	100	100	**100**
Both males and females	Primary	63.4	65.5	63.6	65.6	63.5	**64.5**
	Secondary	20.3	13.8	14.3	12.8	14.7	**14.2**
	University	4.3	1.6	1.5	1.5	1.5	**1.6**
	No education	12	19.2	20.6	20.2	20.3	**19.8**
	Total	100	100	100	100	100	**100**
Total number of farmers (,000)		126	1,028	852	761	1,065	**3,832**

Source: NISR, AHS 2020

2.4. Main livelihood activities of agricultural households

Figure 5 shows that 86.3 percent of agricultural households practice agriculture as their main livelihood activity, while the rest rely on non-agricultural activities though they are engaged in agriculture as the complementary income-generating activity. The Southern province has the highest ratio of agricultural households who practice agriculture as main activity with 90.9 percent of agricultural households.

Figure 3: Percentage of agricultural households practicing agriculture as main livelihood activity, by province

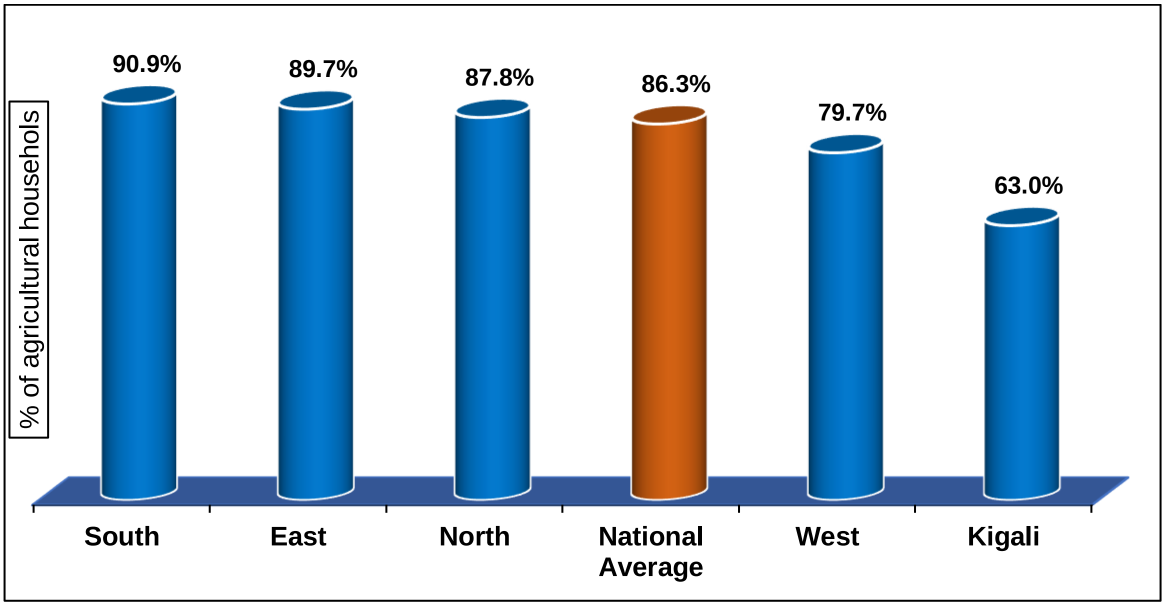

Source: NISR, AHS 2020

Table 13 shows that 64.4 percent of agricultural households practiced other income-generating activities that complement agriculture, including daily labour (36.7 percent), informal sale (5.9 percent), salaried work (4.5 percent), VUP public work (3.1 percent), trading of agricultural products (2.0 percent) among others. However, 35.6 percent did not practice any other livelihood activity.

Table 13: Percentage of agricultural households practicing other livelihood activities that complement agriculture, by province

	Province					
Livelihood activity	Kigali	South	West	North	East	Rwanda
Daily Labour	31.1	35.6	40.5	36.6	35.3	36.7
Fishing, hunting, gathering	0.2	0.9	0.7	0.1	0.8	0.7
Skilled labour	5.4	2.6	1.4	2.3	1.9	2.2
Purchase and Sale of agricultural products	1.1	1.5	2.3	1.7	2.7	2.0
Purchase and sale of livestock	0.3	0.4	0.3	0.2	0.2	0.3
Informal sale	10.4	4.5	6.9	4.8	6.8	5.9
Handicrafts	3.2	1.1	3.0	3.0	2.7	2.4
Transport	3.4	1.1	1.9	1.6	1.7	1.7
Salaried work	7.8	4.0	4.2	4.2	5.1	4.5
Pension	0.8	0.3	0.4	0.3	0.1	0.3
Own Business/Self employed	3.8	2.0	0.9	1.2	1.7	1.6
VUP Public works	2.3	3.4	4.8	2.4	1.8	3.1
VUP Direct Transfers & other social transfer	0.6	3.2	2.8	3.3	2.1	2.7
Remittances from friends and relatives	0.9	0.3	0.4	0.3	0.6	0.4
No other livelihood activity	28.7	39.0	29.5	38.1	36.7	35.6
Total	100.0	100.0	100.0	100.0	100.0	100.0

Source: NISR, AHS 2020

CHAPTER 3: AGRICULTURAL LAND ACQUISITION

3.1. Access to agricultural land

Access to agricultural land refers to the right by households to acquire land for agricultural purposes either owned or rented. Agricultural land includes cultivated land, land left fallow, pasture land and land under forests cultivation.

Table 14 shows that 87.6 percent of agricultural households possess their own agricultural land. However, the findings indicate that 49.5 percent of agricultural households' access agricultural land through rent. Although a large share of agricultural households operates on their own land, 37.1 percent of them still rent additional land to complement their own land. In terms of gender and land ownership, the results revealed no gap between men and women since the figures are closely equal. However, there is a considerable difference between men and women when it comes to access land through renting.

Table 14: Percentage of agricultural households who accessed agricultural land by land ownership and province

	Ownership type			Households who accessed agricultural land (,000)
	Own land	Rented land	Complemented own land with rented land	
Rwanda	87.6	49.5	37.1	2,270
By province				
Kigali	69.4	53.7	23.1	80
South	88.7	55.8	44.5	623
West	90.8	44.7	35.5	522
North	94.5	39.6	34.1	430
East	81.5	53.4	34.9	615
By HHH sex				
Male	87.4	53.2	40.6	1,630
Female	88.3	39.9	28.2	641

Source: NISR, AHS 2020

Table 15 shows that 97.8 percent of agricultural households dedicated their land for cropping, 11.2 percent used the land for fodder crop cultivation, 18.4 percent of agricultural households have a piece of their land under forest plantation, while 1.9 percent of agricultural households have left their land fallow.

Table 15: Percentage of agricultural households by land use type and province

	Agricultural households with at least land used for				Number of agricultural households (,000)
	Cropping	Fodder cultivation	Forest plantation	Fallow land	
Rwanda	97.8	11.2	18.4	1.9	2,322
By Province					
Kigali	93.3	8.3	6.3	1.1	86
South	98.7	10.6	20.5	3.4	631
West	96.4	11.0	19.9	1.1	542
North	98.9	14.9	27.5	1.9	435
East	97.9	9.8	10.3	1.3	628
By HHH sex					
Male-headed	97.8	11.7	19.6	1.9	1,667
Female-headed	97.8	9.7	15.2	1.9	655

Source: NISR, AHS 2020

3.2. Farm size

A household farm, also called land holding, is a collection of all parcels operated by household, both owned and rented land. The results indicated that the national average farm size is 0.4 ha. As presented in table 16, results show that 77.2 percent of agricultural households operate on a farm size of less than 0.5 hectares, whereas less than 10 percent of agricultural households have 1 ha and above. Across all provinces, households operate on smaller farm size except in the eastern province whereby 15 percent operate on-farm ranging from 1 ha and above.

Table 16: Percentage of agricultural households accessing land by farm size categories and province

Province	Average farm size (in ha)	Farm structure					Number of agricultural HHs with access to agricultural land (,000)
		Less than 0.5 ha	0.5 to 1 ha (exc.)	1 to 5 Ha (exc.)	5 ha and above	Total	
Kigali	0.4	81.3	11.7	6.7	0.3	100.0	80
South	0.4	82.3	10.7	6.6	0.5	100.0	607
West	0.4	78.8	14.4	6.6	0.2	100.0	519
North	0.4	82.1	10.9	6.8	0.2	100.0	421
East	0.5	68.1	17.6	13.8	0.6	100.0	605
Rwanda	0.4	77.6	13.5	8.6	0.4	100.0	2,232

Source: NISR, AHS 2020

3.3. Right to land

Table 17 reports that 94.3 percent had access to use the household land for agriculture while 76.1 percentage of farmers reported having the right to sell or use the land as a guarantee for a loan. The findings indicate equal right to access land as well as a decision over land resources for both male and female farmers.

Table 17: Percentage of farmers with right to land

	Percentage		Number of farmers (,000)
	Access to use land	Right to sell/use the land as a guarantee for a loan	
Rwanda	94.3	76.1	3,832
By Province			
Kigali	90.2	60.0	126
South	96.6	78.5	1,028
West	91.5	79.0	852
North	96.6	82.4	761
East	93.2	68.9	1,065
By Farmers sex			
Male	93.7	77.0	1,665
Female	94.8	75.4	2,168

Source: NISR, AHS 2020

CHAPTER 4: CROPS AND FARMING PRACTICES

4.1. Crop produced in 2019/2020 agricultural year

The following section summarise the distribution of the production of staple crops during the agricultural year 2019/2020. Table 18 shows that, bean crop was grown by 88.6 percent of agricultural households. Other crops frequently grown include maize (67.7 percent), cassava (45.6 percent), sweet potato (44.6 percent), banana (40.2 percent), sorghum (20.9 percent), Irish potato (15.5 percent) vegetables (14.0 percent), yams and taro (13.2 percent), soybean (12.3 percent), groundnut (6.3 percent), pea (5.8 percent), wheat (3.6 percent) and paddy rice (3.2 percent).

Table 18: Percentage of households producing staple crops by crop type and province

	Province					
Crop name	Kigali	South	West	North	East	Rwanda
Cereals	67.9	65.8	70.7	81.0	88.3	76.0
Maize	60.7	54.7	69.1	66.0	82.1	67.7
Paddy rice	1.4	6.8	1.1	0.1	3.9	3.2
Sorghum	16.5	23.3	3.7	30.4	26.9	20.9
Wheat	-	4.0	4.7	7.3	0.3	3.6
Tubers and roots	62.6	83.7	75.4	74.7	57.0	72.1
Irish potato	7.2	11.6	17.3	25.8	11.8	15.5
Sweet potato	27.9	55.7	41.1	58.3	29.1	44.7
Taro	6.1	21.8	14.0	7.8	8.4	13.2
Yams	0.5	0.8	1.1	0.1	0.6	0.7
Cassava	53.2	64.4	42.0	21.5	45.6	45.7
Legumes and pulses	87.1	95.3	85.8	90.2	89.5	90.3
Beans	84.8	94.0	83.0	89.9	87.5	88.6
Bush bean	83.5	67.2	25.0	17.1	83.4	53.0
Climbing bean	3.6	43.2	68.6	80.7	7.7	45.1
Pea	2.3	10.6	4.3	5.8	2.7	5.8
Soybean	8.8	24.5	11.5	2.9	7.6	12.3
Groundnut	6.4	6.5	1.0	1.4	14.0	6.3
Bananas	27.4	42.7	30.2	43.4	45.6	40.2
Cooking banana	20.6	23.0	18.1	31.0	41.3	28.2
Dessert banana	14.7	24.7	15.0	25.1	23.0	21.7
Banana beer	11.7	30.3	20.6	27.4	20.4	24.2
Vegetables	20.7	16.7	15.5	16.4	7.5	14.0
Other crops	2.3	3.6	4.4	4.3	1.5	3.3
No. of crop-producing HHs	80	623	522	429	613	2,268

Source: NISR, AHS 2020

Among vegetable growers, amaranths emerged as the top vegetable type produced by 43.7 percent of agricultural households, followed by cabbage (30.1 percent), tomato (25.6 percent), egg plants (24.2 percent) and carrots (14.6 percent) (Table 19).

Table 19: Percentage of households producing major vegetable crops by vegetable type and province

Province	Provinces					Rwanda
	Kigali	South	West	North	East	
Amaranths	44.8	48.2	52.5	43.8	17.6	43.7
Tomato	41.7	27.7	14	19.2	45.3	25.6
Cabbage	15.7	31.3	32	34.5	22.7	30.1
Eggplant	25	28.7	26.5	18.5	18.8	24.2
Carrot	7.2	22.4	12	12.6	7.4	14.6
Onion	4.9	12.9	11.6	8.6	18.7	12
Sweet pepper	10.5	1.8	3.1	2.8	11.9	4.3
Sugar beet	6.3	5.1	2.1	1.6	6.5	3.8
French beans	12	3.9	0.8	1.5	3.3	2.9
Number of HHs who grew vegetables (,000)	17	104	81	70	46	318

Source: NISR, AHS 2020

The question on whether a household grows fruits or not was asked to both agricultural and non-agricultural households. The results indicate that, 59.9 percent of Rwanda households grow fruits (Table 20). The most important types of fruits grown are avocado (67.3 percent of households), followed by mango (32.7 percent), papaya (20.4 percent), guava (11.4 percent), passion fruits (12.4 percent) and tree tomato (11.8 percent), lemon (9.1 percent) and orange grown by 7.8 percent of households (Table 21).

Table 20: Percentage of households producing fruits by province

Province	Percentage of households who grew			Total number of households (,000)
	fruits	permanent fruits trees[2]	temporal fruits	
Kigali	21.6	19.9	4.2	361
South	65.9	54.5	14.7	707
West	61.4	48.7	14.1	624
North	67.8	57.0	24.6	476
East	66.5	54.8	16.6	729
Rwanda	59.9	49.4	15.4	2,898

Source: NISR, AHS 2020

Table 21: Percentage of households per types of fruits produced by province

	Provinces					Rwanda
	Kigali	South	West	North	East	
Avocado	65.1	69.6	65	74.6	63.9	67.3
Mango	49.9	31.5	30	16.7	45.5	32.7
Papaya	34.6	21.3	10.3	13.3	36.9	20.4
Guava	11.7	13.9	11	16	6.4	11.4
Passion fruits	8.6	12.5	10.7	22.7	8.6	12.4
Tree tomato	12	10.6	9.9	17.1	12.3	11.8
Lemon	10.5	9	12.1	6.7	6.3	9.1
Orange	10.2	5.8	12.5	1.9	6.4	7.8
% HHs who grew fruit	21.6	65.9	61.4	67.8	66.5	59.9

Source: NISR, AHS 2020

[2] Permanent fruits are: avocado, papaya, mango, lemon, jack fruits, guava, orange, mandarin, and sugar apple, whereas temporal fruits include passion fruits, pineapple, tree tomato, watermelon, strawberry, gooseberry,

4.2. Use of agricultural inputs

Results show that 44.6 percent of agricultural households used improved seeds. In regards to type of crops, improved seeds were mostly used on maize (61.1 percent), paddy rice (44.7 percent), wheat (29.6 percent), vegetables (23.5 percent) and Irish potato (3.4 percent). Additionally, 83.7 percent of agricultural households used organic fertilizers, 39.1 percent applied inorganic fertilizers while 26.8 percent used pesticides. A large percentage of households (49.3 percent) purchase inorganic fertilizers from agro-dealers, NGOs (33.3 percent), market (6.0 percent), and agriculture cooperative (5.1 percent) (See table 22, 23& 24).

Table 22: Percentage of agricultural households per different agricultural inputs used by province

	Improved seeds	Organic fertilizer	Inorganic fertilizer	Pesticides	Number of crops-producing households (,000)
Rwanda	44.6	83.7	39.1	26.8	2,268
By Province					
Kigali	33.4	78.1	24.2	19.4	80
South	36.1	85.9	32.0	23.8	623
West	48.5	89.2	54.6	30.8	522
North	47.3	92.3	42.7	39.7	429
East	49.6	71.7	32.3	18.4	613
By HHH sex					
Male-headed	47.8	85.5	42.3	30.2	1,628
Female-headed	36.4	79.3	30.8	18.2	640

Source: NISR, AHS 2020

Table 23: Percentage of households who used improved seeds by province and important crop type

	Provinces					
	Kigali	South	West	North	East	Rwanda
Maize	41.9	59.7	65.7	66.0	57.9	61.1
Paddy rice	84.3	31.3	49.7	-	65.6	44.7
Wheat	-	23.0	29.0	36.8	-	29.6
Beans	0.5	0.8	2.0	1.1	1.9	1.4
Irish potato	2.1	5.5	3.7	3.7	0.6	3.4
Soybean	1.7	0.6	1.4	-	9.7	2.3
Vegetables	44.5	20.9	21.1	22.7	27.4	23.5
Other crops	22.0	-	0.9	1.6	7.0	2.1

Source: NISR, AHS 2020

Table 24: Percentage of households who used inorganic fertilizers by province and source of fertilizer

	Source of inorganic fertilizer						No. of HHs who used inorganic Fertilizer (,000)
	Government (MINAGRI/ RAB/ DISTRICT)	Agro-dealers	NGOs	Market	Agriculture cooperative	Other source	
Rwanda	6.0	49.3	33.3	6.0	5.1	0.3	886
By Province							
Kigali	6.0	70.5	6.8	10.4	6.0	0.3	19
South	6.4	36.1	41.4	6.2	9.7	0.3	200
West	4.7	46.0	43.7	4.4	1.1	0.2	285
North	2.7	86.3	3.0	7.4	0.7	-	183
East	10.6	30.7	41.1	6.5	10.1	0.9	198
By HHH sex							
Male	5.1	50.2	33.1	6.1	5.2	0.3	688
Female	9.3	46.0	34.1	5.7	4.6	0.3	197

Source: NISR, AHS 2020

4.3. Agricultural practices

Table 25 shows that 83.8 percent of agricultural households practiced erosion control measures whereby table 26 shows that, cover plants/grasses and trenches emerged are the most frequently applied anti-erosion control measures (68.9 and 26.7 percent respectively). on the other hand, 46.2 percent planted agroforestry trees in their farms and 0.1 percent of agricultural households used mechanical equipment.

In regards to irrigation, 14.6 percent of agricultural households practiced irrigation. 75.9 percent of those who practice irrigation use the traditional irrigation technique. The traditional methods are mostly used by rural small farmers and it is done by using small equipment like watering canes, Jerry can/bassin/bucket, and other local materials that can be available to draw water. Furthermore, water from streams or lakes was the main source of water for irrigation that served 52.4 percent of agricultural households. (Table 26,27 and 28).

Table 25: Percentage of agricultural households per different agricultural practices

	Erosion control measures	Agroforestry trees in their plots	Irrigation	Mechanical equipment	Number of crops-producing households (,000)
Rwanda	83.8	46.2	14.6	0.1	2,270
By Province					
Kigali	66.5	27.8	18.5	0.2	80
South	85.3	44.1	21.8	0.2	623
West	90.5	55.7	9.9	0.2	522
North	94.2	34.6	12.6	0.1	430
East	71.5	50.9	12.2	0.1	615
By HHH sex					
Male	85.2	48.8	16.3	0.2	1,630
Female	80.0	39.6	10.2	0.0	641

Source: NISR, AHS 2020

Table 26: Percentage of agricultural households by types of erosion control measures

Types of erosion control	By Province					Rwanda	By HHs sex	
	Kigali	South	West	North	East		Male-headed	Female-headed
Radical terraces	4.5	6.3	12.4	16	5.3	9.2	9.8	7.6
Progressive terraces	12.4	15.6	5.6	19.1	5.4	11.1	11.2	10.9
Trenches	23.5	30.3	28.6	8.4	34.8	26.7	28.6	21.9
Trees/ Shelter belt	4.9	7.6	10.7	5.5	5.4	7.2	7.8	5.7
Cover plants/ grasses	51.8	74	72.7	80.9	54.4	68.9	70	66.1
Water drainage	2.2	4.2	0.7	1.2	1.9	2.1	2.2	1.9
Mulching	1.3	1.8	1.4	1.2	5.8	2.7	3.2	1.4
Beds /ridges	2.1	3	10.6	18.2	0.6	6.9	7.2	6.2
Other	0.1	0.4	0	0.4	-	0.2	0.2	0.3
Number of HHs who protected soil against erosion (,000)	80	623	522	430	615	2,270	1,630	641

Source: NISR, AHS 2020

Table 27: Percentage of agricultural households who irrigated land by irrigation techniques and province

Province	Traditional irrigation[3]	Modern irrigation techniques				Total	Number of HHs who practiced irrigation (,000)
		Surface irrigation	Flood irrigation	Drip irrigation	Sprinkler irrigation		
Kigali	83.2	10.7	3.8	1.2	1.2	16.8	15
South	78.2	2.6	18.9	-	0.2	21.8	136
West	86.7	4.0	7.9	-	1.4	13.3	52
North	94.0	5.7	-	-	0.3	6.0	54
East	49.6	19.1	23.7	1.4	6.2	50.4	75
Rwanda	75.9	7.4	14.5	0.4	1.8	24.1	332

Source: NISR, AHS 2020

Table 28: Source of water used for irrigation (percentage) by province

Province	Rainwater harvesting	Water treatment plant	Underground water	Lake/ stream water	Water (dam)	Other sources	Number of HHs who practiced irrigation (,000)
Kigali	1.4	11.4	32.1	52.5	2.3	0.4	15
South	0.3	2.4	49.7	43.8	3.5	0.2	136
West	2.8	3.7	28.5	64.5	0.5	-	52
North	0.9	4.4	24.9	67.7	2.1	-	54
East	0.8	7.5	27.9	48.8	15.0	-	75
Rwanda	1.0	4.5	36.6	52.5	5.4	0.1	332

Source: NISR, AHS 2020

The level of use of mechanical equipment is still low (Table 25). This situation is mainly explained by small land operated with 74.7 percent of agricultural households. (Figure 5).

---

[3] The traditional irrigation method refers to the process of application of water to crops through artificial channels using small local receipt/equipment like watering cane, jerrycans, bucket, bassin, and this process needs human or animal labour to function which make it not very efficient. It is not easy to control amount of water and sometimes can cause soil erosion. This irrigation system is mostly used by small rural farmers as it is not expensive as modern method.

Figure 4: Reasons for not using mechanical equipment

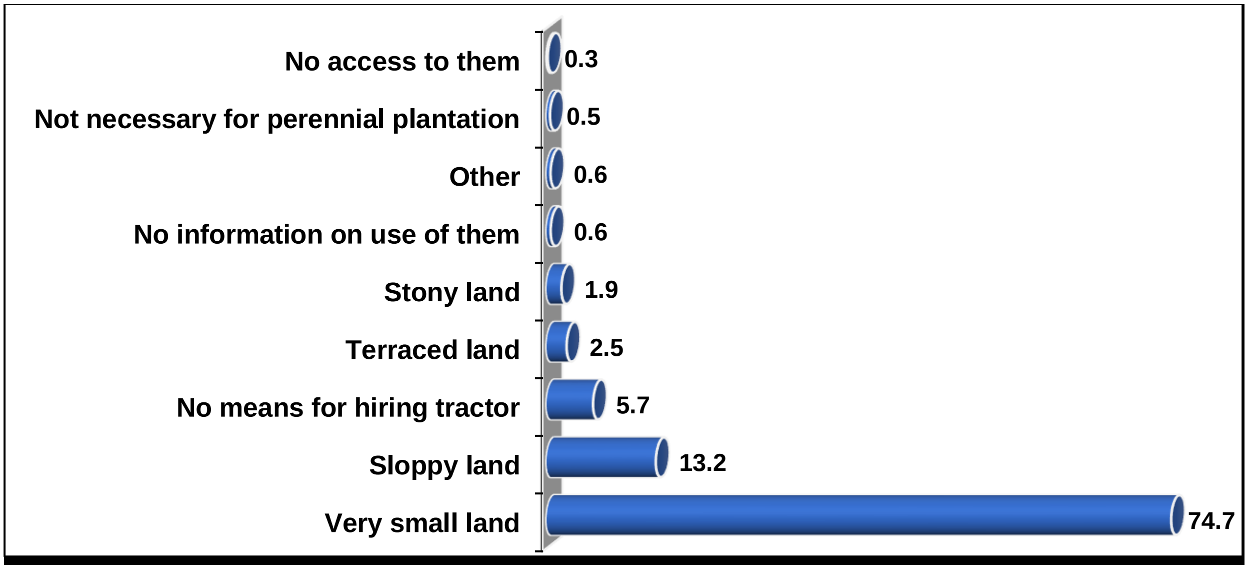

Source: NISR, AHS 2020

4.4. Post-harvest

Crop harvest storage plays an essential part in ensuring domestic food supply. It facilitates farmers to eradicate food insecurity that mostly occurs in a lean season, the time shortly before a new harvest is brought in. By making storage farmers can also be able to improve farm incomes by selling at premium prices when demand exceeds supply later in the post-harvest period. As many farming households use traditional seeds, they prefer to store a part of their harvest to be used as seed later in upcoming seasons.

Figure 5: Percentage of agricultural households who stored crop produce by storage length

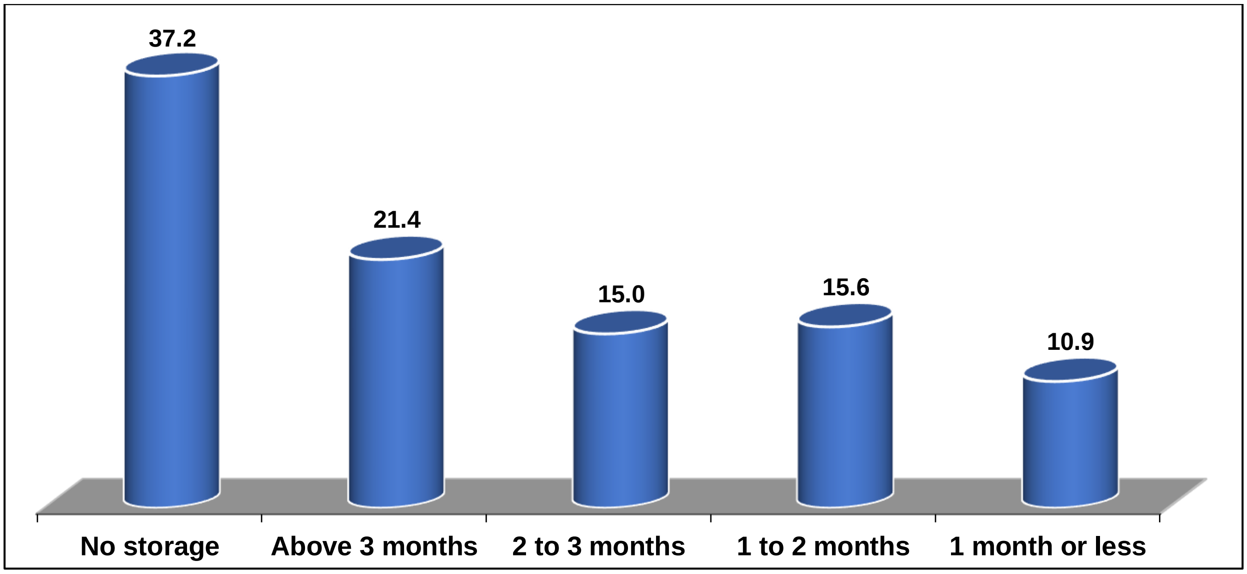

Source: NISR, AHS 2020

Results show that 62.9 percent of agricultural households stored their crop produce after harvest. This is a combination of 10.9 percent of agricultural households who made storage for a period of 1 month or less, 15.6 percent who made storage for a period ranging from 1 to 2 months, 15.0 percent who stored crop produce for 2 to 3 months and 21.4 percent made storage for 3 months and above (Table 29).

Crops that have been stored by a large share of agricultural households were beans (58.6 percent), maize (47.1 percent), wheat (49.4 percent), sorghum (49.3 percent), groundnuts (41.1 percent) and soybean (36.1 percent) (Table 30). The use of improved on-farm storage facilities appeared to be very low since nearly all households (98.5 percent of agricultural households) use own home storage (bags, ground…). Only one percent kept their crop produces in public storage, while 0.6 reserved their harvest in cooperative/private companies' storage facilities (Table 31).

Table 29: Percentage of agricultural households who stored crop produce by storage length and province

	By Storage period						Number of crops-producing households (,000)
Province	1 month or less	1 to 2 months	2 to 3 months	Above 3 months	No storage	Total	
Kigali	7.8	11.9	15.5	27.7	37.1	100.0	80
South	12.1	15.0	12.0	20.4	40.5	100.0	623
West	15.9	19.1	14.3	18.4	32.4	100.0	522
North	8.2	14.7	18.1	20.5	38.5	100.0	429
East	7.7	14.3	16.3	24.8	37.0	100.0	613
Rwanda	10.9	15.6	15.0	21.4	37.2	100.0	2,267

Source: NISR, AHS 2020

Table 30: Percentage of agricultural households who stored crop produce by crop type and province

	Provinces					
	Kigali	South	West	North	East	Rwanda
Beans	65.9	57.5	61.3	55.6	58.9	58.6
Maize	37.4	35.3	51.2	45.9	53.7	47.1
Wheat	-	38	66.6	46.8	17.7	49.4
Sorghum	68.2	47.9	44	58	42.7	49.3
Groundnut	66.1	40.3	48.7	37.9	39.8	41.1
Soybean	45.7	40.6	34	22.6	26.2	36.1
Irish potato	9.5	7.8	35.7	20.6	16	20.7
Pea	13.7	16.3	27.5	23	10.4	18.7
Paddy rice	19.5	16.4	19.2	-	18.3	17.3
Cassava	4.7	6.9	34.1	3.3	5.9	12.0

Source: NISR, AHS 2020

Table 31: Percentage of agricultural households who stored crop produce by type of storage facility and province

	Storage facility type				Number of crops-producing HHs who stored crops (,000)
	Own storage	Public storage	Cooperatives/private companies Storage	Total	
Kigali	99.9	0.1	-	100.0	51
South	98.0	1.2	0.9	100.0	371
West	99.5	0.5	-	100.0	353
North	99.8	0.1	0.1	100.0	264
East	96.9	1.9	1.2	100.0	387
Rwanda	98.5	1.0	0.6	100.0	1,424

Source: NISR, AHS 2020

CHAPTER 5: AGRICULTURE EXTENSION SERVICES

The proximity to extension services forms the basis for empowering farmers with agricultural knowledge, technologies and innovation, consequently leading to the increased agricultural productivity, income and welfare of farmer's family.

5.1 Communication asset ownership

Table 32 shows different communication assets that can enable farmers to gain extension services. Overall, 22.5 percent of agricultural households do not own any communication asset. Among those who own communication assets, 57.3 percent of agricultural households own radio, while 7.8 percent own television. In addition, 70.3 percent of agricultural households own a telephone while 7.0 percent have access to the internet.

Table 32: Percentage of agricultural households owning communication assets

	Communication asset					Number of agricultural households
	Radio	Television	Telephone	Internet	No assets	
Rwanda	57.3	7.8	70.3	7.0	22.5	2,322
By Province						
Kigali	70.8	29.0	85.0	16.8	10.8	86
South	59.1	6.4	64.6	6.2	27.2	631
West	48.3	6.8	70.3	5.9	23.0	542
North	58.6	4.8	70.1	5.5	22.7	435
East	60.3	9.4	74.3	8.3	18.8	628
By HHH sex						
Male-headed	64.4	9.5	76.5	8.1	16.1	1,667
Female-headed	39.1	3.5	54.7	4.1	38.8	654

Source: NISR, AHS 2020

Table 33 indicates that 65.0 percent of agricultural households received extension services. In regards to extension services provided, agriculture practice information was mostly received by 55.8 percent of agricultural households, followed by erosion control measures knowledge gained by 27.1 percent of extension receivers, horticulture skills obtained by 15.5 percent, and integrated pest management skills received by 13.4 percent. In addition, 13.8 percent of extension receivers gained knowledge of using Smart Nkunganire system (SNS) which is a supply chain management system built to digitize the end-to-end value chain of the Agro-Input Subsidy program in Rwanda. Weather and climate information was received by 11.9 percent. At the gender level, among household members, females benefited from extension services more than males with 55.9 and 44.1 percent respectively (Table 34).

Table 33: Percentage of agricultural households who received extension services

	% of HHs by province						% of HH members by sex		Number of HH members who received extension services (,000)
	Kigali	South	West	North	East	Rwanda	Male	Female	
Households who receive extension services (%)	51.1	66.5	63.1	61.6	69.3	65.0			
Type of extension services received									
Agricultural practices	37.6	57.5	53.0	49.2	63.4	55.8	43.7	56.3	2,254
Post-harvest handling and storage	3.7	21.9	8.1	5.3	20.6	14.6	45.7	54.3	607
Erosion control measures	13.8	31.6	31.4	22.5	23.7	27.1	44.9	55.1	1,152
Horticulture skills	10.4	22.3	16.8	6.5	14.4	15.5	42.9	57.1	635
Animal production and nutrition	3.3	12.5	9.3	4.7	8.1	8.8	47.2	52.8	357
Veterinary services	1.9	10.1	7.7	2.5	6.4	6.8	47.4	52.6	281
Agribusiness skills	3.7	9.2	5.9	2.6	6.1	6.2	48.2	51.8	255
Weather and climate information products/ services	11.2	17.7	14.2	4.6	9.3	11.9	46.7	53.3	551
Saving	11.2	23.6	17.4	15.6	19.7	19.1	44.1	55.9	842
Integrated pest management	6.2	19.3	12.5	8.8	12.4	13.4	45.6	54.4	569
Nutrition and food security	10.6	25.2	19.0	20.3	18.1	20.4	42.2	57.8	899
Smart Nkunganire	7.7	21.0	9.0	10.0	13.8	13.7	48	52.0	543
Number of agricultural households (,000)	86	631	542	435	628	2,322			

Source: NISR, AHS 2020

Table 34: Percentage of agricultural households by type and source of extension services

	Source of extension														
Extension service	Government officials (District, Sector, Cell, village	Government extension workers (MINAGRI, NAEB, RAB	NGO/ Company	Farmer Field School facilitator	Farmer /Livestock promoters	Media communication with agriculture technical information	Telephone (Message)	Meeting/ Community work	Friend or family	School	Suppliers (agro-dealer, veterinarian)	Cooperative	Community health workers	Total	Number of Agricultural HH who received extension services
Agricultural practices	24.7	4.0	12.0	4.8	11.8	30.3	0.3	9.4	2.4	0.1	0.0	0.2	-	100.0	1,294
Post-harvest handling & storage	19.4	5.4	10.8	5.0	8.6	34.5	0.4	12.7	2.4	0.4	0.0	0.4	-	100.0	338
Erosion control measures	30.8	2.3	4.6	4.7	10.3	22.3	0.3	22.7	1.5	0.6	-	-	-	100.0	628
Horticulture skills	18.9	2.8	9.4	10.8	22.3	19.8	0.3	11.2	3.6	0.9	-	0.0	-	100.0	359
Animal production and nutrition	24.4	3.8	6.6	2.9	7.7	36.3	0.1	12.7	4.5	1.2	0.0	-	-	100.0	204
Veterinary services	38.0	5.4	3.6	0.9	8.9	25.4	-	14.1	2.6	0.9	0.1	-	-	100.0	158
Agribusiness skills	9.4	5.3	11.2	5.5	10.0	41.0	-	9.6	6.0	1.1	-	0.9	-	100.0	143
Weather & climate information	1.6	0.8	0.5	0.8	2.0	85.0	1.6	1.5	5.1	1.0	-	0.2	-	100.0	277
Saving	26.6	0.5	4.0	2.7	2.0	31.5	0.9	25.4	4.9	1.1	0.0	0.2	0.1	100.0	444
Integrated pest management	13.5	4.0	12.4	5.3	18.3	31.6	0.1	11.0	2.6	0.7	0.2	0.3	-	100.0	311
Nutrition and food security	16.9	0.4	3.7	10.0	13.7	25.8	0.2	21.9	2.3	1.2	-	0.1	3.8	100.0	473
Smart nkunganire	11.7	3.6	15.2	4.2	17.6	34.3	1.6	8.8	2.1	-	0.5	0.6	-	100.0	318
Overall	21.2	3.0	8.4	5.1	11.4	32.3	0.5	14.0	2.9	0.6	0.1	0.2	0.4	100.0	

Source: NISR, AHS 2020

5.2. Farmers' community groups membership

In Rwanda, an agricultural cooperative is widely considered as a vital foundation that can help smallholder farmers to overcome constraints that hinder them from taking advantages of their business. Participation in cooperative or other community group creates a platform for knowledge sharing among farmers, additionally, empowers economically smallholder farmers through enhancing their collective bargaining power, thus reduce risks of market failure.

Findings show that 12.6 percent of agricultural households belong to agricultural cooperatives. Crop producer's cooperative emerged as the cooperative type with the highest agricultural households' members (85.4 percent), while only 11.7 percent belong to livestock cooperatives (Table 35).

Table 35: Percentage of agricultural households by type of cooperatives

	Agricultural cooperative type					HHs with at least one member belonging to agriculture cooperative (,000)
	Crop producers	Livestock producers'	Water users'	Apiculture	Fishery	
Rwanda	85.4	11.7	1.4	0.7	0.8	290
Province						
Kigali	87.3	7.3	2.6	2.7	-	6
South	92.4	5.2	2.3	0.1	0.0	107
West	81.0	14.9	0.4	1.7	2.0	56
North	79.8	19.1	-	1.1	-	41
East	81.9	14.7	1.6	0.4	1.5	80
HHH sex						
Male	84.8	12.0	1.7	0.7	0.8	225
Female	87.4	10.8	0.4	0.8	0.7	66

Source: NISR, AHS 2020

Moreover, various factors associated with agricultural households' decisions to join agricultural cooperatives were explored; 73.6 percent of agricultural households join cooperatives purposely for gaining and share farming knowledge, 56.3 percent consider cooperative as the adequate environment for commercialization of their products. In addition, 55.3 percent of agricultural households stated that cooperatives are a convenient channel to access agricultural inputs. Among other benefits include easy access to financial services and storage facilities with 20.1 and 19.4 percent respectively (Figure 6).

Figure 6: Motivations of joining the agricultural cooperative

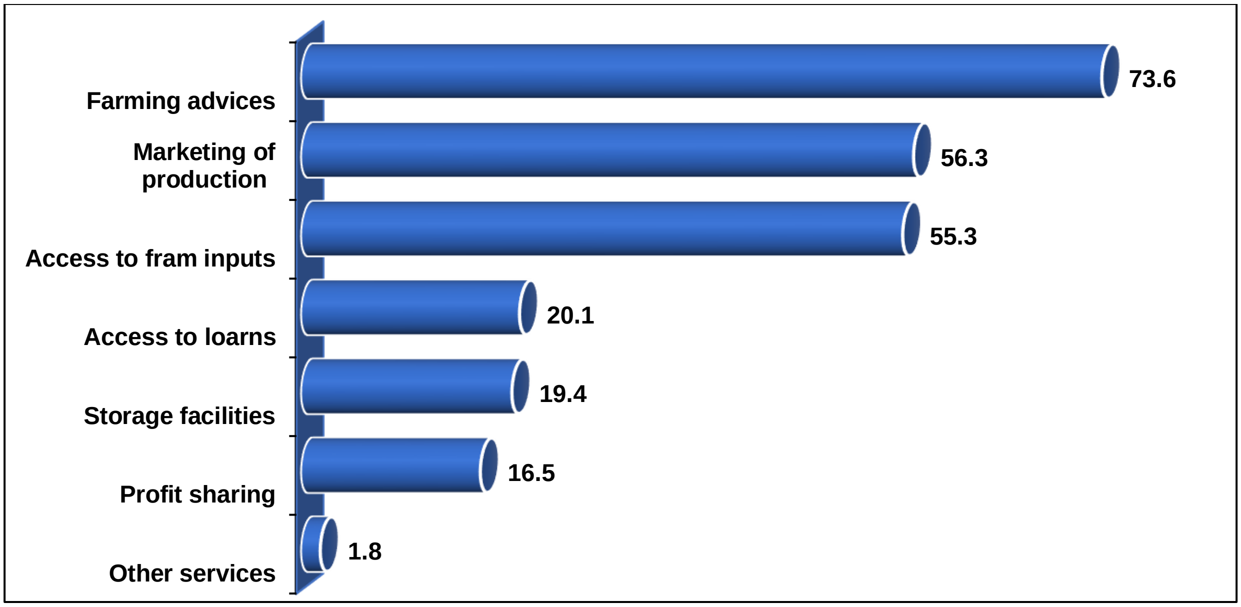

Source: NISR, AHS 2020

Rwanda puts much effort into agricultural extension services to help farmers to improve farming productivity through farming professionalization. Rwanda Agriculture and Animal Resources Development Board (RAB) is promoting Twigire muhinzi program, an extension model based on the farmer-to-farmer extension approach, namely the farmer promoter approach and the Farmer Field School approach.

Table 36 shows that 20.7 percent of agricultural households belong to Twigire muhinzi groups while 11.6 percent of agricultural households belong to Farmer Field School (FFS).

Table 36: Percentage of agricultural households who belong the community groups by province

	Agricultural households who belong to			Total number of agricultural households (,000)
	Agricultural cooperatives/ Association	Twigire muhinzi / mworozi group	Farmer Field School	
Rwanda	12.5	20.7	11.6	2,322
Province				
Kigali	7.4	8.6	11.5	86
South	17.0	19.8	10.4	631
West	10.3	24.8	14.9	542
North	9.3	18.6	6.3	435
East	12.8	21.2	13.5	628
HHH sex				
Male-headed	13.5	22.4	12.7	1,667
Female-headed	10.0	16.4	8.5	655

Source: NISR, AHS 2020

CHAPTER 6: AGRICULTURE POLICIES AND PROGRAMS

6.1 Land consolidation

Figure 7 shows that 24.5 percent of agricultural households' operated farms are located in land-consolidated sites. Across all provinces, North has the largest share of households cultivating in land consolidated sites (32.3 percent).

Figure 7: Percentage of agricultural households operating in land consolidated site by province

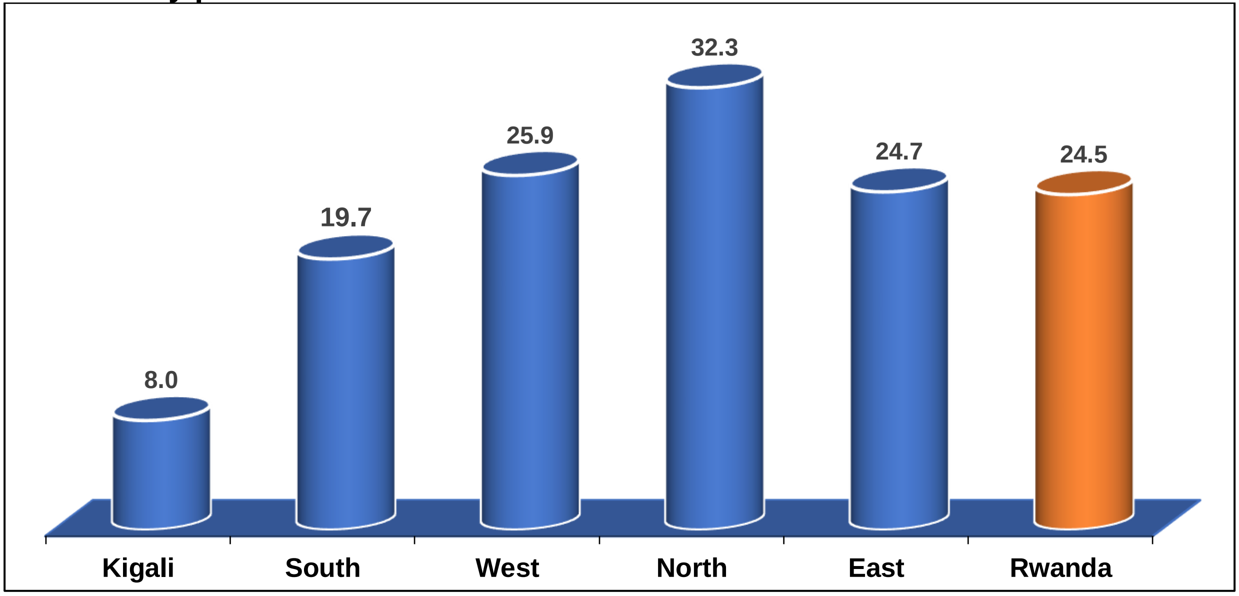

Source: NISR, AHS 2020

Figure 8 shows that, out of total cultivated land, 20.8 percent was covered in land consolidated sites. Southern and Northern provinces have the highest percentage of cultivated land embedded in land consolidation sites (23.6 and 22.9 percent respectively).

Figure 8: Percentage of cultivated land under land consolidation

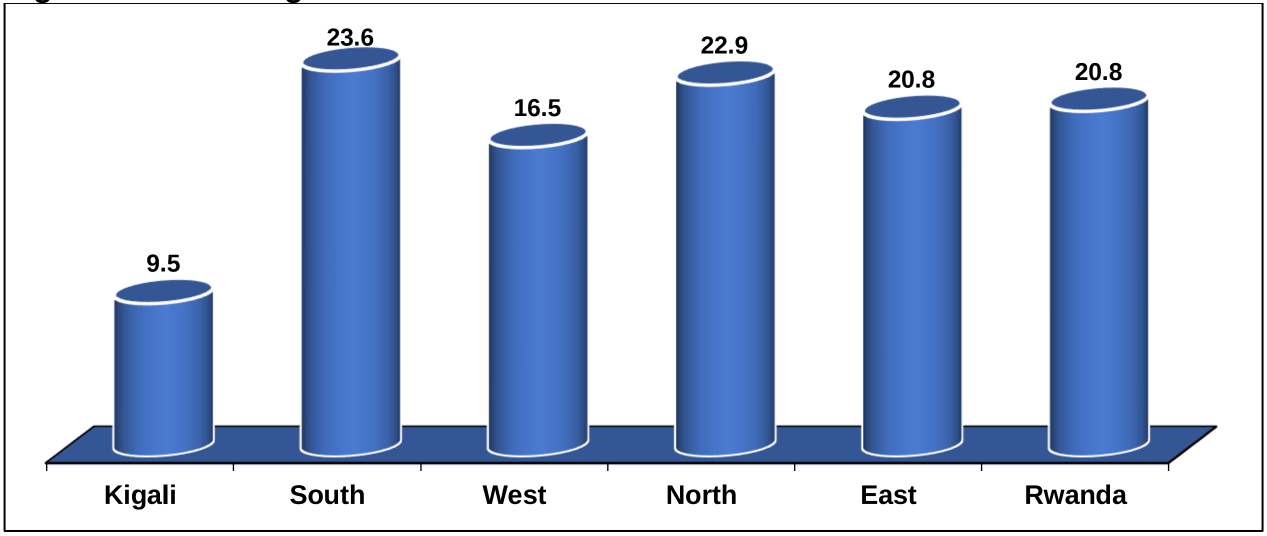

Source: NISR, AHS 2020

6.2. Operating in Kitchen gardens

Figure 9 shows that, 36.3 percent of households (both agricultural and non-agricultural households) own kitchen garden. Northern and western provinces have more share of households (41.4 and 40.1 percent respectively) with kitchen garden compared to other provinces.

Figure 9: Percentage of households with kitchen garden by province

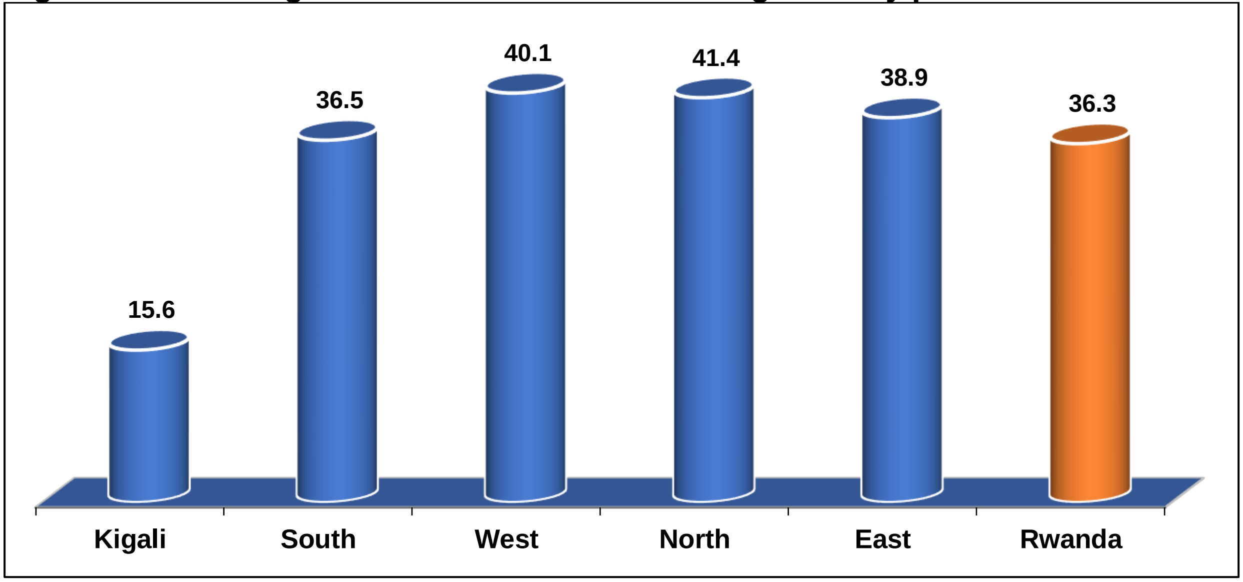

Source: NISR, AHS 2020

6.3. Agriculture insurance programs

The level of agricultural households engaged in crops or/and livestock insurance is relatively low. Survey findings show that 1.0 percent of agricultural households managed to secure crop/livestock insurance (Figure 10).

Figure 10: Percentage of agricultural households with agriculture insurance

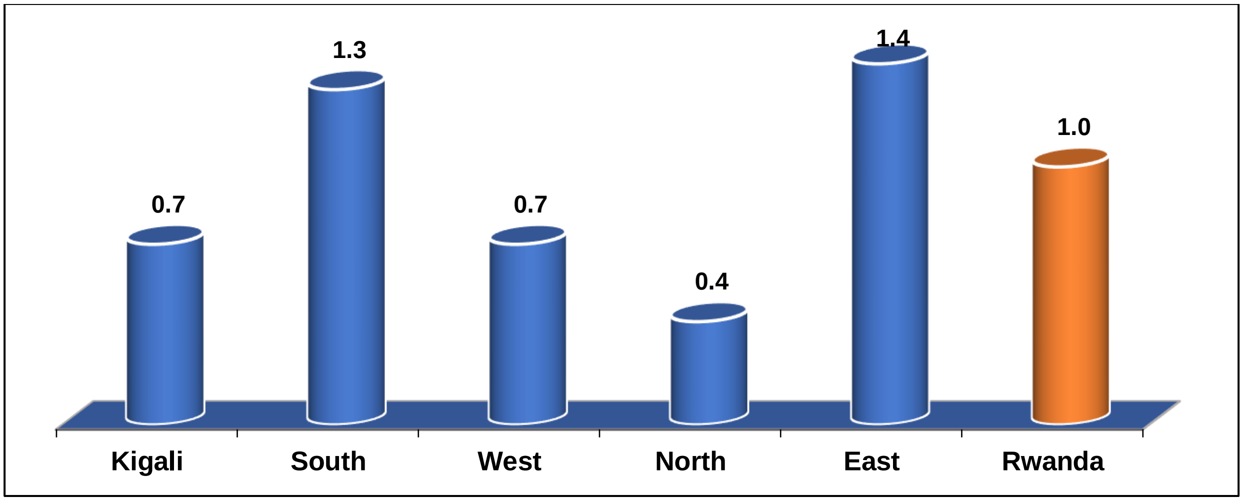

Source: NISR, AHS 2020

6.4. Market linkage (contract farming and agribusiness)

Figure 11 shows that, 3.3 percent of agricultural households signed a contract with wholesalers in 2019/2020 agricultural year.

Figure 11: Percentage of agricultural households with contract farming

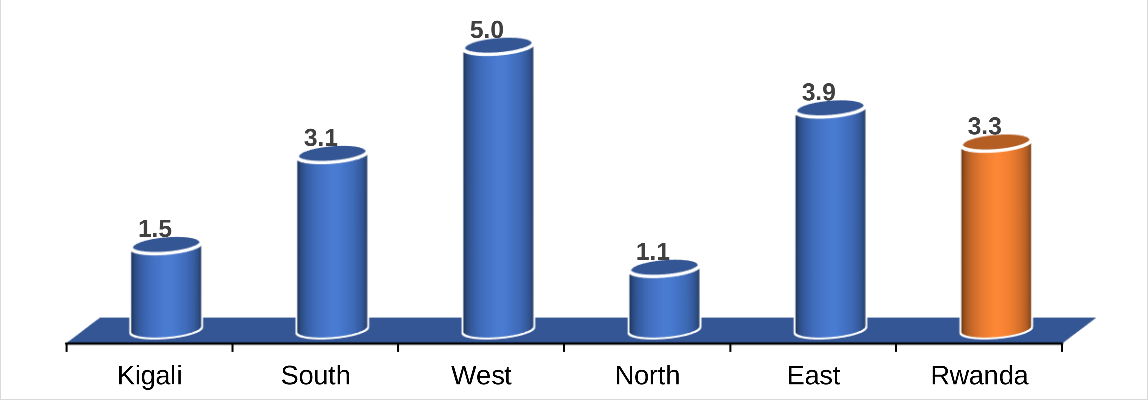

Source: NISR, AHS 2020

6.5. Social protection programs

The One Cow per Poor Family program—called "Girinka," was initiated in 2006 to reduce child malnutrition rates and increase household incomes of vulnerable poor families. The results revealed that 4.1 percent of agricultural households received a cow from Girinka program during the 2019/2020 agricultural year. A large share of a cow from Girinka program was provided by the government (93.4 percent) while non-government organizations contributed 6.6 percent to this program (Table 37).

Table 37: Percentage of HHs who benefited from Girinka program by province and type of providers

	Agricultural HHs who benefited from Girinka program in 2020	Agricultural HHs who still have cow from Girinka program	Type of providers		
			Government	NGO/ company	Total
Rwanda	4.1	85.4	93.4	6.6	100.0
By Province					
Kigali	2.1	92.2	88.8	11.2	100.0
South	4.9	83.1	92.2	7.8	100.0
West	4.2	97.0	97.2	2.8	100.0
North	4.2	88.4	89.4	10.6	100.0
East	3.2	72.7	95.1	4.9	100.0
By HHH sex					100.0
Male	3.8	86.0	93.3	6.7	100.0
Female	4.8	84.3	93.7	6.3	100.0

Source: NISR, AHS 2020

Out of the total households who benefited a cow in girinka program, 85.4 are still have the cow 9 (table 37) while 14.6 percent lost their cow. Among reasons for the loss, death was reported as the main reason associated with cow loss (51.7 percent). Other reasons include lack of fodder and feeding capacity, replacement with other alternative livestock, sold to others, and stolen (Figure 12).

Figure 12:Reasons for abandonment of rearing cow from Girinka program

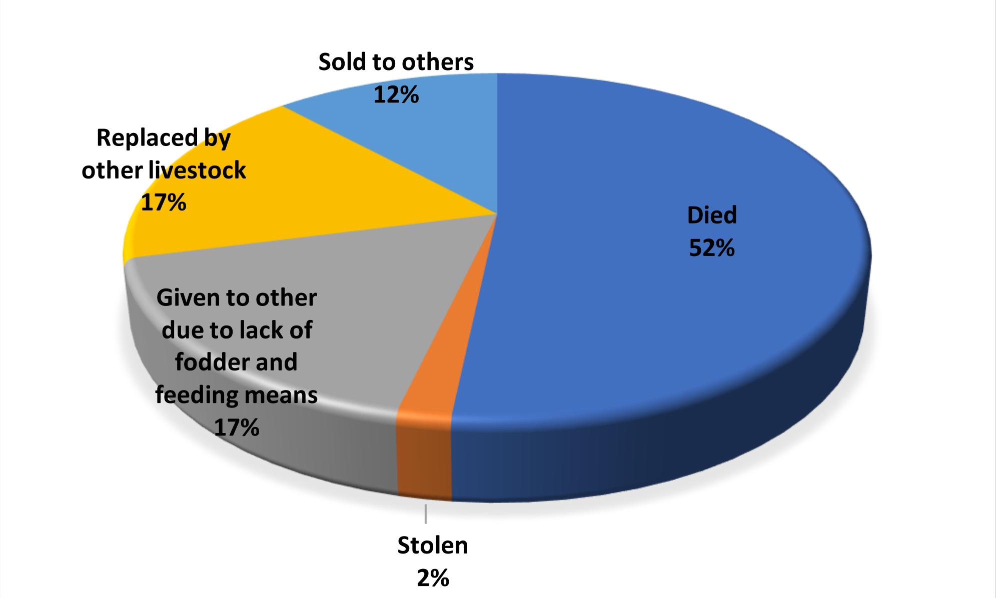

Source: NISR, AHS 2020

Table 38 shows that, 4.3 percent of agricultural households have received small livestock during the 2019/2020 agricultural year. Among distributed small livestock, goats were distributed to 58.5 percent of agricultural households, followed by pigs (21.3 percent), poultry (10.1 percent).

Table 38: Percentage of households who benefited from the small stock program by type of small livestock

	HHs sex		
	Male	Female	Rwanda
HHs who benefited from small livestock program (%)	3.7	5.9	4.3
Type of small livestock			
Goat	53.8	65.7	58.5
Pig	23.2	18.2	21.3
Poultry	11.2	8.6	10.1
Sheep	11.8	6.8	9.8
Other small livestock	-	0.8	0.3
Total	100	100	100

Source: NISR, AHS 2020

The government is the major provider of small livestock, as reported by 64.7 percent of beneficiaries, non-government organizations contributed 33.6 percent while the rest was donated by cooperatives (Figure 13).

Figure 13: Percentage of HHs by source of small livestock received

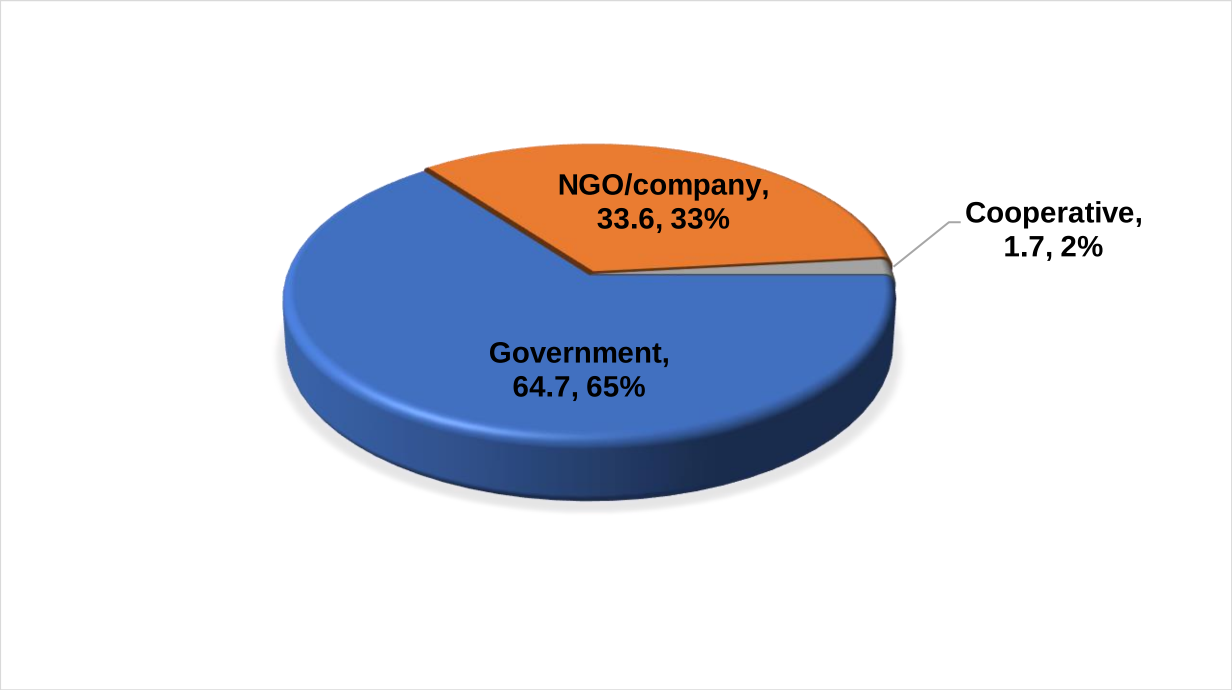

Source: NISR, AHS 2020

CHAPTER 7: FINANCIAL SERVICES AND AGRICULTURAL SUPPORT

Table 39 shows that countrywide, 58.1 percent of all agricultural households own a bank account. The majority of agricultural households (72.3 percent) have a bank account in savings & credits cooperatives, followed by commercial banks and microfinance with 16.2 and 11.5 percent respectively. Besides, 37.9 percent of farmers have a bank account with more male farmers owning a bank account than female farmers (60.2 versus 52.8 percent).

Table 39: Percentage of agricultural households/farmers having bank account by province

	Agricultural HHs with at least one member having a bank account		Farmers having a bank account	
	Percentage	Number (,000)	Percentage	Number (,000)
Rwanda	58.1	2,322	37.9	3,833
Province				
Kigali	66.5	86	45.5	126
South	60.2	631	40.0	1,028
West	60.1	542	39.7	852
North	59.0	435	37.5	761
East	52.4	628	34.0	1,065
Sex of HH/Farmer				
Male	60.2	1,667	47.1	1,665
Female	52.8	655	30.9	2,168

Source: NISR, AHS 2020

Table 40: Percentage of agricultural households by type of financial institutions in which they have a bank account and by province

Province	Commercial banks	Savings & credits cooperatives	Microfinance	Total
Kigali	38.0	45.4	16.5	100.0
South	12.9	76.5	10.6	100.0
West	13.0	71.8	15.2	100.0
North	13.4	74.6	12.0	100.0
East	21.2	71.2	7.5	100.0
Rwanda	16.2	72.3	11.5	100.0

Source: NISR, AHS 2020

Informal financial groups appeared as emerging financial institution whereby 68.5 percent of agricultural households belong to them. These informal groups include tontine/ social solidarity fund/ money lenders or “ikimina”, the latter serve farmers in saving and access to loan. The participation is almost equally in all provinces but slightly lower in the western province. (Figure 14).

Figure 14: Percentage of agricultural households who have at least one member belonging to tontine/solidarity fund(ikimina) by province

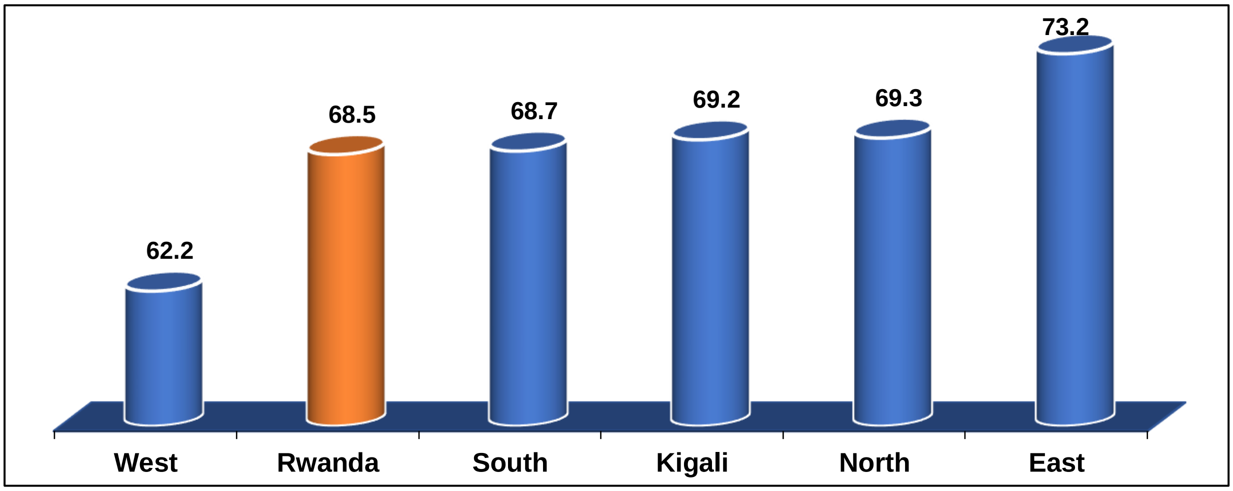

Source: NISR, AHS 2020

7.1. Savings

Table 41 shows that countrywide 68.1 percent of all agricultural households have made savings, while 48.8 percent of individual farmers manage to secure savings. The share of female farmers who made savings is a bit higher than the share of male farmers (49.8 versus 47.5 percent).

Table 41: Percentage of agricultural households/farmers who did savings by province

	Agricultural HHs with at least one member who made savings		Farmers who made savings	
	Percentage	Number (,000)	Percentage	Number (,000)
Rwanda	68.1	2,322	48.8	3,834
By Province				
Kigali	74.7	86	55.3	126
South	72.3	631	51.6	1,028
West	56.2	542	40.6	853
North	67.4	435	48.6	761
East	73.6	628	52.0	1,066
By Sex of HH/Farmer				
Male	71.0	1,667	47.5	1,666
Female	60.6	655	49.8	2,168

Source: NISR, AHS 2020

Table 42 shows that, most agricultural households (72.2 percent) used tontine/solidarity funds for their savings, followed by savings & credits cooperatives (16.7 percent), commercial banks (6.0 percent), microfinance (3.9 percent), and others (Mobile money, Ejo heza and home) with and 1.2 percent (Table 42).

Table 42: Percentage of agricultural households by province and type of financial institutions (formal or informal) in which they made savings

Province	Commercial bank	Saving & credit cooperative	Micro-finance	Tontine/ solidarity fund	Other	Total	Number of agricultural households who made savings (,000)
Kigali	14.0	13.8	4.2	67.6	0.5	100.0	64
South	5.1	17.6	4.0	72.3	1.0	100.0	457
West	5.8	18.2	4.7	69.4	1.9	100.0	305
North	5.1	18.5	5.5	69.8	1.1	100.0	293
East	6.6	13.9	2.1	76.3	1.2	100.0	462
Rwanda	6.0	16.7	3.9	72.2	1.2	100.0	1,580

Source: NISR, AHS 2020

7.2. Access to loan

Table 43 shows that 38.7 percent of all agricultural households had requested a loan while 25.5 percent of individual farmers requested a loan. The share of female farmers who requested a loan is almost the same as one of male farmers (25.6 versus 25.3 percent).

Table 43: Percentage of agricultural households/farmers who requested loan by province

	Agricultural HHs with at least one member who requested for a loan		Farmers who requested a loan	
	Percentage	Number (,000)	Percentage	Number (,000)
Rwanda	38.7	2,322	25.5	3,833
Province				
Kigali	38.2	86	25.2	126
South	44.3	631	29.4	1,028
West	33.0	542	21.8	853
North	36.4	435	23.4	761
East	39.7	628	26.1	1,066
Sex of HH/Farmer				
Male	41.5	1,667	25.3	1,665
Female	31.6	655	25.6	2,168

Source: NISR, AHS 2020

Table 44 shows that, 70.1 percent of agricultural households enquired loan in tontines, followed by microfinance (11.9 percent), relative / friend (9.6 percent), savings & credits cooperatives (3.0 percent), commercial banks (2.9 percent).

Table 44: Percentage of agricultural households by province and source of requested loan

	Provinces					
	Kigali	South	West	North	East	Rwanda
Commercial bank	8.2	1.9	2.3	2.6	4.0	2.9
Microfinance	9.9	10.3	15.7	11.5	11.5	11.9
Credit & saving cooperative	6.5	3.1	4.0	3.2	1.5	3.0
VUP financial services	0.9	2.7	1.4	2.3	0.7	1.7
Ubudehe loan	0.8	0.3	1.0	0.5	0.6	0.6
Employer	-	0.4	0.1	-	0.1	0.2
Relative/friend	6.1	8.3	14.2	13.1	5.9	9.6
Tontine/Solidarity fund	67.6	73.0	61.4	66.8	75.8	70.1
Number of agricultural HHs who requested for loan (,000)	33	279	179	158	249	898

Source: NISR, AHS 2020

Out of the total requested loans, 91.6 percent of them have been approved. Insufficient collateral was the most frequently cited reason for loan rejection (by 28 percent of agricultural households whose loan was rejected), followed by insufficient income (24.0 percent), low financial capacity of the institutions (20.0 percent), unclear purpose of the loan (15.0 percent) and problems related to depts history (13.0 percent) (Figures 15 and 16).

Figure 15: Main reasons for rejecting loans

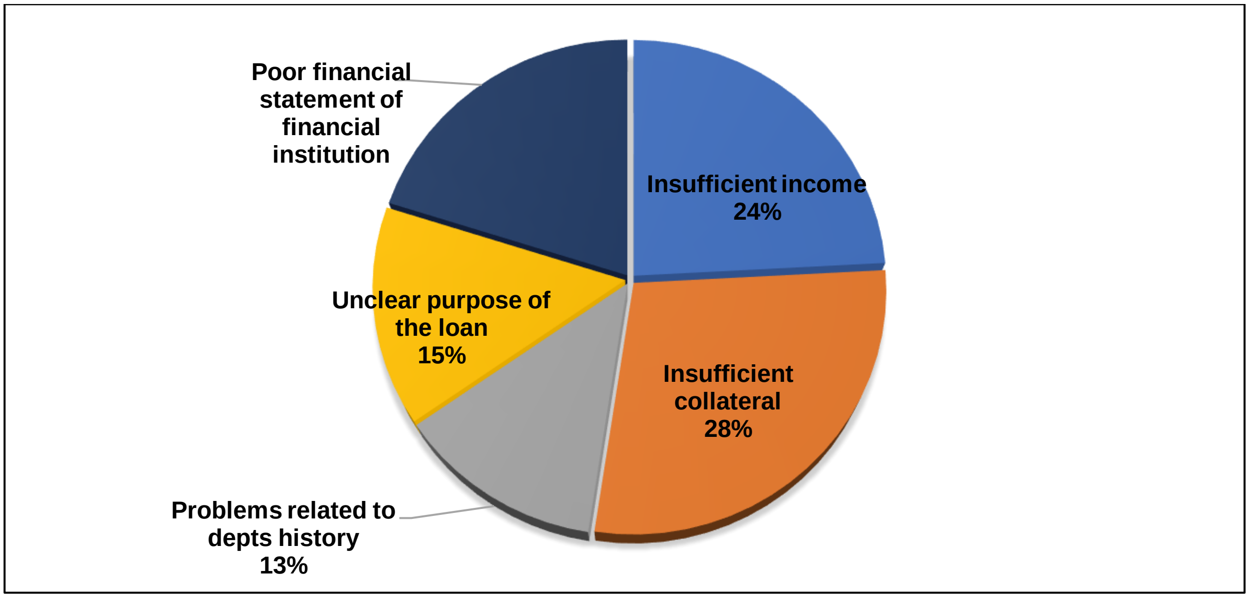

Source: NISR, AHS 2020

Among rejected loans, a high percentage of them (48.0 percent) was planned to be used in agriculture, followed by the ones planned to be used in other households' expenses (25.0 percent), business (15.0 percent).

Figure 16: Planned use of rejected loans

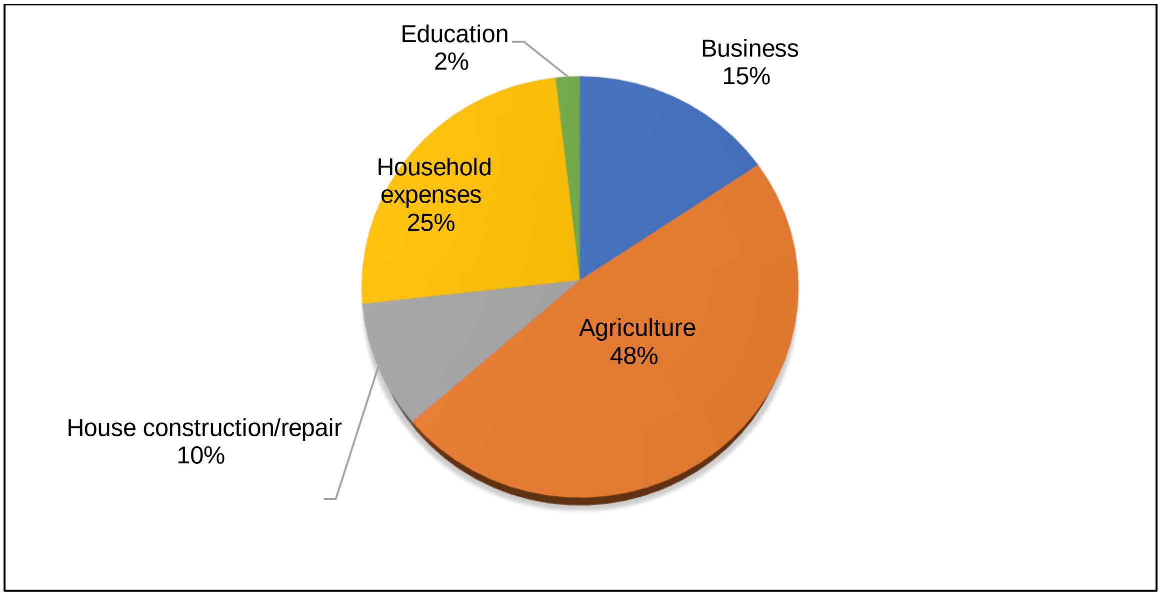

Source: NISR, AHS 2020

7.3. Agricultural funds/support

Figure 17 shows that, on average 2.8 percent of agricultural households received various fund/supports.

Figure 17: Percentage of agricultural households who received any support/fund for grant for agricultural purpose by province

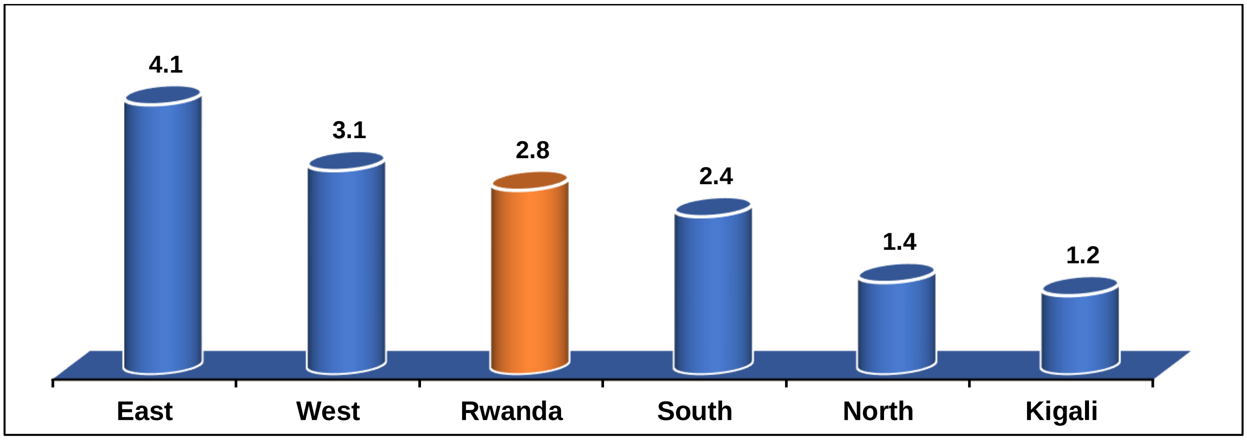

Source: NISR, AHS 2020

Table 45 shows that, out of those who received funs 15.9 percent received money, 37.2 percent received agriculture materials/tools, while 0.7 percent received post-harvest tools.

Table 45: Percentage of agricultural households who received any support by support/fund type and province

	Type of support/fund				
Province	Money	Agriculture materials/ tools	Post-harvest tools	Other	Total
Kigali	14.1	18.6	4.3	62.9	100.0
South	20.4	56.0	-	23.6	100.0
West	24.4	31.2	-	44.4	100.0
North	12.3	42.7	-	44.9	100.0
East	8.7	29.6	1.6	60.2	100.0
Rwanda	15.9	37.2	0.7	46.2	100.0

Source: NISR, AHS 2020

Table 46 shows that, the government provided 80.9 percent of these funds/support, NGOs 12.1 percent, companies 4.0 percent, and friends /relatives 2.3 percent.

Table 46: Percentage of agricultural households who received any support by the source of support/fund and province

	Source of fund					
Province	Government	NGOs	Friends & relatives	Company/ Association	Other source	Total
Kigali	83.5	13.1	-	3.4	-	100.0
South	75.4	16.5	1.5	5.3	1.4	100.0
West	90.6	6.3	-	3.1	-	100.0
North	87.3	8.2	-	4.5	-	100.0
East	76.3	14.1	4.8	3.7	1.0	100.0
Rwanda	80.9	12.1	2.3	4.0	0.7	100.0

Source: NISR, AHS 2020

CHAPTER 8: LIVESTOCK

The livestock section of this report covers several households that raised livestock by numbers, type, breed, age and sex of livestock. Besides, livestock products such as milk, eggs and honey have been reported.

8.1 Livestock numbers

Table 47 shows that, 1.9 million households reared livestock. Major types of livestock reared in Rwanda are cattle with 53.7 percent of agricultural households raising livestock followed by goats (37.7 percent), pigs (33.9 percent), chickens (31.7 percent), sheep (9.9 percent) and rabbits (8.8 percent) (Table 47).

Table 47: Percentage of households raising different types of livestock by province and sex of household head

	Province						By HHH Sex	
Livestock type	Kigali	South	West	North	East	Rwanda	Male-Headed	Female-Headed
Cattle	41.9	58.0	53.4	66.6	40.7	53.7	56.8	45.0
Goats	34.9	43.9	23.9	27.3	52.0	37.7	36.2	41.9
Sheep	2.6	4.1	15.4	22.1	2.7	9.9	10.4	8.5
Pig	17.7	42.5	37.1	24.1	30.6	33.9	36.1	27.9
Chicken	36.4	32.6	28.4	27.6	36.4	31.7	34.7	23.3
Rabbit	12.0	10.0	5.2	11.0	8.7	8.8	9.4	7.0
Other Poultry	3.1	0.9	0.7	0.3	3.5	1.5	1.7	0.8
Other Animal	2.8	1.0	2.2	0.4	0.4	1.1	1.0	1.3
Bee Keeping	1.4	2.5	1.9	2.5	3.4	2.6	3.2	0.9
Households raised Livestock (,000)	52	527	446	360	475	1,859	1,365	494

Source: NISR, AHS 2020

In regard to livestock numbers, AHS 2020 estimates 1.6 million cattle, 1.7 million goats, 0.4 million sheep, 1.2 million pigs, 3.9 million chicken and 0.7 million rabbits (Table 48). Except on cattle where cross breed represents a high proportion (60 percent), goat, sheep, and pigs are still dominated with the local breed (98, 87, 74 percent respectively). Tables 48, 49, 50, 51 and 52 provide more details on the number of livestock by breed, sex, and age.

In terms of livestock products, the AHS 2020 estimated the daily milk production of 3.5 litres per cow on average, annual eggs production of 117 million eggs, and 694 tons of annual honey production (Tables 53, 54 and 55).

Table 48: Number of livestock raised, by type and province

Type of livestock		Province					Rwanda
		Kigali	South	West	North	East	
Cattle	Total	35,795	464,148	366,466	343,366	398,725	1,608,499
	Exotic	5,725	12,888	43,012	20,384	24,931	106,939
	Cross	20,829	268,577	200,740	245,134	230,958	966,237
	Local	9,241	182,682	122,715	77,848	142,836	535,322
Goats	Total	55,900	513,334	242,128	216,035	718,408	1,745,806
	Exotic	60	1,102	455		1,420	3,036
	Cross	1,550	1,990	3,836	4,009	13,091	24,477
	Local	54,290	510,242	237,837	212,026	703,897	1,718,292
Sheep	Total	3,752	41,234	160,162	178,971	36,246	420,365
	Exotic		6	29,484	24,005	176	53,671
	Local	3,752	41,228	130,677	154,967	36,070	366,694
Pig	Total	32,884	403,937	319,031	181,768	309,271	1,246,891
	Exotic	14,906	12,180	34,361	15,786	34,498	111,731
	Cross	6,990	32,704	44,893	73,948	56,028	214,562
	Local	10,988	359,054	239,778	92,034	218,745	920,599
Chicken	Total	323,549	942,087	645,290	622,308	1,421,602	3,954,836
	Broiler	25,344	35,113	33,586	48,407	97,577	240,028
	Layers	150,265	173,731	130,013	187,986	279,143	921,138
	Dual purpose	43,269	148,321	89,633	49,302	178,334	508,858
	Local	104,672	584,922	392,058	336,613	866,547	2,284,812
Rabbit	Total	53,168	208,870	85,111	165,124	237,645	749,917
	Cross	5,600	13,153	8,051	15,269	44,810	86,883
	Local	47,567	195,717	77,060	149,854	192,835	663,034
Guinea pig		14,171	23,239	45,384	8,717	6,362	97,872
Other poultry		7,169	31,413	26,267	4,972	64,709	134,530

Source: NISR, AHS 2020

Table 49: Number of cattle that were reared by agricultural households on the day of the interview, by breeds, sex age and province

Breed type	Gender	Age category	Provinces					Rwanda
			Kigali	South	West	North	East	
Exotic cattle	Male	Calves <=12 months	431	2,164	7,514	1,981	3,741	15,831
		Steers 13-24	173	75	2,049	264	1,244	3,805
		Bulls Above 24	51	19	465	226	46	807
	Female	Calves<=12 months	1,506	1,985	8,766	6,239	4,581	23,077
		Heifers 13-24	1,226	2,640	5,773	3,637	3,320	16,596
		Cows Above 24	2,338	6,005	18,445	8,037	11,998	46,823
Cross cattle	Male	Calves <=12 months	3,102	46,523	39,921	49,953	34,636	174,134
		Steers 13-24	437	7,108	11,729	16,947	11,079	47,300
		Bulls Above 24	98	3,490	3,135	2,826	3,020	12,569
	Female	Calves <=12 months	3,315	55,216	41,494	42,531	40,771	183,327
		Heifers 13-24	4,794	47,261	25,563	38,147	42,331	158,097
		Cows Above 24	9,082	108,979	78,898	94,730	99,121	390,810
Local cattle	Male	Calves <=12 months	1,710	30,752	25,614	16,228	16,928	91,233
		Steers 13-24	264	6,860	8,553	6,116	6,957	28,750
		Bulls Above 24	108	650	1,772	1,553	1,508	5,590
	Female	Calves <=12 months	1,717	37,711	19,838	12,767	23,998	96,031
		Heifers 13-24	1,135	34,140	18,556	10,726	24,778	89,334
		Cows Above 24	4,308	72,570	48,382	30,458	68,667	224,384
Total	Male		6,373	97,640	100,752	96,094	79,160	380,020
	Female		29,421	366,507	265,715	247,271	319,565	1,228,479

Source: NISR, AHS 2020

Table 50: Number of goats that were reared by agricultural households on the day of the interview, by sex age and province

Gender	Age category	Provinces					
		Kigali	South	West	North	East	Rwanda
Male	Kids	7,163	59,733	28,217	26,380	96,525	218,018
	Buck/bulls	4,073	15,501	12,409	12,039	49,536	93,557
	Total	11,235	75,234	40,626	38,418	146,061	311,575
Female	Kids	8,761	99,441	43,590	39,308	134,144	325,244
	Does/namies	35,904	338,660	157,911	138,308	438,204	1,108,987
	Total	44,665	438,101	201,501	177,617	572,347	1,434,231

Source: NISR, AHS 2020

Table 51: Number of sheep that were reared by agricultural households on the day of the interview, by sex age and province

Gender	Age category	Provinces					
		Kigali	South	West	North	East	Rwanda
Male	Ram/lamb	459	5,928	20,406	23,861	3,734	54,388
	Ram/tup	554	3,206	11,376	14,084	3,061	32,281
	Total	1,013	9,134	31,782	37,945	6,795	86,669
Female	Lam	651	7,532	27,472	28,020	5,473	69,147
	Ewe	2,088	24,569	100,907	113,006	23,978	264,548
	Total	2,739	32,100	128,380	141,026	29,451	333,696

Source: NISR, AHS 2020

Table 52: Number of pigs that were reared by agricultural households on the day of the interview, by sex age and province

Gender	Age category	Provinces					
		Kigali	South	West	North	East	Rwanda
Male	Kids	7,455	115,995	77,955	43,314	83,571	328,291
	Buck/bulls	3,258	36,696	40,528	23,140	27,818	131,440
	Total	10,713	152,690	118,483	66,455	111,390	459,731
Female	Kids	10,713	126,721	82,633	46,119	91,409	357,596
	Does/namies	11,458	124,525	117,915	69,194	106,472	429,564
	Total	22,171	251,247	200,548	115,313	197,881	787,160

Source: NISR, AHS 2020

8.2. Livestock products

Table 53: Monthly lactating cows per Province, 2019/20 Agricultural Year

Month	Kigali	South	West	North	East	Total
2019						
September	5,512	43,214	29,803	27,903	44,354	150,787
October	5,812	41,380	29,751	26,354	45,040	148,337
November	5,532	42,655	29,518	27,004	46,046	150,754
December	5,545	43,952	33,665	24,604	50,937	158,703
2020						
January	6,250	46,005	36,483	24,480	50,153	163,371
February	6,053	46,963	35,100	25,695	53,423	167,235
March	6,665	49,210	36,528	30,377	47,005	169,784
April	6,582	52,958	38,512	31,106	46,694	175,852
May	6,356	49,645	37,626	30,665	44,999	169,290
June	6,054	46,083	38,007	31,915	43,134	165,194
July	6,089	45,012	35,643	34,044	44,380	165,169
August	6,080	46,074	39,529	40,392	45,384	177,459

Source: NISR, AHS 2020

Table 54: Average milk production in litters per cow per day by province

Month	Kigali	South	West	North	East	Total
2019						
September	5.3	3.1	3.6	3.5	4.4	3.7
October	5.3	3.1	3.9	4.0	4.5	3.9
November	4.8	3.0	3.7	4.0	4.5	3.8
December	4.7	2.9	3.4	3.9	4.4	3.6
2020						
January	4.6	2.9	3.2	4.2	4.3	3.6
February	4.9	3.1	3.5	4.0	4.0	3.6
March	5.0	3.0	3.5	4.0	3.8	3.6
April	5.3	3.0	3.4	4.1	3.8	3.6
May	5.1	2.9	3.2	3.7	3.6	3.4
June	4.7	2.7	3.0	3.5	3.4	3.2
July	4.1	2.6	3.0	3.3	3.1	3.0
August	4.0	2.4	2.6	2.9	2.8	2.7
Rwanda	4.8	2.9	3.3	3.7	3.9	3.5

Source: NISR, AHS 2020

Table 55: Milk utilisation (in %)

Province	Quantity consumed by HH	Quantity sold	Quantity given to others	Quantity used in other form
Kigali	29.0	64.7	4.8	1.6
South	56.0	32.9	10.4	0.7
West	37.1	50.6	10.1	2.2
North	42.5	50.9	6.1	0.4
East	41.3	51.7	6.5	0.5
Rwanda	43.4	47.6	8.0	1.0

Source: NISR, AHS 2020

Table 56: Average farm gate milk price, year 2019/20 (Frw/litre)

Month	Kigali	South	West	North	East	Total
2019						
September	237	189	156	156	205	182
October	237	187	153	162	206	183
November	244	184	152	163	205	182
December	241	184	147	162	205	182
2020						
January	243	184	138	160	207	180
February	241	186	135	159	208	179
March	237	190	134	161	209	180
April	232	190	145	164	210	181
May	235	191	148	165	211	182
June	231	188	150	162	211	180
July	239	188	145	161	212	180
August	239	190	150	162	217	183
Annual Average	238	187	146	162	208	181

Source: NISR, AHS 2020

Table 57: Annual Egg production (number) per province, 2019/20 Agricultural Year

Province	Total number of eggs produced	Number of eggs consumed by HH	Number of eggs sold	Number of eggs used in another way by the HH
Kigali	19,727,596	1,446,255	17,833,064	448,278
South	16,265,677	4,803,244	9,903,756	1,558,677
West	12,273,704	4,159,498	7,471,393	642,813
North	23,649,643	2,964,671	19,885,155	799,817
East	45,514,275	5,793,198	37,143,184	2,577,893
Rwanda	117,430,896	19,166,866	92,236,552	6,027,478

Source: NISR, AHS 2020

Table 58: Annual honey production [in Kg] and usage during 2019/2020agricultural year

	Honey production			Honey usage		
Province	Traditional beehives	Modern beehives	Total	Quantity Consumed	Quantity Sold	Quantity used in other ways
Kigali	15,871	51,820	67,691	3,597	63,462	632
South	123,256	98,875	222,131	36,756	181,636	3,740
West	97,220	59,258	156,478	25,882	124,141	6,455
North	74,980	8,447	83,426	21,240	57,968	4,218
East	153,563	10,279	163,842	63,760	87,732	12,351
Rwanda	464,890	228,679	693,569	151,235	514,939	27,395

Source: NISR, AHS 2020

8.3 Animal Heath

The results in table 59 shows that 71.3 percent of exotic cattle, 63.0 percent of cross cattle, and 55.1 percent of local cattle have been vaccinated in 2020. Zero grazing was the main feeding practice for cattle and pigs at above 90 percent. The use of improved feeds or and fodder is very low. Exotic pig (14.3) broiler chicken (16.5), layers chicken (12.2) and dual-purpose chicken (11.0) are fed with improved food and less than 10 percent for other breed types (Table 60).

Table 59: Animal Heath and reproduction

Animal type		Percentage of animals: Animal suffered from the disease	Animal treated	Animals vaccinated
Cattle	Exotic	52.8	99.3	71.3
	Cross	39.9	97.1	63.0
	Local	33.4	96.1	55.1
Goats	Exotic	37.5	53.5	
	Cross	24.2	99.8	
	Local	27.6	87.5	
Sheep	Exotic	31	90.2	
	Local	17.7	84.4	
Pigs	Exotic	40.2	86.4	
	Cross	25.2	83	
	Local	25.5	78.4	
Chickens	Broiler	25.3	62.5	7.2
	Layers	29.6	80.9	7.5
	Dual-purpose	33.9	82.1	5.5
	Local	26.4	60.7	0.8
	Duck	6.4	16.2	
	Turkey	38.1	75.7	
	Cross rabbits	27.6	51.8	
	Local rabbits	17.4	30.8	
	Guinea	10.4	23.7	

Source: NISR, AHS 2020

Table 60: Animal feeding practices by livestock type (%)

Animal type		Animals fed with improved feeds / fodder	Major Feeding practice: Pasture grazing	Scave nging	Zero grazing	Animals given water	Watering frequency: Always	Frequently	Rarely
Cattle	Exotic	4.3	3.5	2.3	94.2	97.9	63.9	15.9	20.3
	Cross	2.8	1.7	3.1	95.2	96.4	64.2	16.9	19.0
	Local	1.6	1.9	6.1	92.0	93.7	56.7	20.2	23.2
Goats	Exotic	0.2	1.5	53.5	45.0	79.2	9.3	3.3	87.4
	Cross	9.9	8.8	28.9	62.3	82.8	50.1	12.9	37.0
	Local	0.9	3.6	37.9	58.5	72.7	34.0	17.3	48.8
Sheep	Exotic	0.0	1.1	35.9	63.1	69.0	12.2	23.8	64.0
	Local	0.5	1.5	31.9	66.6	66.1	22.0	21.0	57.0
Pigs	Exotic	14.3	-	0.0	100.0	81.4	79.1	10.2	10.8
	Cross	9.8	-	1.2	98.8	75.9	69.4	11.2	19.3
	Local	5.5	-	4.8	94.9	75.3	67.3	12.3	20.5

Source: NISR, AHS 2020

Table 61: Number of animals born, purchased, sold or consumed by households

Animal Type		Number of animals			
		Born	Purchased	Sold	Consumed by Hhs owners
Cattle	Exotic	31,329	19,966	20,122	1,202
	Cross	262,819	134,980	181,791	4,961
	Local	127,495	57,068	80,021	3,567
	Subtotal	421,644	212,013	281,934	9,730
Goats	Exotic	1,381	166	1,396	262
	Cross	12,333	1,941	4,212	1,323
	Local	773,747	309,764	324,346	36,758
	Subtotal	787,461	311,870	329,954	38,342
Sheep	Exotic	24,845	9,571	9,966	460
	Local	146,602	74,333	73,291	5,078
	Subtotal	171,447	83,904	83,257	5,538
Pigs	Exotic	87,620	53,157	67,142	493
	Cross	147,258	99,111	83,017	1,064
	Local	521,043	436,592	377,335	6,449
	Subtotal	755,921	588,860	527,494	8,006
Chicken	Broiler	26,342	445,260	412,815	11,791
	Layers	136,751	1,057,271	1,457,860	161,249
	Dual purpose	194,106	802,105	531,967	54,917
	Local	2,710,575	624,778	873,785	156,578
	Subtotal	3,067,774	2,929,414	3,276,427	384,535
Rabbits	Cross	115,595	26,128	28,736	11,677
	Local	845,289	316,896	312,747	73,351
	Subtotal	960,884	343,024	341,483	85,028
Others	Duck	76,841	30,189	9,793	5,587
	Turkey	50,312	14,887	19,097	248
	Guinea pig	124,403	33,047	23,311	38,990

Source: NISR, AHS 2020

Annex

Table 62: Socio-demographic characteristics of agricultural households by district

District	Average agric. HH size	Household heads by sex (%)		Agricultural HH members by sex (%)		Agricultural HH members by age group (%)				Agricultural households' members Education attained (%)			
		Male-headed HHs	Female-headed HHs	Male	Female	Below 16 years	16 to 30 years	31 to 64 years	65 years & above	No education	Primary	Secondary	University
Nyarugenge	4.6	74.8	25.2	49.2	50.8	43.2	25.1	29.8	2.0	5.1	62.3	26.5	6.1
Gasabo	4.7	75.6	24.4	47.6	52.4	44.0	25.0	28.9	2.1	7.2	66.5	22.1	4.2
Kicukiro	4.8	77.8	22.2	48.7	51.3	41.1	26.2	29.0	3.7	4.4	59.8	29.2	6.6
Nyanza	4.6	68.4	31.6	49.2	50.8	42.2	26.1	26.8	4.9	12.0	66.3	20.1	1.6
Gisagara	4.3	64.6	35.4	46.3	53.7	42.8	25.7	26.9	4.6	9.4	76.4	13.7	0.6
Nyaruguru	4.9	65.3	34.7	46.4	53.6	42.6	28.9	24.5	3.9	14.8	66.5	17.4	1.3
Huye	4.5	70.4	29.7	47.5	52.5	42.1	22.8	29.4	5.8	9.4	67.4	19.3	3.9
Nyamagabe	4.3	70.5	29.5	48.1	51.9	39.0	26.7	29.2	5.1	11.3	67.8	19.0	2.0
Ruhango	4.3	70.8	29.2	49.2	50.8	40.8	23.1	30.3	5.8	9.1	71.8	17.8	1.4
Muhanga	4.2	73.5	26.5	49.1	50.9	39.7	23.6	32.2	4.6	7.9	75.2	15.6	1.3
Kamonyi	4.2	72.0	28.0	48.2	51.9	39.2	25.1	29.8	5.9	9.7	70.2	19.4	0.7
Karongi	4.5	69.5	30.6	45.3	54.8	41.4	26.1	28.0	4.5	9.3	70.9	18.4	1.4
Rutsiro	4.6	72.5	27.5	47.7	52.3	44.0	26.1	25.1	4.8	13.6	69.8	15.3	1.3
Rubavu	5.3	77.2	22.9	50.3	49.7	46.9	25.7	24.2	3.2	13.4	64.7	19.3	2.7
Nyabihu	4.6	67.3	32.8	47.0	53.0	43.4	26.2	25.9	4.5	11.1	64.1	23.9	0.9
Ngororero	4.6	69.5	30.5	45.3	54.7	42.5	25.9	27.5	4.1	8.1	74.8	16.0	1.1
Rusizi	5.1	73.5	26.5	47.8	52.2	43.4	26.7	25.3	4.5	11.4	66.7	20.9	1.1
Nyamasheke	4.7	70.4	29.6	47.8	52.2	41.8	25.5	27.7	5.0	10.1	71.1	17.8	1.1
Rulindo	4.3	72.5	27.5	47.5	52.6	40.2	26.0	29.5	4.2	10.3	70.9	17.5	1.3
Gakenke	4.1	70.2	29.8	44.7	55.3	37.9	26.9	28.0	7.1	11.3	75.0	13.2	0.5
Musanze	4.6	75.3	24.8	48.3	51.7	41.7	27.2	27.0	4.1	10.0	66.2	20.9	2.9
Burera	4.4	76.3	23.7	47.3	52.7	42.0	28.3	24.7	5.0	13.9	69.6	15.2	1.3
Gicumbi	4.3	73.2	26.8	45.4	54.6	39.0	26.7	28.1	6.2	13.5	66.1	18.9	1.5
Rwamagana	4.7	71.8	28.2	47.7	52.3	42.8	24.8	28.4	4.0	9.0	67.1	21.1	2.7
Nyagatare	4.6	76.9	23.1	47.5	52.5	42.8	27.4	26.8	3.1	13.8	66.7	18.2	1.4
Gatsibo	4.6	74.0	26.0	48.6	51.4	42.0	26.7	27.4	4.0	10.4	71.6	16.6	1.4
Kayonza	4.3	66.0	34.0	49.5	50.5	38.9	28.1	28.3	4.6	12.7	66.0	20.1	1.2
Kirehe	4.5	75.2	24.8	46.5	53.5	43.2	27.0	25.3	4.5	11.5	70.1	17.6	0.8
Ngoma	4.3	74.2	25.8	45.8	54.3	45.0	22.2	28.0	4.8	11.9	71.9	15.2	1.0
Bugesera	4.6	70.5	29.5	48.6	51.4	40.7	28.3	26.7	4.2	12.0	66.0	20.6	1.5
Rwanda	4.5	71.8	28.2	47.5	52.5	41.9	26.1	27.5	4.6	11.0	69.1	18.3	1.6

Source: NISR, AHS 202

Table 63: Percentage of households who engaged in different agricultural activities during the 2019/2020 agricultural year by district

District	Percentage of agricultural households	Percentage of households engaged in crop production	Percentage of households engaged in livestock production	Total estimated number of households (,000)
Nyarugenge	23.8	21.3	13.8	84
Gasabo	27.0	24.6	17.4	182
Kicukiro	18.0	15.4	10.2	96
Nyanza	90.6	88.8	69.9	93
Gisagara	95.0	94.3	70.3	96
Nyaruguru	93.1	91.6	79.1	73
Huye	84.6	81.9	67.4	86
Nyamagabe	91.3	88.9	75.5	92
Ruhango	88.3	86.9	69.4	86
Muhanga	86.3	84.5	74.4	83
Kamonyi	85.5	84.0	67.2	99
Karongi	93.0	92.0	80.2	78
Rutsiro	93.8	92.1	73.3	78
Rubavu	63.0	54.2	37.0	103
Nyabihu	89.5	86.1	68.3	74
Ngororero	92.5	91.4	77.9	95
Rusizi	86.1	85.1	67.2	96
Nyamasheke	94.6	93.2	77.7	99
Rulindo	91.9	91.3	76.7	89
Gakenke	95.5	95.5	86.4	89
Musanze	82.2	79.9	56.2	111
Burera	94.3	93.2	77.1	86
Gicumbi	94.0	93.5	76.8	102
Rwamagana	75.9	73.4	50.8	100
Nyagatare	85.9	83.0	57.6	132
Gatsibo	87.4	84.8	63.8	117
Kayonza	89.2	87.7	59.8	93
Kirehe	93.0	92.1	67.5	92
Ngoma	93.0	91.9	71.6	90
Bugesera	79.9	77.2	56.3	105
Rwanda	80.1	78.1	61.3	2,898

Source: NISR, AHS 2020

Table 64: Percentage of agricultural households by district and agricultural activity types during 2019/2020 agricultural year

District	Total agricultural households (,000)	Percentage of agricultural household who did			
		crop production only	livestock production only	both crop and livestock production	Total
Nyarugenge	20	42.0	10.3	47.8	100.0
Gasabo	49	35.6	9.1	55.3	100.0
Kicukiro	17	43.2	14.4	42.4	100.0
Nyanza	84	22.8	2.0	75.2	100.0
Gisagara	91	26.0	0.7	73.3	100.0
Nyaruguru	68	15.1	1.6	83.3	100.0
Huye	72	20.4	3.2	76.4	100.0
Nyamagabe	84	17.3	2.6	80.1	100.0
Ruhango	76	21.4	1.7	77.0	100.0
Muhanga	72	13.8	2.0	84.2	100.0
Kamonyi	85	21.5	1.8	76.7	100.0
Karongi	73	13.8	1.1	85.2	100.0
Rutsiro	73	21.8	1.7	76.5	100.0
Rubavu	65	41.4	14.0	44.7	100.0
Nyabihu	67	23.7	3.8	72.5	100.0
Ngororero	88	15.9	1.2	82.9	100.0
Rusizi	83	22.0	1.1	76.9	100.0
Nyamasheke	94	17.9	1.5	80.6	100.0
Rulindo	82	16.6	0.7	82.7	100.0
Gakenke	85	9.5	0.0	90.4	100.0
Musanze	91	31.6	2.9	65.5	100.0
Burera	81	18.2	1.2	80.6	100.0
Gicumbi	96	18.3	0.5	81.2	100.0
Rwamagana	76	33.1	3.3	63.7	100.0
Nyagatare	113	32.9	3.4	63.7	100.0
Gatsibo	102	26.9	2.9	70.2	100.0
Kayonza	83	32.9	1.7	65.4	100.0
Kirehe	86	27.4	1.0	71.6	100.0
Ngoma	84	23.0	1.1	75.9	100.0
Bugesera	84	29.6	3.4	67.1	100.0
Rwanda	2,322	23.5	2.5	74.0	100.0

Source: NISR, AHS 2020

Table 65: Number of farmers by District and agricultural activity type

District	Farmers practicing agriculture as main activity (,000)	Farmers practicing agriculture as second activity (,000)	Total farmers (,000)
Nyarugenge	17	10	27
Gasabo	47	27	74
Kicukiro	14	11	25
Nyanza	81	39	120
Gisagara	116	48	163
Nyaruguru	94	18	112
Huye	74	38	112
Nyamagabe	103	51	154
Ruhango	97	26	123
Muhanga	89	15	104
Kamonyi	113	25	138
Karongi	95	22	117
Rutsiro	81	21	103
Rubavu	48	36	84
Nyabihu	70	43	112
Ngororero	77	33	110
Rusizi	104	53	157
Nyamasheke	146	24	170
Rulindo	105	37	142
Gakenke	148	16	164
Musanze	90	59	149
Burera	115	30	145
Gicumbi	128	32	161
Rwamagana	88	32	120
Nyagatare	105	78	183
Gatsibo	137	34	171
Kayonza	94	37	131
Kirehe	111	42	153
Ngoma	115	40	155
Bugesera	113	39	151
Rwanda	2,816	1,016	3,832

Source: NISR, AHS 2020

Table 66: Socio-demographic characteristics of farmers by district (in percentage)

District	Percentage of farmers out of total agricultural working-age population	Percentage of farmers by sex		Percentage of farmers by age group			Percentage of farmers by education			
		Male	Female	16 to 30 years	31 to 64 years	65 years and above	No education	Primary	Secondary	University
Nyarugenge	51.3	45.8	54.2	22.9	71.6	5.4	12.0	62.9	19.0	6.1
Gasabo	57.3	45.9	54.1	27.6	68.8	3.6	13.2	64.3	19.6	2.8
Kicukiro	52.5	44.4	55.6	19.2	71.2	9.6	8.4	61.3	23.6	6.8
Nyanza	53.8	48.0	52.0	20.8	68.6	10.6	23.0	64.0	11.7	1.3
Gisagara	72.3	41.4	58.7	27.4	63.2	9.4	17.1	72.1	10.0	0.7
Nyaruguru	58.9	40.5	59.5	25.9	65.0	9.1	29.9	55.0	13.7	1.5
Huye	59.9	40.6	59.4	18.0	68.7	13.3	18.2	61.5	16.1	4.2
Nyamagabe	70.2	44.5	55.6	29.0	63.0	8.0	18.6	59.3	19.1	3.0
Ruhango	64.8	43.7	56.3	20.2	70.3	9.5	14.5	71.1	13.6	0.8
Muhanga	57.4	42.7	57.3	17.7	73.0	9.3	16.3	70.6	12.1	1.0
Kamonyi	63.8	42.7	57.3	20.8	66.9	12.3	17.6	68.5	13.6	0.4
Karongi	60.8	43.5	56.5	22.4	68.3	9.2	18.3	65.0	15.6	1.1
Rutsiro	54.3	42.4	57.6	27.5	63.0	9.5	27.0	61.6	9.8	1.7
Rubavu	45.8	49.8	50.2	19.6	71.3	9.1	31.2	53.1	13.2	2.6
Nyabihu	65.6	41.8	58.2	28.3	61.8	10.0	20.0	56.1	22.6	1.3
Ngororero	46.9	42.2	57.8	21.8	68.2	10.1	13.4	72.8	11.8	2.1
Rusizi	65.9	44.2	55.8	31.8	59.5	8.7	20.3	61.7	16.6	1.4
Nyamasheke	66.6	44.2	55.8	23.8	65.1	11.1	18.6	70.0	10.6	0.8
Rulindo	67.0	40.6	59.4	29.3	61.4	9.3	18.6	65.2	15.1	1.2
Gakenke	75.3	40.5	59.5	31.0	56.0	13.1	18.2	71.8	9.7	0.3
Musanze	61.0	40.5	59.5	30.7	61.8	7.5	17.0	64.0	15.6	3.4
Burera	70.7	42.6	57.5	36.3	55.0	8.8	22.5	63.9	11.7	1.9
Gicumbi	63.8	45.4	54.6	22.4	64.6	13.0	24.4	62.4	12.1	1.1
Rwamagana	58.6	42.8	57.2	25.3	67.9	6.9	14.6	66.1	15.6	3.7
Nyagatare	61.2	48.3	51.7	32.1	62.7	5.2	22.8	59.2	16.7	1.4
Gatsibo	62.9	44.1	55.9	26.8	65.4	7.9	18.7	68.0	11.9	1.4
Kayonza	60.4	43.7	56.3	28.1	63.3	8.6	24.9	59.9	14.2	1.1
Kirehe	69.8	43.2	56.8	30.9	59.3	9.8	19.5	65.5	14.1	0.9
Ngoma	78.7	44.1	55.9	30.2	60.2	9.6	19.5	65.0	14.3	1.2
Bugesera	66.2	42.7	57.3	28.4	62.5	9.1	21.5	60.9	15.9	1.7
Rwanda	62.9	43.4	56.6	26.6	64.0	9.4	19.8	64.5	14.2	1.6

Source: NISR, AHS 2020

Table 67: Percentage of agricultural households who accessed land by and District and land use type

District	Land-use type: Agricultural households with access to agricultural land	Agricultural households with access to land used for fodder cultivation	Agricultural households with at least land used for forest plantation	Number of agricultural HHs (,000)
Nyarugenge	95.4	13.4	5.7	20
Gasabo	94.7	6.8	8.6	49
Kicukiro	87.0	6.7	2.4	17
Nyanza	98.3	9.4	12.7	84
Gisagara	98.6	1.2	3.1	91
Nyaruguru	98.4	10.1	30.6	68
Huye	98.9	2.5	25.0	72
Nyamagabe	98.7	6.8	41.2	84
Ruhango	99.2	14.4	12.8	76
Muhanga	98.0	39.6	29.6	72
Kamonyi	99.4	4.9	15.3	85
Karongi	98.5	24.8	20.7	73
Rutsiro	98.5	9.4	24.4	73
Rubavu	81.8	5.1	14.2	65
Nyabihu	97.9	11.6	40.3	67
Ngororero	98.3	6.8	23.2	88
Rusizi	99.5	4.2	12.6	83
Nyamasheke	97.8	14.9	12.0	94
Rulindo	99.4	8.3	32.7	82
Gakenke	99.7	19.3	41.8	85
Musanze	96.5	6.0	20.5	91
Burera	99.0	10.9	18.6	81
Gicumbi	99.9	28.4	25.6	96
Rwamagana	97.4	16.5	11.8	76
Nyagatare	96.4	9.3	4.2	113
Gatsibo	97.7	9.5	8.2	102
Kayonza	99.2	6.5	13.8	83
Kirehe	98.3	4.6	15.2	86
Ngoma	99.1	8.3	16.2	84
Bugesera	98.0	14.7	6.5	84
Rwanda	97.8	11.2	18.8	2,322

Source: NISR, AHS 2020

Table 68: Percentage of agriculture households accessing land by District, farm category

District	Average farm size (in ha)	% of HH with access to agricultural land: Less Than 0.5 ha	0.5 to 1 ha (exc.)	1 to 5 Ha (exc.)	5 ha And above	Total	Number of agricultural HHs with access to agricultural land (,000)
Nyarugenge	0.4	78.7	11.8	8.9	0.6	100.0	19
Gasabo	0.3	82.0	12.5	5.4	0.2	100.0	46
Kicukiro	0.3	81.2	9.5	8.7	0.6	100.0	15
Nyanza	0.8	77.6	10.2	10.2	1.9	100.0	81
Gisagara	0.2	92.8	5.3	1.9	-	100.0	89
Nyaruguru	0.4	84.6	7.4	7.4	0.5	100.0	66
Huye	0.4	83.7	9.5	6.3	0.6	100.0	70
Nyamagabe	0.4	78.9	13.7	7.2	0.3	100.0	83
Ruhango	0.4	78.2	15.8	5.5	0.6	100.0	74
Muhanga	0.4	76.3	14.6	8.8	0.3	100.0	69
Kamonyi	0.3	82.7	10.1	7.2	-	100.0	84
Karongi	0.4	74.2	18.2	7.6	-	100.0	71
Rutsiro	0.3	85.6	10.9	3.2	0.3	100.0	72
Rubavu	0.4	84.1	10.3	5.1	0.5	100.0	53
Nyabihu	0.3	80.4	15.7	4.0	-	100.0	65
Ngororero	0.3	80.2	15.3	4.4	-	100.0	86
Rusizi	0.4	77.7	11.2	10.8	0.4	100.0	82
Nyamasheke	0.5	71.2	18.9	9.7	0.2	100.0	92
Rulindo	0.5	76.2	13.5	10.0	0.3	100.0	81
Gakenke	0.4	82.3	10.7	6.7	0.3	100.0	83
Musanze	0.3	88.2	8.5	3.3	-	100.0	88
Burera	0.3	88.5	7.8	3.7	-	100.0	80
Gicumbi	0.5	74.5	14.1	10.4	1.0	100.0	95
Rwamagana	0.4	78.5	12.1	8.8	0.7	100.0	74
Nyagatare	0.7	62.5	16.3	20.3	0.9	100.0	109
Gatsibo	0.6	67.7	20.2	11.5	0.6	100.0	99
Kayonza	0.5	69.6	16.2	13.7	0.5	100.0	82
Kirehe	0.6	61.1	23.4	15.0	0.5	100.0	84
Ngoma	0.5	66.8	21.5	10.9	0.8	100.0	83
Bugesera	0.5	69.5	14.2	15.9	0.5	100.0	82
Rwanda	0.4	77.2	13.6	8.7	0.4	100.0	2,256

Source: NISR, AHS 2020

Table 69: Percentage of crop-producing households by district and crop type

District	Maize	Paddy rice	Sorghum	Wheat	Beans	Bush bean	Climbing bean	Pea	Irish potato	Sweet potato	Taro	Yams	Soybean	Groundnut	Banana	Cooking banana	Dessert banana	Banana beer	Cassava	Vegetables	Other crops	No. of HHs producing crops (,000)
Nyarugenge	53.5	0.1	11.5	-	83.6	81.0	6.0	1.9	3.0	27.4	10.5	0.5	14.0	4.8	25.0	20.7	17.4	13.1	56.0	26.8	2.3	19
Gasabo	62.3	2.2	18.1	-	84.1	83.2	3.3	3.1	10.5	30.7	4.3	0.3	8.8	6.8	31.7	22.4	15.2	12.1	55.0	20.3	2.8	46
Kicukiro	64.6	0.8	18.1	-	88.4	87.6	1.8	0.4	2.0	19.8	5.9	1.0	2.5	7.1	17.1	14.9	9.6	8.8	43.9	14.3	0.6	15
Nyanza	59.1	10.2	23.2	-	94.1	86.7	21.2	11.1	9.1	39.9	23.4	2.1	16.1	19.5	31.4	15.1	14.8	19.2	76.1	18.8	5.8	82
Gisagara	55.6	17.4	27.2	-	97.4	92.0	21.3	4.4	5.6	36.3	13.4	1.8	27.9	5.5	35.4	17.0	20.7	25.5	52.9	10.8	1.5	90
Nyaruguru	74.4	0.5	21.9	10.1	98.1	16.3	94.8	10.6	21.3	81.0	14.4	-	14.0	-	31.9	21.8	16.8	21.5	39.2	12.2	3.6	67
Huye	57.6	15.1	45.3	-	95.8	80.6	46.0	10.8	16.9	55.7	25.9	1.1	32.6	3.2	60.3	37.2	40.7	40.6	67.7	20.1	4.4	71
Nyamagabe	68.7	0.3	27.6	21.6	89.8	27.5	83.8	26.6	19.2	72.4	17.1	-	12.2	1.2	35.0	21.5	18.9	20.1	40.4	17.6	8.0	83
Ruhango	33.0	4.8	20.3	-	95.2	81.4	28.4	8.5	4.6	53.1	18.7	0.1	31.7	10.2	38.5	17.3	24.6	31.8	81.9	9.4	1.2	75
Muhanga	46.9	3.5	1.0	-	88.9	51.7	54.9	8.6	8.2	80.3	58.5	0.7	35.3	0.3	77.0	45.6	46.4	59.4	79.9	35.1	0.9	70
Kamonyi	43.4	0.9	18.9	-	92.7	90.2	8.0	4.0	9.8	37.3	8.3	-	27.3	10.0	37.7	13.7	19.1	29.1	77.6	11.7	3.2	84
Karongi	80.1	-	12.4	2.2	92.8	35.0	81.6	7.5	14.1	65.2	29.6	-	15.7	0.5	37.2	18.2	15.5	31.1	58.9	38.6	0.3	72
Rutsiro	71.8	-	2.8	4.5	72.4	11.8	65.9	7.8	15.6	34.9	14.9	0.7	11.1	-	43.5	32.9	27.4	27.5	31.5	6.0	6.2	72
Rubavu	43.5	-	2.3	-	76.6	4.0	74.0	1.0	37.2	17.7	0.6	-	-	-	12.8	9.1	3.4	6.2	1.9	17.0	9.0	53
Nyabihu	69.3	-	2.8	14.1	77.9	0.6	77.6	8.1	48.4	23.5	2.4	-	1.0	-	17.7	11.6	13.4	13.7	3.2	5.3	8.4	65
Ngororero	71.2	-	2.6	10.2	83.3	10.5	76.6	2.9	11.4	67.7	21.9	0.3	10.9	0.3	32.2	11.8	16.5	26.8	30.1	6.4	1.5	86
Rusizi	82.1	5.9	1.2	0.3	92.7	77.3	36.9	1.7	7.7	27.4	16.6	5.9	13.0	1.3	27.2	17.8	12.5	15.0	86.0	24.8	4.8	82
Nyamasheke	59.2	1.1	2.4	1.5	81.9	23.3	72.1	2.0	1.5	41.0	7.5	0.3	21.7	3.7	34.2	22.1	13.8	19.4	59.2	11.3	3.3	92
Rulindo	70.5	0.3	37.5	1.6	92.8	49.4	63.9	4.5	17.0	69.0	9.9	-	5.0	2.3	67.3	44.2	46.2	48.4	43.4	23.8	1.4	81
Gakenke	80.8	-	1.1	2.3	94.7	8.8	91.4	2.6	9.4	72.9	22.5	0.3	7.5	1.0	71.9	46.1	42.6	59.1	39.6	16.3	8.0	85
Musanze	70.1	-	11.0	9.7	77.1	2.1	76.0	2.0	34.6	24.7	0.6	-	0.4	0.3	8.3	5.8	3.8	3.6	1.1	8.8	9.2	88
Burera	63.3	-	39.3	16.7	90.2	4.1	87.7	8.8	48.3	56.8	3.1	-	-	-	29.3	23.6	9.2	10.8	2.5	19.0	2.8	80
Gicumbi	47.5	-	60.8	6.5	94.8	21.8	84.0	10.9	20.9	68.4	3.7	-	1.6	3.3	42.1	35.8	24.8	17.2	21.7	15.0	0.3	96
Rwamagana	73.4	0.7	38.8	0.4	91.6	90.9	3.3	2.1	22.9	36.0	13.8	0.3	5.9	21.5	48.1	46.7	29.4	23.2	49.8	8.4	0.7	73
Nyagatare	84.1	4.1	19.4	0.3	73.7	71.7	2.7	0.8	1.0	6.4	1.2	-	2.9	10.3	24.6	23.8	13.5	8.2	21.4	2.9	0.5	109
Gatsibo	81.7	3.1	27.6	0.9	85.5	82.3	9.3	6.6	19.2	36.0	8.3	0.3	10.3	21.4	62.0	59.4	26.1	20.5	55.8	8.3	2.6	100
Kayonza	80.7	3.6	42.0	0.3	88.8	88.2	1.7	4.4	21.1	33.2	9.5	-	3.0	7.8	52.2	46.9	21.8	18.5	52.9	7.0	2.4	83
Kirehe	89.6	3.8	24.9	-	92.2	72.8	28.5	1.7	8.1	18.0	4.8	-	8.1	2.7	30.0	25.7	13.3	16.4	41.1	5.2	1.7	84
Ngoma	78.9	7.5	22.2	-	93.6	90.5	4.4	1.3	10.0	33.9	7.6	0.3	9.1	8.5	63.3	56.6	31.2	31.5	46.9	9.2	2.0	83
Bugesera	84.3	4.3	16.6	-	92.4	92.4	4.2	1.4	3.4	47.0	16.4	3.5	14.8	26.8	42.4	32.6	28.7	28.5	57.8	12.8	0.5	82
Rwanda	67.7	3.2	20.9	3.6	88.6	53.0	45.1	5.8	15.5	44.7	13.2	0.7	12.3	6.3	40.2	28.2	21.7	24.2	45.7	14.0	3.3	2,268

NISR, AHS 2020

Table 70: Percentage of households producing vegetable crops by District and crop type

District	Tomato	Cabbage	Onion	Carrot	Eggplant	Sweet pepper	Amaranths	Celery	Spinach	Sugar beet	Garlic	French beans	Number of HHs. that grew vegetables (000)
Nyarugenge	40.9	10.1	1.7	6.3	17.1	7.1	56.7	0.2	7.0	6.4	-	7.5	5
Gasabo	41.2	16.5	6.5	7.7	29.9	11.7	38.5	2.8	2.9	5.9	1.0	14.5	9
Kicukiro	46.1	25.4	5.7	7.6	21.8	13.0	44.7	-	9.4	7.6	-	11.7	2
Nyanza	51.1	31.4	21.2	9.7	17.6	4.7	30.1	1.7	1.5	2.8	1.3	5.2	16
Gisagara	22.5	17.9	11.7	7.8	21.6	-	69.4	-	-	-	-	2.5	10
Nyaruguru	11.6	50.2	19.5	11.4	30.1	-	62.7	2.0	2.3	2.2	-	14.3	8
Huye	25.4	30.6	6.4	14.7	41.0	0.5	46.0	3.4	-	5.2	-	6.3	14
Nyamagabe	4.5	35.4	14.2	41.6	20.9	-	63.2	1.0	-	4.2	7.0	2.9	15
Ruhango	31.2	29.4	3.0	13.9	19.8	3.5	35.4	-	-	10.3	-	5.1	7
Muhanga	28.4	31.5	14.4	41.3	40.3	3.1	52.4	1.0	0.9	4.6	-	-	25
Kamonyi	43.0	24.5	6.1	7.7	22.5	1.1	23.1	-	1.1	14.7	-	1.1	10
Karongi	8.4	42.0	6.2	14.5	30.8	3.6	66.4	1.2	-	3.1	-	1.5	28
Rutsiro	11.4	49.1	-	15.1	47.9	-	47.9	-	-	-	-	5.4	4
Rubavu	11.6	21.4	50.3	20.0	4.5	3.3	4.0	2.5	2.3	5.6	7.8	-	9
Nyabihu	-	39.0	13.0	35.7	17.1	-	5.6	-	-	-	-	-	3
Ngororero	26.0	51.9	20.4	9.3	39.9	-	24.7	-	-	2.0	-	-	6
Rusizi	18.1	15.9	1.2	1.1	31.2	4.9	60.1	-	-	-	0.7	-	20
Nyamasheke	21.9	25.9	12.5	12.2	12.2	2.5	75.4	-	-	2.2	-	-	10
Rulindo	27.2	39.9	4.7	14.5	22.6	8.6	50.4	3.6	1.2	4.7	-	2.5	19
Gakenke	23.5	41.9	3.2	3.1	29.0	-	57.2	-	1.8	-	-	-	14
Musanze	39.8	15.9	11.0	2.4	18.7	-	9.7	-	-	-	20.4	-	8
Burera	1.5	20.4	7.7	3.0	7.7	-	58.3	1.7	1.7	-	-	1.6	15
Gicumbi	11.8	44.8	18.7	35.0	14.4	2.2	24.7	0.6	2.0	1.3	-	2.4	14
Rwamagana	43.2	13.2	1.3	3.6	31.5	24.5	6.7	-	-	3.9	-	4.2	6
Nyagatare	29.7	9.5	53.7	9.5	26.1	9.5	9.5	-	-	-	-	-	3
Gatsibo	30.4	17.8	21.6	7.1	14.3	14.5	38.7	-	3.5	-	-	1.8	8
Kayonza	51.1	30.9	-	4.7	16.4	4.2	8.7	4.4	-	8.7	-	-	6
Kirehe	29.2	18.4	39.1	12.3	10.6	13.8	5.4	-	-	12.3	-	-	4
Ngoma	60.3	36.3	9.5	6.0	6.0	18.1	13.2	3.4	2.9	12.3	-	6.0	8
Bugesera	56.0	23.5	24.6	9.5	26.9	2.1	23.0	-	4.4	7.4	1.2	6.1	10
Rwanda	25.6	30.1	12.0	14.6	24.2	4.3	43.7	1.2	1.2	3.8	1.2	2.9	318

NISR, AHS 2020

Table 71: Percentage of crop-producing households who used different agricultural inputs by District

District	Agricultural HHs who used improved seeds	Agricultural HHs who used organic fertilizer	Agricultural HHs who used inorganic fertilizer	Agricultural HHs who used pesticides	Number of crops-producing households (000)
Nyarugenge	23.4	71.6	22.1	18.7	19
Gasabo	39.3	83.1	23.9	19.4	46
Kicukiro	27.6	70.5	28.0	20.0	15
Nyanza	34.4	80.2	26.3	18.6	82
Gisagara	35.8	77.3	29.0	21.4	90
Nyaruguru	48.7	94.8	59.8	39.0	67
Huye	44.4	90.3	33.9	22.8	71
Nyamagabe	45.5	94.8	48.1	29.2	83
Ruhango	22.0	82.1	16.7	19.6	75
Muhanga	36.7	93.8	29.0	21.6	70
Kamonyi	23.5	77.7	17.8	20.8	84
Karongi	67.2	92.8	58.7	32.2	72
Rutsiro	50.8	93.8	52.5	25.3	72
Rubavu	45.0	63.3	42.3	42.8	53
Nyabihu	36.5	85.4	52.5	58.1	65
Ngororero	60.1	97.8	44.9	26.6	86
Rusizi	45.6	88.6	64.0	22.8	82
Nyamasheke	34.1	92.9	62.2	18.8	92
Rulindo	40.5	96.1	33.5	26.9	81
Gakenke	71.3	98.3	75.2	50.5	85
Musanze	53.0	85.4	45.2	58.6	88
Burera	32.6	91.8	34.4	41.4	80
Gicumbi	39.1	90.4	26.7	22.5	96
Rwamagana	53.1	81.7	36.1	24.1	73
Nyagatare	35.1	55.3	27.4	18.6	109
Gatsibo	41.5	82.7	34.4	17.8	100
Kayonza	50.6	71.9	26.1	15.8	83
Kirehe	56.5	69.8	32.2	19.2	84
Ngoma	65.1	78.7	42.5	17.9	83
Bugesera	51.9	65.7	29.2	16.1	82
Rwanda	44.6	83.7	39.1	26.8	2,268

NISR, AHS 2020

Table 72: Use of agricultural practices among agricultural households (%)

District	Percentage of households who				Number of crops-producing households (000)
	have land protected soil against erosion	planted agroforestry trees in their plots	practiced irrigation	used mechanical equipment	
Nyarugenge	66.2	36.2	20.3	0.2	19
Gasabo	77.9	26.6	20.2	-	46
Kicukiro	31.6	21.1	11.2	0.8	15
Nyanza	83.3	44.4	28.0	-	82
Gisagara	73.0	36.6	21.1	-	90
Nyaruguru	90.6	49.2	17.9	0.5	67
Huye	85.9	44.1	35.7	0.3	72
Nyamagabe	92.1	66.0	15.2	0.5	83
Ruhango	93.3	47.6	15.2	-	75
Muhanga	85.4	33.9	29.3	-	70
Kamonyi	82.0	31.5	13.6	-	84
Karongi	94.4	43.7	17.8	0.3	72
Rutsiro	93.3	54.7	3.7	-	72
Rubavu	89.3	28.7	2.8	-	53
Nyabihu	97.3	56.9	0.8	-	65
Ngororero	96.1	55.5	7.8	0.5	86
Rusizi	78.5	71.5	17.7	0.3	82
Nyamasheke	86.5	66.5	14.3	-	92
Rulindo	96.0	35.6	25.1	-	82
Gakenke	96.5	35.2	16.5	-	85
Musanze	94.6	40.2	6.2	-	88
Burera	92.5	19.5	4.3	0.3	80
Gicumbi	91.7	40.9	11.3	0.3	96
Rwamagana	78.0	58.8	10.0	-	74
Nyagatare	54.8	46.8	6.8	-	109
Gatsibo	87.6	56.3	10.2	0.3	100
Kayonza	63.1	45.7	10.6	-	83
Kirehe	75.5	52.2	18.3	-	84
Ngoma	71.5	47.3	15.3	-	83
Bugesera	72.4	50.0	16.2	0.2	82
Rwanda	83.8	46.2	14.6	0.1	2,270

NISR, AHS 2020

Table 73: Percentage of agricultural households who stored harvested crop produces by storage length and District

District	By Storage period						Number of crops-producing households (,000)
	1 month or less	1.01 to 2 months	2.01 to 3 months	Above 3 months	No storage	Total	
Nyarugenge	8.2	11.6	17.1	22.5	40.6	100.0	19
Gasabo	8.1	12.3	16.5	27.0	36.1	100.0	46
Kicukiro	6.4	11.1	10.5	36.3	35.8	100.0	15
Nyanza	3.7	8.2	10.3	24.0	53.8	100.0	82
Gisagara	15.5	16.1	12.9	13.4	42.1	100.0	90
Nyaruguru	15.4	14.8	7.8	26.8	35.2	100.0	67
Huye	13.3	19.4	9.6	24.7	33.1	100.0	71
Nyamagabe	5.8	14.7	11.4	14.3	53.8	100.0	83
Ruhango	9.7	17.3	18.2	32.3	22.5	100.0	75
Muhanga	11.9	12.8	10.7	4.3	60.4	100.0	70
Kamonyi	21.8	17.3	14.1	24.4	22.5	100.0	84
Karongi	9.3	13.9	5.5	20.0	51.4	100.0	72
Rutsiro	16.7	20.5	15.8	17.5	29.5	100.0	72
Rubavu	17.9	14.0	22.4	16.5	29.1	100.0	53
Nyabihu	10.8	23.6	21.1	20.4	24.2	100.0	65
Ngororero	29.9	24.0	17.3	12.3	16.4	100.0	86
Rusizi	15.3	14.6	14.3	36.6	19.3	100.0	82
Nyamasheke	10.1	21.1	7.6	6.8	54.5	100.0	92
Rulindo	4.2	7.8	18.9	26.8	42.4	100.0	81
Gakenke	6.2	6.2	9.4	21.9	56.2	100.0	85
Musanze	6.8	14.2	14.8	21.0	43.2	100.0	88
Burera	12.7	25.4	31.5	15.6	14.9	100.0	80
Gicumbi	11.0	19.6	16.8	17.4	35.2	100.0	96
Rwamagana	12.0	16.1	23.8	28.4	19.8	100.0	73
Nyagatare	8.2	20.4	18.2	18.9	34.3	100.0	109
Gatsibo	11.4	17.8	12.0	13.5	45.3	100.0	100
Kayonza	5.8	16.0	21.8	35.5	20.9	100.0	83
Kirehe	5.2	12.9	15.1	28.0	38.8	100.0	84
Ngoma	7.2	6.9	10.4	31.5	44.0	100.0	83
Bugesera	3.7	7.2	14.0	22.3	52.9	100.0	82
Rwanda	10.9	15.6	15.0	21.4	37.2	100.0	2,267

NISR, AHS 2020

Table 74: Percentage of agricultural households who own communication assets by District

District	Percentage of HHs by communication asset type					Number of agricultural households (,000)
	Radio	Television	Telephone	Internet	No assets	
Nyarugenge	72.7	32.3	91.9	26.4	4.9	20
Gasabo	70.1	24.5	80.7	12.8	14.7	49
Kicukiro	70.5	37.9	89.1	17.3	6.8	17
Nyanza	62.6	5.7	65.6	7.8	26.4	84
Gisagara	36.6	1.9	52.6	2.1	38.8	91
Nyaruguru	59.9	5.3	63.2	9.7	29.6	68
Huye	64.5	10.6	63.8	10.3	29.2	72
Nyamagabe	58.9	4.6	64.8	8.7	26.9	84
Ruhango	69.3	5.7	65.5	3.8	25.2	76
Muhanga	70.8	8.3	67.7	6.0	23.1	72
Kamonyi	56.0	9.7	74.7	2.7	17.5	85
Karongi	42.8	5.5	66.0	5.1	26.0	73
Rutsiro	32.6	4.0	68.7	6.2	26.8	73
Rubavu	42.5	13.1	75.1	7.0	20.3	65
Nyabihu	59.3	6.4	77.1	5.2	18.4	67
Ngororero	55.1	2.8	64.7	5.9	27.7	88
Rusizi	55.9	11.8	75.9	8.6	19.1	83
Nyamasheke	48.0	5.3	66.9	3.5	21.6	94
Rulindo	69.4	5.1	72.8	2.1	21.3	82
Gakenke	58.9	1.9	64.6	3.9	28.6	85
Musanze	66.0	9.1	78.1	13.4	17.1	91
Burera	53.8	2.5	72.2	4.5	19.7	81
Gicumbi	46.1	5.1	63.4	3.2	26.6	96
Rwamagana	67.5	17.1	83.6	12.9	11.5	76
Nyagatare	61.4	10.0	83.3	11.2	11.0	113
Gatsibo	48.6	6.5	64.2	5.6	27.5	102
Kayonza	59.2	8.1	77.0	4.6	20.1	83
Kirehe	65.5	6.1	66.7	8.1	20.6	86
Ngoma	56.1	6.3	68.1	5.0	23.1	84
Bugesera	66.8	13.0	77.2	10.6	17.6	84
Rwanda	57.3	7.8	70.3	7.0	22.5	2,322

NISR, AHS 2020

Table 75: Percentage of agricultural households who received extension services by extension type, and district

District	Agric. HHs who receive extension services (%)	Type of extension services received												Number of HH members who received extension services (,000)
		Agricultural practices	Post-harvest handling and storage	Erosion control measures	Horticulture skills	Animal production and nutrition	Veterinary services	Agribusiness skills	Weather & climate information products/ services	Saving	Integrated pest management	Nutrition and food security	Smart nkunganire	
Nyarugenge	48.8	41.0	8.3	13.0	12.4	6.3	5.1	5.7	10.3	12.5	7.4	12.3	8.7	20
Gasabo	62.3	46.6	2.4	18.3	12.1	2.6	0.9	3.7	12.5	12.8	7.5	12.1	8.0	49
Kicukiro	22.1	8.3	2.0	2.1	3.2	1.8	0.9	1.5	8.2	5.3	1.3	4.4	5.7	17
Nyanza	67.2	55.2	37.2	46.7	43.4	31.0	33.9	29.7	38.6	44.7	35.4	48.0	30.1	84
Gisagara	49.6	42.1	13.5	13.1	6.9	5.9	9.1	3.2	4.9	3.4	2.8	8.7	9.6	91
Nyaruguru	75.7	64.7	19.7	21.6	29.9	11.2	11.0	7.2	10.3	20.8	27.1	21.5	31.4	68
Huye	73.3	65.1	25.7	35.7	30.0	15.8	5.0	10.9	14.4	32.1	21.5	24.5	18.2	72
Nyamagabe	84.7	72.5	33.4	48.7	18.4	11.2	12.9	9.0	32.1	34.2	26.7	33.8	27.1	84
Ruhango	63.9	51.2	21.7	41.3	24.9	7.5	1.4	3.0	14.0	30.6	16.4	39.5	25.5	76
Muhanga	39.9	36.4	9.0	13.2	7.6	4.4	0.6	1.3	8.3	2.1	0.7	3.3	2.7	72
Kamonyi	77.8	73.0	14.3	31.1	19.5	12.0	4.0	8.2	16.7	21.0	24.2	21.4	23.5	85
Karongi	46.1	41.8	1.2	10.0	4.6	1.7	0.3	0.3	1.2	5.6	4.6	6.3	6.1	73
Rutsiro	53.7	44.2	2.1	20.7	7.5	3.9	1.5	0.3	0.4	7.2	0.3	17.5	5.8	73
Rubavu	33.1	24.6	1.3	4.0	3.6	3.7	2.0	0.6	3.3	1.7	5.7	2.9	2.6	65
Nyabihu	88.5	69.6	19.3	69.9	38.4	25.2	16.9	11.9	56.9	52.2	40.5	45.2	22.4	67
Ngororero	71.5	59.8	3.7	30.5	16.3	7.8	7.1	2.1	17.4	26.4	7.6	40.1	12.8	88
Rusizi	76.2	71.9	25.1	49.0	33.2	19.5	21.6	25.0	18.0	23.7	22.4	19.2	13.8	83
Nyamasheke	67.2	53.3	4.3	33.4	13.3	4.6	3.7	0.8	6.3	6.7	9.1	2.6	1.0	94
Rulindo	66.7	60.4	7.7	21.2	5.3	3.3	2.8	1.7	8.1	18.2	17.3	29.9	10.7	82
Gakenke	74.5	53.3	4.6	26.4	7.2	3.6	2.5	3.0	1.6	14.4	8.6	16.2	34.4	85
Musanze	38.1	26.0	1.2	7.8	6.5	4.9	1.9	1.9	0.3	9.5	4.6	9.0	2.1	91
Burera	56.6	46.1	6.6	25.8	5.9	7.5	4.6	2.5	0.6	13.3	10.8	14.7	2.8	81
Gicumbi	72.5	60.9	6.8	31.4	7.4	4.5	0.9	3.9	11.6	22.4	4.0	31.2	1.2	96
Rwamagana	49.8	47.6	11.5	12.3	9.2	7.0	4.7	4.8	4.8	8.1	6.5	6.3	5.6	76
Nyagatare	70.3	64.0	25.1	33.3	16.5	10.4	7.1	5.1	10.5	22.5	21.1	19.6	15.9	113
Gatsibo	58.5	54.7	13.2	20.2	7.4	4.7	6.3	1.4	3.4	12.9	9.9	8.7	8.0	102
Kayonza	75.6	70.8	20.5	27.6	18.9	11.3	6.3	10.3	11.7	26.8	9.7	21.3	15.8	83
Kirehe	87.9	79.5	24.7	24.4	18.8	5.3	4.6	4.0	4.2	22.5	4.4	23.6	14.1	86
Ngoma	68.3	62.5	31.0	30.7	17.8	11.7	11.7	12.0	18.6	27.1	19.8	20.7	20.3	84
Bugesera	74.2	64.5	16.9	13.6	12.5	6.1	3.7	6.7	12.6	17.3	12.2	26.4	16.5	84
Rwanda	65.0	55.8	14.6	27.1	15.5	8.8	6.8	6.2	11.9	19.1	13.4	20.4	13.7	2,322

NISR, AHS 2020

Table 76: Percentage of agricultural households with at least one member who joined community groups by District

District	Agricultural households who belong to: agricultural cooperatives/ Association	Twigire muhinzi/mworozi group	Farmer field school	Total number of agricultural households (,000)
Nyarugenge	7.0	15.0	7.9	20
Gasabo	8.7	7.9	16.3	49
Kicukiro	4.0	3.2	1.8	17
Nyanza	12.1	39.6	19.0	84
Gisagara	23.7	7.2	7.3	91
Nyaruguru	12.9	17.8	15.0	68
Huye	29.2	15.5	6.7	72
Nyamagabe	14.8	21.9	13.5	84
Ruhango	13.6	26.2	7.4	76
Muhanga	17.6	9.9	6.0	72
Kamonyi	12.0	19.4	8.1	85
Karongi	9.1	19.4	14.2	73
Rutsiro	5.9	19.9	7.6	73
Rubavu	9.2	11.5	3.8	65
Nyabihu	13.0	52.6	9.7	67
Ngororero	3.6	24.8	21.6	88
Rusizi	15.5	34.0	28.5	83
Nyamasheke	15.0	14.4	14.1	94
Rulindo	10.4	16.4	5.9	82
Gakenke	8.8	40.0	9.6	85
Musanze	10.3	7.6	1.6	91
Burera	8.3	15.5	5.8	81
Gicumbi	8.9	14.6	8.7	96
Rwamagana	8.2	9.4	12.9	76
Nyagatare	7.6	24.4	17.9	113
Gatsibo	10.8	12.9	8.0	102
Kayonza	14.3	20.3	16.4	83
Kirehe	22.1	30.8	14.9	86
Ngoma	9.6	31.3	11.8	84
Bugesera	18.9	18.8	12.0	84
Rwanda	12.5	20.7	11.6	2,322

NISR, AHS 2020

Table 77: Percentage of agricultural HHs with at least one member having a bank account

District	Percentage of agricultural HHs with at least one member having a bank account	Number of agricultural households(,000)
Nyarugenge	66.9	20
Gasabo	69.6	49
Kicukiro	57.4	17
Nyanza	58.2	84
Gisagara	55.3	91
Nyaruguru	78.0	68
Huye	61.9	72
Nyamagabe	70.9	84
Ruhango	53.3	76
Muhanga	55.0	72
Kamonyi	51.4	85
Karongi	60.1	73
Rutsiro	61.9	73
Rubavu	57.9	65
Nyabihu	58.2	67
Ngororero	64.2	88
Rusizi	53.8	83
Nyamasheke	63.6	94
Rulindo	70.7	82
Gakenke	58.9	85
Musanze	52.8	91
Burera	56.3	81
Gicumbi	57.2	96
Rwamagana	59.2	76
Nyagatare	45.4	113
Gatsibo	41.4	102
Kayonza	51.6	83
Kirehe	57.1	86
Ngoma	54.0	84
Bugesera	64.0	84
Rwanda	58.1	2,322

NISR, AHS 2020

Table 78: Percentage of agricultural households/farmers who did savings by District

District	Percentage of agricultural HHs with at least one member who made savings	Number of agricultural households (,000)
Nyarugenge	76.2	20
Gasabo	78.4	49
Kicukiro	62.6	17
Nyanza	70.2	84
Gisagara	68.8	91
Nyaruguru	67.1	68
Huye	79.7	72
Nyamagabe	75.0	84
Ruhango	75.4	76
Muhanga	67.4	72
Kamonyi	74.9	85
Karongi	55.6	73
Rutsiro	43.7	73
Rubavu	60.4	65
Nyabihu	77.4	67
Ngororero	62.8	88
Rusizi	69.6	83
Nyamasheke	30.4	94
Rulindo	74.5	82
Gakenke	51.2	85
Musanze	67.7	91
Burera	67.7	81
Gicumbi	75.0	96
Rwamagana	73.2	76
Nyagatare	72.7	113
Gatsibo	69.1	102
Kayonza	80.2	83
Kirehe	80.1	86
Ngoma	65.2	84
Bugesera	75.9	84
Rwanda	68.1	2,322

NISR, AHS 2020

STAFF WHO COTRIBUTED TO THE AGRICULTURAL HOUSEHOLD SURVEY 2020

National coordination

- MURANGWA Yusuf, Director General, NISR
- MURENZI Ivan, Deputy Director General, NISR

AHS technical coordination

- MWIZERWA Jean Claude, Director of Economic Statistics Unit
- ABAYISENGA Aimable, Agriculture and Environmental Statistics Team Leader

Sample Design

- BURAMBA Eric, Seasonal Crop Production Statistician
- ABAYISENGA Aimable, Agriculture and Environmental Statistics Team Leader

AHS fieldwork coordination

- BURAMBA Eric, Seasonal Crop Production Statistician
- KAMANZI SHINGIRO Jean Philbert, Research assistant
- MUKAMAZIMPAKA Francine, Perennial crops and horticulture statistician
- MUREBWAYIRE Divine, Research assistant
- UWIZEYIMANA Lambert, Forestry and Environmental statistics statistician

Data analysis

- ABAYISENGA Aimable, Agriculture and Environmental Statistics Team Leader
- BURAMBA Eric, Seasonal Crop Production Statistician
- MUTEBUTSI Alexis, Research assistant
- MUVUNYI Issa, Livestock and Fisheries Production Statistician
- UWAMAHORO Didas, Regional Price and Economic data collector Officer
-RWAYITARE Jean Bosco, Research assistant

Report writing

-BURAMBA Eric, Seasonal Crop Production Statistician
-MUTEBUTSI Alexis, Research assistant
-UWAMAHORO Didas, Regional price and economic data collector officer

AHS GIS team

- BIZIMUNGU Clément, Field operations Cartographer Officer
- NTAWIHA Athanasie, GIS staff
- IRAMBONA Eddy Marcus, GIS staff
- NIYITEGEKA Beata, GIS support staff
- NDAZIGARUYE Alfred, GIS support staff
- KICONCO Jovia GIS support staff
- MUHIRE NGABO Olympe, GIS support staff

Data processing

- SEBAHIRE Jean Népomuscène, Food Security & Agriculture Surveys data processing Officer
- RWAYITARE Jean Bosco, Research assistant
- HAGENIMANA Damascène, IT Support staff
- NIYONGIRA Partick, IT Support staff
