# Supplementary material for: Genetic diversity and relationship of Bugesera and Rwamagana indigenous chicken populations with SASSO chickens using DArTseq SNPs
Source: PLoS One. 2025 Sep 12;20(9):e0331316. doi: 10.1371/journal.pone.0331316 (PMC12431124; doi:10.1371/journal.pone.0331316)
Supplement: Attachment — Submitted filename: Review for PONE-D-24-58510.docx [file pone.0331316.s009.docx]

**Overall impression:**

I really enjoyed reading this paper! The authors did an excellent job explaining the significance of their work. This manuscript effectively demonstrates the genetic diversity of indigenous chickens from various districts in Rwanda in comparison to SASSO chickens.

The authors successfully contextualized the study’s relevance to Rwanda and identified existing knowledge gaps. Their expertise and the necessity of this research were evident throughout the paper. They also provided a clear understanding of the genetic relationships between the two indigenous chicken populations and the SASSO chickens, offering valuable insights into the effectiveness of breeding methods and their implications for breeding conservation programs.

**Introduction:**

In the introduction, the statistics on the current contribution of livestock are well-presented and convincing. However, I would like to see an update on the current state of affairs, particularly regarding the impact of the Rwanda Livestock Master Plan (LMP), implemented in 2017 to improve the supply of quality breeding stock to farmers.

Since at least five studies have been published after the LMP's implementation, it would be helpful to include findings from these studies. Specifically, how do the current statistics compare to previous levels of inbreeding? Have these studies demonstrated a measurable improvement in genetic variability among the birds? Including this information would provide a more comprehensive understanding of the program’s effectiveness.

**Materials**

The study area and sampling procedure were clearly explained, providing insight into why birds were collected from specific districts and populations. Understanding the economic significance of these districts to rural households is crucial, as well as recognizing how traditional breeding practices among rural farmers influence genetic variability. These factors strengthen the study’s relevance and highlights the broader implications of genetic diversity in indigenous chicken populations. Also, the sample size used in the study—87 DNA samples genotyped using Diversity Array Technology—was substantial and provided valuable insights into the genetic diversity and relationships among the three populations.

- A question for clarity: Why were the indigenous chickens (IC) sampled at ages 8 to 10 months, while only female SASSO chickens were sampled precisely at 9 months of age?

**Results:**

The results section provides a comprehensive and well-structured analysis of key genetic aspects of the study. The selection of 8,087 DArTseq SNP markers from the original 45,677 DArTseq SNP markers after quality control is effectively presented. The study demonstrates high-quality marker selection which enhances the reliability of the findings.

The assessment of genetic distance within populations and the analysis of genetic dissimilarities among populations are thorough, providing valuable insights into the genetic distinctions and similarities among the studied groups. The analysis of population structure was well-defined, showing the clustering patterns and genetic differentiation among the indigenous and SASSO chickens.

**Discussion:**

I was very pleased with the discussion section of the manuscript. I particularly appreciated how it outlines effective strategies for implementing breeding and conservation programs, which are well-supported by the study’s findings. The discussion highlights key genetic factors such as increased inbreeding, co-ancestry mating, and high genetic distance, reinforcing the importance of strategic breeding interventions.

Also, the discussion effectively demonstrates how geographical proximity influences genetic relatedness between populations. The manuscript further provides valuable insights into the areas that require attention when planning breeding and conservation strategies, offering clear explanations on how these strategies can be successfully implemented.

- Towards the end of the discussion on genetic variability and genetic relationships between chicken populations, you briefly mention how the absence of intensive selection within native chicken populations leads to inbreeding, contributing to genetic variation between the two types of chickens. However, I found this part somewhat unclear, especially in relation to your earlier point about mating systems in the study area potentially not being random. It might be helpful to elaborate further on this connection as I was unsure about the exact argument being made and the direction you were leading us toward in this discussion.

**Miscellaneous remarks:**

- I suggest mentioning the authors' names when referencing them in-text before including the numbered citation. Using only a number, such as (1), disrupts the readability of the sentence. For example, instead of writing:

*"There was an increase in poultry production, reported in 2014 by (2),"*

it would read more smoothly as:

*"There was an increase in poultry production, reported in 2014 by Sharpio (2)."*

- There was an inconsistency in the in-text citations, as some were numbered while others were not. I noticed this particularly in the Methods section.
- Throughout the manuscript, the number of chicken genotypes (87 or 86) was inconsistently written as "eight-seven" instead of "eighty-seven" or "eighty-six." I recommend correcting this for clarity and consistency.
- In the Abstract, the authors introduced low He, assuming readers would immediately recognize the term. To avoid confusion or unnecessary assumptions, I suggest replacing it with "expected heterozygosity (He)" for clarity right from the start of the paper.
- Do not start a sentence with an abbreviation; instead, write it out in full when beginning a sentence.
- Please ensure that page numbers are included in the manuscript.
